# Supplementary material for: Characterising COVID-19 empirical research production in Latin America and the Caribbean: A scoping review
Source: PLoS One. 2022 Feb 16;17(2):e0263981. doi: 10.1371/journal.pone.0263981 (PMC8849471; doi:10.1371/journal.pone.0263981)
Supplement: S1 File — (DOCX) [file pone.0263981.s002.docx]

**S1 Supporting information**

# Search strategies

Search strategy used to search articles in OVID (Medline and EMBASE)

| **#** | **QUERY** |
| --- | --- |
| 1 | ((latin or south or central or north or hispanic) adj america*) or carib* |
| 2 | exp Latin America/ or exp Caribbean Region/ |
| 3 | Belize* or "costa rica" or "costa rican" or Salvador* or Guatemala* or hondura* or mexic* or Nicaragua* or panama* or argentin* or Bolivia* or brazil* or Colombia* or chile* or Ecuador* or Guiana* or guyan* or Paraguay* or peru* or suriname* or Uruguay* or Venezuela* or cuba* or Haiti or "Puerto rico" or "Puerto rican" or Anguill* or Aruba* or Barbad* or Bermud* or "Cayman Islands" or Caymanian or Grenad* or Jamaica* or Antilles or "Saint Lucia" or "saint Lucian" or "Turks and Caicos" or Antigua* or Baham* or Dominica* or "virgin islands" or "virgin islanders" or Monstserrat* or "Saint Kitts and Nevis" or kittitian or Nevisian or "Saint Vincent and the Grenadines" or Trinidad* or Tobago* or trinis or trinbagonian or Curacao* or Guadeloupe* or Martiniqu* or "Sint Maarten" or "saint martin" or "saint martinois" |
| 4 | Coronavir* or 2019-nCoV or 2019nCoV or nCoV or SARS-CoV-2 or COVID19 or COVID-19 or “severe acute respiratory syndrome” or SARS |
| 5 | 1 or 2 or 3 |
| 6 | 4 and 5 |
| 7 | limit 6 to yr=”2019-Current” |

Search strategy used to search articles in LILACS (using VHL)

| **#** | **QUERY** |
| --- | --- |
| 1 | ((latin OR south OR central OR north OR hispanic) AND america*) OR carib* OR belize* OR "costa rica" OR "costa rican" OR salvador* OR guatemala* OR hondura* OR mexic* OR nicaragua* OR panama* OR argentin* OR bolivia* OR brazil* OR colombia* OR chile* OR ecuador* OR guiana* OR guyan* OR paraguay* OR peru* OR suriname* OR uruguay* OR venezuela* OR cuba* OR haiti OR "Puerto rico" OR "Puerto rican" OR anguill* OR aruba* OR barbad* OR bermud* OR "Cayman Islands" OR caymanian OR grenad* OR jamaica* OR antilles OR "Saint Lucia" OR "saint Lucian" OR "Turks and Caicos" OR antigua* OR baham* OR dominica* OR "virgin islands" OR "virgin islanders" OR monstserrat* OR "Saint Kitts and Nevis" OR kittitian OR nevisian OR "Saint Vincent and the Grenadines" OR trinidad* OR tobago* OR trinis OR trinbagonian OR curacao* OR guadeloupe* OR martiniqu* OR "Sint Maarten" OR "saint martin" OR "saint martinois") AND (coronavir* OR 2019-ncov OR 2019ncov OR ncov OR sars-cov-2 OR covid19 OR covid-19 OR “severe acute respiratory syndrome” OR sars) |

Search strategy used to search articles in CENTRAL (the Cochrane Library)

| **#** | **QUERY** |
| --- | --- |
| 1 | ((latin or south or central or north or hispanic) and america*) or carib* |
| 2 | MeSH descriptor: [Latin America] explode all trees |
| 3 | MeSH descriptor: [Caribbean Region] explode all trees |
| 4 | Belize* or "costa rica" or "costa rican" or Salvador* or Guatemala* or hondura* or mexic* or Nicaragua* or panama* or argentin* or Bolivia* or brazil* or Colombia* or chile* or Ecuador* or Guiana* or guyan* or Paraguay* or peru* or suriname* or Uruguay* or Venezuela* or cuba* or Haiti or "Puerto rico" or "Puerto rican" or Anguill* or Aruba* or Barbad* or Bermud* or "Cayman Islands" or Caymanian or Grenad* or Jamaica* or Antilles or "Saint Lucia" or "saint Lucian" or "Turks and Caicos" or Antigua* or Baham* or Dominica* or "virgin islands" or "virgin islanders" or Monstserrat* or "Saint Kitts and Nevis" or kittitian or Nevisian or "Saint Vincent and the Grenadines" or Trinidad* or Tobago* or trinis or trinbagonian or Curacao* or Guadeloupe* or Martiniqu* or "Sint Maarten" or "saint martin" or "saint martinois" |
| 5 | coronavir* or "2019-nCoV" or 2019nCoV or nCoV or "SARS-CoV-2" or COVID19 or "COVID-19" or “severe acute respiratory syndrome” or SARS |
| 6 | 1 or 2 or 3 or 4 |
| 7 | 5 and 6 |
| 8 | with Publication Year from 2019 to present , in Trials |

Search strategy used to search articles in Scielo

| **#** | **QUERY** |
| --- | --- |
| 1 | ((latin or south or central or north or hispanic) and america*) or carib* |
| 2 | Belize* or "costa rica" or "costa rican" or Salvador* or Guatemala* or hondura* or mexic* or Nicaragua* or panama* or argentin* or Bolivia* or brazil* or Colombia* or chile* or Ecuador* or Guiana* or guyan* or Paraguay* or peru* or suriname* or Uruguay* or Venezuela* or cuba* or Haiti or "Puerto rico" or "Puerto rican" or Anguill* or Aruba* or Barbad* or Bermud* or "Cayman Islands" or Caymanian or Grenad* or Jamaica* or Antilles or "Saint Lucia" or "saint Lucian" or "Turks and Caicos" or Antigua* or Baham* or Dominica* or "virgin islands" or "virgin islanders" or Monstserrat* or "Saint Kitts and Nevis" or kittitian or Nevisian or "Saint Vincent and the Grenadines" or Trinidad* or Tobago* or trinis or trinbagonian or Curacao* or Guadeloupe* or Martiniqu* or "Sint Maarten" or "saint martin" or "saint martinois" |
| 3 | coronavir* or "2019-nCoV" or 2019nCoV or nCoV or "SARS-CoV-2" or COVID19 or "COVID-19" or “severe acute respiratory syndrome” or SARS |
| 4 | (1 or 2) AND 3 |
| 5 | Published 2019 AND 2020 |

Search strategy used to search articles in Epistemonikos

| **#** | **QUERY** |
| --- | --- |
| 1 | (title:((((latin OR south OR central OR north OR hispanic) AND america*) OR carib*) OR (Belize* OR "costa rica" OR "costa rican" OR Salvador* OR Guatemala* OR hondura* OR mexic* OR Nicaragua* OR panama* OR argentin* OR Bolivia* OR brazil* OR Colombia* OR chile* OR Ecuador* OR Guiana* OR guyan* OR Paraguay* OR peru* OR suriname* OR Uruguay* OR Venezuela* OR cuba* OR Haiti OR "Puerto rico" OR "Puerto rican" OR Anguill* OR Aruba* OR Barbad* OR Bermud* OR "Cayman Islands" OR Caymanian OR Grenad* OR Jamaica* OR Antilles OR "Saint Lucia" OR "saint Lucian" OR "Turks AND Caicos" OR Antigua* OR Baham* OR Dominica* OR "virgin islands" OR "virgin islanders" OR Monstserrat* OR "Saint Kitts AND Nevis" OR kittitian OR Nevisian OR "Saint Vincent AND the Grenadines" OR Trinidad* OR Tobago* OR trinis OR trinbagonian OR Curacao* OR Guadeloupe* OR Martiniqu* OR "Sint Maarten" OR "saint martin" OR "saint martinois")) OR abstract:((((latin OR south OR central OR north OR hispanic) AND america*) OR carib*) OR (Belize* OR "costa rica" OR "costa rican" OR Salvador* OR Guatemala* OR hondura* OR mexic* OR Nicaragua* OR panama* OR argentin* OR Bolivia* OR brazil* OR Colombia* OR chile* OR Ecuador* OR Guiana* OR guyan* OR Paraguay* OR peru* OR suriname* OR Uruguay* OR Venezuela* OR cuba* OR Haiti OR "Puerto rico" OR "Puerto rican" OR Anguill* OR Aruba* OR Barbad* OR Bermud* OR "Cayman Islands" OR Caymanian OR Grenad* OR Jamaica* OR Antilles OR "Saint Lucia" OR "saint Lucian" OR "Turks AND Caicos" OR Antigua* OR Baham* OR Dominica* OR "virgin islands" OR "virgin islanders" OR Monstserrat* OR "Saint Kitts AND Nevis" OR kittitian OR Nevisian OR "Saint Vincent AND the Grenadines" OR Trinidad* OR Tobago* OR trinis OR trinbagonian OR Curacao* OR Guadeloupe* OR Martiniqu* OR "Sint Maarten" OR "saint martin" OR "saint martinois"))) AND (title:(coronavir* OR "2019-nCoV" OR 2019nCoV OR nCoV OR "SARS-CoV-2" OR COVID19 OR "COVID-19" OR "severe acute respiratory syndrome" OR SARS) OR abstract:(coronavir* OR "2019-nCoV" OR 2019nCoV OR nCoV OR "SARS-CoV-2" OR COVID19 OR "COVID-19" OR "severe acute respiratory syndrome" OR SARS)) |
| 2 | Primary studies From 2019 |

# Data extraction fields

| **DATA SOURCES** |  |
| --- | --- |
| *Source where the data that is analyzed in the study comes from. This category is used to identify the country later* | |
| Inert sources | e.g., sewage, surfaces,air particles, etc. The paper needs to specificy that the country where the sources come from to be included |
| Animals |  |
| Directly humans | Information is directly collected from humans |
| Databases | Human information that was not directly collected from humans (i.e., someone else collected the data before the research) |
| Documents | Documentary analysis (e.g., analyses of government documents, bulletins, reports, etc.) |
| Not described | If it is not described, the study should be excluded (e.g., the study analyze molecules coming from samples that the article does not specify where do they come from) |
| Other |  |

| **BROAD METHODOLOGY PARADIGM** |
| --- |
| *High-level methodology that the study uses* |
| Basic science |
| Quantitative |
| Qualitative |
| Mixed-methods |

| **STUDY DESIGNS** |  |
| --- | --- |
| *Depending on the methodology selected, we could use several options here* |  |
| **Basic sciences** | We will not identify the study design if the study is about basic sciences for now |
| **Quantitative** |  |
| Randomized controlled trial |  |
| Non-randomized controlled trial | Patients allocated to intervention and control group without randomization |
| Controlled before and after (including diff in diff analysis) | At least two groups are compared before and after an intervention |
| Single before and after | Only one group is compared before and after an intervention/exposure |
| Interrupted time series | Time series are compared before and after the intervention is implemented. It is differently from a descriptive time series |
| Propensity score matching | Two groups are compared matching by several variables |
| Cohort study |  |
| Case-control study |  |
| Cross-sectional study |  |
| Ecological study | Where the unit of analysis is not a patient but a group of patients (e.g., comparing the rates of two different countries, cities) |
| Descriptive time series | Time series that are used to only describe trends |
| Simulation model used to predict |  |
| Validation of a simulation model |  |
| Economic evaluation | Cost-utility, cost-effectiveness, cost-benefit analysis |
| Cost study | Budget analysis, just measuring how much does it cost an intervention |
| Other |  |
| **Qualitative** |  |
| Case-study | There are single or multiple case studies, where cases (institutions, organizations, etc) are compared in their processes |
| Narrative | Study to describe the main codes coming from speaches or information collected from people |
| Ethnography |  |
| Phenomenological |  |
| Grounded theory |  |
| Other |  |
| **Mixed methods** |  |
| Exploratory | Quantitative is conducted after qualitative phase |
| Explanatory sequential | Qualitative is conducted after quantitative phase |
| Convergent | Qualitative and quantitative are conducted at the same time |
| Other |  |

| **STATUS OF PUBLICATION** |
| --- |
| *Whether or not the study has been finished or not* |
| Completed |
| Ongoing |
| Terminated |

| **WHO CLASSIFICATION** |
| --- |
| *The main research subject is classified in one of these 10 areas.* |
| Virus natural history, transmission and diagnostics |
| Animal and environmental research on the virus origin, and management measures at the human-animal interface |
| Epidemiological studies |
| Clinical management |
| Infection prevention and control, including health care workers' protection |
| Candidate therapeutics R&d |
| Candidate vaccines R&D |
| Ethics considerations for research |
| Social sciences in the outbreak response |
| Health systems arrangements |
| Other, including mental health |

| **RESEARCH QUESTION** |  |
| --- | --- |
| *Type of research question. Knowledge that we are looking from the main intervention/exposure/phenomenon of interest* |  |
| Benefits and harms of an intervention | Effectiveness or efficacy of an intervention |
| Costs and value for money | Budget impacts, economic evaluations, cost estimations,etc. |
| Magnitude of the problem | Measuring how big is a problem/issue (e.g., the impact of pandemic) |
| Views and preferences | Values and preferences of the population on a specific topic |
| How and why it works | Usually questions related to explore the mechanism of action of an intervention |
| Implementation barriers and facilitators |  |
| Feasibility | How feasible is to implement an intervention |
| Diagnostic accuracy |  |
| Prognosis | Study analyzing what factors determine the prospect of a health condition in the future (usually clinical) (e.g., does smoking increase the risk of becoming a critical COVID-19 patient?) |
| To describe a population | Prevalence, incidence on a specific population |
| Other |  |

| **TYPES OF FUNDING** |  |
| --- | --- |
| *Type of organization that is funding the research.* ***We might need to google the organization that is reported to be funding*** |  |
| Government | or government agencies |
| International organizations | e.g., WHO, PAHO, etc. |
| Universities or research centres |  |
| NGOs |  |
| Private companies | e.g., pharmaceutical companies |
| Other |  |

# Included articles

1. No Authors. Willingness to pay for a COVID-19 vaccine high in Chile. PharmacoEconomics outcomes news. 2020;858: 35. doi:10.1007/s40274-020-7008-x
2. No Authors. What is the potential for a second peak in the evolution of SARS-CoV-2 in Brazil? Insights from a SIRASD model considering the informal economy. 2020.
3. No Authors. What is the people posting about symptoms related to Coronavirus in Bogota, Colombia? 2020.
4. No Authors. Update of the Evolution of SARS-CoV-2 Infection, COVID-19, and Mortality in Mexico Until May 15, 2020: An Ecological Study. Int J Trop Dis Heal. 2020. doi:10.9734/IJTDH/2020/V41I530277
5. No Authors. The First Few Hundred Cases for Coronavirus Disease 2019 (COVID-19) in Colombia. Iran J Public Health. 2020;49. doi:10.18502/IJPH.V49IS1.3669
6. No Authors. Predicting COVID-19 distribution in Mexico through a discrete and time-dependent Markov chain and an SIR-like model. 2020.
7. No Authors. Poverty levels, societal and individual heterogeneities explain the SARS-CoV-2 pandemic growth in Latin America. 2020.
8. No Authors. PADRÕES ESPAÇOS-TEMPORAIS DA ROTA DO COVID19 (CORONAVIRUS) NO ESTADO DE SANTA CATARINA, BRASIL. Hygeia  Rev Bras Geogr Médica e da Saúde. 2020. doi:10.14393/HYGEIA0054251
9. No Authors. Modeling the transmission of new coronavirus in São Paulo State, Brazil -- Assessing epidemiological impacts of isolating young and elder persons. 2020.
10. No Authors. Modeling the early evolution of the COVID-19 in Brazil: results from a Susceptible-Infectious-Quarantined-Recovered (SIQR) model. 2020.
11. No Authors. How relevant is the decision of containment measures against COVID-19 applied ahead of time? 2020.
12. No Authors. Different scenarios in the Dynamics of SARS-Cov-2 Infection: an adapted ODE model. 2020.
13. No Authors. Data analysis and modeling of the evolution of COVID-19 in Brazil. 2020.
14. No Authors. COVID-19: estadística de morbi-mortalidad en Venezuela. 2020;5.
15. No Authors. COVID-19: A model for studying the evolution of contamination in Brazil. 2020.
16. No Authors. Clinical Management Strategies in Mild COVID-19 cases in Latin-America: A Decision Model. 2020.
17. No Authors. Cenário atual da covid-19 no estado do Ceará, Brasil. 2020;3. doi:10.31005/IAJMH.V3I0.92
18. No Authors. An Optimal Predictive Control Strategy for COVID-19 (SARS-CoV-2) Social Distancing Policies in Brazil. 2020.
19. No Authors. A utilização do planejamento territorial no combate da COVID-19: considerações sobre a situação dos leitos nos municípios de Pernambuco, Brasil. 2020;8. doi:10.22239/2317-269X.01546
20. Abrahao JS, Sacchetto L, Rezende IM, Rodrigues RAL, Crispim APC, Moura C, et al. Detection of SARS-CoV-2 RNA on public surfaces in a densely populated urban area of Brazil: A potential tool for monitoring the circulation of infected patients. Sci Total Environ. 2020; 142645. doi:http://dx.doi.org/10.1016/j.scitotenv.2020.142645

Jonatas Santos A, Livia Sacchetto P, Izabela Mauricio R, Rodrigo R, Ana Paula Correia C, Cesar M, et al. Detection of SARS-CoV-2 RNA on public surfaces in a densely populated urban area of Brazil. medRxiv. 2020. doi:10.1101/2020.05.07.20094631

1. Acosta G, Escobar G, Bernaola G, Alfaro J, Taype W, Marcos C, et al. Description of patients with severe COVID-19 treated in a national referral hospital in Peru. Rev peru med exp salud publica. 2020;37: 253–258. doi:10.17843/RPMESP.2020.372.5437
2. Acosta LD. [Response capacity to the COVID-19 pandemic in Latin America and the Caribbean]. Rev Panam Salud Publica. 2020;44: e109. doi:10.26633/RPSP.2020.109
3. Acosta LD. Capacidad de respuesta frente a la pandemia de COVID-19 en América Latina y el Caribe. 2020.
4. Acuna-Zegarra MA, Santana-Cibrian M, Velasco-Hernandez JX. Modeling behavioral change and COVID-19 containment in Mexico: A trade-off between lockdown and compliance. Math Biosci. 2020;325: 108370. doi:https://dx.doi.org/10.1016/j.mbs.2020.108370
5. Adan S-M, Jose Roberto Balmori de la M, Lauren H-V. Families under Confinement: COVID-19, Domestic Violence, and Alcohol Consumption. SSRN. 2020. doi:10.2139/ssrn.3688384
6. Adan S-M, Lauren H-V, Jose Roberto Balmori de la M, Abel R. COVID-19 and Women’s Health: Examining Changes in Mental Health and Fertility. SSRN. 2020. doi:10.2139/ssrn.3656596
7. Adrian S-M, Braulio AMG, Erick Martinez R, Jose Omar Barreto R, Alicia Estela Lopez R, Paolo Alberti M, et al. THE LOW-HARM SCORE FOR PREDICTING MORTALITY IN PATIENTS DIAGNOSED WITH COVID-19: A MULTICENTRIC VALIDATION STUDY. medRxiv. 2020. doi:10.1101/2020.05.26.20111120
8. Agenor De  Jr. N, Bernando Araldi da S, Felipe D-P, Luismar Marques P. A two-wave epidemiological model of COVID-19 outbreaks using MS-Excel(R). medRxiv. 2020. doi:10.1101/2020.05.08.20095133
9. Agostinho Antônio Cruz A, Jackeline Vieira A, Juliana do Nascimento S, Maria Clara Santos F, Camila de Meneses Caetano V, Pedro Henrique Moraes M, et al. COVID-19: ANALYSIS OF CONFIRMED CASES IN TERESINA, PIAUI, BRAZIL. 2020;6. doi:10.26694/REPIS.V6I0.10569
10. Aguiar S, Baiocchi G, Duprat JP, Coimbra FJF, Makdissi FB, Vartanian JG, et al. Value of preoperative testing for SARS-CoV-2 for elective surgeries in a cancer center during the peak of pandemic in Brazil. J Surg Oncol. 2020. doi:10.1002/jso.26146
11. Ahmed Sameer El K. Aspectos Psicocomportamentais durante a Pandemia da COVID-19: Uma análise dos efeitos provocados em moradores da região central de São Paulo. (Psychobehavioral Aspects during the COVID-19 Pandemic: An Analysis of the Effects Caused in Residents of the Cen. SSRN. 2020. doi:10.2139/ssrn.3612785
12. Ahumada PSM. Social isolation and quarantine effects over the subjective well-being of Chileans during the COVID-19 outbreak. 2020.
13. Akira O, Yusuke N, Tokuro F. Universality in COVID-19 spread in view of the Gompertz function. medRxiv. 2020. doi:10.1101/2020.06.18.20135210
14. Alberto F, Matther S-K. Factors linked to changes in mental health outcomes among Brazilians in quarantine due to COVID-19. medRxiv. 2020. doi:10.1101/2020.05.12.20099374
15. Alberto F, Matthew S-K. The Relationship Between Behavioural and Psychosocial Factors Among Brazilians in Quarantine Due to COVID-19. SSRN. 2020. doi:10.2139/ssrn.3566245
16. Alberto P-M, Marina M, Carolina F, Sergio G, Angelica R, Lisseth P, et al. SARS-CoV-2 spread across the Colombian-Venezuelan border. medRxiv. 2020. doi:10.1101/2020.07.09.20149856
17. Albitres-Flores L, Pisfil-Farroñay YA, Guillen-Macedo K, Niño-Garcia R, Alarcon-Ruiz CA. Perception of medical interns about the internship during the COVID-19 pandemic in Peru. 2020.
18. Alcibiades V, Giselle R, Xu Z, Digna W, Carolina De La G, Gabrielle B, et al. Performance of a point of care test for detecting IgM and IgG antibodies against SARS-CoV-2 and seroprevalence in blood donors and health care workers in Panama. medRxiv. 2020. doi:10.1101/2020.09.25.20201459
19. Aldo C, Marcel G, Eduardo L, Marcelo O, Gabriel YW, Julio C, et al. The Social Divide of Social Distancing: Lockdowns in Santiago during the COVID-19 Pandemic. SSRN. 2020. doi:10.2139/ssrn.3691373
20. Alejandra C-M, Carlos MG-L, Mercedes A, Ana Cristina S, Hector L-F. Municipality- level predictors of COVID-19 mortality in Mexico: a cautionary tale. medRxiv. 2020. doi:10.1101/2020.07.11.20151522
21. Alejandro C-M, Reinaldo Andres R-S. CCOFEE-GI Study: Colombian COVID19 First Experience in Gastroentrology. Characterization of digestive manifestations in patients diagnosed with COVID-19 at a highly complex institution in Bogota D.C., Colombia. medRxiv. 2020. doi:10.1101/2020.07.24.20161604
22. Alejandro L-F, David H, Fernanda M-P. Air Pollution Exposure and COVID-19: A Look at Mortality in Mexico City Using Individual-Level Data. SSRN. 2020. doi:10.2139/ssrn.3673616
23. Alejandro M-S, Carlos AF-M, Neftali Eduardo A-V, Arsenio V-V, Enrique CG, Alejandro C-M, et al. Adaptive responses to SARS-CoV-2 infection linked to accelerated aging measures predict adverse outcomes in patients with severe COVID-19. medRxiv. 2020. doi:10.1101/2020.11.03.20225375
24. Alejandro M, Alejandra S, Adriana A, Carolina G, Yazmin R, Javier G, et al. Socio-demographic and Clinical Characteristics of Adults with SARS-CoV-2 Infection in Two Hospitals in Bogota, Colombia. medRxiv. 2020. doi:10.1101/2020.08.13.20167445
25. Alejandro R, Alfredo M, Gabriela BS, Patricia MG, Juan ES, Jose MC. A numerical simulation of the COVID-19 epidemic in Argentina using the SEIR model. arXiv Popul Evol. 2020.
26. Alejandro T, María P, Felipe V, Luis Ruso M, Gustavo Rodríguez T, Mauro P, et al. COVID 19 en fase 2 sostenida. Experiencia quirúrgica inicial en el Hospital Maciel. 2020;4. doi:10.31837/CIR.URUG.4.2.6
27. Alessandro CP, Paula de Castro P, Daiane FDL, Alexandre VS, Marco R, Cezar VWR, et al. COVID-19 Seroprevalence in Military Police Forces, Southern Brazil. SSRN. 2020. doi:10.2139/ssrn.3697174
28. Alessi J, De Oliveira GB, Franco DW, Brino Do Amaral B, Becker AS, Knijnik CP, et al. Mental health in the era of COVID-19: Prevalence of psychiatric disorders in a cohort of patients with type 1 and type 2 diabetes during the social distancing. Diabetol Metab Syndr. 2020;12: 76. doi:http://dx.doi.org/10.1186/s13098-020-00584-6
29. Alger J, Cafferata ML, Alvarado T, Ciganda A, Corrales A, Desale H, et al. Using Prenatal Blood Samples to Evaluate COVID-19 Rapid Serologic Tests Specificity. Matern Child Health J. 2020;24: 1099–1103. doi:10.1007/s10995-020-02981-9
30. Aliaga-Castillo V, Fuentes Vejar B, Muñoz Campos J. Descriptive report of self-reported cases of physiotherapists infected by COVID-19 in Chile. Kinesiologia. 2020;39: 8–13.
31. Almeida ALC, Santo T, Mello MSS, Cedro A V, Lopes NL, Ribeiro A, et al. Repercussions of the COVID-19 Pandemic on the Care Practices of a Tertiary Hospital. Arq Bras Cardiol. 2020. doi:10.36660/abc.20200436
32. Almeida J dos S, Cardoso JA, Cordeiro EC, Lemos M, Araújo TME de, Sardinha AH de L. CARACTERIZAÇÃO EPIDEMIOLÓGICA DOS CASOS DE COVID-19 NO MARANHÃO: Uma breve análise. 2020.
33. Almeida S, Villibor C, Carstensen S, Petterle R. Proposal and psychometric validation of the Severe Acute Respiratory Syndrome - Coronavirus-2 Fear Scale (SCoV-2-FS). ResearchSquare. 2020. doi:10.21203/rs.3.rs-48227/v1
34. Almeida W da S de, Szwarcwald CL, Malta DC, Barros MB de A, Souza Júnior PRB de, Azevedo LO, et al. Changes of Brazilians’ socioeconomic and health conditions during the COVID-19 pandemic. 2020.
35. Alomo M, Gagliardi G, Peloche S, Somers E, Alzina P, Prokopez CR. [Psychological effects during the COVID-19 outbreak in Argentina]. Efectos Psicol la pandemia COVID-19 en la Poblac Gen Argentina. 2020;77: 176–181. doi:https://dx.doi.org/10.31053/1853.0605.v77.n3.28561
36. Altair Souza De A, Vinicius JOW de C. Logistic Approach to COVID - 19 Epidemic Evolution in Brazil. medRxiv. 2020. doi:10.1101/2020.06.22.20135921
37. Alvarez-Diaz DA, Franco-Munoz C, Laiton-Donato K, Usme-Ciro JA, Franco-Sierra ND, Florez-Sanchez AC, et al. Molecular analysis of several in-house rRT-PCR protocols for SARS-CoV-2 detection in the context of genetic variability of the virus in Colombia. Infect Genet Evol. 2020;84: 104390. doi:https://dx.doi.org/10.1016/j.meegid.2020.104390
38. Alvarez-Lopez DI, Espinoza-Molina MP, Cruz-Loustaunau ID, Alvarez-Hernandez G. Diabetes and hypertension as factors associated with Covid-19 lethality in Sonora, Mexico, 2020. Salud Publica Mex. 2020;62: 456–457. doi:http://dx.doi.org/10.21149/11546
39. Alvaro Francisco Lopes de S, Layze Braz O, Guilherme S, Artur Acelino Francisco Luz Q, Herica Emilia Felix de C, Telma Maria Evangelista de A, et al. Casual sex among MSM during the period of social isolation in the COVID-19 pandemic: Nationwide study in Brazil and Portugal. medRxiv. 2020. doi:10.1101/2020.06.07.20113142
40. Alvaro Q-A, Oscar E, Marcela MM-R, Diana W, Diana Carolina M. Analysis of the interventions adopted due to the COVID-19 on ARI morbility for Colombia. medRxiv. 2020. doi:10.1101/2020.09.12.20193334
41. Alves JG, Ferreira Lima TP. International air traffic and COVID-19 geographical incidence in Brazil. J Microbiol Immunol Infect. 2020. doi:http://dx.doi.org/10.1016/j.jmii.2020.09.009
42. Alves THE, Souza TA de, Silva S de A, Ramos NA, Oliveira SV de. Analysis of home and hospital deaths from respiratory and cardiovascular causes during the COVID-19 pandemic in Minas Gerais. 2020.
43. Amador-Jiménez M, Millner N, Palmer C, Pennington RT, Sileci L. The Unintended Impact of Colombia’s Covid-19 Lockdown on Forest Fires. Environ Resour Econ. 2020; 1–25. doi:10.1007/s10640-020-00501-5
44. Amaral-Prado HM, Borghi F, Mello T, Grassi-Kassisse DM. The impact of confinement in the psychosocial behaviour due COVID-19 among members of a Brazilian university. Int J Soc Psychiatry. 2020; 20764020971318. doi:10.1177/0020764020971318
45. Amna T, Eduardo AU, Carla Castillo L, Katia V-G, Ruiyan L, Richard R, et al. Early transmission dynamics of COVID-19 in Chile: From sub-exponential ascending growth dynamics to a stationary disease wave, March-April, 2020. medRxiv. 2020. doi:10.1101/2020.05.15.20103069
46. Amorim Filho L, Szwarcwald CL, Mateos SOG, Leon A, Medronho RA, Veloso VG, et al. Seroprevalence of anti-SARS-CoV-2 among blood donors in Rio de Janeiro, Brazil. Rev Saude Publica. 2020;54: 69. doi:10.11606/s1518-8787.2020054002643

Amorim Filho L, Szwarcwald CL, Mateos S de OG, Leon ACMP de, Medronho R de A, Veloso VG, et al. Seroprevalence of IgG and IgM anti-SARS-CoV-2 among voluntary blood donors in Rio de Janeiro, Brazil. 2020. p. 19.

1. Amrita R, Katherine R, Brooke J, Benjamin A, Sara W, Julia M, et al. Potential interruptions in HIV prevention and treatment services for gay, bisexual, and other men who have sex with men associated with COVID-19. medRxiv. 2020. doi:10.1101/2020.08.19.20178285
2. Ana Jessica P, Diego R, Sofia Mendes S, Kamila M, Karina B, Ana CMR, et al. Increased prolonged sitting in rheumatoid arthritis patients during the COVID-19 pandemic: a within-subjects, accelerometer-based study. medRxiv. 2020. doi:10.1101/2020.09.09.20191395
3. Ana Karolina Antunes E, Meriane D, Juliana Schons G, Alana Witt H, Karoline S, Larissa M, et al. Comparison Of Different Kits For SARS-CoV-2 RNA Extraction Marketed In Brazil. bioRxiv. 2020. doi:10.1101/2020.05.29.122358
4. Ana Maria Baptista M, Cesar GV, Fernando PH, Mariangela FS, Bernardo LH, Aluisio JDB, et al. High prevalence of symptoms among Brazilian subjects with antibodies against SARS-CoV-2: a nationwide household survey. medRxiv. 2020. doi:10.1101/2020.08.10.20171942
5. Ana O-G, Erwin C, Jesus A-D, Carlos G-C, Adrián G-Á, Raúl R-R, et al. Sex Differences in the Outcome of Patients with Confirmed SARS-CoV-2 Infection. SSRN. 2020. doi:10.2139/ssrn.3624222
6. Anacleto MA, Brito FA, de Queiroz AR, Passos E, Santos JRL. Diffusive process under Lifshitz scaling and pandemic scenarios. Physica A. 2020;559: 125092. doi:10.1016/j.physa.2020.125092
7. Analía R, Andrea Elvia María B, Pilar BB, Camila Soledad D, Melisa Adriana L, Martina P, et al. Epidemiological characteristics of the first 116 974 cases of COVID-19 in Argentina, 2020. Rev argent salud publica. 2020;12: 1–9.
8. Anderson Castro Soares de O, Lia Hanna Martins M, Eveliny Barroso da S, Daniele Cristina Tita G, Luiz Andre Ribeiro Z, Cor Jesus Fernandes F. Bayesian modeling of COVID-19 cases with a correction to account for under-reported cases. medRxiv. 2020. doi:10.1101/2020.05.24.20112029
9. Anderson Walter Costa S. Epidemiologic profile and social determinant of COVID-19 in Macapá, Amapá, Amazon, Brazil. 2020;04. doi:10.32749/NUCLEODOCONHECIMENTO.COM.BR/HEALTH/COVID-19-IN-MACAPA
10. Andrade EF, Pereira LJ, Oliveira APL, Orlando DR, Alves DAG, Guilarducci JS, et al. Perceived fear of COVID-19 infection according to sex, age and occupational risk using the Brazilian version of the Fear of COVID-19 Scale. Death Stud. 2020; 1–10. doi:http://dx.doi.org/10.1080/07481187.2020.1809786
11. Andrade JV, Moraes RCC. What has the Coronavirus take from us? Potential years of life lost in Minas Gerais. J nurs Heal. 2020;10: 20104014.
12. Andrade LA, Gomes DS, Goes MAO, Souza MSF, Teixeira DCP, Ribeiro CJN, et al. Surveillance of the first cases of COVID-19 in Sergipe using a prospective spatiotemporal analysis: the spatial dispersion and its public health implications. Rev Soc Bras Med Trop. 2020;53: e20200287. doi:http://dx.doi.org/10.1590/0037-8682-0287-2020
13. Andre Filipe de Moraes B, Joao Luiz M, Thiago Henrique Rizzi D, Alexandre Dias Porto Chiavegatto F. COVID-19 diagnosis prediction in emergency care patients: a machine learning approach. medRxiv. 2020. doi:10.1101/2020.04.04.20052092
14. Andre Z, Andréa Burmeister Morais H, Bernardo F, Claudionor Oliveira Gomes J, Cristian Rogério F, Frederico Golbspan S, et al. How Do Networking Capabilities Support SMEs in Surpassing the COVID-19 Crisis? Lessons Learned from Small Firms in a Developing Country. SSRN. 2020. doi:10.2139/ssrn.3669248
15. Andreia Faranha da C, Alan Patricio da S, Luana Dias de V, Marjorie Heloise M, Andreia Zarzour Abou Hala C. O acesso vertiginoso na aquisição de máscaras faciais e suas repercussões na saúde pública e de populações vulneráveis/The vertiginous access in the acquisition of face masks and their repercussions on pubic health and vulnerable populations. 2020;4.
16. Andreia C de M, Luiz Claudio ST, Jesse L da S, Lucas Z de A, Ana Carla P, Luciana ORR, et al. Cancer inpatient with COVID-19: a report from the Brazilian National Cancer Institute. medRxiv. 2020. doi:10.1101/2020.06.27.20141499

De Melo AC, Thuler LCS, Da Silva JL, De Albuquerque LZ, Pecego AC, Rodrigues LDOR, et al. Cancer inpatients with COVID-19: A report from the Brazilian National Cancer Institute. PLoS One. 2020;15: e0241261. doi:http://dx.doi.org/10.1371/journal.pone.0241261

1. Andres IV-O, Juliana Villanueva C, Silvana Zapata B, Zulma MC. Impact of contact tracing on COVID-19 mortality: An impact evaluation using surveillance data from Colombia. medRxiv. 2020. doi:10.1101/2020.08.14.20158535
2. Andres M, Pieter S, Alethea D, Vilma R, Maria Jose Torres H, Alicia L. Impact of COVID-19 on Maternal Mental Health in a Fragile and Conflict-Affected Setting: A Longitudinal Case-Control Study in Tumaco, Colombia. SSRN. 2020. doi:10.2139/ssrn.3706751
3. Andrés N, Gastón V-H. On the dynamics of the Coronavirus epidemic and the unreported cases: the Chilean case. arXiv Popul Evol. 2020.
4. Angel Jose P-C, Marc C, Christian G-C, Guido C-E, José R-S, Carmelo D, et al. Social interventions can lower COVID-19 deaths in middle-income countries. medRxiv. 2020. doi:10.1101/2020.04.16.20063727
5. Angel Santillan H. Caracterización epidemiológica de covid-19 en Ecuador. 2020;3. doi:10.31005/IAJMH.V3I0.99
6. Ángel GCP, Eric A-V, Ugo Avila-Ponce de L. A data driven analysis and forecast of an SEIARD epidemic model for COVID-19 in Mexico. arXiv Popul Evol. 2020.
7. Angela Araújo M, Amanda Stefani Torquato da S, Regiane Lima Gasques P. Implantação do protocolo de manejo de corpos pós-óbito no contexto do novo Coronavírus. 2020;10.
8. Anibal AT, Gabriel C, Ronald Prado de la G, Carolina H, Giovanny H, Luis MP, et al. Epidemiological characterization of asymptomatic carriers of COVID-19 in Colombia. medRxiv. 2020. doi:10.1101/2020.06.18.20134734
9. Annaelise Fritz M, Bruno Barbosa S, Teresa D, Miguel Barbosa R, Francisca Braga C. Empreendedorismo Social, Inovação e Benchmarking no Instagram para combater os efeitos negativos do COVID-19 numa visão luso-brasileira. 2020;6.
10. Antônio Augusto Moura da S, Lídio Gonçalves Lima N, Conceição de Maria Pedrozo e Silva de A, Léa Márcia Melo da C, Maylla Luana Barbosa Martins B, Allan Kardec Duailibe Barros F, et al. Population-based seroprevalence of SARS-CoV-2 is more than halfway through the herd immunity threshold in the State of Maranhao, Brazil. medRxiv. 2020. doi:10.1101/2020.08.28.20180463
11. Antonio da Silva S, Antonio  Jr. P. Estimation and monitoring of COVID-19 transmissibility from publicly available data. medRxiv. 2020. doi:10.1101/2020.05.24.20112128
12. Antonio CCG, Karla Santa Cruz C, Kathleen Tereza da C, Bárbara Soares de Oliveira S, Janimayri Forastieri de A, Gustavo Fialho C, et al. COMPARATIVE ANALYSIS OF COVID-19 MORTALITY IN BRAZIL, RIO DE JANEIRO, CAMPOS DOS GOYTACAZES, MACAE, CABO FRIO AND RIO DAS OSTRAS. medRxiv. 2020. doi:10.1101/2020.09.17.20196444
13. Antunes BB de P, Peres IT, Baiao FA, Ranzani OT, Bastos LDSL, Silva A de AB da, et al. Progression of confirmed COVID-19 cases after the implementation of control measures. Rev Bras Ter intensiva. 2020. doi:https://dx.doi.org/10.5935/0103-507x.20200028

Igor Tona P, Fernanda Araújo B, Otavio Tavares R, Leonardo Dos Santos Lourenço B, Amanda de Araújo Batista da S, Guilherme Faveret Garcia de S, et al. Progressão dos casos confirmados de COVID-19 após implantação de medidas de controle./ Progressão dos casos confirmados de COVID-19 após implantação de medidas de controle./ Progression of confirmed COVID-19 cases after the implementation of control measu. Rev Bras Ter intensiva. 2020.

1. Antunez-Montes OY, Escamilla MI, Figueroa-Uribe AF, Arteaga-Menchaca E, Lavariega-Sarachaga M, Salcedo-Lozada P, et al. COVID-19 and Multisystem Inflammatory Syndrome in Latin American Children: A Multinational Study. Pediatr Infect Dis J. 2020. doi:http://dx.doi.org/10.1097/INF.0000000000002949
2. Aranda-Abreu GE, Aranda-Martinez JD, Araujo R, Hernandez-Aguilar ME, Herrera-Covarrubias D, Rojas-Duran F. Observational study of people infected with SARS-Cov-2, treated with amantadine. Pharmacol Reports. 2020. doi:http://dx.doi.org/10.1007/s43440-020-00168-1
3. Arantes VN, Martins BC, Seqatto R, Milhomen-Cardoso DM, Franzini TP, Zuccaro AM, et al. Impact of coronavirus pandemic crisis in endoscopic clinical practice: Results from a national survey in Brazil. Endosc Int open. 2020;8: E822–E829. doi:https://dx.doi.org/10.1055/a-1183-3324
4. Araújo Neto RA de, Melo GC de. Correlation between weather, population size and COVID-19 pandemic: a study of Brazilian capitals. J Heal Biol Sci. 2020;8: 1–5.
5. Araujo DB, Machado RRG, Amgarten DE, Malta FM, de Araujo GG, Monteiro CO, et al. SARS-CoV-2 isolation from the first reported patients in Brazil and establishment of a coordinated task network. Mem Inst Oswaldo Cruz. 2020;115: e200342. doi:http://dx.doi.org/10.1590/0074-02760200342
6. Araujo EM, Lilly Caldwell K, Pereira Alves dos Santos M, Magalhães de Souza I, Lima Ferreira Santa Rosa P, Beatriz Silva dos Santos A, et al. COVID-19 morbimortality by race/skin color/ethnicity: the experience of Brazil and the United States. 2020.
7. Araujo OR, Almeida CG, Lima-Setta F, Prata-Barbosa A, Colleti Junior J. The Impact of the Novel Coronavirus on Brazilian PICUs. Pediatr Crit Care Med. 2020. doi:http://dx.doi.org/10.1097/PCC.0000000000002583
8. Ardila-Sierra A, Niño-Leal L, Rivera-Triana D, Sarmiento-Medina MI, Alzate JP. Underlying conditions on the southern border between Colombia and Venezuela to face the COVID-19 pandemic. Rev salud pública. 2020;22: e486366–e486366.
9. Arellano-Llamas AA, HernÁNdez-Caballero Ál. COVID-19 en niños y adolescentes mexicanos hasta el 10 de mayo de 2020. Enfoque en pacientes con diabetes. Rev Mex Endocrinol Metab y Nutr. 2020;7: 80–86. doi:10.24875/RME.20000055
10. Arias Velásquez RM, Mejía Lara J V. Gaussian approach for probability and correlation between the number of COVID-19 cases and the air pollution in Lima. Urban Clim. 2020;33: 100664. doi:10.1016/j.uclim.2020.100664
11. Arias-Reyes C, Zubieta-DeUrioste N, Poma-Machicao L, Aliaga-Raudan F, Carvajal-Rodriguez F, Dutschmann M, et al. Does the pathogenesis of SAR-CoV-2 virus decrease at high-altitude? Respir Physiol Neurobiol. 2020;277: 103443. doi:10.1016/j.resp.2020.103443
12. Arizpe-Bravo D, Arizpe-Bravo A, Leon RB, Chacon-Cano R, Arizpe-Vilana D, Anthon F, et al. EXPERIENCE WITH COVID-19 CRITICALLY ILL PATIENTS IN A PRIVATE COMMUNITY HOSPITAL IN PUEBLA CITY, MEXICO. Chest. 2020;158: A2482–A2483. doi:http://dx.doi.org/10.1016/j.chest.2020.09.057
13. Armando da R. Regression Polynomial Analysis of the COVID-19 Epidemics: An Alternative Infection Modeling. SSRN. 2020. doi:10.2139/ssrn.3682535
14. Arregoces HA, Rojano R, Restrepo G. Impact of lockdown on particulate matter concentrations in Colombia during the COVID-19 pandemic. Sci Total Environ. 2020; 142874. doi:http://dx.doi.org/10.1016/j.scitotenv.2020.142874
15. Arturo  Sr. C-V, Berenice Z-B, Jesus Carlos B-G, Guadalupe Mercedes Lucia G-A, Lourdes A-P, Eduardo F-O, et al. Serological Cytokine and chemokine profile in pregnant women with COVID19 in Mexico City. medRxiv. 2020. doi:10.1101/2020.07.14.20153585
16. Asanov I, Flores F, McKenzie D, Mensmann M, Schulte M. Remote-learning, time-use, and mental health of Ecuadorian high-school students during the COVID-19 quarantine. World Dev. 2021;138: 105225. doi:10.1016/j.worlddev.2020.105225
17. Astigueta-Perez J, Abad-Licham M, Chavez-Chirinos C, Beraun-Milla L, Lachos-Davila A, Diaz-Perez E, et al. Cancer disease progression and death during the COVID-19 pandemic: A multidisciplinary analysis for the Peruvian setting. Ecancermedicalscience. 2020;14: 1098. doi:http://dx.doi.org/10.3332/ECANCER.2020.1098
18. Atamari-Anahui N, Cruz-Nina ND, Condori-Huaraka M, Nunez-Paucar H, Rondon-Abuhadba EA, Ordonez-Linares ME, et al. [Characterization of coronavirus disease 2019 (COVID-19) in children and adolescents in Latin American and the Caribbean countries: A descriptive study]. Caracter la Enferm por coronavirus 2019 en ninos y Adolesc en paises Am Lat y El Caribe Estud Descr. 2020;20: e8025. doi:https://dx.doi.org/10.5867/medwave.2020.08.8025
19. Augusto  Sr. SF, Leonardo Sousa  Sr. S, Silvio  Sr. SLS. New S.I.R. model used in the projection of COVID 19 cases in Brazil. medRxiv. 2020. doi:10.1101/2020.04.26.20080218
20. Auler AC, Cassaro FAM, da Silva VO, Pires LF. Evidence that high temperatures and intermediate relative humidity might favor the spread of COVID-19 in tropical climate: A case study for the most affected Brazilian cities. Sci Total Environ. 2020;729: 139090. doi:https://dx.doi.org/10.1016/j.scitotenv.2020.139090
21. Autrán-Gómez AM, Tobia I, Molina RC, Covarrubias FR, Benzing F, Serena Maruccia F, et al. Exploring Urological Experience in the COVID-19 Outbreak: American Confederation of Urology (CAU) Survey. Int Braz J Urol. 2020;46. doi:10.1590/S1677-5538.IBJU.2020.S119
22. Avid R-G, Natalia IV-C. Variation of Aerosol Pollution in Peru during the Quarantine Due to COVID-19. Int J Adv Comput Sci Appl. 2020;11. doi:10.14569/IJACSA.2020.0110407
23. Avila-Ponce de León U, Pérez Á GC, Avila-Vales E. An SEIARD epidemic model for COVID-19 in Mexico: Mathematical analysis and state-level forecast. Chaos Solitons Fractals. 2020;140: 110165. doi:10.1016/j.chaos.2020.110165
24. Badellino H, Gobbo ME, Torres E, Aschieri ME. Early indicators and risk factors associated with mental health problems during COVID-19 quarantine: Is there a relationship with the number of confirmed cases and deaths? Int J Soc Psychiatry. 2020; 20764020966020. doi:http://dx.doi.org/10.1177/0020764020966020
25. Ballivian J, Alcaide ML, Cecchini D, Jones DL, Abbamonte JM, Cassetti I. Impact of COVID-19-Related Stress and Lockdown on Mental Health Among People Living With HIV in Argentina. J Acquir Immune Defic Syndr. 2020;85: 475–482. doi:http://dx.doi.org/10.1097/QAI.0000000000002493
26. Banerjee A, Ray S, Vorselaars B, Kitson J, Mamalakis M, Weeks S, et al. Use of Machine Learning and Artificial Intelligence to predict SARS-CoV-2 infection from Full Blood Counts in a population. Int Immunopharmacol. 2020;86: 106705. doi:10.1016/j.intimp.2020.106705
27. Baptista AS, Prado IM, Perazzo MF, Pinho TM, Paiva SM, Pordeus IA, et al. Can children’s oral hygiene and sleep routines be compromised during the COVID-19 pandemic? Int J Paediatr Dent. 2020. doi:http://dx.doi.org/10.1111/ipd.12732
28. Baqui P, Bica I, Marra V, Ercole A, van der Schaar M. Ethnic and regional variations in hospital mortality from COVID-19 in Brazil: a cross-sectional observational study. Lancet Glob Heal. 2020;8: e1018–e1026. doi:10.1016/S2214-109X(20)30285-0

Pedro B, Ioana B, Valerio M, Ari E, Mihaela van der S. Ethnic and Regional Variation in Hospital Mortality from COVID-19 in Brazil. SSRN. 2020. doi:10.2139/ssrn.3606429

1. Barbosa IR, Galvão MHR, Souza TA de, Gomes SM, Medeiros A de A, Lima KC de. ncidence of and mortality from COVID-19 in the older Brazilian population and its relationship with contextual indicators: an ecological study. Rev bras geriatr gerontol. 2020;23: 200171.
2. Barbosa V, Gomes J, Santana M, Albuquerque J, Souza R, Souza R, et al. Heg.IA: An intelligent system to support diagnosis of Covid-19 based on blood tests. ResearchSquare. 2020. doi:10.21203/rs.3.rs-33864/v1
3. Barbosa-Camacho F, García-Reyna B, Cervantes-Cardona G, Cervantes-Pérez E, Chavarria-Avila E, Pintor-Belmontes K, et al. Comparison of Fear of COVID-19 in Medical and Nonmedical Personnel in a Public Hospital in Mexico. ResearchSquare. 2020. doi:10.21203/rs.3.rs-37662/v1
4. Barone MTU, Villarroel D, de Luca PV, Harnik SB, Lima BL de S, Wieselberg RJP, et al. COVID-19 impact on people with diabetes in South and Central America (SACA region). Diabetes Res Clin Pract. 2020;166: 108301. doi:https://dx.doi.org/10.1016/j.diabres.2020.108301
5. Barra GB, Rita THS, Mesquita PG, Jacomo RH, Nery LFA. Analytical sensitivity and specificity of two RT-qPCR protocols for SARS-CoV-2 detection performed in an automated workflow. Genes (Basel). 2020;11: 1–14. doi:http://dx.doi.org/10.3390/genes11101183

Gustavo Barcelos B, Ticiane Henriques Santa R, Pedro Goes M, Rafael Henriques J, Lidia Freire Abdalla N. Analytical sensibility and specificity of two RT-qPCR protocols for SARS-CoV-2 detection performed in an automated workflow. medRxiv. 2020. doi:10.1101/2020.03.07.20032326

1. Barraza NR, Pena G, Moreno V. A non-homogeneous Markov early epidemic growth dynamics model. Application to the SARS-CoV-2 pandemic. Chaos Solitons Fractals. 2020;139: 110297. doi:10.1016/j.chaos.2020.110297
2. Barros AJD, Victora CG, Menezes AMB, Horta BL, Hartwig F, Victora G, et al. Social distancing patterns in nine municipalities of Rio Grande do Sul, Brazil: the Epicovid19/RS study. Rev Saude Publica. 2020;54: 75. doi:10.11606/s1518-8787.2020054002810
3. Barros MB de A, Lima MG, Malta DC, Szwarcwald CL, Azevedo RCS de, Romero D, et al. Report on sadness/depression, nervousness/anxiety and sleep problems in the Brazilian adult population during the COVID-19 pandemic. Relato tristeza/depressao, Nerv e Probl sono na Popul adulta Bras durante a pandemia COVID-19. 2020;29: e2020427. doi:https://dx.doi.org/10.1590/s1679-49742020000400018
4. Bastidas JAO, Pita APP, Vargas LNO, Montenegro AF. Importance of nonlinear regression models in the interpretation of data from COVID-19 in Colombia. Rev Habanera Ciencias Medicas. 2020;19: e_3309.
5. Bastos LS, Niquini RP, Lana RM, Villela DAM, Cruz OG, Coelho FC, et al. COVID-19 and hospitalizations for SARI in Brazil: a comparison up to the 12th epidemiological week of 2020. Cad Saude Publica. 2020;36: e00070120. doi:https://dx.doi.org/10.1590/0102-311X00070120
6. Bates BR, Moncayo AL, Costales JA, Herrera-Cespedes CA, Grijalva MJ. Knowledge, Attitudes, and Practices Towards COVID-19 Among Ecuadorians During the Outbreak: An Online Cross-Sectional Survey. J Community Health. 2020;45: 1158–1167. doi:http://dx.doi.org/10.1007/s10900-020-00916-7
7. Batista SR, Semeão de Souza AS, Nogueira J, de Andrade FB, Thumé E, Teixeira DS da C, et al. Protective behaviors for COVID-19 among Brazilian adults and elderly living with multimorbidity (ELSI-COVID-19 Initiative). 2020.
8. Batistela CM, Correa DPF, Bueno Á M, Piqueira JRC. SIRSi compartmental model for COVID-19 pandemic with immunity loss. Chaos Solitons Fractals. 2020; 110388. doi:10.1016/j.chaos.2020.110388
9. Batur LK, Hekim N. The role of DBP gene polymorphisms in the prevalence of new coronavirus disease 2019 infection and mortality rate. J Med Virol. 2020. doi:10.1002/jmv.26409
10. Beatrice N, Anthony B, Ankita R, Miguel B, Nol S, Adam RG, et al. The impact of high frequency rapid viral antigen screening on COVID-19 spread and outcomes: a validation and modeling study. medRxiv. 2020. doi:10.1101/2020.09.01.20184713
11. Beatriz Araujo O, Lea Campos de O, Franciane Mendes de O, Geovana Maria P, Regina Maia de S, Erika Regina M, et al. EVALUATION OF ELEVEN IMMUNOCHROMATOGRAPHIC ASSAYS FOR SARS-CoV-2 DETECTION: INVESTIGATING DENGUE CROSS-REACTION. medRxiv. 2020. doi:10.1101/2020.10.09.20210039
12. Beatriz Elena Fonseca M. La radio cubana ante la COVID-19. Un estudio de caso. Rev Esp Comun en Salud. 2020;11: 272–278. doi:10.20318/RECS.2020.5421
13. Beatriz Elena A, Yulieth Ximena T, Diana S, Magda C, Carlos G, Julio Cesar C, et al. Seroprevalence and seroconversion rates to SARS-CoV-2 in interns, residents, and medical doctors in a University Hospital in Bogota, Colombia. medRxiv. 2020. doi:10.1101/2020.09.15.20195313
14. Beatriz HT, Celso FHG, Maria CGPA, Maria CP, Edgar R, Marcia CN, et al. SARS-CoV-2 seroprevalence in the municipality of Sao Paulo, Brazil, ten weeks after the first reported case. medRxiv. 2020. doi:10.1101/2020.06.29.20142331
15. Belen P-V, Monica B-W, Juan Jose G, Sully M, Bernardo G, Patricio R-S, et al. COVID-19 Re-Infection by a Phylogenetically Distinct SARS-CoV-2 Variant, First Confirmed Event in South America. SSRN. 2020. doi:10.2139/ssrn.3686174
16. Bello-Chavolla OY, Antonio-Villa NE, Vargas-Vazquez A, Fermin-Martinez CA, Marquez-Salinas A, Bahena-Lopez JP. Profiling cases with non-respiratory symptoms and asymptomatic SARS-CoV-2 infections in Mexico City. Clin Infect Dis. 2020. doi:http://dx.doi.org/10.1093/cid/ciaa1288

Omar Yaxmehen B-C, Neftali Eduardo A-V, Arsenio V-V, Carlos AF-M, Alejandro M-S, Jessica Paola B-L. Profiling pre-symptomatic and asymptomatic cases with confirmed SARS-CoV-2 infection in Mexico City. medRxiv. 2020. doi:10.1101/2020.07.02.20145516

1. Bello-Chavolla OY, Bahena-Lopez JP, Antonio-Villa NE, Vargas-Vazquez A, Gonzalez-Diaz A, Marquez-Salinas A, et al. Predicting mortality due to SARS-CoV-2: A mechanistic score relating obesity and diabetes to COVID-19 outcomes in Mexico. J Clin Endocrinol Metab. 2020. doi:https://dx.doi.org/10.1210/clinem/dgaa346

Omar Yaxmehen B-C, Jessica Paola B-L, Neftali EA-V, Arsenio V-V, Armando G-D, Alejandro M-S, et al. Predicting mortality attributable to SARS-CoV-2: A mechanistic score relating obesity and diabetes to COVID-19 outcomes in Mexico. medRxiv. 2020. doi:10.1101/2020.04.20.20072223

1. Bello-Chavolla OY, Gonzalez-Diaz A, Antonio-Villa NE, Fermin-Martinez CA, Marquez-Salinas A, Vargas-Vazquez A, et al. Unequal impact of structural health determinants and comorbidity on COVID-19 severity and lethality in older Mexican adults: Considerations beyond chronological aging. J Gerontol A Biol Sci Med Sci. 2020. doi:https://dx.doi.org/10.1093/gerona/glaa163

Omar Yaxmehen B-C, Armando G-D, Neftali EA-V, Carlos AF-M, Alejandro M-S, Arsenio V-V, et al. Unequal impact of structural health determinants and comorbidity on COVID-19 severity and lethality in older Mexican adults: Looking beyond chronological aging. medRxiv. 2020. doi:10.1101/2020.05.12.20098699

1. Benites-Goñi H, Vargas-Carrillo E, Peña-Monge E, Taype-Rondan A, Arróspide-Mormontoy D, Castillo-Córdova M, et al. Clinical characteristics, management and mortality of patients hospitalized with COVID-19 in a reference hospital in Lima, Peru. 2020.
2. Benitez MA, Velasco C, Sequeira AR, Henriquez J, Menezes FM, Paolucci F. Responses to COVID-19 in five Latin American countries. Heal Policy Technol. 2020. doi:http://dx.doi.org/10.1016/j.hlpt.2020.08.014
3. Benjamin V-A, Irma H-U, Luis E-A, Raquel M-A, Javier G-G, Diego O-Z, et al. COVID-19 severe pneumonia in Mexico City - First experience in a Mexican hospital. medRxiv. 2020. doi:10.1101/2020.04.26.20080796
4. Bennett M. All Things Equal? Heterogeneity in Policy Effectiveness against COVID-19 Spread in Chile. World Dev. 2021;137: 105208. doi:10.1016/j.worlddev.2020.105208
5. Bernabe-Ramirez C, Velazquez AI, Olazagasti C, Bergerot CD, Bergerot PG, Soto-Perez-de-Celis E, et al. The HOLA COVID-19 Study: An International Effort to Determine How COVID-19 Has Impacted Oncology Practices in Latin America. Cancer Cell. 2020. doi:http://dx.doi.org/10.1016/j.ccell.2020.10.013
6. Beskow AF, Martinez-Duartez PR, Behrens Estrada EJ, Fiolo FE, Ramos AC. CoViD-19 Pandemic and Bariatric Surgery in Argentina. Obes Surg. 2020. doi:http://dx.doi.org/10.1007/s11695-020-05004-2
7. Bezerra ACV, Silva CEM da, Soares FRG, Silva JAM da. Factors associated with people’s behavior in social isolation during the COVID-19 pandemic. Cien Saude Colet. 2020;25: 2411–2421. doi:https://dx.doi.org/10.1590/1413-81232020256.1.10792020
8. Bigoni A, Fink G. Adding to the debate on the influence of temperature on corona virus disease (COVID-19): the case of Brazil. Public Health. 2020;187: 74–76. doi:http://dx.doi.org/10.1016/j.puhe.2020.07.040
9. Bitar S, Steinmetz WA. Scenarios for the Spread of COVID-19 in Manaus, Northern Brazil. An Acad Bras Cienc. 2020;92: e20200615. doi:https://dx.doi.org/10.1590/0001-3765202020200615
10. Bittar C, Machado RRG, Comelis MT, Bueno LM, Beguelini MR, Morielle-Versute E, et al. Alphacoronavirus Detection in Lungs, Liver, and Intestines of Bats from Brazil. Microb Ecol. 2020;79: 203–212. doi:https://dx.doi.org/10.1007/s00248-019-01391-x
11. Bolano-Ortiz TR, Camargo-Caicedo Y, Puliafito SE, Ruggeri MF, Bolano-Diaz S, Pascual-Flores R, et al. Spread of SARS-CoV-2 through Latin America and the Caribbean region: A look from its economic conditions, climate and air pollution indicators. Environ Res. 2020;191: 109938. doi:http://dx.doi.org/10.1016/j.envres.2020.109938
12. Bolanos-Almeida CE, Espitia Segura OM. Clinical and Epidemiologic Analysis of COVID-19 Children Cases in Colombia PEDIACOVID. Pediatr Infect Dis J. 2020. doi:http://dx.doi.org/10.1097/INF.0000000000002952

Carlos Ernesto B-A, Oscar Mauricio E-S. Clinical and Epidemiological Analysis of COVID-19 Children Cases in Colombia PEDIACOVID. SSRN. 2020. doi:10.2139/ssrn.3678580

1. Borba MGS, Val FFA, Sampaio VS, Alexandre MAA, Melo GC, Brito M, et al. Effect of High vs Low Doses of Chloroquine Diphosphate as Adjunctive Therapy for Patients Hospitalized With Severe Acute Respiratory Syndrome Coronavirus 2 (SARS-CoV-2) Infection: A Randomized Clinical Trial. JAMA Netw open. 2020;3: e208857. doi:https://dx.doi.org/10.1001/jamanetworkopen.2020.8857

Mayla B, Fernando de Almeida V, Vanderson Sousa S, Marcia Araujo A, Gisely Cardoso M, Marcelo B, et al. Chloroquine diphosphate in two different dosages as adjunctive therapy of hospitalized patients with severe respiratory syndrome in the context of coronavirus (SARS-CoV-2) infection: Preliminary safety results of a randomized, double-blinded, phase IIb cl. medRxiv. 2020. doi:10.1101/2020.04.07.20056424

1. Borba PL de O, Bassi BG de C, Pereira BP, Vasters GP, Correia RL, Barreiro RG. “Practical and reflective” challenges for occupational therapy’s undergraduation courses in pandemic times. 2020.
2. Borges GM, Crespo CD. Demographic and socioeconomic characteristics of Brazilian adults and COVID-19: a risk group analysis based on the Brazilian National Health Survey, 2013. Cad Saude Publica. 2020;36: e00141020. doi:http://dx.doi.org/10.1590/0102-311X00141020
3. Borges LL, Guimaraes CC V, Aguiar BGC, Felipe LAF. Military Nursing in “Operation Return to Brazil”: aeromedical evacuation in the coronavirus pandemic. Rev Bras Enferm. 2020;73 2: e20200297. doi:http://dx.doi.org/10.1590/0034-7167-2020-0297
4. Borja-Villanueva CA, Gómez-Carrión CE, Alvarado-Muñoz ER, Bernuy-Torres LA. Knowledge about coronavirus disease (COvId-19) in dentists of Lima and Callao. Rev cient odontol. 2020;8: e019–e019.
5. Borracci RA, Giglio ND. Forecasting the effect of social distancing on COVID-19 autumn-winter outbreak in the metropolitan area of Buenos Aires. Estim del Ef del distanciamiento Soc sobre la epidemia COVID-19 otono-invierno en el area Metrop Buenos Aires. 2020;80 Suppl 3: 7–15.
6. Bottan N, Hoffmann B, Vera-Cossio D. The unequal impact of the coronavirus pandemic: Evidence from seventeen developing countries. PLoS One. 2020;15: e0239797. doi:http://dx.doi.org/10.1371/journal.pone.0239797
7. Bozovich GE, Alves De Lima A, Fosco M, Burgos LM, Martínez R, Dupuy De Lôme R, et al. [Collateral damage of COVID-19 pandemic in private healthcare centres of Argentina]. Medicina (B Aires). 2020;80 Suppl 3: 37–41.
8. Braga JU, Ramos  Jr. AN, Ferreira AF, Lacerda VM, Freire RMC, Bertoncini BV. Propensity for COVID-19 severe epidemic among the populations of the neighborhoods of Fortaleza, Brazil, in 2020. BMC Public Health. 2020;20: 1486. doi:https://dx.doi.org/10.1186/s12889-020-09558-9
9. Brandao Neto D, Fornazieri MA, Dib C, Di Francesco RC, Doty RL, Voegels RL, et al. Chemosensory Dysfunction in COVID-19: Prevalences, Recovery Rates, and Clinical Associations on a Large Brazilian Sample. Otolaryngol - Head Neck Surg (United States). 2020. doi:http://dx.doi.org/10.1177/0194599820954825
10. Brinkley R, Elvis WD, Julianna S, Edith Z, Ynes M, Claudia M, et al. The impact of the COVID-19 pandemic on rabies reemergence in Latin America: the case of Arequipa, Peru. medRxiv. 2020. doi:10.1101/2020.08.06.20169581
11. Brito LGO, Romao GS, Fernandes CE, Silva-Filho AL. Impact of COVID-19 on Brazilian medical residencies in obstetrics and gynecology. Int J Gynaecol Obstet. 2020. doi:https://dx.doi.org/10.1002/ijgo.13283
12. Bruna Caruso M, Fabiana Infante S, Gabriel Perri E, Heloisa CSA, Marcia Thereza C, Milla Cordeiro A, et al. Influence of nutritional status on eating habits and food choice determinants among Brazilian women during the COVID-19 pandemic. medRxiv. 2020. doi:10.1101/2020.11.03.20225136
13. Bruno Campello de S, Fernando Menezes Campello de S. Does Social Isolation Really Curb COVID-19 Deaths? Direct Evidence from Brazil that it Might do the Exact Opposite. SSRN. 2020. doi:10.2139/ssrn.3706464
14. Bruno Campello de S, Fernando Menezes Campello de S. Physical Distancing and Future COVID-19 Deaths in Brazil: Evidence of a Paradoxical Effect. SSRN. 2020. doi:10.2139/ssrn.3711686
15. Bryan Adrian G-P, Arturo T-R, Samanta Mayanini P-G, Cristina D-R, Omar S-N, Marisol Manriquez R, et al. Anxiety, depression, attitudes, and internet addiction during the initial phase of the 2019 coronavirus disease (COVID-19) epidemic: A cross-sectional study in Mexico. medRxiv. 2020. doi:10.1101/2020.05.10.20095844
16. Bryan V, Jose LA, Torres-Roman JS, Julio AP, Janina B-P, Carlo La V. The effect of public health policies in the transmission of COVID-19 for South American countries. medRxiv. 2020. doi:10.1101/2020.08.09.20149286
17. Buckman SR, Glick R, Lansing KJ, Petrosky-Nadeau N, Seitelman LM. Replicating and projecting the path of COVID-19 with a model-implied reproduction number. Infect Dis Model. 2020;5: 635–651. doi:http://dx.doi.org/10.1016/j.idm.2020.08.007
18. Buonafine C, Paiatto B, Leal F, Matos S, Moraes C, Guerra G, et al. High prevalence of SARS-CoV-2 Infection Among Symptomatic Healthcare Workers in a large university tertiary hospital in São Paulo, Brazil. ResearchSquare. 2020. doi:10.21203/rs.3.rs-42898/v1
19. Burgos LM, Diez M, Villalba L, Miranda RM, Belardi J. [Impact of the COVID-19 pandemic on heart failure hospitalizations]. Medicina (B Aires). 2020;80: 315–316.
20. Bustos-Cordova E, Castillo-García D, Cerón-Rodriguez M, Soler-Quiñones N. Clinical Spectrum of COVID-19 in a Pediatric Mexican Population. ResearchSquare. 2020. doi:10.21203/rs.3.rs-81586/v1
21. Caballero-Dominguez CC, Jimenez-Villamizar MP, Campo-Arias A. Suicide risk during the lockdown due to coronavirus disease (COVID-19) in Colombia. Death Stud. 2020; 1–6. doi:https://dx.doi.org/10.1080/07481187.2020.1784312
22. Caicedo-Ochoa Y, Rebellon-Sanchez DE, Penaloza-Rallon M, Cortes-Motta HF, Mendez-Fandino YR. Effective Reproductive Number estimation for initial stage of COVID-19 pandemic in Latin American Countries. Int J Infect Dis. 2020;95: 316–318. doi:https://dx.doi.org/10.1016/j.ijid.2020.04.069
23. Caio Willer Brito G, Dário Luigi Ferraz G, Adir Bernardes Pinto N, Gleiziane Sousa L, Kelvin Hamim José Feitosa R, Eros Silva C. INCIDENCE OF COVID-19 IN THE STATES OF THE NORTHERN REGION OF BRAZIL. 2020;6. doi:10.26694/REPIS.V6I0.10489
24. Calandri IL, Hawkes MA, Marrodan M, Ameriso SF, Correale J, Allegri RF. The impact of an early strict nationwide lockdown on the pattern of consultation for neurological diseases. J Neurol Sci. 2020;418: 117084. doi:http://dx.doi.org/10.1016/j.jns.2020.117084
25. Calderon JM, Ma. Flores DRF, Coria LP, Briones Garduno JC, Figueroa JM, Vargas Contreras MJ, et al. Nitazoxanide against COVID-19 in three explorative scenarios. J Infect Dev Ctries. 2020;14: 982–986. doi:http://dx.doi.org/10.3855/JIDC.13274
26. Calderon JM, Zeron HM, Padmanabhan S. Treatment with Hydroxychloroquine vs Hydroxychloroquine + Nitazoxanide in COVID-19 patients with risk factors for poor prognosis: A structured summary of a study protocol for a randomised controlled trial. Trials. 2020;21: 504. doi:https://dx.doi.org/10.1186/s13063-020-04448-2
27. Calegaro V, Negretto B, Weber L, Kerber N, Zoratto G, Rodrigues L, et al. Monitoring the evolution of posttraumatic symptomatology, depression and anxiety during the COVID-19 pandemic in Brazilians (COVIDPsiq). ResearchSquare. 2020. doi:10.21203/rs.3.pex-945/v1
28. Calvimontes J, Massaro L, Araujo CHX, Moraes RR, Mello J, Ferreira LC, et al. Small-scale gold mining and the COVID-19 pandemic: Conflict and cooperation in the Brazilian Amazon. Extr Ind Soc. 2020. doi:10.1016/j.exis.2020.08.013
29. Camelo-Filho AE, Silva AMS, Estephan EP, Zambon AA, Mendonca RH, Souza PVS, et al. Myasthenia Gravis and COVID-19: Clinical Characteristics and Outcomes. Front Neurol. 2020;11: 1053. doi:http://dx.doi.org/10.3389/fneur.2020.01053
30. Campo KN, Rodrigues ICP, Lopes ESN, Gabriel LP. Early public research funding response to COVID-19 pandemic in Brazil. Rev Soc Bras Med Trop. 2020;53: e20200522. doi:https://dx.doi.org/10.1590/0037-8682-0522-2020
31. Campos JADB, Martins BG, Campos LA, Maroco J, Saadiq RA, Ruano R. Early psychological impact of the COVID-19 pandemic in Brazil: A national survey. J Clin Med. 2020;9: 1–14. doi:http://dx.doi.org/10.3390/jcm9092976
32. Canabarro A, Tenório E, Martins R, Martins L, Brito S, Chaves R. Data-driven study of the COVID-19 pandemic via age-structured modelling and prediction of the health system failure in Brazil amid diverse intervention strategies. PLoS One. 2020;15: e0236310. doi:10.1371/journal.pone.0236310

Askery C, Elayne T, Renato M, Lais M, Samurai B, Rafael C. Data-Driven Study of the the COVID-19 Pandemic via Age-Structured Modelling and Prediction of the Health System Failure in Brazil amid Diverse Intervention Strategies. medRxiv. 2020. doi:10.1101/2020.04.03.20052498

1. Canals M, Cuadrado C, Canals A, Yohannessen K, Lefio LA, Bertoglia MP, et al. Epidemic trends, public health response and health system capacity: the Chilean experience in four months of the COVID-19 pandemic. Rev Panam Salud Publica. 2020;44: e99. doi:10.26633/RPSP.2020.99
2. Candeiro GT de M, Gavini G, Vivan RR, Carvalho BMDF, Duarte MAH, FeijAo CP, et al. Knowledge about Coronavirus disease 19 (COVID-19) and its professional repercussions among Brazilian endodontists. Braz Oral Res. 2020;34: e117. doi:https://dx.doi.org/10.1590/1807-3107bor-2020.vol34.0117
3. Candelaria Brito JC, Díaz Cruz SA, Acosta Pérez DM, Junco Sena B, Rodríguez Méndez A. First Cuban community quarantined by COVID-19. Rev cienc med Pinar Rio. 2020;24: e4485–e4485.
4. Candelaria Brito JC, Díaz Cruz SA, Acosta Pérez DM, Labrador Mazón O, Rodríguez Méndez A. Intervention strategy aimed at the prevention and control of COVID-19 in Consolación del Sur. Rev cienc med Pinar Rio. 2020;24: e4495–e4495.
5. Candido DS, Claro IM, de Jesus JG, Souza WM, Moreira FRR, Dellicour S, et al. Evolution and epidemic spread of SARS-CoV-2 in Brazil. Science. 2020. doi:10.1126/science.abd2161
6. Candido DDS, Watts A, Abade L, Kraemer MUG, Pybus OG, Croda J, et al. Routes for COVID-19 importation in Brazil. J Travel Med. 2020;27. doi:https://dx.doi.org/10.1093/jtm/taaa042

Routes for COVID-19 importation in Brazil ;Journal of Travel Medicine ;Oxford Academic. 2020.

1. Canet-Juric L, Andrés ML, Del Valle M, López-Morales H, Poó F, Galli JI, et al. A Longitudinal Study on the Emotional Impact Cause by the COVID-19 Pandemic Quarantine on General Population. Front Psychol. 2020;11: 565688. doi:10.3389/fpsyg.2020.565688
2. Canizares Fuentes R, Aroca R, Blasco Carlos M. Evaluation of COVID19 surveillance strategy in Ecuador. Disaster Med Public Health Prep. 2020; 1–9. doi:http://dx.doi.org/10.1017/dmp.2020.326
3. Cano-Perez E, Torres-Pacheco J, Fragozo-Ramos MC, Garcia-Diaz G, Montalvo-Varela E, Pozo-Palacios JC. Negative Correlation between Altitude and COVID-19 Pandemic in Colombia: A Preliminary Report. Am J Trop Med Hyg. 2020. doi:http://dx.doi.org/10.4269/ajtmh.20-1027
4. Cárcamo LM, Tejeda MJ, Castro-Clavijo J, Montoya L, Barrezueta LJ, Cardona SV, et al. Características clínicas y sociodemográficas de y sociodemográficas de pacientes fallecidos por pacientes fallecidos por COVID-19 en Colombia COVID-19 en Colombia. Reper med cir. 2020;29: 45–51.
5. Cárcamo LM, Tejeda MJ, Castro-Clavijo J, Montoya L, Barrezueta LJ, Cardona SV, et al. Clinical and sociodemographic characteristics of patients who died from COVID-19 in Colombia patients who died from COVID-19 in Colombia. Reper med cir. 2020;29: 45–51.
6. Cardona-Ospina JA, Arteaga-Livias K, Villamil-Gomez WE, Perez-Diaz CE, Katterine Bonilla-Aldana D, Mondragon-Cardona A, et al. Dengue and COVID-19, overlapping epidemics? An Analysis from Colombia. J Med Virol. 2020. doi:https://dx.doi.org/10.1002/jmv.26194
7. Carla Lourenço Tavares de A, Claudia Cristina de Aguiar P, Mônica M, Sheyla Maria Lemos L, Margareth Crisóstomo P. COVID-19 hospitalizations in Brazil’s Unified Health System (SUS). medRxiv. 2020. doi:10.1101/2020.09.03.20187617
8. Carlos  Jr. AP, Lewis B, Victor Bertollo P, Darlan  Jr. A da SC, Fabio G, Amy D, et al. Serial Interval Distribution of SARS-CoV-2 Infection in Brazil. medRxiv. 2020. doi:10.1101/2020.06.09.20127043
9. Carlos  Sr. AR, Airandes  Sr. SP, Carlito  Sr. LS, Edval  Jr. GS, Livia  Sr. AC, Paulo  Sr. CN, et al. Covid-19 epidemic curve in Brazil: A sum of multiple epidemics, whose income inequality and population density in the states are correlated with growth rate and daily acceleration. medRxiv. 2020. doi:10.1101/2020.09.09.20191353
10. Carlos Augusto Cardoso  Sr. P, Estefano Aparecido  Sr. V, Jose Andre  Sr. L, Jefferson Oliveira do  Sr. N. Characteristics and evolution of COVID-19 cases in Brazil: mathematical modeling and simulation. medRxiv. 2020. doi:10.1101/2020.10.14.20212829
11. Carlos Eduardo R, Marcella Cini O, Tatiana de Araujo E, Suzana Rosa A, Marcele Gonçalves da S, Eny Regina da Silva Q, et al. Spatial Analysis of COVID-19 Incidence and the Sociodemographic Context in Brazil. SSRN. 2020. doi:10.2139/ssrn.3709861
12. Carlos Enrique Bustamante O, Jordy Jose Cevallos C, Cesar M, Jeff S, Edwin M, Anuj M. Modeling and Preparedness: The Transmission Dynamics of COVID-19 Outbreak in Provinces of Ecuador. medRxiv. 2020. doi:10.1101/2020.07.09.20150078
13. Carlos Jesús A-A, Julissa C-U, Luis H, Frank Z-C, Cender UQ-J. Infodemiological study of COVID-19 in Latin America and The Caribbean. medRxiv. 2020. doi:10.1101/2020.08.11.20173054
14. Carlos Magno Castelo Branco F, Raul Borges G, Rafael de Castro C, Claudia Pio F, Gabriel Berg de A, Edmur P. Elementary spatial structures and dispersion of COVID-19: health geography directing responses to public health emergency in Sao Paulo State, Brazil. medRxiv. 2020. doi:10.1101/2020.04.26.20080895
15. Carlos von Krakauer  Sr. H, Marcella Lima B, Rafaella Dourado L. Distress among Brazilian university students due to the Covid-19 pandemic: survey results and reflections. medRxiv. 2020. doi:10.1101/2020.06.19.20135251
16. Carlos F-M, Diego Alejandro A-D, Katherine L-D, Magdalena W, Patricia E, Jose AU-C, et al. Substitutions in Spike and Nucleocapsid proteins of SARS-CoV-2 circulating in Colombia. medRxiv. 2020. doi:10.1101/2020.06.02.20120782
17. Carlos IM. Inhomogeneous mixing and asynchronic transmission between local outbreaks account for the spread of COVID-19 epidemics. medRxiv. 2020. doi:10.1101/2020.08.04.20168443
18. Carlos MH-S. Statistics associated with the lethality of COVID-19 by age group and gender in Mexico. medRxiv. 2020. doi:10.1101/2020.06.28.20142117
19. Carlos MH-S, Efren M-Z. Using COVID-19 deaths as a surrogate to measure the progression of the pandemics. medRxiv. 2020. doi:10.1101/2020.09.27.20202564
20. Carlos MH-S, Paolo V, Efren M-Z. On the estimation of the total number of SARS-CoV-2 infections. medRxiv. 2020. doi:10.1101/2020.04.23.20077446
21. Carlos M-R, Hilario M-L. Data Mining for the Study of the Epidemic (SARS- CoV-2) COVID-19: Algorithm for the Identification of Patients (SARS-CoV-2) COVID 19 in Mexico. SSRN. 2020. doi:10.2139/ssrn.3619549
22. Carlos M-R, Hilario M-L. Impact of (SARS-CoV-2) COVID-19 on the Five Main Indigenous Language-Speaking Areas in Veracruz Mexico: The Case of the Totonacapan Area. SSRN. 2020. doi:10.2139/ssrn.3672636
23. Carlos-Maria A, Sergio S, Gabriela N, Raul R-V, Carolina G-S. Job insecurity, financial threat and mental health in the COVID-19 context: The buffer role of perceived social support. medRxiv. 2020. doi:10.1101/2020.07.31.20165910
24. Carmen Cecilia C-D, Jeimmy De L-S, Adalberto C-A. Social capital and psychological distress during Colombian coronavirus disease lockdown. medRxiv. 2020. doi:10.1101/2020.09.04.20187914
25. Carmo RF, Nunes BEBR, Machado MF, Armstrong AC, Souza CDF. Expansion of COVID-19 within Brazil: the importance of highways. J Travel Med. 2020. doi:https://dx.doi.org/10.1093/jtm/taaa106
26. Carolina P, Marcos Antonio Ferreira J, Andreia Insabralde de Queiroz C, Luciana Scarlazzari C, Mercy da Costa S, Felipe Machado M, et al. Temporal analysis of the clinical evolution of confirmed cases of COVID-19 in the state of Mato Grosso do Sul - Brazil. medRxiv. 2020. doi:10.1101/2020.09.21.20198812
27. Carrascosa MMC, Campos T de, Sampaio JE, Souza RRF, Ribeiro VL, Maia MLN, et al. Medical Interns and COVID-19: results of national research. Rev Assoc Med Bras. 2020;66: 812–817. doi:https://dx.doi.org/10.1590/1806-9282.66.6.812
28. Carriel J, Muñoz-Jaramillo R, Bolaños-Ladinez O, Heredia-Villacreses F, Menéndez-Sanchón J, Martin-Delgado J, et al. [CURB-65 as a predictor of 30-day mortality in patients hospitalized with COVID-19 in Ecuador: COVID-EC StudyAbstract]. Rev Clin Esp. 2020. doi:10.1016/j.rce.2020.10.001
29. Carrillo-Vega MF, Salinas-Escudero G, Garcia-Pena C, Gutierrez-Robledo LM, Parra-Rodriguez L. Early estimation of the risk factors for hospitalization and mortality by COVID-19 in Mexico. PLoS One. 2020;15: e0238905. doi:https://dx.doi.org/10.1371/journal.pone.0238905

Maria Fernanda C-V, Guillermo S-E, Carmen G-P, Luis Miguel G-R, Lorena P-R. Early estimation of the risk factors for hospitalisation and mortality by COVID-19 in Mexico. medRxiv. 2020. doi:10.1101/2020.05.11.20098145

1. Carvalho Malta D, Saar Gomes C, Landmann Szwarcwald C, Berti de Azevedo Barros M, Gomes da Silva A, Junio Sady Prates E, et al. Social distancing, feeling of sadness and lifestyles of the Brazilian population during the COVID-19 pandemic. 2020.
2. Carvalho EC, Souza PHD de O, Varella TCMYML, Souza NVD de O, Farias SNP de, Soares SSS. COVID-19 pandemic and the judicialization of health care: an explanatory case study. Rev Lat Am Enfermagem. 2020;28: e3354. doi:https://dx.doi.org/10.1590/1518-8345.4584.3354
3. Carvalho HEF, Schneider G, Sousa AR, Camargo ELS, Nunes R V, Possani MA, et al. Suspected COVID-19 flu-like syndrome in men who have sex with men and have been involved in casual sex. Rev Bras Enferm. 2020;73: e20200913. doi:http://dx.doi.org/10.1590/0034-7167-2020-0913
4. Carvalho L de F, Pianowski G, Goncalves AP. Personality differences and COVID-19: are extroversion and conscientiousness personality traits associated with engagement with containment measures? Trends psychiatry Psychother. 2020. doi:https://dx.doi.org/10.1590/2237-6089-2020-0029
5. Cassiani-Miranda CA, Campo-Arias A, Tirado-Otálvaro AF, Botero-Tobón LA, Upegui-Arango LD, Rodríguez-Verdugo MS, et al. Stigmatisation associated with COVID-19 in the general Colombian population. Int J Soc Psychiatry. 2020; 20764020972445. doi:10.1177/0020764020972445
6. Castillo AE, Parra B, Tapia P, Acevedo A, Lagos J, Andrade W, et al. Phylogenetic analysis of the first four SARS-CoV-2 cases in Chile. J Med Virol. 2020. doi:https://dx.doi.org/10.1002/jmv.25797
7. Castro R, Luz PM, Wakimoto MD, Veloso VG, Grinsztejn B, Perazzo H. COVID-19: a meta-analysis of diagnostic test accuracy of commercial assays registered in Brazil. Braz J Infect Dis. 2020;24: 180–187. doi:https://dx.doi.org/10.1016/j.bjid.2020.04.003
8. Castro-Paris R, Munoz-Velandia O, Garcia-Pena A, Fernández-Ávila D. Assessment of the Academic Satisfaction of Graduate Students During the SARS-CoV-2 Pandemic, In An Internal Medicine Department. ResearchSquare. 2020. doi:10.21203/rs.3.rs-69949/v1
9. Castro-Rodriguez Y, Valenzuela-Torres O. Implications of COVID-19 pandemic for dental care: A perspective for clinical dentists. Rev Habanera Ciencias Medicas. 2020;19: A58.
10. Catarina Vezetiv  Jr. M, Aluizio M, Vitoria Ferreira C, Maria do Carmo Pinho F, Elke S, Luciana Yuki T. High prevalence of food insecurity, the adverse impact of COVID-19 in Brazilian favela. medRxiv. 2020. doi:10.1101/2020.07.31.20166157
11. Cavalcante FP, Novita GG, Millen EC, Zerwes FP, de Oliveira VM, Sousa ALL, et al. Management of early breast cancer during the COVID-19 pandemic in Brazil. Breast Cancer Res Treat. 2020. doi:10.1007/s10549-020-05877-y
12. Cavalcante JR, Cardoso-Dos-Santos AC, Bremm JM, Lobo AP, Macário EM, Oliveira WK, et al. COVID-19 in Brazil: evolution of the epidemic up until epidemiological week 20 of 2020. Epidemiol e Serv saude  Rev do Sist Unico Saude do Bras. 2020;29: e2020376. doi:10.5123/s1679-49742020000400010
13. Cavalcante JR, Abreu A de JL de. COVID-19 in the city of Rio de Janeiro: spatial analysis of first confirmed cases and deaths. Epidemiol e Serv saude  Rev do Sist Unico Saude do Bras. 2020;29: e2020204. doi:https://dx.doi.org/10.5123/S1679-49742020000300007

Cavalcante JR, Abreu A de JL de. COVID-19 in Rio de Janeiro municipality: spatial distribution of the first deaths and cases confirmed. 2020.

1. Cavalcanti AB, Zampieri FG, Rosa RG, Azevedo LCP, Veiga VC, Avezum A, et al. Hydroxychloroquine with or without Azithromycin in Mild-to-Moderate Covid-19. N Engl J Med. 2020. doi:10.1056/NEJMoa2019014

Alexandre BC, Fernando GZ, Luciani CPA, Regis GR, Alvaro A, Viviane CV, et al. Hydroxychloroquine alone or in combination with azithromycin to prevent major clinical events in hospitalised patients with coronavirus infection (COVID-19): rationale and design of a randomised, controlled clinical trial. medRxiv. 2020. doi:10.1101/2020.05.19.20106997

1. Cavalcanti YW, da Silva RO, Ferreira LF, de Lucena EHG, de Souza AMLB, Cavalcante DFB, et al. Economic impact of new biosafety recommendations for dental clinical practice during covid-19 pandemic. Pesqui Bras Odontopediatria Clin Integr. 2020;20: 1–9. doi:10.1590/pboci.2020.143
2. Cavazos-Arroyo J, Pérez de Celis-Herrero C. Severity, susceptibility and social norms perceived as antecedents of the intention to be vaccinated against COVID-19. Rev salud pública. 2020;22: e486877–e486877.
3. Caycho-Rodriguez T, Carbajal-Leon C, Vilca LW, Heredia-Mongrut J, Gallegos M. COVID-19 and mental health in peruvian police officers: Preliminary results. Acta Medica Peru. 2020;37: 396–398. doi:http://dx.doi.org/10.35663/amp.2020.373.1503
4. Caycho-Rodriguez T, Vilca LW, Cervigni M, Gallegos M, Martino P, Portillo N, et al. Fear of COVID-19 scale: Validity, reliability and factorial invariance in Argentina’s general population. Death Stud. 2020; 1–10. doi:http://dx.doi.org/10.1080/07481187.2020.1836071
5. Cazorla M, Herrera E, Palomeque E, Saud N. What the COVID-19 lockdown revealed about photochemistry and ozone production in Quito, Ecuador. Atmos Pollut Res. 2020. doi:10.1016/j.apr.2020.08.028
6. Cecilia S, Florencia D-V, Marianoel P-G, Ignacio F, Pilar M, Gonzalo M, et al. Multiple introductions, regional spread and local differentiation during the first week of COVID-19 epidemic in Montevideo, Uruguay. bioRxiv. 2020. doi:10.1101/2020.05.09.086223
7. cesar a barbero. A statistical forecast of LOW mortality and morbidity due to COVID-19, in ARGENTINA and other Southern Hemisphere countries. medRxiv. 2020. doi:10.1101/2020.04.20.20072488
8. Cesar Augusto Trinta W, Ingridi Teixeira M, Julia Medeiros G, Wagner Silva de S. The Use of Psychoactive Substances in the Context of the Covid-19 Pandemic in Brazil. medRxiv. 2020. doi:10.1101/2020.09.25.20194431
9. Cesar M, Gerardo C, Amna T, Eduardo AU, Kenji M. Risk of death by age and gender from CoVID-19 in Peru, March-May, 2020. medRxiv. 2020. doi:10.1101/2020.06.14.20123315
10. Chacón-Torres J, Reinoso C, Navas-Leon D, Briceño S, González G. Optimized and scalable synthesis of magnetic nanoparticles for RNA extraction in response to developing countries’ needs in the detection and control of SARS-CoV-2. ResearchSquare. 2020. doi:10.21203/rs.3.rs-34552/v1
11. Chafloque-Vasquez RA, Pampa-Espinoza L, Celis Salinas JC. Seroprevalence of COVID-19 in workers in a hospital in the Peruvian amazon. Acta Medica Peru. 2020;37: 390–392. doi:http://dx.doi.org/10.35663/amp.2020.373.1050
12. Charles R, Marianne S, Luana Marotta Reis de V, Mariana Raquel da Cruz V, Keila Cristina M, Adriano B, et al. Higher education challenges and possibilities: a Brazilian experience in times of COVID-19. Res Soc Dev. 2020;9. doi:10.33448/RSD-V9I8.5970
13. Chate RC, Fonseca EKUN, Passos RBD, Teles GB da S, Shoji H, Szarf G. Presentation of pulmonary infection on CT in COVID-19: initial experience in Brazil. J Bras Pneumol. 2020;46: e20200121. doi:https://dx.doi.org/10.36416/1806-3756/e20200121
14. Chavarri-Guerra Y, Ramos-Lopez WA, Covarrubias-Gomez A, Sanchez-Roman S, Quiroz-Friedman P, Alcocer-Castillejos N, et al. Providing Supportive and Palliative Care Using Telemedicine for Patients with Advanced Cancer During the COVID-19 Pandemic in Mexico. Oncologist. 2020. doi:http://dx.doi.org/10.1002/onco.13568
15. Chavin DG, Dale V, Michelle T, Davlin T. The Epidemiology Characteristics of Positive COVID-19 patients in a Caribbean Territory. medRxiv. 2020. doi:10.1101/2020.08.06.20148288
16. Chen X, Zhang SX, Jahanshahi AA, Alvarez-Risco A, Dai H, Li J, et al. Belief in a COVID-19 Conspiracy Theory as a Predictor of Mental Health and Well-Being of Health Care Workers in Ecuador: Cross-Sectional Survey Study. JMIR public Heal Surveill. 2020;6: e20737. doi:https://dx.doi.org/10.2196/20737
17. Chen YT, Yen YF, Yu SH, Su EC. An Examination on the Transmission of COVID-19 and the Effect of Response Strategies: A Comparative Analysis. Int J Environ Res Public Health. 2020;17: 1–14. doi:10.3390/ijerph17165687
18. Chiesa-Estomba CM, Lechien JR, Portillo-Mazal P, Martinez F, Cuauro-Sanchez J, Calvo-Henriquez C, et al. Olfactory and gustatory dysfunctions in COVID-19. First reports of Latin-American ethnic patients. Am J Otolaryngol. 2020;41: 102605. doi:https://dx.doi.org/10.1016/j.amjoto.2020.102605
19. Chioro A, Calife K, Santos Barros CR dos, Martins LC, Calvo M, Estanislau E, et al. COVID-19 at a metropolitan region: public polices and social vulnerability within an iniquity context. 2020.
20. Chisini LA, Sartori LRM, Costa FDS, Salvi LC, Demarco FF. COVID-19 pandemic impact on prosthetic treatments in the Brazilian Public Health System. Oral Dis. 2020. doi:http://dx.doi.org/10.1111/odi.13668
21. Christian Omar R-P, Erika Areli R-G, Cristina Elizabeth M-M, Monica Patricia B-R, Rafael García R, Carolina B-D, et al. Laboratory Parameters and Complication Risk Factors in Mexican COVID-19 Patients: A Cross-Sectional Study. SSRN. 2020. doi:10.2139/ssrn.3634850
22. Christian A-R, Favio C-R, Liliana P-M, Fernanda A-R, Danuzia AM, Natalia Zubieta D, et al. Decreased incidence, virus transmission capacity, and severity of COVID-19 at altitude on the American continent. medRxiv. 2020. doi:10.1101/2020.07.22.20160168
23. Christian C-C, Medalit L-V, Julio M-A, Blanca S-M, Diana P-A, Ramiro P-M, et al. Clinical-epidemiological and treatment characteristics of children with COVID-19 in a tertiary referral center in Peru. medRxiv. 2020. doi:10.1101/2020.09.18.20186866
24. Christian SP. Analysis of the SARS-CoV-2 outbreak in Rio Grande do Sul / Brazil. arXiv Popul Evol. 2020.
25. Christielly Mendonca B, Marco Tulio Pacheco C, Jose Alexandre Felizola D-F, Thiago Fernando R. The necessary cooperation between governments and public in the fight against COVID-19: why non-pharmaceutical interventions may be ineffective. medRxiv. 2020. doi:10.1101/2020.08.17.20176347
26. Chu AMY, Tsang JTY, Chan JNL, Tiwari A, So MKP. Analysis of travel restrictions for COVID-19 control in Latin America through network connectedness. J Travel Med. 2020. doi:http://dx.doi.org/10.1093/jtm/taaa176
27. Ciampi E, Uribe-San-Martín R, Soler B, Fernández R, García P, Navarrete-Asenjo C, et al. COVID-19 in MS and NMOSD: A multicentric online national survey in Chile. Mult Scler Relat Disord. 2020;45: 102392. doi:10.1016/j.msard.2020.102392
28. Ciampi E, Uribe-San-Martin R, Carcamo C. COVID-19 pandemic: The experience of a multiple sclerosis centre in Chile. Mult Scler Relat Disord. 2020;42: 102204. doi:https://dx.doi.org/10.1016/j.msard.2020.102204
29. Cintra HPC, Fontinele FN. Estimative of real number of infections by COVID-19 in Brazil and possible scenarios. Infect Dis Model. 2020;5: 720–736. doi:http://dx.doi.org/10.1016/j.idm.2020.09.004

Pedro Henrique Pinheiro C, Felipe Fontinele N. Estimative of real number of infections by COVID-19 on Brazil and possible scenarios. medRxiv. 2020. doi:10.1101/2020.05.03.20052779

1. Civantos AM, Bertelli A, Goncalves A, Getzen E, Chang C, Long Q, et al. Mental health among head and neck surgeons in Brazil during the COVID-19 pandemic: A national study. Am J Otolaryngol - Head Neck Med Surg. 2020;41: 102694. doi:http://dx.doi.org/10.1016/j.amjoto.2020.102694
2. Clarice Ribeiro Alves C, Francisco das Chagas Bezerra N, Eliezio Nascimento B, Adryele Gomes M. Impactos da pandemia da covid-19 sob a égide do código de defesa do consumidor. Res Soc Dev. 2020;9. doi:10.33448/RSD-V9I6.3578
3. Clarisse Lins de L, Cecilia Cordeiro da S, Ana Clara Gomes da S, Eduardo Luiz S, Gabriel Souza M, Lucas Job Brito de A, et al. COVID-SGIS: A smart tool for dynamic monitoring and temporal forecasting of Covid-19. medRxiv. 2020. doi:10.1101/2020.05.30.20117945
4. Claudia R-R, Beatriz Piedad U. Modelling strategies to predict hospital demand during the COVID-19 outbreak in Bogota, Colombia. medRxiv. 2020. doi:10.1101/2020.04.14.20065466
5. Cobre AF, Boger B, Fachi MM, Vilhena RO, Domingos EL, Tonin FS, et al. Risk factors associated with delay in diagnosis and mortality in patients with COVID-19 in the city of Rio de Janeiro, Brazil. Cien Saude Colet. 2020;25: 4131–4140. doi:http://dx.doi.org/10.1590/1413-812320202510.2.26882020
6. Coelho FC, Lana RM, Cruz OG, Villela DAM, Bastos LS, Pastore Y Piontti A, et al. Assessing the spread of COVID-19 in Brazil: Mobility, morbidity and social vulnerability. PLoS One. 2020;15: e0238214. doi:https://dx.doi.org/10.1371/journal.pone.0238214
7. Cohen G, Russo MJ, Campos JA, Allegri RF. COVID-19 Epidemic in Argentina: Worsening of Behavioral Symptoms in Elderly Subjects With Dementia Living in the Community. Front Psychiatry. 2020;11: 866. doi:http://dx.doi.org/10.3389/fpsyt.2020.00866
8. Cohen G, Russo MJ, Campos JA, Allegri RF. Living with dementia: increased level of caregiver stress in times of COVID-19. Int psychogeriatrics. 2020; 1–11. doi:10.1017/S1041610220001593
9. Colunga-Salas P, Hernandez-Canchola G. Bats and humans during the SARS-CoV-2 outbreak: The case of bat-coronaviruses from Mexico. Transbound Emerg Dis. 2020. doi:http://dx.doi.org/10.1111/tbed.13751
10. Connerton P, Vicente de Assunção J, Maura de Miranda R, Dorothée Slovic A, José Pérez-Martínez P, Ribeiro H. Air Quality during COVID-19 in Four Megacities: Lessons and Challenges for Public Health. Int J Environ Res Public Health. 2020;17: 1–24. doi:10.3390/ijerph17145067
11. Conselho Nacional de Secretários de S. Segunda fase da pesquisa &quot;Evolução da Prevalência de Infecção por Covid-19 no Brasil: Estudo de Base Populacional&quot. 2020.
12. Conte D, Bahia L, Laurentino de Carvalho E, Monte Cardoso A, Souza PM. Public and private supply of beds and access to health care in the COVID-19 pandemic in Brazil. 2020.
13. Contreras S, Biron-Lattes JP, Villavicencio HA, Medina-Ortiz D, Llanovarced-Kawles N, Olivera-Nappa Á. Statistically-based methodology for revealing real contagion trends and correcting delay-induced errors in the assessment of COVID-19 pandemic. Chaos Solitons Fractals. 2020;139: 110087. doi:10.1016/j.chaos.2020.110087
14. Contreras-Rodríguez L, Sandoval-Hernández A, Maya-Hoyos M, Soto C. Remote learning of biochemistry during the COVID-19 pandemic: case of undergraduate students in Bogota, Colombia. ResearchSquare. 2020. doi:10.21203/rs.3.rs-72902/v1
15. Cordova-Lepe F, Gutierrez-Aguilar R, Gutierrez-Jara JP. Number of COVID-19 cases in Chile at 120 days with data at 21/03/2020 and threshold of daily effort to flatten the epi-curve. Medwave. 2020;20: e7861. doi:https://dx.doi.org/10.5867/medwave.2020.02.7861
16. Cornejo N, Candia P. Descripción de la aplicación de screening universal para SARS-CoV-2 en mujeres embarazadas que ingresan para interrupción en Hospital de Carabineros – Santiago de Chile. Rev Chil Obstet Ginecol. 2020;85: S2–S8.
17. Corpus-Mendoza AN, Ruiz-Segoviano HS, Rodriguez-Contreras SF, Yanez-Davila D, Hernandez-Granados A. Decrease of mobility, electricity demand, and NO2 emissions on COVID-19 times and their feedback on prevention measures. Sci Total Environ. 2020; 143382. doi:http://dx.doi.org/10.1016/j.scitotenv.2020.143382
18. Corrêa PRL, Ishitani LH, Abreu DMX, Teixeira RA, Marinho F, França EB. The importance of surveillance in cases of and mortality from the COVID-19 epidemic in Belo Horizonte, Brazil, 2020. Rev Bras Epidemiol. 2020;23: e200061. doi:10.1590/1980-549720200061
19. Corrêa PRL, Ishitani LH, Abreu DMX de, Teixeira RA, Marinho F, França EB. The importance of surveillance in cases and mortality from COVID-19 epidemic in Belo Horizonte, 2020. 2020.
20. Correa-Araneda F, Ulloa-Yañez A, Núñez D, Boyero L, Tonin A, Cornejo A, et al. Environmental determinants of COVID-19 transmission across a wide climatic gradient in Chile. ResearchSquare. 2020. doi:10.21203/rs.3.rs-30393/v1
21. Correa-Cuadros JP, Muñoz-Rodríguez MA. SARS-C0V.2/COVID-I9 in Colombia: tendencies, predictions, and tensions about health system. Rev salud pública. 2020;22: e386614–e386614.
22. Correa-Cuadros JP, Muñoz-Rodríguez MA. SARS-COV2/COVID-19 en Colombia: tendencias, predicciones y tensiones sobre el sistema sanitario. Rev salud pública. 2020;22: e386614–e386614.
23. Cortés JA, Espitia P, Rosero-Lasso YL. Citywide preparedness for a pandemic: A crosssectional survey of knowledge, attitudes, and practices about respiratory infection prevention in Bogotá, Colombia. Biomedica. 2020;40: 159–165. doi:10.7705/biomedica.5526
24. Cortes-Alvarez NY, Pineiro-Lamas R, Vuelvas-Olmos CR. Psychological effects and associated factors of COVID-19 in a Mexican sample. Disaster Med Public Health Prep. 2020; 1–27. doi:https://dx.doi.org/10.1017/dmp.2020.215
25. Cortés-Tellés A, López-Romero S, Mancilla-Ceballos R, Ortíz-Farías DL, Núñez-Caamal N, Figueroa-Hurtado E. Risk factors for mortality among hospitalized patients with COVID-19. An overview in Mexican population. Tuberc Respir Dis (Seoul). 2020. doi:10.4046/trd.2020.0095
26. Costa AN, de Sa ERA, Bezerra RDS, Souza JL, Lima F das CA. Constituents of buriti oil (Mauritia flexuosa L.) like inhibitors of the SARS-Coronavirus main peptidase: an investigation by docking and molecular dynamics. J Biomol Struct Dyn. 2020; 1–8. doi:https://dx.doi.org/10.1080/07391102.2020.1778538
27. Costa CLA, Costa TM, Barbosa Filho VC, Bandeira PFR, Siqueira RCL. Influence of social distancing on the physical activity level during the COVID-19 pandemic. Rev bras ativ fís saúde. 2020;25: 1–6.
28. Costa IPA, Maêda S, Teixeira L, Gomes CFS, Santos MD. Choosing a hospital assistance ship to fight the covid-19 pandemic. Rev Saude Publica. 2020;54: 79. doi:10.11606/s1518-8787.2020054002792
29. Costa MF. Health belief model for coronavirus infection risk determinants. Rev Saude Publica. 2020;54: 47. doi:https://dx.doi.org/10.11606/s1518-8787.2020054002494
30. Costa MA, Lui L, Santos RM dos, Curi RLC, Albuquerque CG de, Tavares SR, et al. Apontamentos sobre a dimensão territorial da pandemia da Covid-19 e os fatores que contribuem para aumentar a vulnerabilidade socioespacial nas unidades de desenvolvimento humano de áreas metropolitanas brasileiras. 2020. p. 59.
31. Costa SM, Lacerda GT de, Villafort RN, Silveira RL, Amaral MBF. What Do We Know About COVID-19?: Maxillofacial Surgeons Survey. J Craniofac Surg. 2020;31: e661–e663. doi:https://dx.doi.org/10.1097/SCS.0000000000006658
32. Costa SF, Buss L, Espinoza EPS, Vieira  Jr. JM, de Oliveira da Silva LC, de Souza RM, et al. Performance of a qualitative rapid chromatographic immunoassay to diagnose COVID-19 in patients in a middle-income country. J Clin Virol. 2020;131: 104592. doi:https://dx.doi.org/10.1016/j.jcv.2020.104592
33. Cota G, Freire ML, de Souza CS, Pedras MJ, Saliba JW, Faria V, et al. Diagnostic performance of commercially available COVID-19 serology tests in Brazil. Int J Infect Dis. 2020. doi:http://dx.doi.org/10.1016/j.ijid.2020.10.008
34. Cota W. Monitoring the number of COVID-19 cases and deaths in Brazil at municipal and federative units level. 2020.
35. Cotrin P, Bahls AC, da Silva DO, Pereira Girao VM, Maio Pinzan-Vercelino CR, de Oliveira RCG, et al. The use of facemasks during the COVID-19 pandemic by the Brazilian population. J Multidiscip Healthc. 2020;13: 1169–1178. doi:http://dx.doi.org/10.2147/JMDH.S281524
36. Cotrin P, Moura W, Gambardela-Tkacz CM, Pelloso FC, Santos LD, Carvalho MDB, et al. Healthcare Workers in Brazil during the COVID-19 Pandemic: A Cross-Sectional Online Survey. Inquiry. 2020;57: 46958020963711. doi:http://dx.doi.org/10.1177/0046958020963711
37. Cotrin P, Peloso RM, Pini NIP, Oliveira RC, de Oliveira RCG, Valarelli FP, et al. Urgencies and emergencies in orthodontics during the coronavirus disease 2019 pandemic: Brazilian orthodontists’ experience. Am J Orthod Dentofacial Orthop. 2020. doi:10.1016/j.ajodo.2020.06.028
38. Cotta RM, Naveira-Cotta CP, Magal P. Mathematical Parameters of the COVID-19 Epidemic in Brazil and Evaluation of the Impact of Different Public Health Measures. Biology (Basel). 2020;9. doi:10.3390/biology9080220

Renato Machado C, Carolina Palma N-C, pierre magal. Modelling the COVID-19 epidemics in Brasil: Parametric identification and public health measures influence. medRxiv. 2020. doi:10.1101/2020.03.31.20049130

1. Cristiana Costa G, Crispim  Jr. C, Eliana Z, Ethel Leonor Noia M, Filomena Euridice Carvalho de A, Gilton Luiz A, et al. A population-based study of the prevalence of COVID-19 infection in Espirito Santo, Brazil: methodology and results of the first stage. medRxiv. 2020. doi:10.1101/2020.06.13.20130559
2. Cristiane Coimbra de P, Joao Pedro Castoldo P, Walkiria Shimoya Bittencour I V, Caroline Aquino Vieira de  II L, Ruberlei Godinho de  Sr. O. PREVALENCE OF MOLECULAR AND SEROLOGICAL TESTS OF THE NEW CORONAVIRUS (SARS-CoV-2) IN CARLOS CHAGAS-SABIN LABORATORIES IN CUIABA. medRxiv. 2020. doi:10.1101/2020.10.26.20219683
3. Cristiane Ravagnani F, Thomas Nogueira V, Gabriel Berg de A, Claudia Pio F, Rejane Maria Tommasini G, Raul Borges G, et al. Impact of nonpharmaceutical governmental strategies for prevention and control of COVID-19 in Sao Paulo State, Brazil. medRxiv. 2020. doi:10.1101/2020.08.23.20180273
4. Cristiano Aguiar de O. Does “Staying at Home” Save Lives? An Estimation of the Impacts of Social Isolation in the Registered Cases and Deaths by COVID-19 in Brazil. SSRN. 2020. doi:10.2139/ssrn.3593947
5. Cristiano Aguiar de O, Rafael Mesquita P, Gabriel M. Using a Natural Experiment to Assess the Costs and Benefits of Intensifying Social Isolation. SSRN. 2020. doi:10.2139/ssrn.3660440
6. Cristiano E, Patrucco L, Rojas JI, Nunez S. Estimating the risk of COVID-19 in multiple sclerosis patients in Buenos Aires, Argentina. Mult Scler Relat Disord. 2020;44: 102307. doi:https://dx.doi.org/10.1016/j.msard.2020.102307
7. Cristóbal C, María José M, Jean G, María Paz B, Manuel N, Tania A, et al. Impact of small-area lockdowns for the control of the COVID-19 pandemic. medRxiv. 2020. doi:10.1101/2020.05.05.20092106
8. Cristy Leonor Azanza R, Esteban Abelardo Hernandez V. The Risk of Lifting COVID-19 Confinement in Mexico. medRxiv. 2020. doi:10.1101/2020.05.28.20115063
9. Crokidakis N. COVID-19 spreading in Rio de Janeiro, Brazil: Do the policies of social isolation really work? Chaos Solitons Fractals. 2020;136: 109930. doi:https://dx.doi.org/10.1016/j.chaos.2020.109930
10. Cruz CH de B. Social distancing in Sao Paulo State: demonstrating the reduction in cases using time series analysis of deaths due to COVID-19. Rev Bras Epidemiol. 2020;23: e200056. doi:https://dx.doi.org/10.1590/1980-549720200056

Carlos Henrique de Brito C. Social distancing in são paulo state: Demonstrating the reduction in cases using time series analysis of deaths due to covid-19/ Distanciamento social no estado de são paulo: Uso de série temporal dos óbitos por causa da covid-19 para demonstrar a redução. Rev Bras Epidemiol. 2020.

1. Cruz-Pacheco G, Bustamante-Castaneda JF, Caputo JG, Jimenez-Corona ME, Ponce-de-Leon-Rosales S. DISPERSION OF A NEW CORONAVIRUS SARS-COV-2 BY AIRLINES IN 2020: TEMPORAL ESTIMATES OF THE OUTBREAK IN MEXICO. Rev Invest Clin. 2020;72: 138–143. doi:https://dx.doi.org/10.24875/RIC.20000113
2. Cuartas DE, Arango-Londoño D, Guzmán-Escarria G, Muñoz E, Caicedo D, Ortega D, et al. SARS-coV-2 spatio - temporal analysis in Cali, Colombia. Rev salud pública. 2020;22: e286431–e286431.

Gissel G-E, Edgar M, Diana C, Delia O, Andrés F-L, Jorge M, et al. Análisis espacio-temporal del SARS-COV-2 en Cali, Colombia/ SARS-coV-2 spatio - temporal analysis in Cali, Colombia. 2020;22.

1. Cuenca-Jaque CR, Osorio-Tarrillo ML, Pastor-Ramos JL, G DELPP-P, Torres-Vasquez LE. Economic and health aspects in quarantine times per covid 19 in the peruvian population, year 2020. Rev la Fac Med Humana. 2020;20: 630–639. doi:http://dx.doi.org/10.25176/RFMH.V20I4.3067
2. Cuesta J, Pico J. The Gendered Poverty Effects of the COVID-19 Pandemic in Colombia. Eur J Dev Res. 2020; 1–34. doi:10.1057/s41287-020-00328-2
3. Cuevas-Barragan CE, Buenrostro-Nava MT, Palos-Gomez GM, Ramirez-Padilla EA, Mendoza-Macias BI, Rivas-Caceres RR. Use of Nasoil via intranasal to control the harmful effects of Covid-19. Microb Pathog. 2020;149: 104504. doi:http://dx.doi.org/10.1016/j.micpath.2020.104504
4. Da Cunha de Sa-Caputo D, Sonza A, Bachur JA, Bernardo-Filho M. Development, validation and reliabilty of a questionnaire to evaluate the changes on the level of physical exercises and in daily life habits due to COVID-19 pandemic social distancing. Acta Biomed. 2020;91: e2020004. doi:https://dx.doi.org/10.23750/abm.v91i3.9888
5. da Cunha AR, Antunes JLF, Martins MD, Petti S, Hugo FN. The impact of the COVID-19 pandemic on oral biopsies in the Brazilian National Health System. Oral Dis. 2020. doi:http://dx.doi.org/10.1111/odi.13620
6. da Silva Dittz E, Leão da Silveira Rocha A. The repercussions in daily routine of mothers of babies admitted in Neonatal Intensive Care Unit during the social isolation to avoid COVID-19. 2020.
7. da Silva RG, Ribeiro M, Mariani VC, Coelho LDS. Forecasting Brazilian and American COVID-19 cases based on artificial intelligence coupled with climatic exogenous variables. Chaos Solitons Fractals. 2020;139: 110027. doi:10.1016/j.chaos.2020.110027
8. Dal’Bosco EB, Floriano LSM, Skupien S V, Arcaro G, Martins AR, Anselmo ACC. Mental health of nursing in coping with COVID-19 at a regional university hospital. Rev Bras Enferm. 2020;73 2: e20200434. doi:http://dx.doi.org/10.1590/0034-7167-2020-0434
9. Dalton Garcia Borges de S, Francisco Tarcísio Alves J, Nei Yoshihiro S. Forecasting COVID-19 cases at the Amazon region: a comparison of classical and machine learning models. bioRxiv. 2020. doi:10.1101/2020.10.09.332908
10. Dan M-K, Bruno B-T, Mercedes M-G, Andrea Zaldivar-Perez P, Juan OT. U-shaped-aggressiveness of SARS-CoV-2: Period between onset of nonspecific-specific symptoms for COVID-19. A population-based cohort study. medRxiv. 2020. doi:10.1101/2020.10.28.20221697
11. Daniel AA, Andrea B. Emerging Mental Health Challenges, Strategies and Opportunities in the context of the COVID-19 Pandemic: Perspectives from South American Decision-makers. medRxiv. 2020. doi:10.1101/2020.07.16.20155630
12. Daniel CPJ, Moreno SR, Mateus SS, Luciana LC, Nivea B da S, Ismael HS, et al. Assessing the nationwide impact of COVID-19 mitigation policies on the transmission rate of SARS-CoV-2 in Brazil. medRxiv. 2020. doi:10.1101/2020.06.26.20140780
13. Daniel G-C, Daniel R-A, Marco B, Juan G, Daniel M, Narcisa G, et al. Adapting for the COVID-19 pandemic in Ecuador, a characterization of hospital strategies and patients. medRxiv. 2020. doi:10.1101/2020.07.25.20161661
14. Daniele Melo S, Karla VBL, Ana LSF, Juliana CDG, Thalyta MRLU, Yan Correa R, et al. Clinical characteristics of Severe Acute Respiratory Syndrome by COVID-19 in Indigenous of Brazil. medRxiv. 2020. doi:10.1101/2020.10.24.20218701
15. Danilo F, Claudia G, Leyda EA, Jean PC, Yamilka D, Yaset C, et al. Early transmission dynamics, spread, and genomic characterization of SARS-CoV-2 in Panama. medRxiv. 2020. doi:10.1101/2020.07.31.20160929
16. Dantas F. Outcomes research of homeopathic treatment in suspected or confirmed patients with COVID-19 IN Brazil: Clinical research protocol. 2020.

Dantas F. Resultados terapêuticos da homeopatia em pacientes suspeitos ou confirmados de COVID-19 no Brasil: Protocolo para estudo observacional prospectivo. 2020. p. 46.

1. Dantas G, Siciliano B, Franca BB, da Silva CM, Arbilla G. The impact of COVID-19 partial lockdown on the air quality of the city of Rio de Janeiro, Brazil. Sci Total Environ. 2020;729: 139085. doi:https://dx.doi.org/10.1016/j.scitotenv.2020.139085
2. Daon Y, Thompson RN, Obolski U. Estimating COVID-19 outbreak risk through air travel. J Travel Med. 2020. doi:https://dx.doi.org/10.1093/jtm/taaa093
3. David de Paulo F, Fábio Fernandes de A. Will COVID-19 affect food supply in distribution centers of Brazilian regions affected by the pandemic? Trends Food Sci Technol. 2020;103: 361–366. doi:10.1016/J.TIFS.2020.05.023
4. Dayrell A, Cristina de Souza Andrade A, Leandro Machado E, Lúcia Meireles A, Silva Magalhães A, do Prado Ribeiro E, et al. Through the COVID-19 magnifying glass: an examination of disparities in the Brazilian capitals. 2020.
5. de Alencar JCG, Moreira CL, Muller AD, Chaves CE, Fukuhara MA, da Silva EA, et al. Double-blind, randomized, placebo-controlled trial with N-acetylcysteine for treatment of severe acute respiratory syndrome caused by COVID-19. Clin Infect Dis. 2020. doi:http://dx.doi.org/10.1093/cid/ciaa1443
6. De Boni RB, Balanza-Martinez V, Mota JC, Cardoso TA, Ballester P, Carbonell B, et al. Depression, Anxiety and Lifestyle Among Essential Workers: A Websurvey From Brazil And Spain During The Covid-19 Pandemic. J Med Internet Res. 2020. doi:http://dx.doi.org/10.2196/22835
7. de Brito CAA, de Brito MCM, Fernandes Martins TH, de Brito CCM, Militao Albuquerque MF, de Cassia Coelho Moraes de Brito R. Clinical laboratory and dispersion pattern of COVID-19 in a family cluster in the social-distancing period. J Infect Dev Ctries. 2020;14: 987–993. doi:http://dx.doi.org/10.3855/JIDC.13580
8. de Farias ECF, Pedro Piva J, de Mello M, do Nascimento L, Costa CC, Machado MMM, et al. Multisystem Inflammatory Syndrome Associated With Coronavirus Disease in Children: A Multi-centered Study in Belém, Pará, Brazil. Pediatr Infect Dis J. 2020. doi:10.1097/INF.0000000000002865
9. De la Hoz-Restrepo F, Alvis-Zakzuk NJ, De la Hoz-Gomez JF, De la Hoz A, Gomez Del Corral L, Alvis-Guzman N. Is Colombia an example of successful containment of the 2020 COVID-19 pandemic? A critical analysis of the epidemiological data, March to July 2020. Int J Infect Dis. 2020;99: 522–529. doi:http://dx.doi.org/10.1016/j.ijid.2020.08.017
10. de la Miyar JRB, Hoehn-Velasco L, Silverio-Murillo A. Druglords Don’t stay at home: COVID-19 pandemic and crime patterns in Mexico City. J Crim Justice. 2020; 101745. doi:10.1016/j.jcrimjus.2020.101745
11. de Lima Filho BF, Bessa NPOS, Fernandes ACT, da Silva Patricio IF, de Oliveira Alves N, da Costa Cavalcanti FA. Knowledge levels among elderly people with Diabetes Mellitus concerning COVID-19: an educational intervention via a teleservice. Acta Diabetol. 2020. doi:http://dx.doi.org/10.1007/s00592-020-01580-y
12. de Lucena EHG, Freire AR, Freire DEWG, de Araujo ECF, Lira GNW, Brito ACM, et al. Offer and use of oral health in primary care before and after the beginning of the COVID-19 pandemic in Brazil. Pesqui Bras Odontopediatria Clin Integr. 2020;20: 1–6. doi:http://dx.doi.org/10.1590/pboci.2020.163
13. de Maio Nascimento M. Covid-19: U3A students’ report on the impacts of social isolation on physical and mental health and access to information about the virus during the pandemic. Educ Gerontol. 2020;46: 499–511. doi:10.1080/03601277.2020.1795371
14. de Mattos Matheus AS, Cabizuca CA, Tannus LRM, Passos AC, Schmidt AC, de Gouveia AT, et al. Telemonitoring type 1 diabetes patients during the COVID-19 pandemic in Brazil: was it useful? Arch Endocrinol Metab. 2020. doi:10.20945/2359-3997000000309
15. de Melo T, Figueiredo CMS. A first public dataset from Brazilian twitter and news on COVID-19 in Portuguese. Data Br. 2020;32: 106179. doi:10.1016/j.dib.2020.106179
16. de Sousa LE, Neto PHO, Filho DADS. Kinetic Monte Carlo model for the COVID-19 epidemic: Impact of mobility restriction on a COVID-19 outbreak. Phys Rev E. 2020;102: 32133. doi:http://dx.doi.org/10.1103/PhysRevE.102.032133
17. de Souza Ferreira LP, Valente TM, Tiraboschi FA, da Silva GPF. Description of Covid-19 Cases in Brazil and Italy. SN Compr Clin Med. 2020; 1–4. doi:https://dx.doi.org/10.1007/s42399-020-00307-y
18. de Souza Luna LK, Perosa DAH, Conte DD, Carvalho JMA, Alves VRG, Cruz JS, et al. Different patterns of Influenza A and B detected during early stages of COVID-19 in a university hospital in Sao Paulo, Brazil. J Infect. 2020. doi:https://dx.doi.org/10.1016/j.jinf.2020.05.036
19. de Souza CDF, de Arruda Magalhaes AJ, Lima AJPD, Nunes DN, de Fatima Machado Soares E, de Castro Silva L, et al. Clinical manifestations and factors associated with mortality from COVID-19 in older adults: Retrospective population-based study with 9807 older Brazilian COVID-19 patients. Geriatr Gerontol Int. 2020. doi:http://dx.doi.org/10.1111/ggi.14061
20. de Souza CDF, do Carmo RF, Machado MF. The burden of COVID-19 in Brazil is greater in areas with high social deprivation. J Travel Med. 2020. doi:http://dx.doi.org/10.1093/jtm/taaa145
21. de Souza CDF, Machado MF, do Carmo RF. Human development, social vulnerability and COVID-19 in Brazil: a study of the social determinants of health. Infect Dis poverty. 2020;9: 124. doi:https://dx.doi.org/10.1186/s40249-020-00743-x
22. de Souza WM, Buss LF, Candido DDS, Carrera JP, Li S, Zarebski AE, et al. Epidemiological and clinical characteristics of the COVID-19 epidemic in Brazil. Nat Hum Behav. 2020. doi:10.1038/s41562-020-0928-4

William Marciel de S, Lewis Fletcher B, Darlan da Silva C, Jean Paul C, Sabrina L, Alexander Z, et al. Epidemiological and clinical characteristics of the early phase of the COVID-19 epidemic in Brazil. medRxiv. 2020. doi:10.1101/2020.04.25.20077396

1. Del Brutto OH, Costa AF, Mera RM, Recalde BY, Bustos JA, Garcia HH. Late incidence of SARS-CoV-2 infection in a highly-endemic remote rural village. A prospective population-based cohort study. Pathog Glob Health. 2020. doi:http://dx.doi.org/10.1080/20477724.2020.1826152
2. Del Brutto OH, Costa AF, Mera RM, Recalde BY, Bustos JA, Garcia HH. SARS-CoV-2-related mortality in a rural Latin American population. Int J Infect Dis. 2020;99: 226–228. doi:http://dx.doi.org/10.1016/j.ijid.2020.08.003
3. Del Brutto OH, Costa AF, Mera RM, Recalde BY, Bustos JA, García HH. Household Clustering of SARS-CoV-2 in Community Settings: A Study from Rural Ecuador. Am J Trop Med Hyg. 2020. doi:10.4269/ajtmh.20-0688
4. Del Brutto OH, Costa AF, Mera RM, Recalde BY, Bustos JA, García HH. SARS-CoV-2 in rural Latin America. A population-based study in coastal Ecuador. Clin Infect Dis. 2020. doi:10.1093/cid/ciaa1055
5. Del Brutto OH, Costa AF, Recalde BY, Mera RM. Frailty and SARS-CoV-2 infection. A population-based study in a highly endemic village. J Neurol Sci. 2020;418: 117136. doi:http://dx.doi.org/10.1016/j.jns.2020.117136
6. Del Brutto OH, Mera RM, Recalde BY, Costa AF. Social Determinants of Health and Risk of SARS-CoV-2 Infection in Community-Dwelling Older Adults Living in a Rural Latin American Setting. J Community Health. 2020. doi:10.1007/s10900-020-00887-9
7. Delgado D, Wyss Quintana F, Perez G, Sosa Liprandi A, Ponte-Negretti C, Mendoza I, et al. Personal Safety during the COVID-19 Pandemic: Realities and Perspectives of Healthcare Workers in Latin America. Int J Environ Res Public Health. 2020;17. doi:https://dx.doi.org/10.3390/ijerph17082798
8. Delgado-Enciso I, Paz-Garcia J, Barajas-Saucedo C, Mokay-Ramírez K, Meza-Robles C, Lopez-Flores R, et al. Patient-Reported Health Outcomes After Treatment of COVID-19 with Nebulized and/or Intravenous Neutral Electrolyzed Saline Combined with Usual Medical Care Versus Usual Medical care alone: A Randomized, Open-Label, Controlled Trial. ResearchSquare. 2020. doi:10.21203/rs.3.rs-68403/v1
9. Delgado-Gallegos JL, Montemayor-Garza RJ, Padilla-Rivas GR, Franco-Villareal H, Islas JF. Prevalence of stress in healthcare professionals during the covid-19 pandemic in Northeast Mexico: A remote, fast survey evaluation, using an adapted covid-19 stress scales. Int J Environ Res Public Health. 2020;17: 1–12. doi:http://dx.doi.org/10.3390/ijerph17207624
10. Demenech LM, Dumith S de C, Vieira MECD, Neiva-Silva L. Income inequality and risk of infection and death by COVID-19 in Brazil. Desigual Econ e risco Infecc e morte por COVID-19 no Bras. 2020;23: e200095. doi:https://dx.doi.org/10.1590/1980-549720200095
11. Denova-Gutiérrez E, Lopez-Gatell H, Alomia-Zegarra JL, López-Ridaura R, Zaragoza-Jimenez CA, Dyer-Leal DD, et al. The association between obesity, type 2 diabetes, and hypertension with severe COVID-19 on admission among Mexicans. Obesity (Silver Spring). 2020;28: 1826–1832. doi:10.1002/oby.22946
12. Di Mascio D. Maternal and Perinatal Outcomes of Pregnant Women with SARS-COV-2 infection. Ultrasound Obstet Gynecol. 2020. doi:http://dx.doi.org/10.1002/uog.23107
13. Di Tommaso F, Maurice MF, Sastre P, Prado AH, Domine E, Aguero P, et al. Whatsapp consultations in the department of electrophysiology of a public hospital of the City of Buenos Aires in times of COVID-19. Rev Argent Cardiol. 2020;88: 225–228. doi:http://dx.doi.org/10.7775/rac.v88.i3.17976
14. Di M, Gonzalo y otros. PROTECCIÓN Y SITUACIÓN LABORAL DE LOS MÉDICOS ONCÓLOGOS DURANTE LA PANDEMIA DE COVID-19 EN LA ARGENTINA. 2020;8.
15. Diana C, Lenice de Castro Muniz de Q, Lílian Moura de Lima S, Tuany Nunes C, Deisi Cardoso S. Educational actions to combat COVID-19: experience report. Res Soc Dev. 2020;9. doi:10.33448/RSD-V9I8.5207
16. Diana MR-G, Sandra CG-C, Natalia M, Erika VJ-P, Jaime AC-O. COVID-19 in Latin America: Contrasting phylodynamic inference with epidemiological surveillance. medRxiv. 2020. doi:10.1101/2020.05.23.20111443
17. Díaz Castrillón C, Cortés N, Rey S, Pineda M, Díaz Castrillón J, Sierra S. Percepción de la pandemia COVID-19 en los servicios de cirugía en Colombia. Rev colomb cir. 2020;35: 290–301.
18. Díaz Castrillón C, Cortés N, Rey S, Pineda M, Díaz Castrillón J, Sierra S. Perception of the COVID-19 pandemic in the departments of surgery in Colombia. Rev colomb cir. 2020;35: 290–301.
19. Díaz Morales Y, Hidalgo Mesa CJ, Reyes Roque AC, Filgueira Morilla LM, Tamayo Díaz YB, Aguilera Calzadilla Y. Training for professionals and other health workers as regard as hospital management of COVID-19. Edumecentro. 2020;12: 8–24.
20. Díaz FJ, Aguilar-Jiménez W, Flórez-Álvarez L, Valencia G, Laiton-Donato K, Franco-Muñoz C, et al. Isolation and characterization of an early SARS-CoV-2 isolate from the 2020 epidemic in Medellín, Colombia. Biomedica. 2020;40: 148–158. doi:10.7705/biomedica.5834
21. Diaz LA, Garcia-Salum T, Fuentes-Lopez E, Ferres M, Medina RA, Riquelme A, et al. Symptom profiles and risk factors for hospitalization in patients with SARS-CoV-2 and COVID-19: A Large Cohort from South America. Gastroenterology. 2020. doi:https://dx.doi.org/10.1053/j.gastro.2020.05.014
22. Diaz-Corvillon P, Monckeberg M, Barros A, Illanes SE, Soldati A, Nien J-K, et al. Routine screening for SARS CoV-2 in unselected pregnant women at delivery. PLoS One. 2020;15: e0239887. doi:https://dx.doi.org/10.1371/journal.pone.0239887
23. Diaz-Guio DA, Ricardo-Zapata A, Ospina-Velez J, Gomez-Candamil G, Mora-Martinez S, Rodriguez-Morales AJ. Cognitive load and performance of health care professionals in donning and doffing PPE before and after a simulation-based educational intervention and its implications during the COVID-19 pandemic for biosafety. Le Infez Med. 2020;28: 111–117.
24. Díaz-Narváez V, San-Martín-Roldán D, Calzadilla-Núñez A, San-Martín-Roldán P, Parody-Muñoz A, Robledo-Veloso G. Which curve provides the best explanation of the growth in confirmed COVID-19 cases in Chile? Rev latinoam enferm. 2020;28: e3346–e3346.
25. Díaz-Pinzón JE. Accuracy of COVID-19 propagation forecast in Colombia. Reper med cir. 2020;29: 27–33.
26. Díaz-Pinzón JE. Análysis of the geographic distribution of COVID-19 cases in Colombia. Reper med cir. 2020;29: 59–64.
27. Díaz-Pinzón JE. Calculation of COVID-19 infection and recovery ratio and proportionality in Colombia. Reper med cir. 2020;29: 83–88.
28. Díaz-Pinzón JE. Comparative study on the COVID-19 contagion rate during the mandatory quarantine and during the gradual and controlled reopening of some sectors of the economy in Colombia. Reper med cir. 2020;29: 52–58.
29. Díaz-Pinzón JE. Statistical analysis of COVID-19 cases according to age group in Colombia. Reper med cir. 2020;29: 76–82.
30. Díaz-Pinzón JE. Use of predictive modeling for estimating Covid-19 transmission dynamics in Colombia. Reper med cir. 2020;29: 34–44.
31. Diaz-Quijano FA, da Silva JMN, Ganem F, Oliveira S, Vesga-Varela AL, Croda J. A model to predict SARS-CoV-2 infection based on the first three-month surveillance data in Brazil. Trop Med Int Health. 2020. doi:10.1111/tmi.13476
32. Díaz-Salazar C, Sánchez-García A, Rodríguez-Gutiérrez R, Camacho-Ortiz A, Saldívar-Rodríguez D, González-González J. Prevalence and associated characteristics of anti-SARS-CoV-2 antibodies in Mexico 5 months after pandemic arrival. ResearchSquare. 2020. doi:10.21203/rs.3.rs-84890/v1
33. Diego Tavares V, Anna Claudia Mello R, Lucas A, Joao Vitor Oliveira S, Claudia Mazza D, Regina Cerqueira A, et al. Spreading of COVID-19 in Brazil: Impacts and uncertainties in social distancing strategies. medRxiv. 2020. doi:10.1101/2020.05.30.20117283
34. Diego O, Maria Mora Gonzalez Lopez L, Horacio P, Guadalupe Costa N, Lautaro S, Beatriz P, et al. Emergency Response for Evaluating SARS-CoV-2 Immune Status, Seroprevalence and Convalescent Plasma in Argentina. medRxiv. 2020. doi:10.1101/2020.10.21.20216960
35. Diegoli H, Magalhaes PSC, Martins SCO, Moro CHC, Franca PHC, Safanelli J, et al. Decrease in Hospital Admissions for Transient Ischemic Attack, Mild, and Moderate Stroke During the COVID-19 Era. Stroke. 2020; STROKEAHA120030481. doi:https://dx.doi.org/10.1161/STROKEAHA.120.030481
36. Dimitris B, Alison B, Luca M, Omid N, Agni O, Bartolomeo S, et al. Personalized Prescription of ACEI/ARBs for Hypertensive COVID-19 Patients. medRxiv. 2020. doi:10.1101/2020.10.30.20223594
37. D’Imperio H, Gagliardi J, Zoni R, Charask A, Costa YC, Marturan MP, et al. Results of the covid-19 survey on the impact on cardiovascular care. National infarction registry: Argen iam-st. Rev la Fed Argentina Cardiol. 2020;49: 69–73.

D’Imperio H, Gagliardi J, Zoni R, Charask A, Costa YC, Marturano MP, et al. Results of the COVID-19 survey. Impact on cardiovascular care in the argen-iam-st national infarction registry. Rev Argent Cardiol. 2020;88: 217–224. doi:http://dx.doi.org/10.7775/rac.v88.i3.18150

1. Djilali S, Ghanbari B. Coronavirus pandemic: A predictive analysis of the peak outbreak epidemic in South Africa, Turkey, and Brazil. Chaos Solitons Fractals. 2020;138: 109971. doi:https://dx.doi.org/10.1016/j.chaos.2020.109971
2. Dolce Filho R, Nechar RC, Ribeiro Filho A. Preliminary study to assess symptoms and prevalent drugs of the “epidemic genius” of the COVID-19 pandemic in Brazil. 2020; 21.
3. Dolhnikoff M, Duarte-Neto AN, de Almeida Monteiro RA, da Silva LFF, de Oliveira EP, Saldiva PHN, et al. Pathological evidence of pulmonary thrombotic phenomena in severe COVID-19. J Thromb Haemost. 2020;18: 1517–1519. doi:http://dx.doi.org/10.1111/jth.14844
4. Douglas F, Paula F, Paulo  Jr. O, Jennifer R, Elicardo G, Andres P. Temporal and spatial characteristics of the spread of COVID-19 in Rio de Janeiro state and city. medRxiv. 2020. doi:10.1101/2020.05.13.20101113
5. Dowall MPM, Barrionuevo-Poquet A, Carnero-Fuentes O, Pareja-Begazo G, Coayla-Cano C, Gallo-Lopez A, et al. Clinical-pathological characterization, viral genotipification and genetic heterogeneity as risk determinants in covid-19: Study design and initial findings. Rev la Fac Med Humana. 2020;20: 433–443. doi:http://dx.doi.org/10.25176/RFMH.v20i3.3040
6. Duarte MMS, Haslett MIC, Freitas LJA de, Gomes NTN, Silva DCC da, Percio J, et al. Description of COVID-19 hospitalized health worker cases in the first nine weeks of the pandemic, Brazil, 2020. Descricao dos casos Hosp pela COVID-19 em profissionais saude nas Prim nove Sem da pandemia, Bras 2020. 2020;29: e2020277. doi:https://dx.doi.org/10.1590/S1679-49742020000500011
7. Duarte M de Q, Santo MA da S, Lima CP, Giordani JP, Trentini CM. Covid-19 and the impacts on mental health: a sample from Rio Grande do Sul, Brazil. COVID-19 e os impactos na saude Ment uma amostra do Rio Gd do Sul, Bras. 2020;25: 3401–3411. doi:https://dx.doi.org/10.1590/1413-81232020259.16472020
8. Duarte RB, Medeiros LMF, Araújo MJA de M, Cavalcante ASP, Souza EC de, Alencar OM de, et al. Community health agents in front of COVID-19: experiences with nursing professionals. Enferm foco. 2020;11: 252–256.
9. Duarte-Filho GC, Brum AA, Ospina R, Almeida FAG, Macêdo AMS, Vasconcelos GL. Recife and Belém are currently the only capitals that are already in the saturation phase of Covid-19 in Brazil. 2020.
10. Duarte-Filho GC, Brum AA, Ospina R, Almeida FAG, Macêdo AMS, Vasconcelos GL. Situation of the Covid-19 Epidemic in Brazil in August 2020: Most Northern and Northeastern States in a Saturation Phase, while Southern States in Acceleration or Slight Deceleration. 2020.
11. Duarte-Neto AN, Monteiro RAA, da Silva LFF, Malheiros DMAC, de Oliveira EP, Theodoro-Filho J, et al. Pulmonary and systemic involvement in COVID-19 patients assessed with ultrasound-guided minimally invasive autopsy. Histopathology. 2020;77: 186–197. doi:http://dx.doi.org/10.1111/his.14160
12. Duczmal LH, Almeida ACL, Duczmal DB, Alves CRL, Magalhaes FCO, Lima MS de, et al. Vertical social distancing policy is ineffective to contain the COVID-19 pandemic. Cad Saude Publica. 2020;36: e00084420. doi:https://dx.doi.org/10.1590/0102-311x00084420
13. Durán Morera N, Botello Ramírez E. Detection of emerging “active” clusters with high incidence rates, for rapid surveillance of COVID-19. Medicentro (Villa Clara). 2020;24: 642–655.
14. Eddy L-N, Paola S-P, Santiago R-V, Jose L-N, Paul V-M, Giuseppe B-B, et al. Paradigms About the COVID-19 Pandemic: What Medical Students Know and Feel. SSRN. 2020. doi:10.2139/ssrn.3594642

Eddy L-N, Paola S-P, Santiago R-V, Jose L-N, Paul V-M, Giuseppe B-B, et al. Paradigms about the COVID-2 pandemic: knowledge, attitudes and practices from medical students. medRxiv. 2020. doi:10.1101/2020.05.21.20105858

1. Edilson C. The SARS-COV-2 outbreak around the Amazon rainforest: the relevance of the airborne transmission. medRxiv. 2020. doi:10.1101/2020.08.06.20169433
2. Eduardo Atem De C, Rogerio Atem De C. Identification of Patterns in Epidemic Cycles and Methods for Estimating Their Duration: COVID-19 Case Study. medRxiv. 2020. doi:10.1101/2020.07.13.20153080
3. Eduardo Juscamayta L, David T, Faviola Valdivia G, Nancy Rojas S, Dennis C, Lenin Maturrano H, et al. Phylogenomic reveals multiple introductions and early spread of SARS-CoV-2 into Peru. bioRxiv. 2020. doi:10.1101/2020.09.14.296814
4. Eduardo AU, Gerardo C, Kenji M. Case fatality risk by age from COVID-19 in a high testing setting in Latin America: Chile, March-May, 2020. medRxiv. 2020. doi:10.1101/2020.05.25.20112904
5. Edwin  Sr. NBR, Anthony  Sr. JFE, Fernando EAL, oacutepez  Sr., Pa, uacutel HV, et al. Environmental and social analysis as risk factors for the spread of the novel coronavirus (SARS-CoV-2) using remote sensing, GIS and analytical hierarchy process (AHP): Case of Peru. medRxiv. 2020. doi:10.1101/2020.05.31.20118653
6. Efren M-Z, Oliver M-C, Ivan D-E, Carlos MH-S. Predictors of severe symptomatic laboratory-confirmed SARS-COV-2 reinfection. medRxiv. 2020. doi:10.1101/2020.10.14.20212720
7. Elachola H, Gozzer E, Rahman NMM, Ditekemena J, Pando-Robles V, Pa K, et al. Photo-epidemiology to estimate face covering use in select areas in Asia versus the Americas and Africa during the COVID-19 pandemic. J Travel Med. 2020. doi:10.1093/jtm/taaa121
8. Eliandro Rodrigues C, Miguel C, Paulo N, Neyva R, Jeinny P, Austin W, et al. One Study of COVID-19 Spreading at The United States - Brazil - Colombia. medRxiv. 2020. doi:10.1101/2020.08.29.20184465
9. Elías-Gutiérrez M, Mendoza-Carranza M, Takahashi-Aguilar U, Valdez-Moreno M, Prado-Bernal J, Mora-Loya C. Why does the COVID-19 outbreak in Mexico seem to be slower? Some hypotheses for testing. ResearchSquare. 2020. doi:10.21203/rs.3.rs-51450/v1
10. Elsa Berenice G-P, Verónica M-R, Ignacio M-G-H, Luz María G-A, Marco Antonio Á-A, Lucía C-N, et al. Vulnerabilidad, conocimiento sobre medidas de mitigación y exposición ante COVID-19 en adultos de México: Resultados de la ENSARS-CoV-2. Salud Publica Mex. 2020. doi:10.21149/11875
11. Elvira Maria G-S, Simone Schneider W, Clovis P, Guilherme Wataru G, Eduardo Jun S, Tiago Borges Ribeiro G, et al. Overview on COVID-19 outbreak indicators across Brazilian federative units. medRxiv. 2020. doi:10.1101/2020.06.02.20120220
12. Emer C, Maia KP, Santana PC, Santana EM, Silva DGM da, Cosmo LG, et al. Vulnerability of the micro-regions of the South of Brazil to the new coronavirus (SARS-CoV-2) pandemic. 2020.
13. Emerson Lucas Silva C, Bruna Isabela Adolpho de O, Igor Fessina S, Anderson Reis de S, Jules Ramon Brito T, Isabel Amelia Costa M, et al. LOW PSYCHOLOGICAL WELL-BEING IN MEN WHO HAVE SEX WITH MEN (MSM) DURING THE SHELTER-IN-PLACE ORDERS TO PREVENT THE COVID-19 SPREAD: RESULTS FROM A NATIONWIDE STUDY. medRxiv. 2020. doi:10.1101/2020.09.21.20198929
14. Emilio G, Adrian R, Tiago T. Information and Behavioral Responses during a Pandemic: Evidence from Delays in COVID-19 Death Reports. SSRN. 2020. doi:10.2139/ssrn.3645317
15. Endo PT, Silva I, Lima L, Bezerra L, Gomes R, Ribeiro-Dantas M, et al. #StayHome: Monitoring and benchmarking social isolation trends in Caruaru and the Região Metropolitana do Recife during the COVID-19 pandemic. Rev Soc Bras Med Trop. 2020;53: e20200271. doi:10.1590/0037-8682-0271-2020
16. Enner A, Jose M, Luiz R, Edward P, Thanan R, Fernando C, et al. Investigating spatiotemporal patterns of the COVID-19 in Sao Paulo State, Brazil. medRxiv. 2020. doi:10.1101/2020.05.28.20115626
17. Eric Luis Barroso C, Juliana Cristina Cardoso F. Surge capacities and predicted demands of Brazil’s health system associated with severe COVID-19 cases. medRxiv. 2020. doi:10.1101/2020.04.02.20050351
18. Ernandes Gonçalves D. Casos confirmados do novo Coronavírus: interiorização da doença em um município de Minas Gerais. J Nurs Heal. 2020;10: 1–12.
19. Escalera-Antezana JP, Lizon-Ferrufino NF, Maldonado-Alanoca A, Alarcón-De-la-Vega G, Alvarado-Arnez LE, Balderrama-Saavedra MA, et al. Clinical features of cases and a cluster of Coronavirus Disease 2019 (COVID-19) in Bolivia imported from Italy and Spain. Travel Med Infect Dis. 2020;35: 101653. doi:10.1016/j.tmaid.2020.101653

Escalera-Antezana JP, Lizon-Ferrufino NF, Maldonado-Alanoca A, Alarcon-De-la-Vega G, Alvarado-Arnez LE, Balderrama-Saavedra MA, et al. Clinical features of the first cases and a cluster of Coronavirus Disease 2019 (COVID-19) in Bolivia imported from Italy and Spain. Travel Med Infect Dis. 2020;35: 101653. doi:https://dx.doi.org/10.1016/j.tmaid.2020.101653

1. Escalera-Antezana JP, Cerruto-Zelaya PE, Apaza-Huasco M, Miranda-Rojas SH, Flores-Cardenas CA, Rivera-Zabala L, et al. Healthcare workers’ and students’ knowledge regarding the transmission, epidemiology and symptoms of COVID-19 in 41 cities of Bolivia and Colombia. Travel Med Infect Dis. 2020; 101702. doi:https://dx.doi.org/10.1016/j.tmaid.2020.101702
2. Escalera-Antezana JP, Lizon-Ferrufino NF, Maldonado-Alanoca A, Alarcon-De-la-Vega G, Alvarado-Arnez LE, Balderrama-Saavedra MA, et al. Risk factors for mortality in patients with Coronavirus Disease 2019 (COVID-19) in Bolivia: An analysis of the first 107 confirmed cases. Le Infez Med. 2020;28: 238–242.
3. Escobar G, Matta J, Taype Huamaní W, Ayala R, Amado J. Clinical - Epidemiological Characteristics Of Patients Deceased By COVID-19 In A National Hospital In Lima, Peru. Rev Fac Med Hum. 2020;20: 180–185.
4. Escosteguy CC, Eleuterio T de A, Pereira AGL, Marques MRVE, Brandão AD, Batista JPM. COVID-19: sectional study of suspected cases admitted to a federal hospital in Rio de Janeiro, Brazil, and factors associated with hospital death. 2020. doi:10.1590/s1679-49742021000100022
5. Espinosa MM, Oliveira EC de, Melo JS, Damaceno RD, Terças-Trette ACP. Prediction of COVID -19 cases and deaths in Mato Grosso state and Brazil. J Heal Biol Sci. 2020;8.
6. Esposito M, Bustos S, Cardonetti L, Alvarado MJP, Latorre B, Parrilla L, et al. Community isolation as a strategy to mitigate a COVID19 outbreak: a case study of Villa Azul, Argentina. 2020.
7. Esquivel-Moynelo I, Perez-Escribano J, Duncan-Robert Y, Vazque-Blonquist D, Bequet-Romero M, Baez-Rodriguez L, et al. Effect and safety of combination of interferon alpha-2b and gamma or interferon alpha-2b for negativization of SARS-CoV-2 viral RNA. Preliminary results of a randomized controlled clinical trial. medRxiv. 2020. doi:10.1101/2020.07.29.20164251v1
8. Esteban O-P, katherine S-R, Ana Maria D, Alejandra B, Carla M, Vanessa A, et al. Epidemiological, socio-demographic and clinical features of the early phase of the COVID-19 epidemic in Ecuador. medRxiv. 2020. doi:10.1101/2020.05.08.20095943
9. Esteve A, Permanyer I, Boertien D, Vaupel JW. National age and coresidence patterns shape COVID-19 vulnerability. Proc Natl Acad Sci U S A. 2020. doi:https://dx.doi.org/10.1073/pnas.2008764117
10. Estrada García CB, Recio Fornaris I, Vega Torres R, Collejo Rosabal YM, Martínez Orozco D. Clinical epidemiological behavior of COVID-19. Granma, March-May 2020. Multimed (Granma). 2020;24: 870–886.
11. Evaldo Stanislau Affonso de A, Fatima Maria Bernardes Henriques A, Dongmin P, Ana Paola Ceraldi C, Murilo Augustinho Muniz da C, Evelyn Gutierrez K, et al. Teach, and teach and teach: does the average citizen use masks correctly during daily activities? Results from an observational study with more than 12,000 participants. medRxiv. 2020. doi:10.1101/2020.06.25.20139907
12. Evangelina Cervantes H, Pavel Roel Gutiérrez S. Resistir la Covid-19: intersecciones en la educación de Ciudad Juárez, México. Rev Int Educ para la Justicia Soc. 2020;9.
13. Ezequiel A, Franco M. COVID-19 mild cases determination from correlating COVID-line calls to reported cases. medRxiv. 2020. doi:10.1101/2020.07.26.20162008
14. Ezequiel A, Franco M, Nicolas K, Senastian C, Daniela O, Enio G. Estimating COVID-19 cases and outbreaks on-stream through phone-calls. medRxiv. 2020. doi:10.1101/2020.10.09.20210351
15. Fabian D, Alvaro J-M. Psychological distress during the COVID-19 epidemic in Chile: the role of economic uncertainty. medRxiv. 2020. doi:10.1101/2020.09.27.20202648
16. Fabiana G, Fabio Macedo M, Silvano Barbosa O, Victor Bertolo Gomes P, Wildo A, Helder N, et al. The impact of early social distancing at COVID-19 Outbreak in the largest Metropolitan Area of Brazil. medRxiv. 2020. doi:10.1101/2020.04.06.20055103
17. Fabio Rocha de P, Marcia Gomide da Silva M. Análise de Redes Sociais: a formação de grupos do Facebook frente à epidemia da COVID-19 no Brasil. 2020;32.
18. Fabio EL, Maria CM-C, Lewis FB, Silvia FC, Joao CSB, Sonia RPS, et al. A primary care approach to the COVID-19 pandemic: clinical features and natural history of 2,073 suspected cases in the Corona Sao Caetano programme, Sao Paulo, Brazil. medRxiv. 2020. doi:10.1101/2020.06.23.20138081
19. Faccini M, Ferruzzi F, Mori AA, Santin GC, Oliveira RC, Oliveira RCG, et al. Dental Care during COVID-19 Outbreak: A Web-Based Survey. Eur J Dent. 2020. doi:10.1055/s-0040-1715990
20. Faccini-Martinez AA, Rivero R, Garay E, Garcia A, Mattar S, Botero Y, et al. Serological cross-reactivity using a SARS-CoV-2 ELISA test in acute Zika virus infection, Colombia. Int J Infect Dis. 2020;101: 191–193. doi:http://dx.doi.org/10.1016/j.ijid.2020.09.1451
21. Faíco-Filho KS, Carvalho JMA, Conte DD, de Souza Luna LK, Bellei N. COVID-19 in health care workers in a university hospital during the quarantine in São Paulo city. Braz J Infect Dis. 2020;24: 462–465. doi:10.1016/j.bjid.2020.08.003
22. Faico-Filho KS, Passarelli VC, Bellei N. Is Higher Viral Load in SARS-CoV-2 Associated with Death? Am J Trop Med Hyg. 2020. doi:http://dx.doi.org/10.4269/ajtmh.20-0954
23. Faissal Nemer H, Miguel Morita F-S, Gustavo SP da C, Geny H, Ali H, Valderilio FA. Epidemiological and clinical characteristics of COVID-19 in Brazil using digital technology. medRxiv. 2020. doi:10.1101/2020.09.30.20204917
24. Falcón Hernández A, Navarro Machado VR, Díaz Brito A, Delgado Acosta HM, Valdés Gómez ML. Massive active population inquiry for COVID-19. Experience with medical science students. Cienfuegos, 2020. Medisur. 2020;18: 381–387.
25. Falcón Hernández A, Navarro Machado VR, Díaz Brito A, Delgado Acosta HM, Valdés Gómez ML. Pesquisa activa masiva poblacional para la COVID-19. Experiencia con estudiantes de las ciencias médicas. Cienfuegos, 2020. Medisur. 2020;18: 381–387.
26. Farias DLC, Prats J, Cavalcanti AB, Rosa RG, Machado FR, Berwanger O, et al. Rationale and design of the “Tocilizumab in patients with moderate to severe COVID-19: an open-label multicentre randomized controlled” trial (TOCIBRAS). Rev Bras Ter intensiva. 2020;32: 337–347. doi:http://dx.doi.org/10.5935/0103-507X.20200060
27. Federico Niccoló Daverio O, María M-A, Woo-Mora LG. Moral Force: Leaders’ Actions and Social Distancing. SSRN. 2020. doi:10.2139/ssrn.3678980
28. Felipe O, Daniel M, Alberto F, Ignacio D, Ignacia A, Maritza N, et al. Covid-19 in Chile. The experience of a Regional reference Center. Preliminary report. medRxiv. 2020. doi:10.1101/2020.06.14.20130898
29. Felipe S, Rugero B, Jacob M, Guillermo L-S, Igor G, Peter W, et al. Moderate to vigorous physical activity and sedentary behavior change in self-isolating adults during the COVID-19 pandemic in Brazil: A cross-sectional survey exploring correlates. medRxiv. 2020. doi:10.1101/2020.07.15.20154559
30. Felipe S, Aline V, Michel Jose A, Flavio Sanson F, Marco I, Mark S. A novel high specificity COVID-19 screening method based on simple blood exams and artificial intelligence. medRxiv. 2020. doi:10.1101/2020.04.10.20061036
31. Félix Lélis da S, Javier Dias P, Maryjane Diniz Araújo G, Andréa Pereira Lélis da S, Gabriel Lélis P da S. INTRAREGIONAL PROPAGATION OF COVID-19 CASES IN PARA, BRAZIL. ASSESSMENT OF ISOLATION REGIME TO LOCKDOWN. medRxiv. 2020. doi:10.1101/2020.06.10.20127886
32. Felix Lelis da S, Maryjane Diniz AG, Andrea Pereira Lelis da S, Samio Costa de S, Marcos Francisco Serafim de S, Gabriel Lelis P da S. Correlation between meteorological factors and COVID-19 infection in the Belem Metropolitan Region. medRxiv. 2020. doi:10.1101/2020.06.10.20127506
33. Fernanda Sumika Hojo S, Natália Satchiko H-S, Ben Dêivide de Oliveira B, Cristiano Maciel da S, Daniel Ludovico G. On the Analysis of Mortality Risk Factors for Hospitalized COVID-19 Patients: a Data-driven Study Using the Major Brazilian Database. medRxiv. 2020. doi:10.1101/2020.09.24.20200766
34. Fernanda Sumika Hojo S, Natália Satchiko H-S, Edimilson Batista S, Cristiano Maciel S, Daniel Ludovico G. Predicting the disease outcome in COVID-19 positive patients through Machine Learning: a retrospective cohort study with Brazilian data. medRxiv. 2020. doi:10.1101/2020.06.26.20140764
35. Fernanda M-P, Biani S. COVID-19 and (Un)Desired Fertility: The Effect of Stay-at-Home Orders on Abortions in Mexico City. SSRN. 2020. doi:10.2139/ssrn.3703581
36. Fernanda S, Livia K, Eugenia Z, Fernanda R, Rossana CS, Zelia MCL, et al. Gender, race and parenthood impact academic productivity during the COVID-19 pandemic: from survey to action. bioRxiv. 2020. doi:10.1101/2020.07.04.187583
37. Fernandes DE, Ferreira PRA, Kirsztajn GM. Pre-exposure prophylaxis during the SARS-CoV-2 pandemic: can PrEP prevent COVID-19-related symptoms? Epidemiol Infect. 2020. doi:http://dx.doi.org/10.1017/S0950268820002253

Fernandes D, Ferreira P, Kirsztajn G. Pre-Exposure Prophylaxis During COVID-19 Pandemic: Can PrEP Prevent Flu-Like Symptoms? ResearchSquare. 2020. doi:10.21203/rs.3.rs-27798/v1

1. Fernandes EG, Santos J da S, Sato HK. Outbreak investigation in cargo ship in times of COVID-19 crisis, Port of Santos, Brazil. Rev Saude Publica. 2020;54: 34. doi:https://dx.doi.org/10.11606/s1518-8787.2020054002461
2. Fernandes J, Silva R, Silva A, Villela D, Mendonça V, Lacerda A. Altitude conditions seem to determine the evolution of COVID-19 in Brazil: a significance relationship with cases and deaths. ResearchSquare. 2020. doi:10.21203/rs.3.rs-76633/v1
3. Fernandes L, Araújo F, Silva M. Insights into the dynamics of SARS-CoV-2 pandemicvia Shannon-Fisher causality plane. ResearchSquare. 2020. doi:10.21203/rs.3.rs-36581/v1
4. Fernandez RS, Crivelli L, Guimet NM, Allegri RF, Pedreira ME. Psychological distress associated with COVID-19 quarantine: Latent profile analysis, outcome prediction and mediation analysis. J Affect Disord. 2020;277: 75–84. doi:http://dx.doi.org/10.1016/j.jad.2020.07.133
5. Fernando Pérez F, Adrial Despaigne B, Ernesto Sánchez H, Odalis Cardona G, Jacno Erik Ferrer C. Variabilidad de los resultados de la PCR evolutiva en pacientes con la COVID-19. 2020;24.
6. Fernando Timoteo F, Tiago Almeida de O, Cristiane Esteves T, Andre Filipe de Moraes B, Gabriel Dalla C, Alexandre Chiavegatto F. A multipurpose machine learning approach to predict COVID-19 negative prognosis in Sao Paulo, Brazil. medRxiv. 2020. doi:10.1101/2020.08.26.20182584
7. Fernando AB, Leonardo SLB, Pedro K, Leila FD, Fernando GZ, Marcio S, et al. Characteristics and Outcomes of 4,942 Critically Ill Adult Patients with COVID-19 in Brazil. SSRN. 2020. doi:10.2139/ssrn.3670662
8. Fernando R, Luis Gabriel P-L, Olga Lucía A-R, Diana Marcela M-R, Virginia Z. Differential Diagnosis between Dengue and COVID-19 Infection: Diagnosis Challenge in Tropical Regions. SSRN. 2020. doi:10.2139/ssrn.3590469
9. Fernando T, Adrian Ezequiel Y, Daniel L, Pablo L, Pedro B, Marcelo C, et al. Sooner than you think: a very early affective reaction to the COVID-19 pandemic and quarantine in Argentina. medRxiv. 2020. doi:10.1101/2020.07.31.20166272
10. Feroze N. Forecasting the patterns of COVID-19 and causal impacts of lockdown in top five affected countries using Bayesian Structural Time Series Models. Chaos Solitons Fractals. 2020;140: 110196. doi:10.1016/j.chaos.2020.110196
11. Ferraz LMR. Saúde e política na crise da Covid-19: apontamentos sobre a pandemia na imprensa brasileira. RECIIS (Online). 2020;14: 273–278.
12. Ferreira JC, Ho Y-L, Besen BAMP, Malbuisson LMS, Taniguchi LU, Mendes P V, et al. Characteristics and outcomes of patients with COVID-19 admitted to the ICU in a university hospital in Sao Paulo, Brazil - study protocol. Clinics (Sao Paulo). 2020;75: e2294. doi:https://dx.doi.org/10.6061/clinics/2020/e2294
13. Ferreira S. On the efficiency of restrictive sanitary barriers to contain the progress of COVID-19: A simple mathematical modeling. 2020.
14. Ferrer Castro JE, Sánchez Hernández E, Poulout Mendoza A, del Río Caballero G, Figueredo Sánchez D. Clinical and epidemiological characterization of patients confirmed with COVID-19 in Santiago de Cuba. Medisan. 2020;24: 473–485.
15. Ferrero F, Ossorio MF, Torres FA, Debaisi G. Impact of the COVID-19 pandemic in the paediatric emergency department attendances in Argentina. Arch Dis Child. 2020. doi:https://dx.doi.org/10.1136/archdischild-2020-319833
16. Fiehler J, Brouwer P, Díaz C, Hirsch JA, Kulcsar Z, Liebeskind D, et al. COVID-19 and neurointerventional service worldwide: a survey of the European Society of Minimally Invasive Neurological Therapy (ESMINT), the Society of NeuroInterventional Surgery (SNIS), the Sociedad Iberolatinoamericana de Neuroradiologia Diagnostica y. J Neurointerv Surg. 2020;12: 726–730. doi:10.1136/neurintsurg-2020-016349
17. Figueroa F, Figueroa D, Calvo-Mena R, Narvaez F, Medina N, Prieto J. Orthopedic surgery residents’ perception of online education in their programs during the COVID-19 pandemic: should it be maintained after the crisis? Acta Orthop. 2020; 1–4. doi:https://dx.doi.org/10.1080/17453674.2020.1776461
18. Fitzgibbon WE, Morgan JJ, Webb GF, Wu Y. ANALYSIS of A REACTION-DIFFUSION EPIDEMIC MODEL with ASYMPTOMATIC TRANSMISSION. J Biol Syst. 2020;28: 561–587. doi:http://dx.doi.org/10.1142/S0218339020500126
19. Flavio Codeco C, Raquel Martins L, Oswaldo GC, Claudia TC, Daniel V, Leonardo SB, et al. Assessing the potential impacts of COVID-19 in Brasil: Mobility, Morbidity and Impact to the Health System. medRxiv. 2020. doi:10.1101/2020.03.19.20039131

Flávio CC, Raquel ML, Oswaldo GC, Daniel V, Leonardo SB, Ana Pastore y P, et al. Assessing the Potential Impact of COVID-19 in Brazil: Mobility, Morbidity and the Burden on the Health Care System. SSRN. 2020. doi:10.2139/ssrn.3559609

1. Fonseca M, Beatriz E. The cuban radio against the COVID-19. A case study. 2020.
2. Forlenza O V, Stella F, HCFMUSP LIMPC. Impact of SARS-CoV-2 pandemic on mental health in the elderly: perspective from a psychogeriatric clinic at a tertiary hospital in Sao Paulo, Brazil. Int psychogeriatrics. 2020; 1–5. doi:https://dx.doi.org/10.1017/S1041610220001180
3. Fortaleza CMCB, Guimaraes RB, de Almeida GB, Pronunciate M, Ferreira CP. Taking the inner route: spatial and demographic factors affecting vulnerability to COVID-19 among 604 cities from inner Sao Paulo State, Brazil. Epidemiol Infect. 2020;148: e118. doi:https://dx.doi.org/10.1017/S095026882000134X
4. Fowler Z, Moeller E, Roa L, Castañeda-Alcántara ID, Uribe-Leitz T, Meara JG, et al. Projected impact of COVID-19 mitigation strategies on hospital services in the Mexico City Metropolitan Area. PLoS One. 2020;15: e0241954. doi:10.1371/journal.pone.0241954
5. Francis Ribeiro S, Daisy M-S, Douglas dos Santos S, Juliana Beust L, Gustavo Goncalves C, Luciano Santos Pinto G, et al. Physical Activity Decreases the Prevalence of COVID-19-associated Hospitalization: Brazil EXTRA Study. medRxiv. 2020. doi:10.1101/2020.10.14.20212704
6. Francisca Marli Rodrigues de A, Tarssio Brito B, Andrés H-F, Andrea U, Yu Ting L. Twitter in Brazil: Discourses on China in Times of Coronavirus. SSRN. 2020. doi:10.2139/ssrn.3608566
7. Francisco HCF, Juvenia BF. Instantaneous R calculation for COVID-19 epidemic in Brazil. medRxiv. 2020. doi:10.1101/2020.04.23.20077172
8. Franco AS, Henrique DM, Almeida LF de, Prado LM do, Gomes RVS, Silva RCL. Safety in the Rapid sequence intubation recommended in COVID-19: Experience report. rev Cuid (Bucaramanga 2010). 2020;11: e1260–e1260.
9. Franco-Munoz C, Alvarez-Diaz DA, Laiton-Donato K, Wiesner M, Escandon P, Usme-Ciro JA, et al. Substitutions in Spike and Nucleocapsid proteins of SARS-CoV-2 circulating in South America. Infect Genet Evol. 2020;85: 104557. doi:http://dx.doi.org/10.1016/j.meegid.2020.104557
10. Freire-Paspuel B, Vega-Mariño P, Velez A, Castillo P, Masaquiza C, Cedeño-Vega R, et al. “One health” inspired SARS-CoV-2 surveillance: The Galapagos Islands experience. One Heal (Amsterdam, Netherlands). 2020; 100185. doi:10.1016/j.onehlt.2020.100185
11. Freitas ARR, Medeiros NM, Frutuoso LCV, Beckedorff OA, Martin LMA de, Medeiros MM de, et al. Use of excess mortality associated with the COVID-19 epidemic as an epidemiological surveillance strategy - preliminary results of the evaluation of six Brazilian capitals. 2020. doi:10.1590/SciELOPreprints.442
12. Freitas VLS, Konstantyner TCRO, Mendes JF, Sepetauskas CSDN, Santos LBL. The correspondence between the structure of the terrestrial mobility network and the spreading of COVID-19 in Brazil. Cad Saude Publica. 2020;36: e00184820. doi:http://dx.doi.org/10.1590/0102-311X00184820

Vander LSF, Jeferson F, Catia SNS, Leonardo BLS. The correspondence between the structure of the terrestrial mobility network and the emergence of COVID-19 in Brazil. medRxiv. 2020. doi:10.1101/2020.05.17.20104612

1. Freitas-Jesus J V, Rodrigues L, Surita FG. The experience of women infected by the COVID-19 during pregnancy in Brazil: a qualitative study protocol. Reprod Health. 2020;17: 108. doi:10.1186/s12978-020-00958-z
2. Friedman J, Calderon-Villarreal A, Bojorquez I, Vera Hernandez C, Schriger DL, Tovar Hirashima E. Excess Out-of-Hospital Mortality and Declining Oxygen Saturation: The Sentinel Role of Emergency Medical Services Data in the COVID-19 Crisis in Tijuana, Mexico. Ann Emerg Med. 2020;76: 413–426. doi:http://dx.doi.org/10.1016/j.annemergmed.2020.07.035

Joseph F, Alheli C-V, Ietza B, Carlos Vera H, David S, Eva Tovar H. Excess Out-Of-Hospital Mortality and Declining Oxygen Saturation Documented by EMS During the COVID-19 Crisis in Tijuana, Mexico. medRxiv. 2020. doi:10.1101/2020.05.13.20098186

1. Friedman J, Calderon-Villarreal A, Bojorquez I, Vera Hernandez C, Schriger D, Tovar Hirashima E. Excess Out-Of-Hospital Mortality and Declining Oxygen Saturation: The Sentinel Role of EMS Data in the COVID-19 Crisis in Tijuana, Mexico. medRxiv  Prepr Serv Heal Sci. 2020. doi:https://dx.doi.org/10.1101/2020.05.13.20098186
2. Friedrich F, Ongaratto R, Scotta MC, Veras TN, Stein R, Lumertz MS, et al. Early Impact of social distancing in response to COVID-19 on hospitalizations for acute bronchiolitis in infants in Brazil. Clin Infect Dis. 2020. doi:http://dx.doi.org/10.1093/cid/ciaa1458
3. Furtado RHM, Berwanger O, Fonseca HA, Correa TD, Ferraz LR, Lapa MG, et al. Azithromycin in addition to standard of care versus standard of care alone in the treatment of patients admitted to the hospital with severe COVID-19 in Brazil (COALITION II): a randomised clinical trial. Lancet. 2020;396: 959–967. doi:http://dx.doi.org/10.1016/S0140-6736%2820%2931862-6
4. Gabriel Berg de A, Thomas V, Claudia F, Carlos Magno Castelo Branco F. Several countries in one: a mathematical modeling analysis for COVID-19 in inner Brazil. medRxiv. 2020. doi:10.1101/2020.04.23.20077438
5. Gaitan-Rossi P, Vilar-Compte M, Teruel G, Perez-Escamilla R. Food insecurity measurement and prevalence estimates during the COVID-19 pandemic in a repeated cross-sectional survey in Mexico. Public Health Nutr. 2020; 1–25. doi:http://dx.doi.org/10.1017/S1368980020004000
6. Galan-Rodas E, Tarazona-Fernandez A, Palacios-Celi M. Risk and death of doctors 100 days after the state of emergency by COVID-19 in Peru. Acta Medica Peru. 2020;37: 119–121. doi:http://dx.doi.org/10.35663/amp.2020.372.1033
7. Galhardi CP, Freire NP, Minayo MCS, Fagundes MCM. Fact or Fake? An analysis of disinformation regarding the Covid-19 pandemic in Brazil. Cien Saude Colet. 2020;25: 4201–4210. doi:http://dx.doi.org/10.1590/1413-812320202510.2.28922020
8. Galván-Tejada CE, Zanella-Calzada LA, Villagrana-Bañuelos KE, Moreno-Báez A, Luna-García H, Celaya-Padilla JM, et al. Demographic and Comorbidities Data Description of Population in Mexico with SARS-CoV-2 Infected Patients(COVID19): An Online Tool Analysis. Int J Environ Res Public Health. 2020;17. doi:10.3390/ijerph17145173
9. Garces TS, Florencio RS, Cestari VRF, Pereira MLD, Moreira TMM, Bezerra Sousa GJ. COVID-19 in a state of Brazilian Northeast: prevalence and associated factors in people with flu-like syndrome. J Clin Nurs. 2020. doi:http://dx.doi.org/10.1111/jocn.15472
10. Garces-Ayala F, Araiza-Rodriguez A, Mendieta-Condado E, Rodriguez-Maldonado AP, Wong-Arambula C, Landa-Flores M, et al. Full genome sequence of the first SARS-CoV-2 detected in Mexico. Arch Virol. 2020. doi:https://dx.doi.org/10.1007/s00705-020-04695-3
11. Garcez FB, Aliberti MJR, Poco PCE, Hiratsuka M, de Fatima Takahashi S, Coelho VA, et al. Delirium and adverse outcomes in hospitalized patients with COVID-19. J Am Geriatr Soc. 2020. doi:10.1111/jgs.16803
12. Garcia de Avila MA, Hamamoto Filho PT, Jacob F, Alcantara LRS, Berghammer M, Jenholt Nolbris M, et al. Children’s Anxiety and Factors Related to the COVID-19 Pandemic: An Exploratory Study Using the Children’s Anxiety Questionnaire and the Numerical Rating Scale. Int J Environ Res Public Health. 2020;17: 1–13. doi:10.3390/ijerph17165757
13. Garcia Filho C. Simulating social distancing measures in household and close contact transmission of SARS-CoV-2. Cad Saude Publica. 2020;36: e00099920. doi:https://dx.doi.org/10.1590/0102-311X00099920
14. Garcia Filho C, Vieira LJE de S, Silva RM da. Internet searches for measures to address COVID-19 in Brazil: a description of searches in the first 100 days of 2020. Epidemiol e Serv saude  Rev do Sist Unico Saude do Bras. 2020;29: e2020191. doi:https://dx.doi.org/10.5123/S1679-49742020000300011
15. García LY, Cerda AA. Contingent assessment of the COVID-19 vaccine. Vaccine. 2020;38: 5424–5429. doi:10.1016/j.vaccine.2020.06.068
16. Garcia LP, Traebert J, Boing AC, Santos GFZ, Pedebôs LA, Orsi E, et al. The potential propagation of Covid-19 and government decision-making: a retrospective analysis in Florianópolis, Brazil. 2020.
17. Garcia-Huidobro D, Rivera S, Chang S V, Bravo P, Capurro D. System-wide accelerated implementation of telemedicine in response to COVID-19: Mixed methods evaluation. J Med Internet Res. 2020;22: e22146. doi:http://dx.doi.org/10.2196/22146
18. Garcia-Huidobro D, Rivera S, Valderrama S, Bravo P, Capurro D. System-wide Accelerated Implementation of Telemedicine in Response to COVID-19: A Mixed-Methods Evaluation. J Med Internet Res. 2020;22: e22146. doi:10.2196/22146
19. García-Reyna B, Castillo-García GD, Barbosa-Camacho FJ, Cervantes-Cardona GA, Cervantes-Pérez E, Torres-Mendoza BM, et al. Fear of covid-19 scale for hospital staff in regional hospitals in mexico: A brief report. Int J Ment Health Addict. 2020; 1–12. doi:10.1007/s11469-020-00413-x

García-Reyna B, Castillo-García G, Barbosa-Camacho F, Cervantes-Cardona G, Cervantes-Pérez E, Torres-Mendoza B, et al. Fear of COVID-19 Scale for Hospital Staff in Regional Hospitals in Mexico: A Survey Study. ResearchSquare. 2020. doi:10.21203/rs.3.rs-34065/v1

1. García-Salirrosas EE, Sánchez-Poma RA. PREVALENCIA DE LOS TRASTORNOS MUSCULOESQUELÉTICO EN DOCENTES UNIVERSITARIOS QUE REALIZAN TELETRABAJO EN TIEMPOS DE COVID-19. 2020.
2. Gaspar GDS, Figueiredo N, de Lucena EHG, Ceissler CAS, Cavalcanti RP, de Goes PSA. Characterization of dental surgeons of pernambuco state in the covid-19 pandemic context: Preliminary data. Pesqui Bras Odontopediatria Clin Integr. 2020;20: 1–7. doi:http://dx.doi.org/10.1590/pboci.2020.152

Gaspar G da S, Figueiredo N, Lucena EHG de, Ceissler CAS, Cavalcanti RP, Goes PSA de. Characterization of dental surgeons of Pernambuco in the COVID-19 pandemic context: preliminary data. 2020.

1. Gaston V-H, Andres N. Extension and implementation of a system modelling the COVID-19 pandemic in Chile. medRxiv. 2020. doi:10.1101/2020.06.21.20136606
2. Genny C, Nina Mendez D, Kassandra DSZ, Andrea Rochel P, Mario Azuela M, Osman Cuevas K, et al. Clinical course and severity outcome indicators among COVID 19 hospitalized patients in relation to comorbidities distribution Mexican cohort. medRxiv. 2020. doi:10.1101/2020.07.31.20165480
3. Geremia DS, Vendruscolo C, Celuppi IC, Adamy EK, Toso BRG de O, Souza JB de. 200 Years of Florence and the challenges of nursing practices management in the COVID-19 pandemic. Rev Lat Am Enfermagem. 2020;28: e3358. doi:https://dx.doi.org/10.1590/1518-8345.4576.3358
4. Geremia DS, Vendruscolo C, Celuppi IC, Souza JB de, Schopf K, Maestri E. Pandemia COVID-2019: formação e atuação da enfermagem para o Sistema Único de Saúde. Enferm foco. 2020;11: 40–47.
5. Gerson I, Graziela A, Cristina Berger F. Nonspecific blood tests as proxies for COVID-19 hospitalization: are there plausible associations after excluding noisy predictors? medRxiv. 2020. doi:10.1101/2020.10.12.20211557
6. Giannouchos T V, Sussman RA, Mier JM, Poulas K, Farsalinos K. Characteristics and risk factors for COVID-19 diagnosis and adverse outcomes in Mexico: an analysis of 89,756 laboratory-confirmed COVID-19 cases. Eur Respir J. 2020. doi:10.1183/13993003.02144-2020
7. Giardino DL, Huck-Iriart C, Riddick M, Garay A. The endless quarantine: the impact of the COVID-19 outbreak on healthcare workers after three months of mandatory social isolation in Argentina. Sleep Med. 2020;76: 16–25. doi:http://dx.doi.org/10.1016/j.sleep.2020.09.022
8. Giordano Perez G, Rosalino Flores R, Julio Cesar Valadez V, Melissa Hernandez A, Gilberto Herrera M, Miguel Alejandro Del Real L. Clinical and epidemiological characteristics of children with SARS-CoV-2 infection: case series in Sinaloa. medRxiv. 2020. doi:10.1101/2020.07.07.20146332
9. Gislaine F, Patricia Hermes S, Doris Sobral Marques S, Edmundo Carlos G, Maria Elisa M, Paula R, et al. SARS-CoV-2 in human sewage in Santa Catalina, Brazil, November 2019. medRxiv. 2020. doi:10.1101/2020.06.26.20140731
10. Gláucio de Castro J, Luciana de Souza A, Helena Carla C, Gildete A, Alex Sandro Lins R. Coronavirus, Deafness and the Use of Different Signs of the Area in Health during a Period of Pandemic Time: Is That the Best Option to Do? Creat Educ. 2020;11. doi:10.4236/CE.2020.114042
11. Glenn FW, William EF, Jeffrey JM, Yixiang W. Predicting the end-stage of the COVID-19 epidemic in Brazil. medRxiv. 2020. doi:10.1101/2020.05.28.20116103
12. Goes FGB, Silva ACSS da, Santos AST Dos, Pereira-Avila FMV, Silva LJ da, Silva LF da, et al. Challenges faced by pediatric nursing workers in the face of the COVID-19 pandemic. Rev Lat Am Enfermagem. 2020;28: e3367. doi:https://dx.doi.org/10.1590/1518-8345.4550.3367
13. Goessler KF, Nicoletti CF, Rezende DAN, Sieczkowska SM, Esteves GP, Genario R, et al. Outpatient Screening of Health Status Among Postbariatric Patients during the COVID-19 Pandemic in Sao Paulo, Brazil. Obesity. 2020. doi:http://dx.doi.org/10.1002/oby.23030

Karla Fabiana G, Carolina Ferreira N, Diego Augusto Nunes R, Sofia Mendes S, Gabriel Perri E, Rafael G, et al. Outpatient screening of health status and lifestyle among post-bariatric patients during the Covid-19 pandemic in Sao Paulo, Brazil. medRxiv. 2020. doi:10.1101/2020.07.30.20165068

1. Goiás . Secretaria da S. Comorbidade, idade e letalidade no COVID-19 no estado de Goiás. 2020.
2. Gois AN, Laureano EE, Santos DDS, Sanchez DE, Souza LF, Vieira RCA, et al. Lockdown as an Intervention Measure to Mitigate the Spread of COVID-19: a modeling study. Rev Soc Bras Med Trop. 2020;53: e20200417. doi:http://dx.doi.org/10.1590/0037-8682-0417-2020
3. Gomes CM, Favorito LA, Henriques JVT, Canalini AF, Anzolch KMJ, de Carvalho Fernandes R, et al. Impact of COVID-19 on clinical practice, income, health and lifestyle behavior of Brazilian urologists. Int Braz J Urol. 2020;46. doi:https://dx.doi.org/10.1590/S1677-5538.IBJU.2020.99.15
4. Gomes DS, Andrade LA, Ribeiro CJN, Peixoto MVS, Lima SVMA, Duque AM, et al. Risk clusters of COVID-19 transmission in Northeastern Brazil: Prospective space-time modeling. Epidemiol Infect. 2020. doi:http://dx.doi.org/10.1017/S0950268820001843
5. Gomez-Cravioto D, Diaz-Ramos R, Cantu-Ortiz F, Ceballos H. An Exploration and Forecast of COVID-19 in Mexico with Machine Learning. ResearchSquare. 2020. doi:10.21203/rs.3.rs-62035/v1
6. Gomides A, Ferreira G, Kakehasi A, Lacerda M, Marques C, Mota L, et al. IMPACT OF CHRONIC USE OF ANTIMALARIALS ON SARS-CoV-2 INFECTION IN PATIENTS WITH IMMUNE-MEDIATED RHEUMATIC DISEASES: PROTOCOL DESIGN FOR A MULTICENTRIC OBSERVATIONAL COHORT IN BRAZIL. JMIR Res Protoc. 2020. doi:10.2196/23532
7. Goncalves AP, Zuanazzi AC, Salvador AP, Jaloto A, Pianowski G, Carvalho LDF. Preliminary findings on the associations between mental health indicators and social isolation during the COVID-19 pandemic. Arch Psychiatry Psychother. 2020;22: 10–19. doi:http://dx.doi.org/10.12740/APP/122576
8. Gondim JAM, Machado L. Optimal quarantine strategies for the COVID-19 pandemic in a population with a discrete age structure. Chaos Solitons Fractals. 2020;140: 110166. doi:10.1016/j.chaos.2020.110166
9. Gonzales-Castillo JR, Varona-Castillo L, Domínguez-Morante MG, Ocaña-Gutierrez VR. COVID-19 pandemic and Public Health Policies in Peru: March-May 2020. Rev salud pública. 2020;22: e387373–e387373.
10. Gonzales-Luna AC, Torres-Valencia JO, Alarcon-Santos JE, Segura-Saldana PA. Impact of COVID-19 on pacemaker implant. J Arrhythmia. 2020;36: 845–848. doi:http://dx.doi.org/10.1002/joa3.12411
11. González Espangler L, Lafargue Gainza F, Borges Toirac MA, Romero García LI. Care to patients with oral problems during COVID-19: a challenge to the professionals of the health sector. Medisan. 2020;24: 593–609.
12. González Ramírez LP, Martínez Arriaga RJ, Hernández-Gonzalez MA, De la Roca-Chiapas JM. Psychological Distress and Signs of Post-Traumatic Stress in Response to the COVID-19 Health Emergency in a Mexican Sample. Psychol Res Behav Manag. 2020;13: 589–597. doi:10.2147/PRBM.S259563
13. Gonzalez M, Ameri L, Muñoz L, Luzuriaga JP, Pifano M, Velázquez V, et al. COVID-19 AND SOCIAL VULNERABILITY: DESCRIPTIVE STUDY ABOUT A CASE SERIE IN THE METROPOLITAN AREA OF BUENOS AIRES*. 2020.
14. González R, M M, Estupiñán-López C, Morales C. An adapted ODE model to study the Dynamics of SARS-Cov-2 Infection (COVID-19): different scenarios for Brazil and other countries. ResearchSquare. 2020. doi:10.21203/rs.3.rs-29563/v1
15. Gonzalez SE, Regairaz L, Ferrando NS, Martinez VVG, Salazar MR, Estenssoro E. Convalescent plasma therapy in covid-19 patients, in the province of Buenos Aires. Med. 2020;80: 417–424.
16. Gonzalez SMP, Tartaglione F, Babio GRD, Schiavone MJ, Gelpi FC, Angrisani MA, et al. Risks of cardiovascular disease patients in times of covid-19. A survey of the argentine foundation of cardiology. Rev Argent Cardiol. 2020;88: 212–216. doi:10.7775/rac.v88.i3.18019
17. González-Bustamante B. Evolution and early government responses to COVID-19 in South America. World Dev. 2021;137: 105180. doi:10.1016/j.worlddev.2020.105180
18. Gonzalez-Calatayud DM, Vargas-Abrego DB, Gutierrez-Uvalle DGE, Lopez-Romero DSC, Gonzalez-Perez DLG, Carranco-Martinez DJA, et al. Observational study of the suspected or confirmed cases of sars COV-2 infection needing emergency surgical intervention during the first months of the pandemic in a third level hospital: Case series. Ann Med Surg. 2020;60: 149–154. doi:http://dx.doi.org/10.1016/j.amsu.2020.10.038
19. Gonzalez-Dambrauskas S, Vasquez-Hoyos P, Camporesi A, Diaz-Rubio F, Pineres-Olave BE, Fernandez-Sarmiento J, et al. Pediatric Critical Care and COVID-19. Pediatrics. 2020;146. doi:https://dx.doi.org/10.1542/peds.2020-1766
20. González-Jaramillo V, González-Jaramillo N, Gómez-Restrepo C, Palacio-Acosta CA, Gómez-López A, Franco OH. Impact of the COVID-19 pandemic on the Colombian population according to mitigation measures: preliminary data from epidemiological models for the period March 18 to April 18, 2020. Rev salud pública. 2020;22: e185989–e185989.
21. González-Seguel F, Adasme RS, Henríquez LI, Sufán JL, Merino- Osorio C. Modificaciones en las prácticas de los kinesiólogos de cuidados Críticos durante y después de la Pandemia COVID-19 en Chile: protocolo de una Encuesta Nacional. Kinesiologia. 2020;39: 2–7.
22. González-Seguel F, Adasme RS, Henríquez LI, Sufán JL, Merino- Osorio C. Modifications in Critical Care Physiotherapists Practices during and after COVID-19 Pandemic in Chile: Study Protocol of a National Survey. Kinesiologia. 2020;39: 2–7.
23. Gopaul C, Ventour D, Thomas D. Laboratory Predictors for COVID-19 ICU Admissions in a Caribbean Territory. ResearchSquare. 2020. doi:10.21203/rs.3.rs-103394/v1
24. Goularte JF, Serafim SD, Colombo R, Hogg B, Caldieraro MA, Rosa AR. COVID-19 and mental health in Brazil: Psychiatric symptoms in the general population. J Psychiatr Res. 2021;132: 32–37. doi:http://dx.doi.org/10.1016/j.jpsychires.2020.09.021
25. Gouveia AO de, Silva HRDS, Batista Neto JBDS. Mental health in times of COVID-19: construction of educational booklet with guidelines for the pandemic period. Enferm foco. 2020;11: 168–173.
26. Gouveia GP de M, Gouveia SSV, Vasconcelos SS, Silva E de SM, Medeiros RG, Silva RL da. Historical series of cases of COVID-19 in brazilian regions in the year of 2020. J Heal Biol Sci. 2020;8: 1–7.
27. Grando RD, Brentano VB, Zanardo AP, Hertz FT, Júnior LCA, Prietto Dos Santos JF, et al. Clinical usefulness of tomographic standards for COVID-19 pneumonia diagnosis: Experience from a Brazilian reference center. Braz J Infect Dis. 2020. doi:10.1016/j.bjid.2020.10.002
28. Gravas S, Bolton D, Gomez R, Klotz L, Kulkarni S, Tanguay S, et al. Impact of COVID-19 on Urology Practice: A Global Perspective and Snapshot Analysis. J Clin Med. 2020;9. doi:https://dx.doi.org/10.3390/jcm9061730
29. Grebe G, Velez JA, Tiutiunnyk A, Aragon-Caqueo D, Fernandez-Salinas J, Navarrete M, et al. Dynamic Quarantine: A comparative analysis of the Chilean Public Health response to COVID-19. Epidemiol Infect. 2020; 1–26. doi:http://dx.doi.org/10.1017/S0950268820002678
30. Grillo-Rojas PF, Romero-Onofre R. Estimate of the excess of deaths reported compared to the report of deaths from covid-19 in peru from march to may 2020. Rev la Fac Med Humana. 2020;20: 646–650. doi:http://dx.doi.org/10.25176/RFMH.V20I4.3220
31. Grillo-Rojas PF, Romero-Onofre R, Aldana-Carrasco J. Comparison of early non-pharmacological complications in covid-19 mortality from Peru and the United States of america. Rev la Fac Med Humana. 2020;20: 425–432. doi:http://dx.doi.org/10.25176/RFMH.v20i3.3114
32. Grotto RMT, Santos Lima R, de Almeida GB, Ferreira CP, Guimaraes RB, Pronunciate M, et al. Increasing molecular diagnostic capacity and COVID-19 incidence in Brazil. Epidemiol Infect. 2020;148: e178. doi:https://dx.doi.org/10.1017/S0950268820001818
33. Guadalupe Espitia-Hernandez LMDD-CRL-EFJ-P. Effects of Ivermectin-azithromycin-cholecalciferol combined therapy on COVID-19 infected patients: A proof of concept study. Biomed Res. 2020;31: 129–133.
34. Guenther F, Nina O-F, Tobias S, Sandra G, Ana Paula F, Claudia D, et al. Inactivated trivalent influenza vaccine is associated with lower mortality among Covid-19 patients in Brazil. medRxiv. 2020. doi:10.1101/2020.06.29.20142505
35. Guerrero R, Parra V, Cahua J, Holl J. Self-Reported Psychological Problems and Coping Strategies: A Web-Based Study in Peruvian Population During the COVID-19 Pandemic. ResearchSquare. 2020. doi:10.21203/rs.3.rs-42462/v1
36. Guerrero-Latorre L, Ballesteros I, Villacrés-Granda I, Granda MG, Freire-Paspuel B, Ríos-Touma B. SARS-CoV-2 in river water: Implications in low sanitation countries. Sci Total Environ. 2020;743: 140832. doi:10.1016/j.scitotenv.2020.140832

Laura G-L, Isabel B, Irina V, Maria Genoveva G-A, Byron F, Blanca R-T. First SARS-CoV-2 detection in river water: implications in low sanitation countries. medRxiv. 2020. doi:10.1101/2020.06.14.20131201

1. Guerrero-Nancuante C, Manriquez P R. [An epidemiological forecast of COVID-19 in Chile based on the generalized SEIR model and the concept of recovered]. Medwave. 2020;20: e7898. doi:https://dx.doi.org/10.5867/medwave.2020.04.7898
2. Guerrero-Torres L, Caro-Vega Y, Crabtree-Ramirez B, Sierra-Madero JG. Clinical Characteristics and Mortality of Healthcare Workers with SARS-CoV-2 infection in Mexico City. Clin Infect Dis. 2020. doi:http://dx.doi.org/10.1093/cid/ciaa1465
3. Guilherme Pertinni de Morais G, Samara Sousa Vasconcelos G, Samila Sousa V, Elisson de Sousa Mesquita S, Rebeca Galdino M, Rosangela Lago da S. INCIDÊNCIA E LETALIDADE DA COVID-19 NO CEARÁ, 2020. 2020;14.
4. Guilherme SC, Wesley  Sr. C, Silvio CF. Metapopulation modeling of COVID-19 advancing into the countryside: an analysis of mitigation strategies for Brazil. medRxiv. 2020. doi:10.1101/2020.05.06.20093492
5. Guillermo Alberto Pérez F. ¿Es la comorbilidad cardiovascular la causante de la elevación de la proteína C reactiva en pacientes positivos a la COVID-19? 2020;14.
6. Guimaraes RM, Magalhaes M de AFM, Xavier DR, Saldanha R de F, Catao R de C. Is it time to talk about the end of social distancing? A joinpoint analysis of COVID-19 time series in Brazilian capitals. Rev Soc Bras Med Trop. 2020;53: e20200469. doi:https://dx.doi.org/10.1590/0037-8682-0469-2020
7. Guiroy A, Gagliardi M, Coombes N, Landriel F, Zanardi C, Camino Willhuber G, et al. COVID-19 Impact Among Spine Surgeons in Latin America. Glob Spine J. 2020. doi:http://dx.doi.org/10.1177/2192568220928032
8. Guiroy A, Gagliardi M, Coombes N, Landriel F, Zanardi C, Camino Willhuber G, et al. Psychosocial impact of the COVID-19 pandemic on Argentine spinal surgeons. Rev argent neurocir. 2020;34: 187–193.
9. Gustavo Lugo G, Carmen H-C, Carlos T-S, Felipe J, Hector S, Cristina A, et al. Clinical characteristics and outcomes of patients with COVID-19 and ARDS admitted to a third level health institution in Mexico City. medRxiv. 2020. doi:10.1101/2020.09.12.20193409
10. Gustavo C, Maria-Isabel L, Mauricio G-N, Claudia Alvarado De La B, Santiago A-R, amy peralta-prado, et al. Acute kidney injury in patients with severe COVID-19 in Mexico. medRxiv. 2020. doi:10.1101/2020.08.28.20167379
11. Gutiérrez ED, Puche R, Hernández F. Estimation of COVID-19 cases in South American countries using ARIMA models. Obs del Conoc Rev Espec en Gestión Soc del Conoc. 2020;5: 11–25.
12. Haider N, Yavlinsky A, Simons D, Osman AY, Ntoumi F, Zumla A, et al. Passengers’ destinations from China: low risk of Novel Coronavirus (2019-nCoV) transmission into Africa and South America. Epidemiol Infect. 2020;148: e41. doi:https://dx.doi.org/10.1017/S0950268820000424
13. Hallal PC, Hartwig FP, Horta BL, Silveira MF, Struchiner CJ, Vidaletti LP, et al. SARS-CoV-2 antibody prevalence in Brazil: results from two successive nationwide serological household surveys. Lancet Glob Heal. 2020;8: e1390–e1398. doi:http://dx.doi.org/10.1016/S2214-109X%2820%2930387-9
14. Hallal PC, Barros FC, Silveira MF, Barros AJD de, Dellagostin OA, Pellanda LC, et al. EPICOVID19 protocol: repeated serological surveys on SARS-CoV-2 antibodies in Brazil. Cien Saude Colet. 2020;25: 3573–3578. doi:https://dx.doi.org/10.1590/1413-81232020259.25532020
15. Hallal PC, Horta BL, Barros AJD, Dellagostin OA, Hartwig FP, Pellanda LC, et al. Trends in the prevalence of COVID-19 infection in Rio Grande do Sul, Brazil: repeated serological surveys. Cien Saude Colet. 2020;25: 2395–2401. doi:https://dx.doi.org/10.1590/1413-81232020256.1.09632020
16. Heberto AB, Carlos PCJ, Antonio CRJ, Patricia PP, Enrique TR, Danira MPJ, et al. Implications of myocardial injury in Mexican hospitalized patients with coronavirus disease 2019 (COVID-19). IJC Hear Vasc. 2020;30: 100638. doi:http://dx.doi.org/10.1016/j.ijcha.2020.100638
17. Hector Eduardo C, Roberto Raul H, Maria Eugenia F. Safety and Efficacy of the combined use of ivermectin, dexamethasone, enoxaparin and aspirin against COVID-19. medRxiv. 2020. doi:10.1101/2020.09.10.20191619
18. Helcio RBO, Marcelo C, George SD, Luiz FSF. Estimation Of State Variables And Model Parameters For The Evolution Of COVID-19 In The City Of Rio de Janeiro. medRxiv. 2020. doi:10.1101/2020.05.21.20108407
19. Henrique Matheus S, Rafael Silva P, Raquel de Abreu G, Adolfo S, Fabio Andre P. Projection of hospitalization by COVID-19 in Brazilfollowing different social distances policies. medRxiv. 2020. doi:10.1101/2020.04.26.20080143
20. Hernández-Flores ML, Escobar-Sánchez J, Paredes-Zarco JE, Franyuti Kelly GA, Carranza-Ramírez L. Prediction and Potential Spatially Explicit Spread of COVID-19 in Mexico’s Megacity North Periphery. Healthc (Basel, Switzerland). 2020;8. doi:10.3390/healthcare8040453
21. Hernández-Galdamez DR, González-Block MÁ, Romo-Dueñas DK, Lima-Morales R, Hernández-Vicente IA, Lumbreras-Guzmán M, et al. Increased Risk of Hospitalization and Death in Patients with COVID-19 and Pre-existing Noncommunicable Diseases and Modifiable Risk Factors in Mexico. Arch Med Res. 2020. doi:10.1016/j.arcmed.2020.07.003
22. Hernández-García I, Giménez-Júlvez T. Characteristics of YouTube Videos in Spanish on How to Prevent COVID-19. Int J Environ Res Public Health. 2020;17: 1–10. doi:10.3390/ijerph17134671
23. Hernandez-Garduno E. Comorbidities that predict acute respiratory syndrome coronavirus 2 test positivity in Mexican Children: A case-control study. Pediatr Obes. 2020. doi:http://dx.doi.org/10.1111/ijpo.12740
24. Hernandez-Garduno E. Obesity is the comorbidity more strongly associated for Covid-19 in Mexico. A case-control study. Obes Res Clin Pract. 2020. doi:https://dx.doi.org/10.1016/j.orcp.2020.06.001
25. Hernandez-Huerta MT, Perez-Campos Mayoral L, Romero Diaz C, Martinez Cruz M, Mayoral-Andrade G, Sanchez Navarro LM, et al. Analysis of SARS-CoV-2 mutations in Mexico, Belize, and isolated regions of Guatemala and its implication in the diagnosis. J Med Virol. 2020. doi:http://dx.doi.org/10.1002/jmv.26591
26. Hernández-Sánchez BR, Cardella GM, Sánchez-García JC. Psychological Factors that Lessen the Impact of COVID-19 on the Self-Employment Intention of Business Administration and Economics’ Students from Latin America. Int J Environ Res Public Health. 2020;17: 1–22. doi:10.3390/ijerph17155293
27. Hernández-Vásquez A, Azañedo D, Vargas-Fernández R, Bendezu-Quispe G. Association of Comorbidities With Pneumonia and Death Among COVID-19 Patients in Mexico: A Nationwide Cross-sectional Study. J Prev Med Public Health. 2020;53: 211–219. doi:10.3961/jpmph.20.186
28. Hernandez-Vasquez A, Gamboa-Unsihuay JE, Vargas-Fernandez R, Azanedo D. Excess mortality in Metropolitan Lima during the COVID-19 pandemic: A district level comparison. Medwave. 2020;20: e8031. doi:http://dx.doi.org/10.5867/medwave.2020.08.8032
29. Hernandez-Vasquez A, Vargas-Fernandez R, Bendezu-Quispe G. Association between hypertension and pneumonia caused by SARS-CoV-2 in Mexican population. J Hypertens. 2020;38: 1857–1858. doi:http://dx.doi.org/10.1097/HJH.0000000000002544
30. Herrera-Valenzuela T, Narrea Vargas JJ, Merlo R, Valdés-Badilla PA, Pardo-Tamayo C, Franchini E. Effect of the COVID-19 quarantine on body weight among combat sports athletes. Nutr Hosp. 2020. doi:10.20960/nh.03207
31. Hildegart A, Santos E, Fernando HN. COVID-19 with Uncertain Phases: Estimation Issues with An Illustration for Argentina. SSRN. 2020. doi:10.2139/ssrn.3633500
32. Hillesheim D, Tomasi YT, Figueiró TH, Paiva KM de. Síndrome respiratória aguda grave por COVID-19 em crianças e adolescentes no Brasil: perfil dos óbitos e letalidade hospitalar até a 38a Semana Epidemiológica de 2020. Epidemiol e Serviços Saúde. 2020;29. doi:10.1590/s1679-49742020000500021
33. Horta BL, Silveira MF, Barros AJD, Barros FC, Hartwig FP, Dias MS, et al. Prevalence of antibodies against SARS-CoV-2 according to socioeconomic and ethnic status in a nationwide Brazilian survey. Rev Panam Salud Publica. 2020;44: e135. doi:10.26633/RPSP.2020.135
34. Huamanchumo-Suyon ME, Urrunaga-Pastor D, Ruiz-Perez PJ, Rodrigo-Gallardo PK, Toro-Huamanchumo CJ. Impact of the COVID-19 pandemic on general surgery residency program in Peru: A cross-sectional study. Ann Med Surg. 2020;60: 130–134. doi:http://dx.doi.org/10.1016/j.amsu.2020.10.031
35. Huamani C, Huamani C, Timana-Ruiz R, Pinedo J, Pinedo J, Perez J, et al. Estimated conditions to control the COVID-19 pandemic in pre- and postquarantine scenarios in Peru. Rev peru med exp salud publica. 2020;37: 195–202. doi:10.17843/RPMESP.2020.372.5405
36. Huerta Saenz IH, Elias Estrada JC, Del Castillo KC, Taya RM, Coronado JC. Maternal and perinatal characteristics of pregnant women with COVID-19 in a national hospital in Lima, Peru. Rev Peru Ginecol y Obstet. 2020;66: 19–24. doi:http://dx.doi.org/10.31403/rpgo.v66i2245
37. Hurtado-Ortiz A, Moreno-Montoya J, Prieto-Alvarado FE, Idrovo Á J. Benchmarking of public health surveillance of COVID-19 in Colombia: First semester. Biomedica. 2020;40: 131–138. doi:10.7705/biomedica.5812
38. Hyun Mo Y, Luis Pedro Lombardi J, Ariana Campos Y. The SIR model estimates incorrectly the basic reproduction number for the covid-19 epidemic. medRxiv. 2020. doi:10.1101/2020.10.11.20210831
39. Hyun Mo Y, Luis Pedro Pedro Lombardi J, Ariana Campos Y, Fabio Fernandes Morato C. Evaluating the impacts of release in Sao Paulo State (Brazil) on the epidemic of covid-19 based on mathematical model. medRxiv. 2020. doi:10.1101/2020.08.03.20167221
40. Hyun Mo Y, Luis Pedro Pedro Lombardi J, Fábio Fernandes Morato C, Ariana Campos Y. Evaluating epidemiological scenarios of isolation and further releases considering protection actions to control transmission of CoViD-19 in Sao Paulo State, Brazil. medRxiv. 2020. doi:10.1101/2020.05.19.20099309
41. Idrovo Á J, Moreno-Montoya J, Pinzón-Flórez CE. Performance of rapid IgM-IgG combined antibody tests in the occupational surveillance of COVID-19 in Colombian enterprises. Biomedica. 2020;40: 139–147. doi:10.7705/biomedica.5829
42. Idrovo AJ, Manrique-Hernandez EF, Fernandez Nino JA. Report From Bolsonaro’s Brazil: The Consequences of Ignoring Science. Int J Health Serv. 2020; 20731420968446. doi:http://dx.doi.org/10.1177/0020731420968446
43. Ietza B, Cesar I, Isabel V, Silvana L, Chiara S. MIGRANTS IN TRANSIT AND ASYLUM SEEKERS IN MEXICO: AN EPIDEMIOLOGICAL ANALYSIS OF THE COVID-19 PANDEMIC. medRxiv. 2020. doi:10.1101/2020.05.08.20095604
44. Igor Gadelha P, Joris MG, Andouglas Goncalves  Jr. S, Cosimo D, Gabriel Santos G, Luiz MGG. Forecasting Covid-19 dynamics in Brazil: a data driven approach. medRxiv. 2020. doi:10.1101/2020.05.11.20098392
45. Igor Hermann Huerta S, Jose Carlos Elias E, Katherine Campos Del C, Rossana Muñoz T, Julia Cristina C. Características materno perinatales de gestantes COVID-19 en un hospital nacional de Lima, Perú. Rev Peru Ginecol y Obstet. 2020;66. doi:10.31403/RPGO.V66I2245
46. Igor Silva C, Vinicius Ferreira A, Karina Baltor C, Jean Ezequiel L, Stefan Vilges de O. A vulnerability analysis for the management of and response to the COVID-19 epidemic in the second most populous state in Brazil. medRxiv. 2020. doi:10.1101/2020.07.20.20158345
47. Iragorri N, Gomez-Restrepo C, Barrett K, Herrera S, Hurtado I, Khan Y, et al. Covid-19: Adaptation of a model to predict healthcare resource needs in valle del Cauca, Colombia. Colomb Med. 2020;51: 1–12. doi:http://dx.doi.org/10.25100/cm.v51i3.e4534
48. Iraldo B, Hernandez-Bernal F, Nodarse-Cuni H, Duncan-Roberts Y, Suarez C, Campa-Legrá I, et al. Evaluation of the Effect and Safety of HeberFERON vs Heberon Alpha in Patients Infected with Corona Virus SARS-CoV-2 (Study ESPERANZA/HOPE): Study Protocol for a Randomized Controlled Trial. ResearchSquare. 2020. doi:10.21203/rs.3.rs-28958/v1
49. Irigoyen-Camacho ME, Velazquez-Alva MC, Zepeda-Zepeda MA, Cabrer-Rosales MF, Lazarevich I, Castano-Seiquer A. Effect of income level and perception of susceptibility and severity of covid-19 on stay-at-home preventive behavior in a group of older adults in Mexico City. Int J Environ Res Public Health. 2020;17: 1–16. doi:http://dx.doi.org/10.3390/ijerph17207418
50. Irvin R. On the Economic Benefits and Costs of COVID-19 Mitigation Measures in Mexico. SSRN. 2020. doi:10.2139/ssrn.3592209
51. Isaac JN, Pablo FB-Z, Yanink C-V. Impact of false negative results of RT-PCR test for SARS-CoV-2 in COVID-19 case count as applied to Mexico. medRxiv. 2020. doi:10.1101/2020.09.17.20197038
52. Isaac N, Pablo FB-Z, Yanink C-V. Corrected Estimation of COVID-19 Cases in Mexico: A Nation-Wide Open Data Modelling Study. SSRN. 2020. doi:10.2139/ssrn.3691134
53. Isabel RF, Mary C, Wesler L, Fernet Renand L, Bethany H-G, Wanying L, et al. Adapting Lot Quality Assurance Sampling to accommodate imperfect tests: application to COVID-19 serosurveillance in Haiti. medRxiv. 2020. doi:10.1101/2020.09.11.20193052
54. Isadora CC, Eloiza DF, Janaína C da S, Guilherme S, Daisy MS, Guilherme FS. Predictive model of COVID-19 incidence and socioeconomic description of municipalities in Brazil. medRxiv. 2020. doi:10.1101/2020.06.28.20141952
55. Isaure D, Julia E, Werner P. The Distributional Consequences of Social Distancing on Poverty and Labour Income Inequality in Latin America and the Caribbean. SSRN. 2020. doi:10.2139/ssrn.3710062
56. Isaure D, Werner P. Working From Home Under COVID-19: Who Is Affected? Evidence From Latin American and Caribbean Countries. SSRN. 2020. doi:10.2139/ssrn.3610885
57. Isea R. The Transmission Dynamics of Covid-19 from a MathematicalPerspective. Obs del Conoc Rev Espec en Gestión Soc del Conoc. 2020;5: 15–23.
58. Ismael C, Silva PAIA, Silva CM da, Melo MSV de, Neto BAF, Melo JV de, et al. Universal Screening of SARS-CoV-2 of Oncology Healthcare Workers — a Brazilian experience. 2020. doi:10.1590/SciELOPreprints.293
59. Ismael I-N, Kathia GF-R, Violeta R-H, Hilda CO-B, Alfonso S-Z, Magaly P-O, et al. Ethnic disparities in COVID-19 mortality in Mexico: a cross-sectional study based on national data. medRxiv. 2020. doi:10.1101/2020.08.26.20182543
60. Itiki R, Roy Chowdhury P. Fast deployment of COVID-19 disinfectant from common ethanol of gas stations in Brazil: COVID-19 disinfectant from common ethanol. Heal Policy Technol. 2020. doi:10.1016/j.hlpt.2020.07.002
61. Ivan F, Gustavo Almeida Lopes F, Guilherme F, Pedro Ivo S. Ideology, Isolation, and Death. An Analysis of the Effects of Bolsonarism in the COVID-19 Pandemic. SSRN. 2020. doi:10.2139/ssrn.3654538
62. Iwona H, Thomas AM, Henrique HH, Ricardo S, Swapnil M, Charles W, et al. Inference of COVID-19 epidemiological distributions from Brazilian hospital data. medRxiv. 2020. doi:10.1101/2020.07.15.20154617
63. Izabela Sobiech P. Untimely Reopening? Change in the Number of New COVID-19 Cases after Reopening in One Brazilian State. SSRN. 2020. doi:10.2139/ssrn.3623930
64. Jacno Erik Ferrer C, Ernesto Sánchez H, Abel Poulout M, Germán del Río C, Daicy Figueredo S. Caracterización clínica y epidemiológica de pacientes confirmados con la COVID-19 en la provincia de Santiago de Cuba. 2020;24.
65. Jacobo G-C, Ricardo M, Ernesto L-V, Fabricia R, Rogerio L. The Effect of Management Control Systems during the COVID-19 Pandemic: Does the Market Appreciate the Breadth of Vision? SSRN. 2020. doi:10.2139/ssrn.3675688
66. Jacques N, Bruce M, Joshua C, Fabio C, Salah D, Barry D, et al. Worldwide Effectiveness of Various Non-Pharmaceutical Intervention Control Strategies on the Global COVID-19 Pandemic: A Linearised Control Model. medRxiv. 2020. doi:10.1101/2020.04.30.20085316
67. Jaime AY, Sun Ah C, Fiorella I-B, Christian M. Demographic and Geographic COVID-19 Death Risk Factors in Peru. A Nationwide Analysis. SSRN. 2020. doi:10.2139/ssrn.3648543
68. Jaime B, Max S, Jesus A, Guadalupe G, Gustavo O, Rosa MW-C, et al. Risk of infection and hospitalization by Covid-19 in Mexico: a case-control study. medRxiv. 2020. doi:10.1101/2020.05.24.20104414
69. Jairo Jesus Gomez T, Ronny Alejandro Dieguez G, Manuel Ramon Perez A, Odalis Tamayo V, Aida Elizabeth Iparraguirre T. Evaluación del nivel de conocimiento sobre COVID-19 durante la pesquisa en la población de un consultorio. 16 Abril. 2020;59.
70. Javier M-G, Jorge Oscar G-M, Jessica M-S, Rodrigo Estefano R-C, Eduardo A-A, Hector Isaac R-G, et al. Use of antivirals and antibiotics for COVID-19 in Mexico City: A Real-World Multicenter Cohort Study. medRxiv. 2020. doi:10.1101/2020.10.13.20211797
71. Javier M-G, Juan Mauricio V-Z, Addi Rhode N-C, Orietta S-B, Gerardo R-V, Francisco Javier T-L, et al. Development and Validation of the Patient History COVID-19 (PH-Covid19) Scoring System: A Multivariable Prediction Model of Death in Mexican Patients with COVID-19. medRxiv. 2020. doi:10.1101/2020.09.05.20189142
72. Javier O-C, Javier D-N, Ana S-T, Rosa P-A, Laura P-A, Juan L-L, et al. Inequality in times of pandemics: How online media are starting to treat the economic consequences of the coronavirus crisis. Prof La Inf. 2020;29. doi:10.3145/EPI.2020.JUL.03
73. Jean Franco Q-C, Evelyn F-R, Diego M, Jesús M-Z, Katia Medalith H-C, Scherlli EC, et al. Prevalence of Self-Medication During the COVID-19 Pandemic in Peru. SSRN. 2020. doi:10.2139/ssrn.3688880
74. Jeany D, Luiz Claudio N, Rinaldo Focaccia S, Gisely Cardoso de M, Estela Natacha Brandt B, José Carlos N, et al. Covid-19 automated diagnosis and risk assessment through Metabolomics and Machine-Learning. medRxiv. 2020. doi:10.1101/2020.07.24.20161828
75. Jeff H-V, David V-Z, Angela P, María Alejandra L-C. Psychometric Properties of a Spanish Version of the Fear of COVID-19 Scale in General Population of Lima, Peru. Int J Ment Health Addict. 2020. doi:10.1007/S11469-020-00354-5
76. Jeronimo CMP, Farias MEL, Val FFA, Sampaio VS, Alexandre MAA, Melo GC, et al. Methylprednisolone as Adjunctive Therapy for Patients Hospitalized With COVID-19 (Metcovid): A Randomised, Double-Blind, Phase IIb, Placebo-Controlled Trial. Clin Infect Dis. 2020. doi:10.1093/cid/ciaa1177
77. Jessica  Sr. R-S, Valery  Sr. D-O, Diayan C-G, Jennifer  Sr. R-V, John B-O, Randol  Sr. V-A, et al. Hospital practice in COVID-19 times: Perceptions of the midwifery interns in Peru. medRxiv. 2020. doi:10.1101/2020.06.05.20094482
78. Jessyka Viana Valadares F, Thiago Franco O. O avanço do COVID-19 na Amazônia Legal : Uma análise do crescimento de casos na cidade de Gurupi- Tocantins. 2020;8. doi:10.18606/2318-1419/AMAZONIA.SCI.HEALTH.V8N2P64-76
79. Jesus Arturo  Sr. R-Q, Crystell  Sr. G-P, German Alberto  Sr. N-R, Carlos Alfonso T-Z, Oscar Israel F-B, Victor N-O, et al. Features of patients that died for COVID-19 in a Hospital in the south of Mexico: A observational cohort study. medRxiv. 2020. doi:10.1101/2020.09.21.20199117
80. Jesús Ojino S-G, Alan Omar G-V, Alondra G-B, Juan Pablo R-G, Eva J-H, Octavio G-C. Clinical Characteristics of Severe COVID-19 Patients in an ICU in Mexico. SSRN. 2020. doi:10.2139/ssrn.3605185
81. Jesús C-M, Panayotis K, Qian-Yong C, George K, Víctor V-D, Zoi R, et al. Lockdown Measures and their Impact on Single- and Two-age-structured Epidemic Model for the COVID-19 Outbreak in Mexico. medRxiv. 2020. doi:10.1101/2020.08.11.20172833
82. Jesus JG de, Sacchi C, Candido D da S, Claro IM, Sales FCS, Manuli ER, et al. Importation and early local transmission of COVID-19 in Brazil, 2020. Rev Inst Med Trop Sao Paulo. 2020;62: e30. doi:https://dx.doi.org/10.1590/s1678-9946202062030
83. Jimmy M-D, Eduardo V, Aurora M, Piedad S, Stefany P, Diana P, et al. Personal Protective Equipment, Diagnostic and Treatments Facilities for COVID-19 Patients. A Cross-Sectional Study in Brazil, Colombia and Ecuador. SSRN. 2020. doi:10.2139/ssrn.3667626
84. João Vitor A, Clarah Chaveiro MR. O que o Coronavírus tem nos tirado? Anos potenciais de vida perdidos em Minas Gerais. J Nurs Heal. 2020;10: 1–12. doi:10.15210/jonah.v10i4.19043
85. Joffily L, Ungierowicz A, David AG, Melo B, Brito CLT, Mello L, et al. The close relationship between sudden loss of smell and COVID-19. Braz J Otorhinolaryngol. 2020. doi:https://dx.doi.org/10.1016/j.bjorl.2020.05.002
86. Johnson CD, Green BN, Konarski-Hart KK, Hewitt EG, Napuli JG, Foshee WK, et al. Response of Practicing Chiropractors during the Early Phase of the COVID-19 Pandemic: A Descriptive Report. J Manipulative Physiol Ther. 2020. doi:https://dx.doi.org/10.1016/j.jmpt.2020.05.001
87. Johnson MC, Saletti-Cuesta L, Tumas N. [Emotions, concerns and reflections regarding the COVID-19 pandemic in Argentina]. Cien Saude Colet. 2020;25: 2447–2456. doi:https://dx.doi.org/10.1590/1413-81232020256.1.10472020
88. Joilson X, Marta G, Talita A, Vagner F, Alana Vitor Barbosa da C, Adriana Aparecida R, et al. The ongoing COVID-19 epidemic in Minas Gerais, Brazil: insights from epidemiological data and SARS-CoV-2 whole genome sequencing. medRxiv. 2020. doi:10.1101/2020.05.05.20091611
89. Jonatan G, Jeisson P, Elizabeth L, Arles R. INFEKTA: A General Agent-based Model for Transmission of Infectious Diseases: Studying the COVID-19 Propagation in Bogota - Colombia. medRxiv. 2020. doi:10.1101/2020.04.06.20056119
90. Jorge Humberto R, Marlio P, Malay B, Olcay A, Anuj M. Mathematical Modeling & the Transmission Dynamics of SARS-CoV-2 in Cali, Colombia: Implications to a 2020 Outbreak & public health preparedness. medRxiv. 2020. doi:10.1101/2020.05.06.20093526
91. Jorge A-C, Juan Pablo A, Patricio D-R. Activity and the Incidence of Emergencies: Evidence From Daily Data at the Onset of a Pandemic. SSRN. 2020. doi:10.2139/ssrn.3595246
92. Jorge P, Francisca C, Tania A, Maria Soledad M, Cristobal C. Gender disparities in access to care for time-sensitive conditions during COVID-19 pandemic in Chile. medRxiv. 2020. doi:10.1101/2020.09.11.20192880
93. José Alejandro M-P. Risk perception, coronavirus and precariousness. A reflection on fieldwork under quarantine. Heal Sociol Rev. 2020;29: 113–121. doi:10.1080/14461242.2020.1785321
94. José Augusto Santos P, Tatiana Helena R, Patrícia S, Ana Cláudia Tonelli de O, Tarsila V, Rafael Barberena M, et al. Incidence of Venous Thromboembolism Among Patients with Severe COVID-19 Requiring Mechanical Ventilation Compared to Other Causes of Respiratory Failure: A Prospective Cohort Study. SSRN. 2020. doi:10.2139/ssrn.3696842
95. José Edson L, Bruno Pelizzaro Dias A, Paulo Emílio Silva V, Tarcísio A. A NEW MODEL FOR A DISTRICT HEALTH SYSTEM SUPPLY CHAIN: PROPOSITION AND APPLICATION FROM CLASSIC TO CORONAVIRUS CARE. 2020;17. doi:10.21450/RAHIS.V17I1.6180
96. Jose Maria R-T, Antonio R-M, Marisol M-R, Laura Martinez-Perez M, Maria Antonieta S-G, Karina H-F, et al. Prevalence of SARS-CoV-2 IgG antibodies in a population from Veracruz (Southeastern Mexico). medRxiv. 2020. doi:10.1101/2020.10.19.20215558
97. Jose Paulo Guedes P, Patricia Camargo M, Gerusa Maria F, Domingos A, Diana Maritza S-A. Local protection bubbles: an interpretation of the decrease in the velocity of coronavirus’s spread in the city of Sao Paulo. medRxiv. 2020. doi:10.1101/2020.08.11.20173039
98. Jose M, Jose NR, Albida De L, Juan MC, Nelson T, Johnny T, et al. The use of compassionate Ivermectin in the management of symptomatic outpatients and hospitalized patients with clinical diagnosis of COVID-19 at the Medical Center Bournigal and the Medical Center Punta Cana, Rescue Group, Dominican Republic, from may 1 . medRxiv. 2020. doi:10.1101/2020.10.29.20222505
99. Jose P, Emanuel GD, Sebastian W, Veronica G, David W. Psychosocial aspects of the COVID-19 pandemic in the mendoza population. Rev Argent Cardiol. 2020;88: 203–206. doi:http://dx.doi.org/10.7775/rac.v88.i3.17925
100. Josimar ECS. Data Mining Approach to Analyze Covid19 Dataset of Brazilian Patients. medRxiv. 2020. doi:10.1101/2020.08.13.20174508
101. Josimar ECS. How was the Mental Health of Colombian people on March during Pandemics Covid19? medRxiv. 2020. doi:10.1101/2020.07.02.20145425
102. Josimar ECS. Infoveillance based on Social Sensors to Analyze the impact of Covid19 in South American Population. medRxiv. 2020. doi:10.1101/2020.04.06.20055749
103. Josimar ECS, Anabel P-B. Text Mining Approach to Analyze Coronavirus Impact: Mexico City as Case of Study. medRxiv. 2020. doi:10.1101/2020.05.07.20094466
104. Josimar ECS, Jimmy O. Covid19 Surveillance in Peru on April using Text Mining. medRxiv. 2020. doi:10.1101/2020.05.24.20112193
105. Josimar ECS, Roselyn L-M. Infoveillance to Analyze Covid19 Impact on Central America Population. medRxiv. 2020. doi:10.1101/2020.05.26.20113514
106. Juan Alonso L-A. Modeling the progression of SARS-CoV-2 infection in patients with COVID-19 risk factors through predictive analysis. medRxiv. 2020. doi:10.1101/2020.07.14.20154021
107. Juan Alonso L-A, Roha Saeed M, Bahar R, Maimoona I, Antara C. The impact of COVID-19 in diabetic kidney disease and chronic kidney disease: A population-based study. medRxiv. 2020. doi:10.1101/2020.09.12.20193235
108. Juan Carlos  Sr. R, Rocio M, Danny R, Mariana C, Marcela S, Lina C, et al. Social response to early-stage government control measures of COVID-19 in Colombia: population survey, April 8-20 2020. medRxiv. 2020. doi:10.1101/2020.06.18.20135145
109. Juan David R, Carolina F, Marina M, Carolina H, Adriana C, Sergio G, et al. The arrival and spread of SARS-CoV2 in Colombia. medRxiv. 2020. doi:10.1101/2020.06.11.20125799
110. Juan David R, Marina M, Carolina H, Carolina F, Sergio G, Angelica R, et al. Genetic diversity among SARS-CoV2 strains in South America may impact performance of Molecular detection. medRxiv. 2020. doi:10.1101/2020.06.18.20134759
111. Juan Felipe Medina M, Manuel Eduardo Cortes C, Manuel Cortes I, Annia del Carmen Perez F, Marianelis Manzano C. Study on predictive models for COVID-19 in Cuba. 2020;18.
112. Juan Felipe Medina M, Manuel Eduardo Cortés C, Manuel Cortés I, Annia del Carmen Pérez F, Marianelis Manzano C. Estudio sobre modelos predictivos para la COVID-19 en Cuba. 2020;18.
113. Juan Fidel O-R, Horacio Rendon A, Luis Adrian De J-G, Jose Manuel Reyes R, Arely Montserrat Espinoza O, Luis Antonio Ochoa R, et al. Serum lipid profile changes and their clinical diagnostic significance in COVID-19 Mexican Patients. medRxiv. 2020. doi:10.1101/2020.08.24.20169789
114. Juan Pablo G, Stefano MB. Non-communicable diseases and inequalities increase risk of death among COVID-19 patients in Mexico. medRxiv. 2020. doi:10.1101/2020.05.27.20115204
115. Juan AGC. SEI1I2HRSVM model applied to the coronavirus pandemic (COVID-19) in Paraguay. arXiv Popul Evol. 2020.
116. Juan F-S, Jose Enrique Olvera V, Juan Javier G-B, Guadalupe C-V, Juan Paulo S-H, Joaquin P-O. The Hybrid Forecasting Method SVR-ESAR forCovid-19. medRxiv. 2020. doi:10.1101/2020.05.20.20103200
117. Juan MCL. Sars-Cov-2 in Argentina: Following Virus Spreading using Granger Causality. medRxiv. 2020. doi:10.1101/2020.10.06.20207993
118. Juan MCL. Sars-Cov-2 in Argentina: Lockdown, Mobility, and Contagion. medRxiv. 2020. doi:10.1101/2020.10.03.20203232
119. Julian Alfredo F-N, Jhon AG-G, Alvaro Javier I-V. Multimorbidity patterns among COVID-19 deaths: considerations for a better medical practice. medRxiv. 2020. doi:10.1101/2020.07.28.20163816
120. Juliana Marques de A, Roberta Andrade de S, Livia Gomes V-M, Landeira-Fernandez J, Alberto F. Effects of physical activity and exercise on well-being in the context of the Covid-19 pandemic. medRxiv. 2020. doi:10.1101/2020.06.08.20125575
121. Juliane Fonseca O, Daniel CPJ, Rafael VV, Moreno SR, Matheus FT, Nivea B da S, et al. Evaluating the burden of COVID-19 on hospital resources in Bahia, Brazil: A modelling-based analysis of 14.8 million individuals. medRxiv. 2020. doi:10.1101/2020.05.25.20105213
122. Julio Cesar Sampaio D, Wellington Betencurte da S, Jose Mir Justino da C. Monitoring and forecasting the number of reported and unreported cases of the COVID-19 epidemic in Brazil using Particle Filter. medRxiv. 2020. doi:10.1101/2020.05.27.20115212
123. Junior J, Freire G, Gonçalves M, Oliveira D, Moraes J, Fidelix Y. Do Age and Symptoms of Anxiety, Stress and Depression Predict Risk Behaviors for Eating Disorders in Brazilian Men and Women During the Covid-19 Pandemic? ResearchSquare. 2020. doi:10.21203/rs.3.rs-100005/v1
124. Junqueira-Kipnis AP, dos Anjos LRB, Barbosa LCS, da Costa AC, Borges KCM, Cardoso ARO, et al. BCG revaccination of health workers in Brazil to improve innate immune responses against COVID-19: A structured summary of a study protocol for a randomised controlled trial. Trials. 2020;21: 881. doi:http://dx.doi.org/10.1186/s13063-020-04822-0
125. Kalbusch A, Henning E, Brikalski MP, Luca FVD, Konrath AC. Impact of coronavirus (COVID-19) spread-prevention actions on urban water consumption. Resour Conserv Recycl. 2020;163: 105098. doi:http://dx.doi.org/10.1016/j.resconrec.2020.105098
126. Kammar-Garcia A, Vidal-Mayo J de J, Vera-Zertuche JM, Lazcano-Hernandez M, Vera-Lopez O, Segura-Badilla O, et al. IMPACT OF COMORBIDITIES IN MEXICAN SARS-COV-2-POSITIVE PATIENTS: A RETROSPECTIVE ANALYSIS IN A NATIONAL COHORT. Rev Invest Clin. 2020;72: 151–158. doi:https://dx.doi.org/10.24875/RIC.20000207
127. Karina C, Marcos Montani C, Claudia Renata dos Santos B, Lourdes Conceicao M, Arthur C, Evaldo E, et al. COVID-19 Seroprevalence in Baixada Santista Metropolitan Area, Sao Paulo, Brazil. medRxiv. 2020. doi:10.1101/2020.08.28.20184010
128. katherine L-D, Christian Julian Villabona A, Jose AUC, Carlos Franco M, Diego Alejandro A-D, Liz SV-A, et al. Genomic epidemiology of SARS-CoV-2 in Colombia. medRxiv. 2020. doi:10.1101/2020.06.26.20135715
129. Katherine S-R, Lenin G-B, Jhon G, Fernanda S-G, Raul F, Andres  Sr. L-C, et al. Interim Analysis of Pandemic Coronavirus Disease 2019 (COVID-19) and the SARS-CoV-2 virus in Latin America and the Caribbean: Morbidity, Mortality and Molecular Testing Trends in the Region. medRxiv. 2020. doi:10.1101/2020.04.25.20079863
130. Kelly Cristine Lemos S, Maria Eliara Gomes L. A INSERÇÃO DE DUAS PSICÓLOGAS RESIDENTES EM TEMPOS DE COVID-19. 2020;14.
131. Kenzo A, Eduardo AU, Rodrigo V, Rodrigo W. The effect of COVID-19 on the economy: evidence from an early adopter of localized lockdowns. medRxiv. 2020. doi:10.1101/2020.09.21.20198887
132. Kerr L, Kendall C, Silva AAMD, Aquino EML, Pescarini JM, Almeida RLF, et al. COVID-19 in Northeast Brazil: achievements and limitations in the responses of the state governments. Cien Saude Colet. 2020;25: 4099–4120. doi:http://dx.doi.org/10.1590/1413-812320202510.2.28642020
133. Kevin SR, Sebastian D, Gyan B. The transmission of SARS-CoV-2 is likely comodulated by temperature and by relative humidity. medRxiv. 2020. doi:10.1101/2020.05.23.20111278
134. Khondoker Nazmoon N, Islam Md R. HAS COUNTRYWIDE LOCKDOWN WORKED AS A FEASIBLE MEASURE IN BENDING THE COVID-19 CURVE IN DEVELOPING COUNTRIES? medRxiv. 2020. doi:10.1101/2020.06.23.20138685
135. Klôh VP, Silva GD, Ferro M, Araújo E, de Melo CB, de Andrade Lima JRP, et al. The virus and socioeconomic inequality: An agent-based model to simulate and assess the impact of interventions to reduce the spread of COVID-19 in Rio de Janeiro, Brazil. Brazilian J Heal Rev. 2020;3: 3647–3673.
136. Kosugi EM, Lavinsky J, Romano FR, Fornazieri MA, Luz-Matsumoto GR, Lessa MM, et al. Incomplete and late recovery of sudden olfactory dysfunction in COVID-19. Braz J Otorhinolaryngol. 2020. doi:https://dx.doi.org/10.1016/j.bjorl.2020.05.001
137. Kowalski LP, Imamura R, Castro Junior G, Marta GN, Chaves ALF, Matos LL, et al. Effect of the COVID-19 Pandemic on the Activity of Physicians Working in the Areas of Head and Neck Surgery and Otorhinolaryngology. Int Arch Otorhinolaryngol. 2020;24: e258–e266. doi:10.1055/s-0040-1712169
138. Krüger-Malpartida H, Pedraz-Petrozzi B, Arevalo-Flores M, Salmavides-Cuba F, Anculle-Arauco V, Dancuart-Mendoza M. Effects on the mental health after COVID-19 lockdown period: results from a population survey study in Lima, Peru. ResearchSquare. 2020. doi:10.21203/rs.3.rs-81117/v1
139. Kumaira Fonseca M, Trindade EN, Costa Filho OP, Nácul MP, Seabra AP. Impact of COVID-19 Outbreak on the Emergency Presentation of Acute Appendicitis. Am Surg. 2020; 3134820972098. doi:10.1177/0003134820972098
140. Kunkel M, Vasques M, Perfeito J, Zambrana N, Bina T, Passoni L, et al. Mass-production and Distribution of Medical Face Shields Using Additive Manufacturing and Injection Molding Process for Healthcare System Support During COVID-19 Pandemic in Brazil. ResearchSquare. 2020. doi:10.21203/rs.3.rs-63872/v1
141. Kutralam-Muniasamy G, Pérez-Guevara F, Roy P, Elizalde-Martínez I, Shruti V. Impacts of the COVID-19 lockdown on air quality and its association with human mortality trends in megapolis Mexico City. ResearchSquare. 2020. doi:10.21203/rs.3.rs-90079/v1
142. Larissa F-M, Irma Eloisa M-M, Luis Antonio U-N, María de los Angeles H-C, Brenda S-M, Héctor Daniel P, et al. Weak association of coinfection by SARS-CoV-2 and other respiratory viruses with severe cases and death. medRxiv. 2020. doi:10.1101/2020.07.22.20159400
143. Laura AR-V, Luis Carlos B-C, Julian Alfredo F-N, Diana Marcela M-P, Oscar Alberto R-S, Lizbeth Alexandra A-M, et al. Air pollution, sociodemographic and health conditions effects on COVID-19 mortality in Colombia: an ecological study. medRxiv. 2020. doi:10.1101/2020.07.22.20159293
144. Laura A, Oscar B, Marcela E. EMEs and COVID-19: Shutting Down in a World of Informal and Tiny Firms. SSRN. 2020. doi:10.2139/ssrn.3626861
145. Lauren H-V, Adan S-M, Jose Roberto Balmori de la M. COVID-19 and Crimes Against Women: Evidence from Mexico. SSRN. 2020. doi:10.2139/ssrn.3701472
146. Lazarus J V, Ratzan S, Palayew A, Billari FC, Binagwaho A, Kimball S, et al. COVID-SCORE: A global survey to assess public perceptions of government responses to COVID-19 (COVID-SCORE-10). PLoS One. 2020;15: e0240011. doi:http://dx.doi.org/10.1371/journal.pone.0240011
147. Leal-Neto OB, Santos FAS, Lee JY, Albuquerque JO, Souza W V. Prioritizing COVID-19 tests based on participatory surveillance and spatial scanning. Int J Med Inform. 2020;143: 104263. doi:http://dx.doi.org/10.1016/j.ijmedinf.2020.104263

Onicio Batista Leal N, Francisco Assis S, Julia Yeri L, Jones A, Wayner Vieira S. Prioritizing COVID-19 tests based in Participatory Surveillance and Spatial Scanning. medRxiv. 2020. doi:10.1101/2020.05.25.20109058

1. Leandro Pereira G, Andre Vinicius G, Matheus Pacheco de A, Lucas Alexandre P, Ana Cristina V, Roberto Z, et al. ESTIMATING UNDERDIAGNOSIS OF COVID-19 WITH NOWCASTING AND MACHINE LEARNING: EXPERIENCE FROM BRAZIL. medRxiv. 2020. doi:10.1101/2020.07.01.20144402
2. Leandro Simões P, Nobuiuki Costa I. O surto da COVID-19 e as respostas da administração municipal: munificência de recursos, vulnerabilidade social e eficácia de ações públicas. 2020.
3. Leila F D, Igor T P, Leonardo S.L B, Janaina F M, Guilherme F.G. De S, Joao Gabriel M G, et al. App-based symptom tracking to optimize SARS-CoV-2 testing strategy using machine learning. medRxiv. 2020. doi:10.1101/2020.09.01.20186049
4. Lemos ACB, do Espirito Santo DA, Salvetti MC, Gilio RN, Agra LB, Pazin-Filho A, et al. Therapeutic versus prophylactic anticoagulation for severe COVID-19: a randomized phase II clinical trial (HESACOVID). Thromb Res. 2020;196: 359‐366. doi:10.1016/j.thromres.2020.09.026
5. Lemos DRQ, D’Angelo SM, Farias LABG, Almeida MM, Gomes RG, Pinto GP, et al. Health system collapse 45 days after the detection of COVID-19 in Ceara, Northeast Brazil: a preliminary analysis. Rev Soc Bras Med Trop. 2020;53: e20200354. doi:https://dx.doi.org/10.1590/0037-8682-0354-2020
6. Lenisse MR, Lilibeth O, Maxwell A, Yenifel L, Wilma R, Pablo J de JR. Misinformation on COVID-19 origin and social distancing: A cross-sectional study. medRxiv. 2020. doi:10.1101/2020.10.06.20207894
7. Leonardo JB, Felipe S, Hugo ES. Public Transport Policies after COVID-19 Confinement. SSRN. 2020. doi:10.2139/ssrn.3693098
8. Leung C. The younger the milder clinical course of COVID-19: Even in newborns? Pediatr Allergy Immunol. 2020. doi:http://dx.doi.org/10.1111/pai.13371
9. Lewis FB, Carlos Augusto  Jr. P, Claudia MMA, Alfredo  Jr. M, Tassila S, Cesar de A-N, et al. COVID-19 herd immunity in the Brazilian Amazon. medRxiv. 2020. doi:10.1101/2020.09.16.20194787
10. Liana RW, Clare W. Do Men and Women Lockdown Differently? An Examination of Panamas COVID-19 Sex-Segregated Social Distancing Policy. medRxiv. 2020. doi:10.1101/2020.06.30.20143388
11. Lima DLF, Dias AA, Rabelo RS, Cruz ID da, Costa SC, Nigri FMN, et al. Covid-19 in the State of Ceara: behaviors and beliefs in the arrival of the pandemic. Cien Saude Colet. 2020;25: 1575–1586. doi:https://dx.doi.org/10.1590/1413-81232020255.07192020
12. Lima DLF, Veras PJL, Marques TM, Costa SC, Santos HPG dos, Neri JR. Transmission care: what led Ceará to be the epicenter of COVID-19? Rev bras promoç saúde. 2020;33: 1–14.
13. Lima E de P, Vasconcelos AG, Corrêa LRT, Batista AG. Baixas na linha de frente: absenteísmo entre bombeiros durante o combate à pandemia da COVID-19. Rev Bras Saúde Ocup. 2020;45. doi:10.1590/2317-6369000016420
14. Lima FET, Albuquerque NLS de, Florencio S de SG, Fontenele MGM, Queiroz APO, Lima GA, et al. Time interval between onset of symptoms and COVID-19 testing in Brazilian state capitals, August 2020. 2020.
15. Lima KC de, Medeiros A de A, Barbosa IR. Trend analysis of cases and deaths by Coronavirus Disease-2019 (COVID-19) in the main affected countries and in Brazil: an analysis of the first 50 days of the pandemic. J Heal Biol Sci. 2020;8.
16. Lima L. Modeling and forecasting of epidemic spreading of the SARS-CoV-2 based in the nonlinear stochastic dynamics. ResearchSquare. 2020. doi:10.21203/rs.3.rs-29533/v1
17. Lima L. Modelling based in the stochastic dynamics for the time evolution of the COVID-19. ResearchSquare. 2020. doi:10.21203/rs.3.rs-23014/v1
18. Lima L. Stochastic analysis using public data for forecasting of epidemic spreading of the novel coronavirus disease. ResearchSquare. 2020. doi:10.21203/rs.3.rs-78321/v1
19. Lima MA, Silva MTT, Oliveira R V, Soares CN, Takano CL, Azevedo AE, et al. Smell dysfunction in COVID-19 patients: More than a yes-no question. J Neurol Sci. 2020;418: 117107. doi:http://dx.doi.org/10.1016/j.jns.2020.117107
20. Lima-Costa MF, Macinko J, Andrade FB, Souza Junior PRB, Vasconcellos MTL, Oliveira CM. ELSI-COVID-19 initiative: methodology of the telephone survey on coronavirus in the Brazilian Longitudinal Study of Aging. Cad Saude Publica. 2020;36Suppl 3: e00183120. doi:http://dx.doi.org/10.1590/0102-311X00183120

Lima-Costa MF, Mambrini JVM, Andrade FB, Peixoto SW V, Macinko J. Social distancing, use of face masks and hand washing among participants in the Brazilian Longitudinal Study of Aging: the ELSI-COVID-19 initiative. Cad Saude Publica. 2020;36Suppl 3: e00193920. doi:http://dx.doi.org/10.1590/0102-311X00193920

1. Lincoln Luis S, Amanda Carvalho D, Pedro Henrique I, Guilherme Luiz Rodrigues R, Gabriel Antonio Fernandes M, Iago Amado Peres G, et al. Brazil Health Care System preparation against COVID-19. medRxiv. 2020. doi:10.1101/2020.05.09.20096719
2. Lino DODC, Barreto R, Souza FDD, Lima CJMD, Silva Junior GBD. Impact of lockdown on bed occupancy rate in a referral hospital during the COVID-19 pandemic in northeast Brazil. Brazilian J Infect Dis. 2020;24: 466–469. doi:http://dx.doi.org/10.1016/j.bjid.2020.08.002
3. Lins S, Aquino S. Development and initial psychometric properties of a panic buying scale during COVID-19 pandemic. Heliyon. 2020;6: e04746. doi:10.1016/j.heliyon.2020.e04746
4. Lins-Filho PC, Araújo MMS de, Macêdo TS de, Melo MCF de, Ferreira AKA, Silva ELMS da, et al. The impact of socioeconomic vulnerability on COVID-19 outcomes and social distancing in Brazil. 2020.
5. Lira-Lucio JA, Roldan-Rodriguez E, Ochoa-Millan JG, Hernandez-Escobar L, Padilla-Rivera CI, Ochoa-Gaitan G. Factors associated with poor forecasting in pregnant women with SARS-Cov-2 diagnosis. Ginecol Obstet Mex. 2020;88: 450–457. doi:http://dx.doi.org/10.24245/gom.v88i7.4242
6. Lisandro L, Diego HSC, Rodrigo BB, Malu G, Pamella C-T, Americo  Jr. C. COVID-19 mortality according to civilian records. medRxiv. 2020. doi:10.1101/2020.08.07.20170183
7. Lizet S, Patricia L-L, Carlos S, Antonio T, Claudia F, Marelis C, et al. How Mathematical Approaches Could Help Decision-Making to Epidemic Control? The Successful Experience against COVID-19 in Cuba. SSRN. 2020. doi:10.2139/ssrn.3629271
8. Lobo A de P, Cardoso-Dos-Santos AC, Rocha MS, Pinheiro RS, Bremm JM, Macario EM, et al. COVID-19 epidemic in Brazil: Where are we at? Int J Infect Dis. 2020;97: 382–385. doi:https://dx.doi.org/10.1016/j.ijid.2020.06.044
9. Lopes RD, Macedo AVS, de Barros e Silva PGM, Moll-Bernardes RJ, Feldman A, D’Andrea Saba Arruda G, et al. Continuing versus suspending angiotensin-converting enzyme inhibitors and angiotensin receptor blockers: Impact on adverse outcomes in hospitalized patients with severe acute respiratory syndrome coronavirus 2 (SARS-CoV-2)-The BRACE CORONA Trial: BRACE CO. Am Heart J. 2020;226: 49–59. doi:http://dx.doi.org/10.1016/j.ahj.2020.05.002
10. Lopez Santi R, Piskorz DL, Marquez MF, Ramirez Ramos C, Renna NF, Ibarrola M, et al. Impact of the Pandemic on NonInfected Cardiometabolic Patients: A Survey in Countries of Latin America-Rationale and Design of the CorCOVID LATAM Study. CJC Open. 2020. doi:http://dx.doi.org/10.1016/j.cjco.2020.08.007
11. López JH, Romo Á S, Molina DC, Hernández GÁ, Gutiérrez Cureño Á B, Acosta MA, et al. DETECTION OF SARS-CoV-2 IN AIR OF TWO HOSPITAL IN HERMOSILLO, SONORA, MÉXICO, UTILIZING A LOW-COST ENVIRONMENTAL MONITORING SYSTEM. Int J Infect Dis. 2020. doi:10.1016/j.ijid.2020.10.089
12. Lopez-Aguilar E, Cardenas-Navarrete R, Simental-Toba A, Pacheco-Rosas D, Thome-Ortiz P, Soto-Perez G, et al. Children with cancer during COVID-19 pandemic: Early experience in Mexico. Pediatr Blood Cancer. 2020. doi:http://dx.doi.org/10.1002/pbc.28660
13. Lorena P, Paulette L, Valeska V, Ximena A, José MM, Rafael A, et al. Evaluation of Novel Antigen-Based Rapid Detection Test for the Diagnosis of SARS-CoV-2 in Respiratory Samples. SSRN. 2020. doi:10.2139/ssrn.3569871
14. Lucas  Sr. J, Jose Alexandre  Sr. D-F, Thiago Fernando  Sr. R, Cristiana Maria  II T. The effective reproductive number (Rt) of COVID-19 and its relationship with social distancing. medRxiv. 2020. doi:10.1101/2020.07.28.20163493
15. Lucchetti G, Goes LG, Amaral SG, Ganadjian GT, Andrade I, Almeida POA, et al. Spirituality, religiosity and the mental health consequences of social isolation during Covid-19 pandemic. Int J Soc Psychiatry. 2020; 20764020970996. doi:http://dx.doi.org/10.1177/0020764020970996
16. Lucélia Maria Carneiro da S, Laécio Guedes do N, Francisca das Chagas O, Linardy de Moura S, Samuel Campelo D, Samuel Jônatas de Castro L. Civil Construction Scenario in Brazil during the COVID-19 Pandemic. Res Soc Dev. 2020;9. doi:10.33448/RSD-V9I7.4464
17. Lucena EHG de, Freire AR, Freire DEWG, Araújo ECF de, Lira G de NW, Brito ACM, et al. Access to oral health in primary care before and after the beginning of the COVID-19 pandemic in Brazil. 2020.
18. Lucia CP, Eliana MW, Alan JAM, Luciana T-R, Marcos RAF, Odir AD, et al. Sensitivity and specificity of a rapid test for assessment of exposure to SARS-CoV-2 in a community-based setting in Brazil. medRxiv. 2020. doi:10.1101/2020.05.06.20093476
19. Luciano Magalhães V, Gerson Hiroshi Yoshinari J, Gabriela G, Isabela Faria D, Isabela Marum Góes R, João Pedro Lambert P, et al. Impact of Social Isolation Strategies Due to COVID-19 on Mental Health and Quality of Life in Brazil. SSRN. 2020. doi:10.2139/ssrn.3666238
20. Luciano D, Daniela P, Claudia M, Almir  Jr. T, Silvia GC, Marcia A, et al. Sleep Disturbances, Anxiety, and Burnout during the COVID-19 Pandemic: a nationwide cross-sectional study in Brazilian Healthcare Professionals. medRxiv. 2020. doi:10.1101/2020.09.08.20190603
21. Lucila C, Daniel P, Aldo M, Laura B, Rosana C, Cristina F, et al. Clinical Characteristics of Patients Coinfected with SARS-CoV-2 and Dengue. SSRN. 2020. doi:10.2139/ssrn.3618313
22. Lugo I, Alatriste-Contreras M. Intervention strategies with 2D cellular automata for testing SARS-CoV-2 and reopening the economy. ResearchSquare. 2020. doi:10.21203/rs.3.rs-40739/v1
23. Luis Cristovao P, Claudia HC, Alessandra SN, Isabel B, Tiago F, Vinicius MP, et al. Clinical and laboratory characteristics in outpatient diagnosis of COVID-19 in healthcare professionals in Rio de Janeiro, Brazil. medRxiv. 2020. doi:10.1101/2020.10.22.20217851
24. Luis Eduardo Alencar S, Gilberto de S, Layane Aiala de Sousa L, Rosana de Oliveira P, Tiago Medeiros da S, Alan Rodrigues da S, et al. Epidemiological profile of Covid-19 morbidity and mortality in the State of Piauí: an update of the current scenario. Res Soc Dev. 2020;9. doi:10.33448/RSD-V9I8.6091
25. Luis Fernando  Sr. LP, Ivan  Jr. II. LUNG ULTRASOUND FINDINGS IN MEXICAN PATIENTS WITH SARS COV2 INFECTION. medRxiv. 2020. doi:10.1101/2020.07.16.20146704
26. Luis Pendola G, Elizalde R, Vargas PS, Mallarino JC, Gonzalez E, Parada J, et al. Management of non-invasive tumours, benign tumours and breast cancer during the COVID-19 pandemic: Recommendations based on a Latin American survey. Ecancermedicalscience. 2020;14: 1115. doi:http://dx.doi.org/10.3332/ECANCER.2020.1115
27. Luisa Campos Caldeira B, Bruno Ramos N, Renato T, Marcelo Antonio Queiroga L, Deborah Carvalho M, Glaucia Maria Moraes O, et al. Excess of Cardiovascular Deaths During the COVID-19 Pandemic in Brazilian Capital Cities. medRxiv. 2020. doi:10.1101/2020.06.24.20139295
28. Luiz B, Tiago C, Daniel Da M, Cezar S. Slums and Pandemics. SSRN. 2020. doi:10.2139/ssrn.3665695
29. Luizi Basso de S, Denise Gonçalves S, Laís Siepmann SK, Naiana Oliveira dos S, Regina Caino Teixeira MM. Estágio curricular supervisionado em enfermagem durante a pandemia de Coronavírus: experiências na atenção básica. J Nurs Heal. 2020;10: 1–10. doi:10.15210/jonah.v10i4.19050
30. Luján-Piedrahíta M. Virtuality in the internal medicine theoretical course in students of V, VI and VII semester as a result of the COVID-19 pandemic during the first semester of 2020, School of Medicine, School of Health Sciences, Universidad Pontificia Bolivariana, Medellí. Med UPB. 2020;39: 66–72.
31. Lumbreras-Marquez MI, Campos-Zamora M, Seifert SM, Kim J, Lumbreras-Marquez J, Vazquez-Alaniz F, et al. Excess Maternal Deaths Associated With Coronavirus Disease 2019 (COVID-19) in Mexico. Obstet Gynecol. 2020. doi:http://dx.doi.org/10.1097/AOG.0000000000004140
32. Lumbreras-Marquez MI, Campos-Zamora M, Lizaola-Diaz de Leon H, Farber MK. Maternal mortality from COVID-19 in Mexico. Int J Gynaecol Obstet. 2020. doi:https://dx.doi.org/10.1002/ijgo.13250
33. Lyra W, do Nascimento  Jr. J-D, Belkhiria J, de Almeida L, Chrispim PPM, de Andrade I. COVID-19 pandemics modeling with modified determinist SEIR, social distancing, and age stratification. The effect of vertical confinement and release in Brazil. PLoS One. 2020;15: e0237627. doi:https://dx.doi.org/10.1371/journal.pone.0237627

Wladimir L, Jose Dias do N, Jaber B, Leandro de A, Pedro Paulo C, Ion de A. COVID-19 pandemics modeling with SEIR(+CAQH), social distancing, and age stratification. The effect of vertical confinement and release in Brazil. medRxiv. 2020. doi:10.1101/2020.04.09.20060053

1. Macarena P-L. Tweets de la autoridad sanitaria en Chile en los albores de la crisis del coronavirus. Rev Esp Comun en Salud. 2020;11: 117–127. doi:10.20318/RECS.2020.5447
2. Macarena RV, Anne P, Inia P, Maria S, Mario B, Mabel A, et al. Clinical and Epidemiological Characteristics of the First Month of the Covid-19 Pandemic in Chile. medRxiv. 2020. doi:10.1101/2020.08.11.20171975
3. Machado CJ, Pereira CC de A, Viana B de M, Oliveira GL, Melo DC, Carvalho JFMG de, et al. Estimates of the impact of COVID-19 on mortality of institutionalized elderly in Brazil. Estim impacto da COVID-19 na mortalidade idosos Inst no Bras. 2020;25: 3437–3444. doi:https://dx.doi.org/10.1590/1413-81232020259.14552020
4. Machado MH, Pereira EJ, Ximenes Neto FRG, Wermelinger MC de MW. Enfermagem em tempos de COVID-19 no Brasil: um olhar da gestão do trabalho. Enferm foco. 2020;11: 32–39.
5. Maciel EL, Jabor P, Goncalves Junior E, Tristao-Sa R, Lima R de CD, Reis-Santos B, et al. Factors associated with COVID-19 hospital deaths in Espirito Santo, Brazil, 2020. Fatores Assoc ao obito Hosp por COVID-19 no Espirito Santo, 2020. 2020;29: e2020413. doi:https://dx.doi.org/10.1590/S1679-49742020000400022
6. Maciel EL, Jabor P, Júnior EG, Tristão-Sá R, Lima R de CD, Reis-Santos B, et al. Mortality in patients admitted to hospital by COVID-19 in Espírito Santo, Brazil, 2020. 2020.
7. Maciel FBM, Santos HLPCD, Carneiro RADS, Souza EA, Prado NMBL, Teixeira CFS. Community health workers: reflections on the health work process in Covid-19 pandemic times. Cien Saude Colet. 2020;25: 4185–4195. doi:http://dx.doi.org/10.1590/1413-812320202510.2.28102020
8. Maciel JAC, Castro-Silva II, Farias MR de. Initial analysis of the spatial correlation between the incidence of COVID-19 and human development in the municipalities of the state of Ceara in Brazil. Rev Bras Epidemiol. 2020;23: e200057. doi:https://dx.doi.org/10.1590/1980-549720200057
9. Macinko J, Woolley NO, Seixas B V, Andrade FB, Lima-Costa MF. Health care seeking due to COVID-19 related symptoms and health care cancellations among older Brazilian adults: the ELSI-COVID-19 initiative. Cad Saude Publica. 2020;36Suppl 3: e00181920. doi:http://dx.doi.org/10.1590/0102-311X00181920
10. Madhuvanti MM, Selvi MJ, Christina H, Natalie G, Heather H, Kim RQ, et al. COVID-19 containment in the Caribbean: the experience of Small Island Developing States. medRxiv. 2020. doi:10.1101/2020.05.27.20114538
11. Mækelæ MJ, Reggev N, Dutra N, Tamayo RM, Silva-Sobrinho RA, Klevjer K, et al. Perceived efficacy of COVID-19 restrictions, reactions and their impact on mental health during the early phase of the outbreak in six countries. R Soc open Sci. 2020;7: 200644. doi:10.1098/rsos.200644
12. Magalhaes JJFD, Mendes RPG, Silva CTAD, Silva SJRD, Guarines KM, Pena L. Epidemiological and clinical characteristics of the first 557 successive patients with COVID-19 in Pernambuco state, Northeast Brazil. Travel Med Infect Dis. 2020;38: 101884. doi:http://dx.doi.org/10.1016/j.tmaid.2020.101884
13. Magalhaes M, Arruda do Rego L, Vieira de Reboucas C, de Brito Alves R, Adami F, Cruz FJSM. Clinical and epidemiologic aspects of patients with cancer and COVID-19 in a Brazilian cancer center. Ann Oncol. 2020;31: S1027. doi:http://dx.doi.org/10.1016/j.annonc.2020.08.1834
14. Malagón-Rojas J, Gómez-Rendón C, Parra EL, Almentero J, Palma R, López R, et al. SARS-CoV-2 and RT-PCR in asymptomatic patients: Results of a cohort of workers at El Dorado International Airport in Bogotá, 2020. Biomedica. 2020;40: 166–172. doi:10.7705/biomedica.5802
15. Malgor RD, Sobreira ML, Mouawad NJ, Johnson AP, Wohlauer M V, Coogan SM, et al. Brazilian vascular surgeons experience during the coronavirus (COVID-19) pandemic. Vascular. 2020. doi:http://dx.doi.org/10.1177/1708538120954961
16. Malta DC, Szwarcwald CL, Barros MB de A, Gomes CS, Machado IE, Souza Junior PRB de, et al. The COVID-19 Pandemic and changes in adult Brazilian lifestyles: a cross-sectional study, 2020. A pandemia da COVID-19 e as mudancas no estilo vida dos Bras adultos um Estud transversal, 2020. 2020;29: e2020407. doi:https://dx.doi.org/10.1590/S1679-49742020000400026
17. Mamani-Benito O, Apaza Tarqui EE, Carranza Esteban RF, Rodriguez-Alarcon JF, Mejía CR. Perceived job insecurity in employment due to the impact of COVID-19: validation of an instrument on Peruvian workers (LABOR-PE-COVID-19). Rev Asoc Esp Espec Med Trab. 2020;29: 184–193.
18. Manchein C, Brugnago EL, da Silva RM, Mendes CFO, Beims MW. Strong correlations between power-law growth of COVID-19 in four continents and the inefficiency of soft quarantine strategies. Chaos. 2020;30: 41102. doi:https://dx.doi.org/10.1063/5.0009454
19. Manrique-Abril FG, Agudelo-Calderon CA, González-Chordá VM, Gutiérrez-Lesmes O, Téllez-Piñerez CF, Herrera-Amaya G. SIR model of the COVID-19 pandemic in Colombia. Rev salud pública. 2020;22: e185977–e185977.
20. Manrique-Hernández EF, Moreno-Montoya J, Hurtado-Ortiz A, Prieto-Alvarado FE, Idrovo Á J. Performance of the Colombian surveillance system during the COVID-19 pandemic: A rapid evaluation of the first 50 days. Biomedica. 2020;40: 96–103. doi:10.7705/biomedica.5582
21. Manuel Adrian A-Z, Andreu C-G, Esteban H-V, Mario S-C, Jorge XV-H. The SARS-CoV-2 epidemic outbreak: a review of plausible scenarios of containment and mitigation for Mexico. medRxiv. 2020. doi:10.1101/2020.03.28.20046276
22. Manuel A, Santiago V, Fernando V-E, Ricardo S-R, Gonzalo PB, Jonas C, et al. SARS-CoV-2 Detection in Sewage in Santiago, Chile - Preliminary results. medRxiv. 2020. doi:10.1101/2020.07.02.20145177
23. Maquilon C, Gongora J, Antolini M, Alvarado B, Valdes N, Benavente A, et al. Risk factors on admission and condition at discharge of 529 consecutive COVID-19 patients at a tertiary care center in Santiago, Chile. ResearchSquare. 2020. doi:10.21203/rs.3.rs-71187/v1
24. Marcel G, Mirko B-L, Magdalena B, Leonardo JB. COVID-19: Short-Term Forecast of ICU Beds in Times of Crisis. SSRN. 2020. doi:10.2139/ssrn.3693447
25. Marcelo Domingos M. TAMING COVID-19 EPIDEMIC IN SAO PAULO WITH ALOGISTIC MODEL AND NON-PHARMACEUTICAL MEASURES. medRxiv. 2020. doi:10.1101/2020.05.06.20093666
26. Marcelo Henrique Santos P, Duschinka Ribeiro Duarte G, Cassia D, Matheus Filgueira B, Filipe Zimmer D, Lais Ceschini M, et al. Multiple introductions followed by ongoing community spread of SARS-CoV-2 at one of the largest metropolitan areas in the Northeast of Brazil. medRxiv. 2020. doi:10.1101/2020.08.25.20171595
27. Marcelo M, Mehran S, Cesar C, Joao AMG. ASSESSING THE EFFICIENCY OF DIFFERENT CONTROL STRATEGIES FOR THE COVID-19 EPIDEMIC. Electron J Differ Equations. 2020.

César C, João AMG, Marcelo M, Mehran S. Assessing the Efficiency of Different Control Strategies for the Coronavirus (COVID-19) Epidemic. arXiv Popul Evol. 2020.

1. Marcia CC, Lucas Resende de C, Taylor C, Rebecca K, Giovanny VAF, Eduardo Marques M, et al. Demand for hospitalization services for COVID-19 patients in Brazil. medRxiv. 2020. doi:10.1101/2020.03.30.20047662
2. Marcos Montani C, Monica M, Bruno B, Estela Capelas B, Antonio Pires B. Positivity of SARS-CoV-2, by RT-PCR among workers of a Public Hospital in the city of Santos, SP, Brazil 2020. medRxiv. 2020. doi:10.1101/2020.06.30.20143529
3. Marcos Roberto M, Ricardo Vicente F, Rogerio HT, Luiza A, Michael Richard D, Eric MD. Detecting space-time clusters of COVID-19 in Brazil: mortality, inequality, socioeconomic vulnerability, and the relative risk of the disease in Brazilian municipalities. medRxiv. 2020. doi:10.1101/2020.06.14.20131102
4. Marcos AC, Antonio C, Christen JA. Forecasting hospital demand in metropolitan areas during the current COVID-19 pandemic and estimates of lockdown-induced 2nd waves. medRxiv. 2020. doi:10.1101/2020.07.16.20155721
5. Marcos M, Pablo Rodriguez M, Carlos M-G, Victor L, Francisco G. Mathematical estimation of COVID-19 prevalence in Latin America. medRxiv. 2020. doi:10.1101/2020.06.09.20126326
6. Margaret S, Carlos D, Matthew BBM, Adrian G, Miguel AS-M, Michael KM, et al. The association of UV with rates of COVID-19 transmission and deaths in Mexico: the possible mediating role of vitamin D. medRxiv. 2020. doi:10.1101/2020.05.25.20112805
7. María Alejandra B, Carolina V, Ana Rita S, Josefa H, Flavio MM, Francesco P. COVID-19 Pandemic in Five Countries of Latin America. SSRN. 2020. doi:10.2139/ssrn.3663292
8. Maria de Fátima Rodrigues D, Maira Freire C, Karen Saori Shiraishi S, Carolina Rocha Brito M, Alessandro Cavalcanti L, Maria Fernanda Bádue P, et al. Pediatric Heart in COVID-19: New Insights from a Systematic Echocardiographic Study in a Tertiary Brazilian Hospital. SSRN. 2020. doi:10.2139/ssrn.3658311
9. María Elvira B, Luis R, Nicole Le C, Constanza M-V, María Elena C, Marcela F, et al. Early Anti-SARS-CoV-2 Convalescent Plasma in Patients Admitted for COVID-19: A Randomized Phase II Clinical Trial. medRxiv. 2020. doi:10.1101/2020.09.17.20196212
10. Maria Gerusa Brito A, Francisco Isaac Fernandes G, Camila Siqueira Silva C, Leticia Pinho Maia Paixao de M, Silmara Aparecida Milori C. Where do Brazilian dental students seek information about COVID-19? medRxiv. 2020. doi:10.1101/2020.08.24.20179614
11. Maria Gerusa Brito A, Francisco Isaac Fernandes G, Leticia Pinho Maia Paixao de M, Silmara Aparecida Milori C. Brazilian dental students and COVID-19: a survey on knowledge and perceptions. medRxiv. 2020. doi:10.1101/2020.07.28.20163964
12. Mariangela S, Aluisio B, Bernardo H, Lucia P, Gabriel V, Odir D, et al. Repeated population-based surveys of antibodies against SARS-CoV-2 in Southern Brazil. medRxiv. 2020. doi:10.1101/2020.05.01.20087205
13. Marinelli NP, Albuquerque LP de A, Sousa IDB de, Batista FM de A, Mascarenhas MDM, Rodrigues MTP. Evolution of indicators and service capacity at the beginning of the COVID-19 epidemic in Northeast Brazil, 2020. Epidemiol e Serv saude  Rev do Sist Unico Saude do Bras. 2020;29: e2020226. doi:https://dx.doi.org/10.5123/S1679-49742020000300008
14. Marín-Sánchez A. [Basic clinical characteristics in the first 100 fatal cases of COVID-19 in Colombia]. Rev Panam Salud Publica. 2020;44: e87. doi:10.26633/RPSP.2020.87
15. Mario Arturo Ruiz E. Can COVID-19 Shows Income Inequality? SSRN. 2020. doi:10.2139/ssrn.3638160
16. Mario Arturo Ruiz E. How COVID-19 Quarantine(s) Can Generate Poverty? SSRN. 2020. doi:10.2139/ssrn.3580703
17. Mario Arturo Ruiz E. Is Poverty the Best Allied of COVID-19 in Developing Countries? The Case of Guatemala. SSRN. 2020. doi:10.2139/ssrn.3645045
18. Mario S-C, Manuel Adrian A-Z, Jorge XV-H. Flattening the curve and the effect of atypical events on mitigation measures in Mexico: a modeling perspective. medRxiv. 2020. doi:10.1101/2020.05.21.20109678
19. Marisa S, Tereza Fellipe G, Helena Cramer R, Fabiana M, Adriana C, Izabella B, et al. Epidemic Curve of Contamination in a Hospital That Served as Sentinel of the Spread of the SARS-Cov-2 Epidemic in the City of Rio de Janeiro. medRxiv. 2020. doi:10.1101/2020.10.19.20215079
20. Marques C, Kakehasi AM, Gomides APM, Paiva EDS, Dos Reis Neto ET, Pileggi GCS, et al. ReumaCoV Brasil Registry: a Brazilian cohort of Patients with Immuno-mediated Chronic Inflammatory Diseases Infected by SARS-CoV-2. JMIR Res Protoc. 2020. doi:10.2196/24357

Marques C, Kakehasi A, Gomides A, Martins D, Paiva E, Neto E, et al. ReumaCoV Brasil Registry: Brazilian Study of Patients with Immuno-mediated Chronic Inflammatory Diseases Infected by SARS-CoV-2 . ResearchSquare. 2020. doi:10.21203/rs.3.pex-1104/v1

1. Marques NP, Silveira DMM, de Lima Martelli PJ, Martelli DRB, Hilan de Lucena E, Martelli-Junior H. Brazilian Oral Medicine and public health system: the enormous impact of the COVID-19 Era. Oral Dis. 2020. doi:http://dx.doi.org/10.1111/odi.13677
2. Marquez S, Prado-Vivar B, Guadalupe JJ, Gutierrez B, Becerra-Wong M, Jibaja M, et al. Metagenome of a bronchoalveolar lavage fluid sample from a confirmed COVID-19 case in quito, ecuador, obtained using oxford nanopore MinION technology. Microbiol Resour Announc. 2020;9: e00996-20. doi:http://dx.doi.org/10.1128/MRA.00996-20
3. Marquez S, Prado-Vivar B, Guadalupe JJ, Gutierrez Granja B, Jibaja M, Tobar M, et al. Genome sequencing of the first SARS-CoV-2 reported from patients with COVID-19 in Ecuador. medRxiv  Prepr Serv Heal Sci. 2020. doi:https://dx.doi.org/10.1101/2020.06.11.20128330
4. Marquiony S, Eudes ESL, Kenio CL, Andiara ACB, Monica BB, Diego B. Survival and predictors of deaths of patients hospitalized due to COVID-19 from a retrospective and multicenter cohort study in Brazil. medRxiv. 2020. doi:10.1101/2020.06.07.20125047
5. Martin RS, Soledad EG, Lorena R, Noelia SF, Veronica G, Patricia MC, et al. EFFECT OF CONVALESCENT PLASMA ON MORTALITY IN PATIENTS WITH COVID-19 PNEUMONIA. medRxiv. 2020. doi:10.1101/2020.10.08.20202606
6. Martinez D, Sarria GJ, Wakefield D, Flores C, Malhotra S, Li B, et al. COVID’s Impact on Radiation Oncology: A Latin American Survey Study. Int J Radiat Oncol Biol Phys. 2020;108: 374–378. doi:https://dx.doi.org/10.1016/j.ijrobp.2020.06.058
7. Martinez EZ, Silva FM, Morigi TZ, Zucoloto ML, Silva TL, Joaquim AG, et al. Physical activity in periods of social distancing due to COVID-19: a cross-sectional survey. Cien Saude Colet. 2020;25: 4157–4168. doi:http://dx.doi.org/10.1590/1413-812320202510.2.27242020
8. Martinez EZ, Aragon DC, Nunes AA. Long-term forecasts of the COVID-19 epidemic: a dangerous idea. Rev Soc Bras Med Trop. 2020;53: e20200481. doi:https://dx.doi.org/10.1590/0037-8682-0481-2020
9. Martinez EZ, Aragon DC, Nunes AA. Short-term forecasting of daily COVID-19 cases in Brazil by using the Holt’s model. Rev Soc Bras Med Trop. 2020;53: e20200283. doi:https://dx.doi.org/10.1590/0037-8682-0283-2020
10. Martínez L, Valencia I, Trofimoff V. Subjective wellbeing and mental health during the COVID-19 pandemic: Data from three population groups in Colombia. Data Br. 2020;32: 106287. doi:10.1016/j.dib.2020.106287
11. Martinez-Fierro ML, Rios-Jasso J, Garza-Veloz I, Reyes-Veyna L, Cerda-Luna RM, Duque-Jara I, et al. The role of close contacts of COVID-19 patients in the SARS-CoV-2 transmission: an emphasis on the percentage of nonevaluated positivity in Mexico. Am J Infect Control. 2020. doi:http://dx.doi.org/10.1016/j.ajic.2020.10.002
12. Martins CM, Gomes RZ, Muller EV, Borges PK de O, Coradassi CE, Montiel EM da S. Predictive model for covid-19 incidence in a medium-sized municipality in brazil (ponta grossa, paraná). Texto &amp; Context enferm. 2020;29: e20200154–e20200154.
13. Martins LD, da Silva I, Batista W V, Andrade MDF, Freitas EDD, Martins JA. How socio-economic and atmospheric variables impact COVID-19 and influenza outbreaks in tropical and subtropical regions of Brazil. Environ Res. 2020;191: 110184. doi:http://dx.doi.org/10.1016/j.envres.2020.110184
14. Martins-Filho PR, de Souza Araujo AA, Quintans-Junior LJ, Santos VS. COVID-19 fatality rates related to social inequality in Northeast Brazil: a neighborhood-level analysis. J Travel Med. 2020. doi:http://dx.doi.org/10.1093/jtm/taaa128
15. Martins-Filho PR, Souza Araujo AA, Quintans-Junior LJ, Santos VS. Digital public interest on coronavirus information and social distancing: a Brazilian nationwide analysis using an infodemiology approach. J Travel Med. 2020. doi:http://dx.doi.org/10.1093/jtm/taaa154
16. Maskin LP, Olarte GL, Palizas  Jr. F, Velo AE, Lurbet MF, Bonelli I, et al. High dose dexamethasone treatment for Acute Respiratory Distress Syndrome secondary to COVID-19: a structured summary of a study protocol for a randomised controlled trial. Trials. 2020;21: 743. doi:https://dx.doi.org/10.1186/s13063-020-04646-y
17. Massuda A, Tasca R, Malik AM. Use of private hospital beds by public health systems in response to COVID-19. 2020.
18. Matheus TB, Fernando ML-T, Marco Tulio PC, Ricardo D, Jose Alexandre FD-F. Social distancing and movement constraint as the most likely factors for COVID-19 outbreak control in Brazil. medRxiv. 2020. doi:10.1101/2020.05.02.20088013
19. Matos A da R, Motta FC, Caetano BC, Ogrzewalska M, Garcia CC, Lopes JCO, et al. Identification of SARS-CoV-2 and additional respiratory pathogens cases under the investigation of COVID-19 initial phase in a Brazilian reference laboratory. Mem Inst Oswaldo Cruz. 2020;115: e200232. doi:https://dx.doi.org/10.1590/0074-02760200232
20. Matos R, Akutsu R, Zandonadi RP, Rocha A, Botelho RBA. Wellbeing at Work before and during the SARS-COV-2 Pandemic: A Brazilian Nationwide Study among Dietitians. Int J Environ Res Public Health. 2020;17: 1–15. doi:10.3390/ijerph17155541
21. Mattar S, Martinez-Bravo C, Rivero R, Contreras H, Faccini_Martinez A, Guzman C, et al. Epidemiological and viral features of a cohort of SARS-CoV-2 symptomatic and asymptomatic individuals in an area of the Colombian Caribbean. ResearchSquare. 2020. doi:10.21203/rs.3.rs-57254/v3
22. Mattar S, Martinez-Bravo C, Rivero R, Contreras H, Faccini_Martinez A, Guzman C, et al. High Number of RNA Copies in Asymptomatic Individuals Infected with SARS-CoV-2 in an Area of the Colombian Caribbean. ResearchSquare. 2020. doi:10.21203/rs.3.rs-57254/v1
23. Matteo S, Higor SM, Shaojun L, Saulo DSR, Carles I, Antonio SLN, et al. Superspreading k-cores at the center of COVID-19 pandemic persistence. medRxiv. 2020. doi:10.1101/2020.08.12.20173476
24. Matthew DL, Nishanth TA, Mehak A, Sharut G, Praveer S, Brent PL, et al. Improvement and Multi-Population Generalizability of a Deep Learning-Based Chest Radiograph Severity Score for COVID-19. medRxiv. 2020. doi:10.1101/2020.09.15.20195453
25. Mayol J, Artucio C, Batista I, Puentes A, Villegas J, Quizpe R, et al. An international survey in Latin America on the practice of interventional cardiology during the COVID-19 pandemic, with a particular focus on myocardial infarction. Neth Heart J. 2020. doi:10.1007/s12471-020-01440-y
26. Mayra R-S, Yayquier D, Yordanis M, Nestor Antonio C, William S, Orlando V, et al. Use of a humanized anti-CD6 monoclonal antibody (itolizumab) in elderly patients with moderate COVID-19. medRxiv. 2020. doi:10.1101/2020.07.24.20153833
27. Mayra T-E, Jorge AE-Z, José AM-L, Manuel Adrian A-Z, Daniel O-L, Pablo AR-C, et al. Lockdown, relaxation, and ACME period in COVID-19: A study of disease dynamics on Hermosillo, Sonora, Mexico. medRxiv. 2020. doi:10.1101/2020.08.20.20178509
28. McCoy J, Cadegiani FA, Wambier CG, Herrera S, Vano-Galvan S, Mesinkovska NA, et al. 5-Alpha-Reductase Inhibitors are Associated with Reduced Frequency of COVID-19 Symptoms in Males with Androgenetic Alopecia. J Eur Acad Dermatol Venereol. 2020. doi:http://dx.doi.org/10.1111/jdv.17021
29. Mecler N, Senna T, Tholt B, Lima-Junior J, Ferreira D de C. Covid-19 and Dentistry: analysis of available information on a virtual platform. A descriptive and observational study. Rev bras odontol. 2020;77: 1–5.
30. Medina-Mendieta JF, Cortés-Cortés M, Cortés-Iglesias M. COVID-19 Forecasts for Cuba Using Logistic Regression and Gompertz Curves. MEDICC Rev. 2020;22: 32–39. doi:10.37757/MR2020.V22.N3.8
31. Mejia CR, Sancho AQ, Rodriguez-Alarcon JF, Valero LC, Ponce Lopez VL, Varela Villanueva ES, et al. Factors associated with fatalism in the face of COVID-19 in 20 Peruvian cities in March 2020. Rev Habanera Ciencias Medicas. 2020;19: e_323.
32. Mejia CR, Rodriguez-Alarcon JF, Garay-Rios L, Enriquez-Anco M de G, Moreno A, Huaytán-Rojas K, et al. Perception of exaggeration or fear propagated by the media among the Peruvian population during the COVID-19 pandemic. Rev Cuba invest bioméd. 2020;39: e698–e698.
33. Mejía-Vilet JM, Córdova-Sánchez BM, Fernández-Camargo DA, Méndez-Pérez RA, Morales-Buenrostro LE, Hernández-Gilsoul T. A risk score to predict admission to the intensive care unit in patients with Covid-19: the ABC-GOALS score. Salud Publica Mex. 2020. doi:10.21149/11684
34. Melendi SE, Pérez MM, Salas CE, Aguirre C, Baleta ML, Balsano FJ, et al. Estudio de cohorte prospectivo de pacientes con COVID-19 hospitalizados en servicio de clínica médica del Hospital Durand Protocolo de estudio. Rev argent salud publica. 2020;12: 1–6.
35. Melin P, Monica JC, Sanchez D, Castillo O. Multiple Ensemble Neural Network Models with Fuzzy Response Aggregation for Predicting COVID-19 Time Series: The Case of Mexico. Healthc (Basel, Switzerland). 2020;8. doi:https://dx.doi.org/10.3390/healthcare8020181
36. Mella-Abarca W, Barraza-Sanchez V, Ramirez-Parada K. Telerehabilitation for people with breast cancer through the COVID-19 pandemic in Chile. Ecancermedicalscience. 2020;14: 1085. doi:http://dx.doi.org/10.3332/ECANCER.2020.1085
37. Melo GC, Araújo Neto RA, Araújo K. Forecasting the rate of cumulative cases of COVID-19 infection in Northeast Brazil: a Boltzmann function-based modeling study. Cad Saude Publica. 2020;36: e00105720. doi:10.1590/0102-311X00105720
38. Melo GC, Duprat IP, Araújo K, Fischer FM, Araújo Neto RA. Prediction of cumulative rate of COVID-19 deaths in Brazil: a modeling study. Rev Bras Epidemiol. 2020;23: e200081. doi:10.1590/1980-549720200081
39. Mena RH, Velasco-Hernandez JX, Mantilla-Beniers NB, Carranco-Sapiens GA, Benet L, Boyer D, et al. Using posterior predictive distributions to analyse epidemic models: COVID-19 in Mexico City. Phys Biol. 2020;17: 65001. doi:https://dx.doi.org/10.1088/1478-3975/abb115
40. Mendez-Arriaga F. The temperature and regional climate effects on communitarian COVID-19 contagion in Mexico throughout phase 1. Sci Total Environ. 2020;735: 139560. doi:https://dx.doi.org/10.1016/j.scitotenv.2020.139560
41. Mendez-Dominguez N, Alvarez-Baeza A, Carrillo G. Demographic and Health Indicators in Correlation to Interstate Variability of Incidence, Confirmation, Hospitalization, and Lethality in Mexico: Preliminary Analysis from Imported and Community Acquired Cases during COVID-19 Outbreak. Int J Environ Res Public Health. 2020;17. doi:https://dx.doi.org/10.3390/ijerph17124281
42. Mendez-Espinosa JF, Rojas NY, Vargas J, Pachón JE, Belalcazar LC, Ramírez O. Air quality variations in Northern South America during the COVID-19 lockdown. Sci Total Environ. 2020;749: 141621. doi:10.1016/j.scitotenv.2020.141621
43. Mendieta JFM, Cortes Cortes ME, Iglesias MC. Adjustment of population growth curve applied to COVID-19 in Cuba. Rev Habanera Ciencias Medicas. 2020;19: e3353.
44. Mendonça F, Anjos M, Collischonn E, Murara P, F D, Limberger L, et al. Climate and Covid-19 - Upgrade and solar radiation influences based on Brazil cases. ResearchSquare. 2020. doi:10.21203/rs.3.rs-32885/v1
45. Mendonça FD, Rocha SS, Pinheiro DLP, Oliveira SV. North region of Brazil and the COVID-19 pandemic: socioeconomic and epidemiologic analysis. J Heal NPEPS. 2020;5: 20–37.
46. Menezes MO, Takemoto MLS, Nakamura-Pereira M, Katz L, Amorim MMR, Salgado HO, et al. Risk factors for adverse outcomes among pregnant and postpartum women with acute respiratory distress syndrome due to COVID-19 in Brazil. Int J Gynecol Obstet. 2020. doi:http://dx.doi.org/10.1002/ijgo.13407
47. Menezes TM de O, Freitas AV da S, Pedreira LC, Amaral JB do. Telemonitoring of Brazilian Nursing homes before Coronavirus and COVID-19 Infections. Rev Bras Enferm. 2020;73: e20200350. doi:https://dx.doi.org/10.1590/0034-7167-2020-0350
48. Mengana López E, Pérez Medina Y, Portuondo Kindelán D, Domínguez Redondo D, Álvarez Lambert R, Rodríguez Aguirre Y. Clinical-epidemiological characterization of pediatric patients infected with SARS-CoV-2 in Santiago de Cuba province. Rev Cuba pediatr. 2020;92: e1177–e1177.
49. Michard F, Malbrain ML, Martin GS, Fumeaux T, Lobo S, Gonzalez F, et al. Haemodynamic Monitoring and Management in COVID-19 Intensive Care Patients: An International Survey. Anaesthesia, Crit care pain Med. 2020. doi:10.1016/j.accpm.2020.08.001
50. Michel FM-R, Fernando C-L, Alejandro T-Q, Augusto R-M, Gerardo G-R, Rocio O-L, et al. Initial experience in Mexico with convalescent plasma in COVID-19 patients with severe respiratory failure, a retrospective case series. medRxiv. 2020. doi:10.1101/2020.07.14.20144469
51. Miguel Reina O, Vinita S. Modeling the COVID-19 outbreak in Ecuador: Is it the right time to lift social distancing containment measures? medRxiv. 2020. doi:10.1101/2020.05.21.20109520
52. Miguel ALN, Rafael LGR, Pedro SP, Cecilia Siliansky de A. How super-spreader cities, highways, hospital bed availability, and dengue fever influenced the COVID-19 epidemic in Brazil. medRxiv. 2020. doi:10.1101/2020.09.19.20197749
53. Miguel L. Effects of COVID-19 Early Release of Pension Funds: The Case of Chile. SSRN. 2020. doi:10.2139/ssrn.3694889
54. Miñan-Tapia A, Conde-Escobar A, Calderon-Arce D, Cáceres-Olazo D, Peña-Rios AJ, Donoso-Romero RC. Associated factors to self-medication with drugs related to COVID-19 in health science students from a peruvian city. 2020.
55. Miot HA, Ianhez M, Ramos PM. Self-reported cutaneous manifestations in 1,429 Brazilian COVID-19 patients. J Eur Acad Dermatol Venereol. 2020. doi:http://dx.doi.org/10.1111/jdv.17024
56. Mirleide C dos S, Edivaldo Costa  Jr. S, Jessylene AF, Sandro PS, Michel PCS, Jedson FC, et al. MOLECULAR EPIDEMIOLOGY TO UNDERSTAND THE SARS-CoV-2 EMERGENCE IN THE BRAZILIAN AMAZON REGION. medRxiv. 2020. doi:10.1101/2020.09.04.20184523
57. Mirza N, Hasnaoui JA, Naqvi B, Rizvi SKA. The impact of human capital efficiency on Latin American mutual funds during Covid-19 outbreak. Swiss J Econ Stat. 2020;156: 16. doi:10.1186/s41937-020-00066-6
58. Moiseis Santos C, Geraldo LD, Eveliny Barroso S. Using the infection fatality rate to predict the evolution of Covid-19 in Brazil. medRxiv. 2020. doi:10.1101/2020.07.01.20144279
59. Mondal MRH, Bharati S, Podder P. Data analytics for novel coronavirus disease. Informatics Med Unlocked. 2020;20: 100374. doi:http://dx.doi.org/10.1016/j.imu.2020.100374
60. Monica Santos de  Sr. M, Lysandro Pinto  Sr. B, Daniela Raguer Valadao  Sr. S, Aline Fagundes  Sr. M, Jose Melquiades De Rezende  Sr. N, Anderson Alves  Sr. R, et al. Anti-SARS-CoV-2 IgM and IgG antibodies in health workers in Sergipe, Brazil. medRxiv. 2020. doi:10.1101/2020.09.24.20200873
61. Montanheiro L, Dartora C. Characterizing COVID-19 epidemics dissemination and previsions for Curitiba,Brazil using a modified SIR model. 2020.
62. Montano W, Gushiken E. Lima soundscape before confinement and during curfew. Airplane flights suppressions because of Peruvian lockdown. J Acoust Soc Am. 2020;148: 1824. doi:http://dx.doi.org/10.1121/10.0002112
63. Monteiro de Oliveira M, Fuller TL, Brasil P, Gabaglia CR, Nielsen-Saines K. Controlling the COVID-19 pandemic in Brazil: a challenge of continental proportions. Nat Med. 2020;26: 1505–1506. doi:http://dx.doi.org/10.1038/s41591-020-1071-5
64. Monterrosa-Castro Á, Dávila-Ruiz R, Mejía-Mantilla A, Contreras-Saldarriaga J, Mercado-Lara M, Flores-Monterrosa C. Occupational Stress, Anxiety and Fear of COVID-19 in Colombian Physicians. MedUNAB. 2020;23: 214–232.
65. Monterrosa-Castro A, Redondo-Mendoza V, Mercado-Lara M. Psychosocial factors associated with symptoms of generalized anxiety disorder in general practitioners during the COVID-19 pandemic. J Investig Med. 2020;68: 1228–1234. doi:https://dx.doi.org/10.1136/jim-2020-001456
66. Monterrubio-Flores E, Ramírez-Villalobos M, Espinoza-Montero J, Hernandez B, Barquera S, Villalobos-Daniel V, et al. Impact of a Double Epidemic in Mexico: Non-Communicable Diseases Increase the Case Fatality Rate with Covid-19. ResearchSquare. 2020. doi:10.21203/rs.3.rs-80669/v1
67. Moquillaza-Alcántara V, Romero-Cerdán A, Munares-García O, Merellano-Navarro E. Variation in the epidemiological indicators of COVID-19 a part of the emergency policies adopted in south american countries. 2020.
68. Moraes RF de. Determinants of physical distancing during the covid-19 epidemic in Brazil: effects from mandatory rules, numbers of cases and duration of rules. Cien Saude Colet. 2020;25: 3393–3400. doi:https://dx.doi.org/10.1590/1413-81232020259.21892020
69. Mora-Magana I, Lee SA, Maldonado-Castellanos I, Jimenez-Gutierrez C, Mendez-Venegas J, Maya-Del-Moral A, et al. Coronaphobia among healthcare professionals in Mexico: A psychometric analysis. Death Stud. 2020; 1–10. doi:http://dx.doi.org/10.1080/07481187.2020.1808762
70. Moreau VH. Forecast predictions for the COVID-19 pandemic in Brazil by statistical modeling using the Weibull distribution for daily new cases and deaths. Braz J Microbiol. 2020. doi:10.1007/s42770-020-00331-z
71. Moreira RM, Villa Montoya AC, Silveria Araujo SL, Trindade RA, da Cunha Oliveira D, de Oliveira Marinho G. How prepared is Brazil to tackle the COVID-19 disease? J Glob Health. 2020;10: 20321. doi:http://dx.doi.org/10.7189/jogh.10.020321
72. Moreira R da S. COVID-19: intensive care units, mechanical ventilators, and latent mortality profiles associated with case-fatality in Brazil. Cad Saude Publica. 2020;36: e00080020. doi:https://dx.doi.org/10.1590/0102-311x00080020
73. Motta JC, Novoa DJ, Gómez CC, Moreno JM, Vargas L, Pérez J, et al. Prognostic factors in hospitalized patients diagnosed with SARS-CoV-2 infection, Bogotá, Colombia. Biomedica. 2020;40: 116–130. doi:10.7705/biomedica.5764
74. Moura DTH, Proenca IM, McCarty TR, Sagae VMT, Ribeiro IB, Oliveira GHP, et al. Gastrointestinal Manifestations and Associated Health Outcomes of COVID-19: A Brazilian Experience From the Largest South American Public Hospital. Clinics (Sao Paulo). 2020;75: e2271. doi:http://dx.doi.org/10.6061/clinics/2020/e2271
75. Moura RR, Agrelli A, Santos-Silva CA, Silva N, Assunção BR, Brandão L, et al. Immunoinformatic approach to assess SARS-CoV-2 protein S epitopes recognised by the most frequent MHC-I alleles in the Brazilian population. J Clin Pathol. 2020. doi:10.1136/jclinpath-2020-206946
76. Moura RF, Mundim-Pombo APM, Oliveira J de FM de. New Coronavirus (2019-nCoV): analysis of the magnitude in the first two months of epidemic. Nurs (Säo Paulo). 2020;23: 4311–4316.
77. Mozart Julio Tabosa S, Ligia SK, Regina Vianna B, Ivana Cristina de Holanda Cunha B, Rosa Livia Freitas A, Paulo G, et al. Fernando de Noronha: how an island controlled the community transmission of COVID-19 in Brazil. medRxiv. 2020. doi:10.1101/2020.10.22.20216010
78. Munayco C V, Tariq A, Rothenberg R, Soto-Cabezas GG, Reyes MF, Valle A, et al. Early transmission dynamics of COVID-19 in a southern hemisphere setting: Lima-Peru: February 29th-March 30th, 2020. Infect Dis Model. 2020. doi:https://dx.doi.org/10.1016/j.idm.2020.05.001

Cesar VM, Amna T, Gabriela GS-C, Mary FR, Andree V, Leonardo R-M, et al. Early transmission dynamics and control of COVID-19 in a southern hemisphere setting: Lima-Peru, February 29th-March 30th, 2020. medRxiv. 2020. doi:10.1101/2020.04.30.20077594

1. Munguía-López A, Ponce-Ortega J. Fair allocation of potential COVID-19 vaccines using an optimization-based strategy. ResearchSquare. 2020. doi:10.21203/rs.3.rs-83772/v1
2. Muñoz L, Pífano M, Bolzán A, Varela T, Comes Y, Specogna M, et al. Surveillance and Seroprevalence: Evaluation of IgG antibodies for SARS-Cov2 by ELISA in the popular neighborhood Villa Azul, Quilmes, province of Buenos Aires, Argentina. 2020.
3. Murillo-Zamora E, Guzman-Esquivel J, Sanchez-Pina RA, Cedeno-Laurent G, Delgado-Enciso I, Mendoza-Cano O. Physical distancing reduced the incidence of influenza and supports a favorable impact on SARS-CoV-2 spread in Mexico. J Infect Dev Ctries. 2020;14: 953–956. doi:http://dx.doi.org/10.3855/JIDC.13250
4. Murillo-Zamora E, Hernandez-Suarez C. Performance of the case definition of suspected influenza before and during the COVID-19 pandemic. Rev Clin Esp. 2020. doi:http://dx.doi.org/10.1016/j.rce.2020.09.001

Efren M-Z, Carlos MH-S. Performance of suspected influenza case definitionbefore and during the COVID-19 pandemic. medRxiv. 2020. doi:10.1101/2020.06.01.20119446

1. Murillo-Zamora E, Trujillo X, Huerta M, Rios-Silva M, Mendoza-Cano O. Male gender and kidney illness are associated with an increased risk of severe laboratory-confirmed coronavirus disease. BMC Infect Dis. 2020;20: 674. doi:https://dx.doi.org/10.1186/s12879-020-05408-6

Efren M-Z, Xochitl T, Miguel H, Monica R-S, Oliver M-C. Male gender and kidney illness associated with an increased risk of severe laboratory-confirmed coronavirus disease. medRxiv. 2020. doi:10.1101/2020.06.29.20142562

1. Nabi KN. Forecasting COVID-19 pandemic: A data-driven analysis. Chaos Solitons Fractals. 2020;139: 110046. doi:10.1016/j.chaos.2020.110046

Khondoker Nazmoon N. FORECASTING COVID-19 PANDEMIC: A DATA-DRIVEN ANALYSIS. medRxiv. 2020. doi:10.1101/2020.05.12.20099192

1. Nadim SS, Chattopadhyay J. Occurrence of backward bifurcation and prediction of disease transmission with imperfect lockdown: A case study on COVID-19. Chaos Solitons Fractals. 2020;140: 110163. doi:10.1016/j.chaos.2020.110163
2. Nagata Cavalheiro M, Fonseca VR, Zeigelboim BS, Costa DF, de Lima L V, Bozzo MK, et al. Evaluating the Quality of Rigid Optic Videolaryngoscopy Image Taken Through Dental Protection Cap and Its Feasibility as Additional Barrier Method Against COVID-19. J Voice. 2020. doi:10.1016/j.jvoice.2020.10.014
3. Nakada LYK, Urban RC. COVID-19 pandemic: environmental and social factors influencing the spread of SARS-CoV-2 in Sao Paulo, Brazil. Environ Sci Pollut Res Int. 2020. doi:http://dx.doi.org/10.1007/s11356-020-10930-w

Nakada L, Urban R. COVID-19 pandemic: environmental and social factors influencing the spread of SARS-CoV-2 in the expanded metropolitan area of São Paulo, Brazil. ResearchSquare. 2020. doi:10.21203/RS.3.RS-34613/V1

1. Nakada LYK, Urban RC. COVID-19 pandemic: Impacts on the air quality during the partial lockdown in Sao Paulo state, Brazil. Sci Total Environ. 2020;730: 139087. doi:https://dx.doi.org/10.1016/j.scitotenv.2020.139087
2. Namendys-Silva SA, Alvarado-Avila PE, Dominguez-Cherit G, Rivero-Sigarroa E, Sanchez-Hurtado LA, Gutierrez-Villasenor A, et al. Outcomes of patients with COVID-19 in the intensive care unit in Mexico: A multicenter observational study. Hear Lung. 2020. doi:http://dx.doi.org/10.1016/j.hrtlng.2020.10.013
3. Namendys-Silva SA, Gutierrez-Villasenor A, Romero-Gonzalez JP. Hospital mortality in mechanically ventilated COVID-19 patients in Mexico. Intensive Care Med. 2020;46: 2086–2088. doi:http://dx.doi.org/10.1007/s00134-020-06256-3
4. Nascimento Figueredo W, Santana de Macêdo TT, Porto Cardoso GM, Boa Sorte Fernandes ET. ANÁLISE BIBLIOMÉTRICA DA PRODUÇÃO BRASILEIRA SOBRE A COVID-19. Rev Baiana Enferm. 2020;34: 1–10. doi:10.18471/rbe.v34.37107
5. Nascimento BR, Brant LC, Castro ACT, Froes LE V, Ribeiro ALP, Cruz L V, et al. Impact of a large-scale telemedicine network on emergency visits and hospital admissions during the coronavirus disease 2019 pandemic in Brazil: Data from the UNIMED-BH system. J Telemed Telecare. 2020; 1357633X20969529. doi:http://dx.doi.org/10.1177/1357633X20969529
6. Nascimento MLF. A multivariate analysis on spatiotemporal evolution of Covid-19 in Brazil. Infect Dis Model. 2020;5: 670–680. doi:http://dx.doi.org/10.1016/j.idm.2020.08.012
7. Nascimento VF do, Espinosa MM, Silva MCN da, Freire NP, Terças-Trettel ACP. Impact of COVID-19 on brazilian nursing work: epidemiological aspects. Enferm foco. 2020;11: 24–31.
8. Nascimento VF do, Hattori TY, Terças-Trette ACP. Difficulties and fears of nurses facing the COVID-19 pandemic in Brazil. Humanidad med. 2020;20.
9. Nascimento VF do, Hattori TY, Trettel ACPT. Personal needs of nurses during the COVID-19 pandemic in Mato Grosso. Enferm foco. 2020;11.
10. Nascimento VA do, Corado A de LG, Nascimento FO do, Costa AKA da, Duarte DCG, Luz SLB, et al. Genomic and phylogenetic characterisation of an imported case of SARS-CoV-2 in Amazonas State, Brazil. Mem Inst Oswaldo Cruz. 2020;115: e200310. doi:https://dx.doi.org/10.1590/0074-02760200310
11. Natalia BM, Janaina W, Marina R, Mizziara De P, Juliana D, Gabriela S, et al. Dreaming during the Covid-19 pandemic: Computational assessment of dream reports reveals mental suffering associated with negative feelings and contagion fear. medRxiv. 2020. doi:10.1101/2020.05.19.20107078
12. Natan F, Eduardo LC, Igor RD, Jayne SL, Julia C, Felipe FR, et al. Longitudinal study about low back pain, mental health, and access to healthcare system during COVID-19 pandemic: protocol of an ambispective cohort. medRxiv. 2020. doi:10.1101/2020.07.22.20160309
13. Natividade MDS, Bernardes K, Pereira M, Miranda SS, Bertoldo J, Teixeira M da G, et al. Social distancing and living conditions in the pandemic COVID-19 in Salvador-Bahia, Brazil. Distanciamento Soc e condicoes vida na pandemia COVID-19 em Salvador Bras. 2020;25: 3385–3392. doi:https://dx.doi.org/10.1590/1413-81232020259.22142020
14. Navarro Machado VR, Moracén Rubio B, Santana Rodríguez D, Rodríguez González O, Oliva Santana M, Blanco González G. Active community inquiry facing COVID-19: Experiences in the Cumanayagua municipality, 2020. Medisur. 2020;18: 388–395.
15. Navarro Machado VR, Moracén Rubio B, Santana Rodríguez D, Rodríguez González O, Oliva Santana M, Blanco González G. Pesquisa activa comunitaria ante la COVID-19. Experiencias en el municipio de Cumanayagua, 2020. Medisur. 2020;18: 388–395.
16. Neftali Eduardo A-V, Omar Yaxmehen B-C, Arsenio V-V, Carlos AF-M, Alejandro M-S, Jessica Paola B-L. Health-care workers with COVID-19 living in Mexico City: clinical characterization and related outcomes. medRxiv. 2020. doi:10.1101/2020.07.02.20145169
17. Negri EM, Piloto BM, Morinaga LK, Jardim CVP, Lamy SAED, Ferreira MA, et al. Heparin Therapy Improving Hypoxia in COVID-19 Patients - A Case Series. Front Physiol. 2020;11: 573044. doi:http://dx.doi.org/10.3389/fphys.2020.573044
18. Neiva MB, Carvalho I, Costa Filho EDS, Barbosa-Junior F, Bernardi FA, Sanches TLM, et al. Brazil: the emerging epicenter of COVID-19 pandemic. Rev Soc Bras Med Trop. 2020;53: e20200550. doi:http://dx.doi.org/10.1590/0037-8682-0550-2020
19. Nepomuceno TCC, Silva WMN, Nepomuceno KTC, Barros IKF. A DEA-Based Complexity of Needs Approach for Hospital Beds Evacuation during the COVID-19 Outbreak. J Healthc Eng. 2020;2020: 8857553. doi:http://dx.doi.org/10.1155/2020/8857553
20. Nestor Gabriel I, Leopoldo German G, Juan Manuel C, Ignacio A, Emiliano R, Gabriel T, et al. SARS-CoV-2 surveillance in untreated wastewater: first detection in a low-resource community in Buenos Aires, Argentina. medRxiv. 2020. doi:10.1101/2020.10.21.20215434
21. Neto O, Reis J, Brizzi A, Zambrano G, Souza J, Pedroso W, et al. Mathematical model of COVID-19 intervention scenarios for São Paulo- Brazil. ResearchSquare. 2020. doi:10.21203/rs.3.rs-32962/v1
22. Neves AGM, Guerrero G. Predicting the evolution of the COVID-19 epidemic with the A-SIR model: Lombardy, Italy and São Paulo state, Brazil. Physica D. 2020;413: 132693. doi:10.1016/j.physd.2020.132693
23. Neylan Leal D. Predição da propagação do SARS-CoV-2 no Estado do Amapá, Amazônia, Brasil, por modelagem matemática. 2020;06. doi:10.32749/NUCLEODOCONHECIMENTO.COM.BR/SAUDE/PREDICAO-DA-PROPAGACAO
24. Nicolas A, Tiago C, Daniel Da M. More Than Words: Leaders’ Speech and Risky Behavior during a Pandemic. SSRN. 2020. doi:10.2139/ssrn.3582908
25. Nicolò G, Michele T, Matteo C, Leo F, Alessandro V, Nicola P. Estimating the effect of social inequalities in the mitigation of COVID-19 across communities in Santiago de Chile. medRxiv. 2020. doi:10.1101/2020.10.08.20204750
26. Nieto-Calvache AJ, Quintero-Santacruz M, Macia-Mejia C, Lopez-Giron MC, Vergara-Galliadi LM, Ariza F. Dangerous shortage of blood banks as an indirect effect of SARS-CoV-2: An obstetrics perspective. Int J Gynecol Obstet. 2020. doi:http://dx.doi.org/10.1002/ijgo.13409
27. Niquini RP, Lana RM, Pacheco AG, Cruz OG, Coelho FC, Carvalho LM, et al. Description and comparison of demographic characteristics and comorbidities in SARI from COVID-19, SARI from influenza, and the Brazilian general population. SRAG por COVID-19 no Bras descricao e Comp Caracter Demogr e comorbidades com SRAG por Influ e com a Popul geral. 2020;36: e00149420. doi:https://dx.doi.org/10.1590/0102-311x00149420
28. Noel Gutierrez B, Humberto Gutierrez P, Kimberlyn R, Nestor Garcia C, Jorge H-B, Jose Francisco M-V, et al. Prevention of household transmission crucial to stop the catastrophic spread of COVID-19 in cities. medRxiv. 2020. doi:10.1101/2020.06.05.20123711
29. Noronha KVM de S, Guedes GR, Turra CM, Andrade MV, Botega L, Nogueira D, et al. The COVID-19 pandemic in Brazil: analysis of supply and demand of hospital and ICU beds and mechanical ventilators under different scenarios. Cad Saude Publica. 2020;36: e00115320. doi:https://dx.doi.org/10.1590/0102-311X00115320
30. Nunes Neto GV, Bernardino da Silva JC, Gouveia Câmara Guerra MC, Lopes Oliveira DA, da Silva Cavalcante R, Santos Barbosa de Araújo JF. Fluxos assistenciais e cuidados no controle da COVID-19 de uma instituição hospitalar. Enferm Bras. 2020;19: 6–12. doi:10.33233/eb.v19i4.4089
31. Oblitas González Correo A, Sempertegui Sánchez Correo N. Ansiedad en tiempos de aislamiento social por COVID-19. Chota, Perú, 2020. Av en Enferm. 2020;38: 11–21. doi:10.15446/av.enferm.v38n2.87589
32. Ochoa-Rosales C, González-Jaramillo N, Vera-Calzaretta A, Franco OH. Impact of mitigation measures on the COVID-19 pandemic in Chile: preliminary data for the period April 14 to May 14. Rev salud pública. 2020;22: e386380–e386380.
33. Oilson Alberto  Jr. G, Diego Carvalho do N, Cibele Maria R, Marcos Jardel H, Caio Paziani T, Maristela Oliveira S, et al. Safety Stock: Predicting demand on the supply chain in Brazilian hospitals during the COVID-19 pandemic. medRxiv. 2020. doi:10.1101/2020.05.27.20114330
34. Olivar-Lopez V, Leyva-Barrera A, Lopez-Martinez B, Parra-Ortega I, Marquez-Gonzalez H. Clinical risk profile associated with SARS-CoV-2 infection and complications in the emergency area of a pediatric COVID-19 center. Bol Med Hosp Infant Mex. 2020;77: 221–227. doi:http://dx.doi.org/10.24875/BMHIM.20000198
35. Oliveira LM, Zanatta FB. Self-reported dental treatment needs during the COVID-19 outbreak in Brazil: an infodemiological study. Braz Oral Res. 2020;34: e114. doi:https://dx.doi.org/10.1590/1807-3107bor-2020.vol34.0114
36. Oliveira MHS de, Wong J, Lippi G, Henry BM. Analysis of clinical and demographic heterogeneity of patients dying from COVID-19 in Brazil versus China and Italy. Braz J Infect Dis. 2020. doi:https://dx.doi.org/10.1016/j.bjid.2020.05.002
37. Oliveira SB de, Ganem F, Araujo WN de, Casabona J, Sanchez MN, Croda J. Imputation method to reduce undetected severe acute respiratory infection cases during the coronavirus disease outbreak in Brazil. Rev Soc Bras Med Trop. 2020;53: e20200528. doi:https://dx.doi.org/10.1590/0037-8682-0528-2020
38. Olivier B, Ulugbek A. Between a Rock and a Hard Place: Poverty and Covid-19 in Developing Countries. SSRN. 2020. doi:10.2139/ssrn.3614245
39. Omar V, Sebastian EI, Maria P, Patricia R, Mabel A, Gigia R, et al. First report of tocilizumab use in a cohort of Latin American patients hospitalized for severe COVID-19 pneumonia. medRxiv. 2020. doi:10.1101/2020.08.12.20173104
40. Orellana JDY, Cunha GM da, Marrero L, Horta BL, Leite I da C. Explosion in mortality in the Amazonian epicenter of the COVID-19 epidemic 19. Explosao da mortalidade no Epic Amaz da epidemia COVID-19. 2020;36: e00120020. doi:https://dx.doi.org/10.1590/0102-311x00120020
41. Orison OW, Richard NB. Mortality Attributed to COVID-19 in High-Altitude Populations. medRxiv. 2020. doi:10.1101/2020.06.10.20128025
42. Ornelas-Aguirre JM. El nuevo coronavirus que llego de Oriente: analisis de la epidemia inicial en Mexico, The new coronavirus that came from the East: analysis of the initial epidemic in Mexico. Gac Med Mex. 2020;156: 208–216. doi:http://dx.doi.org/10.24875/GMM.M20000377

José MO-A. El nuevo coronavirus que llegó de Oriente: análisis de la epidemia inicial en México. Gac Med Mex. 2020;156. doi:10.24875/GMM.20000165

1. Ortega-Lenis D, Arango-Londoño D, Muñoz E, Cuartas DE, Caicedo D, Mena J, et al. Predicciones de un modelo SEIR para casos de COVID-19 en Cali, Colombia. Rev salud pública. 2020;22: e286432–e286432.
2. Ortiz Z, Antonietti L, Capriati A, Ramos S, Romero M, Mariani J, et al. [Concerns and demands regarding COVID-19. Survey of health personnel]. Preocupaciones y demandas frente a COVID-19 Encuesta al Pers salud. 2020;80 Suppl 3: 16–24.
3. Ortiz-Brizuela E, Villanueva-Reza M, Gonzalez-Lara MF, Tamez-Torres KM, Roman-Montes CM, Diaz-Mejia BA, et al. CLINICAL AND EPIDEMIOLOGICAL CHARACTERISTICS OF PATIENTS DIAGNOSED WITH COVID-19 IN A TERTIARY CARE CENTER IN MEXICO CITY: A PROSPECTIVE COHORT STUDY. Rev Invest Clin. 2020;72: 165–177. doi:https://dx.doi.org/10.24875/RIC.20000211
4. Ortiz-Hernández L, Pérez-Sastré MA. [Social inequalities in the progression of COVID-19 in the Mexican population]. Rev Panam Salud Publica. 2020;44: e106. doi:10.26633/RPSP.2020.106
5. Ortiz-Martinez Y, Garcia-Robled JE, Vasquez-Castaneda DL, Bonilla-Aldana DK, Rodriguez-Morales AJ. Can Google R trends predict COVID-19 incidence and help preparedness? The situation in Colombia. Travel Med Infect Dis. 2020; 101703. doi:https://dx.doi.org/10.1016/j.tmaid.2020.101703
6. Oscar San Roman O, Santiago Agraz O, Isidro AGA, Vasiliki R. The impact of mobility restriction measures on the reproduction index of Covid-19 in the city of Queretaro, Mexico. medRxiv. 2020. doi:10.1101/2020.09.06.20189373
7. Oscar E-M, Margot V-A, Fernando D, Solis-Sanchez GI, Italo G, Luis P-E, et al. Reverse Transcription-Loop-Mediated Isothermal Amplification (RT-LAMP) is an effective alternative for SARS-CoV-2 molecular detection in middle-income countries. medRxiv. 2020. doi:10.1101/2020.10.14.20212977
8. Osmar Pinto N, Josa Clark R, Ana Carolina Brisola B, Gustavo Jose Z, Joabe Marcos de S, Wellington Pedroso EA, et al. COVID-19 mathematical model reopening scenarios for Sao Paulo - Brazil. medRxiv. 2020. doi:10.1101/2020.04.26.20081208
9. Pablo  Sr. C, Helmer  Sr. Z, Isabel H, Alberto  Sr. A, Maria Cristina L, Socrates  Sr. H. COVID-19 trends in Colombian regions with the highest disease burden. medRxiv. 2020. doi:10.1101/2020.10.09.20210187
10. Pablo Villalobos D, Claudio C, Felipe de la F, Matilde M. COVID-19 incidence and mortality in the Metropolitan Region, Chile: time, space, and structural factors. medRxiv. 2020. doi:10.1101/2020.09.15.20194951
11. Pablo G-R, Mireya V-C, Graciela T, Rafael P-E. Measurement lessons of a repeated cross-sectional household food insecurity survey during the COVID-19 pandemic in Mexico. medRxiv. 2020. doi:10.1101/2020.08.04.20167650
12. Pacheco H, Díaz-López S, Jarre E, Pacheco H, Méndez W, Zamora-Ledezma E. NO2 levels after the COVID-19 lockdown in Ecuador: A trade-off between environment and human health. Urban Clim. 2020;34: 100674. doi:10.1016/j.uclim.2020.100674
13. Pachiega J, Afonso A, Sinhorin GT, Alencar BT, Araújo M, Longhi FG, et al. Chronic heart diseases as the most prevalent comorbidities among deaths by COVID-19 in Brazil. Rev Inst Med Trop Sao Paulo. 2020;62: e45. doi:10.1590/S1678-9946202062045
14. Padilla-Rojas C, Lope-Pari P, Vega-Chozo K, Balbuena-Torres J, Caceres-Rey O, Bailon-Calderon H, et al. Near-Complete Genome Sequence of a 2019 Novel Coronavirus (SARS-CoV-2) Strain Causing a COVID-19 Case in Peru. Microbiol Resour Announc. 2020;9. doi:https://dx.doi.org/10.1128/MRA.00303-20
15. Padrão EM, Valente FS, Besen BA, Rahhal H, Mesquita PS, de Alencar JC, et al. Awake prone positioning in COVID-19 hypoxemic respiratory failure: exploratory findings in a single-center retrospective cohort study. Acad Emerg Med. 2020. doi:10.1111/acem.14160
16. Paes de Almeida Ferreira Braga D, Setti AS, Iaconelli A, Borges E. MOTHERHOOD PLAN: HAS IT CHANGED IN FACE OF THE COVID-19 PANDEMICS? Fertil Steril. 2020;114: e181. doi:http://dx.doi.org/10.1016/j.fertnstert.2020.08.515
17. Paesano N, Santomil F, Tobia I. Impact of COVID-19 Pandemic on Ibero-American Urology Residents: Perspective of American Confederation of Urology (CAU). Int Braz J Urol. 2020;46. doi:https://dx.doi.org/10.1590/S1677-5538.IBJU.2020.S120
18. Pagotto VPF, Abbas L, Goldenberg DC, Lobato RC, do Nascimento BB, Monteiro GGR, et al. The impact of COVID-19 on the plastic surgery activity in a high-complexity university hospital in Brazil: the importance of reconstructive plastic surgery during the pandemic. Eur J Plast Surg. 2020. doi:http://dx.doi.org/10.1007/s00238-020-01729-6
19. Paixão B, Pedroso M, Baroni L, Salles R, Escobar L, Sousa C, et al. Estimation of COVID-19 under-reporting in the Brazilian States through SARI. ResearchSquare. 2020. doi:10.21203/rs.3.rs-50075/v1
20. Palacios R, Patino EG, de Oliveira Piorelli R, Conde MTRP, Batista AP, Zeng G, et al. Double-Blind, Randomized, Placebo-Controlled Phase III Clinical Trial to Evaluate the Efficacy and Safety of treating Healthcare Professionals with the Adsorbed COVID-19 (Inactivated) Vaccine Manufactured by Sinovac - PROFISCOV: A structured summary of a . Trials. 2020;21: 853. doi:http://dx.doi.org/10.1186/s13063-020-04775-4
21. Palamim CVC, Marson FAL. COVID-19 - The Availability of ICU Beds in Brazil during the Onset of Pandemic. Ann Glob Heal. 2020;86: 100. doi:https://dx.doi.org/10.5334/aogh.3025
22. Pan American Health O. Alcohol Use during the COVID-19 Pandemic in Latin America and the Caribbean, 8 September 2020. 2020.
23. Pan American Health O. Summary of the Status of National Immunization Programs during the COVID-19 Pandemic, July 2020. 2020.
24. Paola Cristina R, Edson D, Tiago G, Daiana M, Fernando CM, Luciana A, et al. Genomic surveillance of SARS-CoV-2 reveals community transmission of a major lineage during the early pandemic phase in Brazil. bioRxiv. 2020. doi:10.1101/2020.06.17.158006
25. Paroli M, Sirinian MI. Predicting SARS-CoV-2 infection trend using technical analysis indicators. Disaster Med Public Health Prep. 2020; 1–9. doi:10.1017/dmp.2020.254
26. Parra-Bracamonte GM, Lopez-Villalobos N, Parra-Bracamonte FE. Clinical characteristics and risk factors for mortality of patients with COVID-19 in a large dataset from Mexico. Ann Epidemiol. 2020. doi:10.1016/j.annepidem.2020.08.005
27. Parra-Saavedra M, Villa-Villa I, Pérez-Olivo J, Guzman-Polania L, Galvis-Centurion P, Cumplido-Romero Á, et al. Attitudes and collateral psychological effects of COVID-19 in pregnant women in Colombia. Int J Gynaecol Obstet. 2020. doi:10.1002/ijgo.13348
28. Parro V, Lafetá M, Ipolito F, Toporcov T. Predicting COVID-19 in very large countries: the case of Brazil. ResearchSquare. 2020. doi:10.21203/rs.3.rs-79708/v1
29. Passos L, Prazeres F, Teixeira A, Martins C. Impact on Mental Health Due to COVID-19 Pandemic: Cross-Sectional Study in Portugal and Brazil. Int J Environ Res Public Health. 2020;17. doi:https://dx.doi.org/10.3390/ijerph17186794
30. Patricia T, Fernanda MA-C, Renata K, Marina AMS, Matheus MT, Bianca QRCA, et al. Medical students perceptions and motivations in time of COVID-19 pandemic. medRxiv. 2020. doi:10.1101/2020.05.28.20115956
31. Patricio S, Hiram C. COVID-19 Fatality and Comorbidity Risk Factors among Confirmed Patients in Mexico. medRxiv. 2020. doi:10.1101/2020.04.21.20074591
32. Patricio V, Sebastian A, Eugenio EV, Sigismund K. Rapid characterization of the propagation of COVID-19 in different countries. medRxiv. 2020. doi:10.1101/2020.06.09.20126631
33. Patrick EB, Zoë RG, Luis Ernesto S, Gabriel Aguirre M, Leslie N, Peter SR, et al. Mortality from COVID in Colombia and Peru: Analyses of Mortality Data and Statistical Forecasts. medRxiv. 2020. doi:10.1101/2020.08.24.20181016
34. Patrick H. Estimating the Impact of COVID-19 on the Individual Lifespan:A Conceptual Detour and an Empirical Shortcut. medRxiv. 2020. doi:10.1101/2020.08.09.20171264
35. Paulino-Ramirez R, Baez AA, Vallejo Degaudenzi A, Tapia L. Seroprevalence of Specific Antibodies against SARS-CoV-2 from Hotspot Communities in the Dominican Republic. Am J Trop Med Hyg. 2020. doi:http://dx.doi.org/10.4269/ajtmh.20-0907
36. Paulo Cardoso Lins F, Jaciel Leandro de Melo F, Millena Mirella Silva de A, Thuanny Silva de M, Andressa Kelly Alves F, Maria Cecilia Freire de M, et al. Predicting social distancing index during COVID-19 outbreak through online search engines trends. medRxiv. 2020. doi:10.1101/2020.05.28.20115816
37. Paulo Cardoso Lins F, Thuanny Silva de M, Andressa Kelly Alves F, Maria Cecilia Freire de M, Millena Mirella Silva de A, Jaciel Leandro de Melo F, et al. Assessing the quality, readability and reliability of online information on COVID-19: aninfoveillance observational study. medRxiv. 2020. doi:10.1101/2020.05.30.20117614
38. Paulo JSS, Tiago P, Luis Gustavo N. Robot dance: a city-wise automatic control of Covid-19 mitigation levels. medRxiv. 2020. doi:10.1101/2020.05.11.20098541
39. Paz C, Mascialino G, Adana-Díaz L, Rodríguez-Lorenzana A, Simbaña-Rivera K, Gómez-Barreno L, et al. Anxiety and depression in patients with confirmed and suspected COVID-19 in Ecuador. Psychiatry Clin Neurosci. 2020. doi:10.1111/pcn.13106
40. Paz C, Mascialino G, Adana-Diaz L, Rodriguez-Lorenzana A, Simbana-Rivera K, Gomez-Barreno L, et al. Behavioral and sociodemographic predictors of anxiety and depression in patients under epidemiological surveillance for COVID-19 in Ecuador. PLoS One. 2020;15: e0240008. doi:https://dx.doi.org/10.1371/journal.pone.0240008
41. Pedro de Lemos M, David MG, Vitor EV. BRAZIL IS PROJECTED TO BE THE NEXT GLOBAL COVID-19 PANDEMIC EPICENTER. medRxiv. 2020. doi:10.1101/2020.04.28.20083675
42. Pedro Emanuel F, Jorge AP, Mario IS, Carlos V, Ruben OC, Alejandro JK, et al. Understanding the value of clinical symptoms of COVID-19. A logistic regression model. medRxiv. 2020. doi:10.1101/2020.10.07.20207019
43. Pedro Henrique dos Santos S, Sara Sabrina Vieira C, Lorena Sousa S, Franciele Basso Fernandes S. Déficit e ocupação de leitos de unidade de terapia intensiva adulto do Sistema Único de Saúde no estado do Piauí sob a ótica da COVID-19. 2020.
44. Pedro CH, Fernando PH, Bernardo Lessa H, Mariangela FS, Claudio JS, Luís Paulo V, et al. Remarkable Variability in SARS-CoV-2 Antibodies across Brazilian Regions: Report on Two Successive Nationwide Serological Household Surveys. SSRN. 2020. doi:10.2139/ssrn.3640428

Pedro H, Fernando H, Bernardo H, Gabriel DV, Mariangela S, Claudio S, et al. Remarkable variability in SARS-CoV-2 antibodies across Brazilian regions: nationwide serological household survey in 27 states. medRxiv. 2020. doi:10.1101/2020.05.30.20117531

1. Pedro MCLP, Marcelo AS, Pedro VS. COVID-19 DYNAMICS CONSIDERING THE INFLUENCE OF HOSPITAL INFRASTRUCTURE: AN INVESTIGATION OF BRAZILIAN SCENARIOS. medRxiv. 2020. doi:10.1101/2020.06.03.20121608
2. Pedro SP, Diego RM, Cláudia MP, Lucas Q, Rafael G, Afonso D, et al. Potential dissemination of epidemics based on Brazilian mobile geolocation data. Part I: Population dynamics and future spreading of infection in the states of Sao Paulo and Rio de Janeiro during the pandemic of COVID-19. medRxiv. 2020. doi:10.1101/2020.04.07.20056739
3. Pedrosa NL, Albuquerque NLS de. Spatial Analysis of COVID-19 cases and intensive care beds in the State of Ceara, Brazil. Cien Saude Colet. 2020;25: 2461–2468. doi:https://dx.doi.org/10.1590/1413-81232020256.1.10952020
4. Pedrozo-Pupo JC, Campo-Arias A. Depression, perceived stress related to COVID, post-traumatic stress, and insomnia among asthma and COPD patients during the COVID-19 pandemic. Chron Respir Dis. 2020;17. doi:http://dx.doi.org/10.1177/1479973120962800
5. Pedrozo-Pupo JC, Pedrozo-Cortes MJ, Campo-Arias A. Perceived stress associated with COVID-19 epidemic in Colombia: an online survey. Cad Saude Publica. 2020;36: e00090520. doi:https://dx.doi.org/10.1590/0102-311x00090520
6. Peixoto PS, Marcondes D, Peixoto C, Oliva SM. Modeling future spread of infections via mobile geolocation data and population dynamics. An application to COVID-19 in Brazil. PLoS One. 2020;15: e0235732. doi:10.1371/journal.pone.0235732
7. Pelayo-Nieto M, Linden-Castro E, Gomez-Alvarado MO, Bravo-Castro EI, Rodriguez-Covarrubias FT. Has the COVID-19 pandemic impacted the practice of urology in Mexico? Rev Mex Urol. 2020;80.
8. Peloso RM, Ferruzzi F, Mori AA, Camacho DP, Franzin LC da S, Margioto Teston AP, et al. Notes from the Field: Concerns of Health-Related Higher Education Students in Brazil Pertaining to Distance Learning During the Coronavirus Pandemic. Eval Health Prof. 2020;43: 201–203. doi:https://dx.doi.org/10.1177/0163278720939302
9. Peña E, Amin S, Bustos V, Muñoz S, Martinez E, Gomez J, et al. Experience of Clinical Screening for Elective Orthopedic Surgery in The Midst of The COVID-19 Pandemic: An Alternative Proposal. ResearchSquare. 2020. doi:10.21203/rs.3.rs-91961/v1
10. Pena VH, Espinosa A. Predictive modeling to estimate the demand for intensive care hospital beds nationwide in the context of the COVID-19 pandemic. Medwave. 2020;20: e8039. doi:http://dx.doi.org/10.5867/medwave.2020.09.8039
11. Pequeno P, Mendel B, Rosa C, Bosholn M, Souza JL, Baccaro F, et al. Air transportation, population density and temperature predict the spread of COVID-19 in Brazil. PeerJ. 2020;2020: e9322. doi:http://dx.doi.org/10.7717/peerj.9322
12. Peralta EA, Taveras M. Effectiveness of teleconsultation use in access to mental health services during the coronavirus disease 2019 pandemic in the Dominican Republic. Indian J Psychiatry. 2020;62: S492–S494. doi:http://dx.doi.org/10.4103/psychiatry.IndianJPsychiatry_1047_20
13. Peralta O, Ortínez-Alvarez A, Torres-Jardón R, Suárez-Lastra M, Castro T, Ruíz-Suárez LG. Ozone over Mexico City during the COVID-19 pandemic. Sci Total Environ. 2020; 143183. doi:10.1016/j.scitotenv.2020.143183
14. Percy  Sr. S-B, Carlos C, Yamilee H-R, Roger VA-C. Real-World Effectiveness of hydroxychloroquine, azithromycin, and ivermectin among hospitalized COVID-19 patients: Results of a target trial emulation using observational data from a nationwide Healthcare System in Peru. medRxiv. 2020. doi:10.1101/2020.10.06.20208066
15. Pereda R, González D, Rivero HB, Rivero JC, Pérez A, López LDR, et al. Therapeutic Effectiveness of Interferon-α2b Against COVID-19: The Cuban Experience. J Interferon Cytokine Res. 2020;40: 438–442. doi:10.1089/jir.2020.0124
16. Pereira LA, Mansano Soares LC, Nascimento PA, Netto Cirillo LR, Sakuma HT, Da Veiga GL, et al. Retinal findings in hospitalised patients with severe COVID-19. Br J Ophthalmol. 2020; 317576. doi:http://dx.doi.org/10.1136/bjophthalmol-2020-317576
17. Pereira-Ávila F, Botelho E, Góes F, Gir E, Sousa L, Caldeira N, et al. Public Utilization of Face Masks in Brazil During COVID-19 Pandemic Outbreak: Temporal Trend Analysis. ResearchSquare. 2020. doi:10.21203/rs.3.rs-48087/v1
18. Pereira-Avila FMV, Lam SC, Goes FGB, Gir E, Pereira-Caldeira NMV, Teles SA, et al. Factors associated with the use and reuse of face masks among Brazilian individuals during the COVID-19 pandemic. Rev Lat Am Enfermagem. 2020;28: e3360. doi:https://dx.doi.org/10.1590/1518-8345.4604.3360
19. Pérez Milian JM, Berrio Aguila JE, Ojeda YP, Rodríguez Quesada NY, Fernández Pérez SE, Aranguren Torriente D. Evaluación de la satisfacción de pacientes atendidos por la COVID-19 en el Hospital Universitario Clínico Quirúrgico Cmdte. Manuel Fajardo Rivero. Medicentro (Villa Clara). 2020;24: 630–641.
20. Pérez Milian JM, Berrio Aguila JE, Ojeda YP, Rodríguez Quesada NY, Fernández Pérez SE, Aranguren Torriente D. Evaluation of satisfaction of patients seen at &#8223; Cmdte. Manuel Fajardo Rivero" Clinico -Surgical University Hospital due to COVID-19. Medicentro (Villa Clara). 2020;24: 630–641.
21. Perez-Cano HJ, Moreno-Murguia MB, Morales-Lopez O, Crow-Buchanan O, English JA, Lozano-Alcazar J, et al. Anxiety, depression, and stress in response to the coronavirus disease-19 pandemic. Cir y Cir (English Ed. 2020;88: 562–568. doi:http://dx.doi.org/10.24875/CIRU.20000561
22. Pescarini J, Silveira I, Souza-Filho J, Aquino R, Barreto M, Aquino E. Covid-19 in Latin America countries: Course of the pandemic and the different responses towards control. ResearchSquare. 2020. doi:10.21203/rs.3.rs-56504/v1
23. Picon R V, Carreno I, da Silva AA, Mossmann M, Laste G, Domingues GDC, et al. Coronavirus disease 2019 population-based prevalence, risk factors, hospitalization, and fatality rates in southern Brazil. Int J Infect Dis. 2020;100: 402–410. doi:http://dx.doi.org/10.1016/j.ijid.2020.09.028
24. Pierrotti LC, Reusing Junior JO, Freire MP, Barros Machado DJ, Megale Moreira R, Ventura CG, et al. COVID-19 among kidney transplant recipients requiring hospitalization: preliminary data and outcomes from a single-center in Brazil. Transpl Int. 2020. doi:http://dx.doi.org/10.1111/tri.13745
25. Pimentel FF, Borba JMC, Rodrigues TCGF, Peruchi KPI, Carrara HHA, de Andrade JM, et al. Chemotherapy prescriptions and emergency medical care utilization by patients in neo/adjuvant breast cancer treatment during COVID-19 pandemic: Patterns in a Brazilian University Hospital. Ann Oncol. 2020;31: S1020. doi:http://dx.doi.org/10.1016/j.annonc.2020.08.1811
26. Pineda-Sic RA, Galarza-Delgado DA, Serna-Pena G, Castillo-Torres SA, Flores-Alvarado DE, Esquivel-Valerio JA, et al. Treatment adherence behaviours in rheumatic diseases during COVID-19 pandemic: a Latin American experience. Ann Rheum Dis. 2020. doi:https://dx.doi.org/10.1136/annrheumdis-2020-218198
27. Pintado JF, Gibaja W, Vallejos RA, Rosas W, Guerra-Farfan E, Nunez JH. How COVID-19 has affected emergent visits to a Latin-American trauma department: Experience at a Peruvian national trauma referral center. Injury. 2020. doi:http://dx.doi.org/10.1016/j.injury.2020.11.005
28. Pinto Neto O, Pinto I, Pinto O. Lightning during the COVID-19 pandemic in Brazil. J Atmos solar-terrestrial Phys. 2020;211: 105463. doi:10.1016/j.jastp.2020.105463
29. Pinto A de S, Santos Junior EG Dos, Rodrigues CA, Nunes PCM, Cruz LA da, Costa MGR, et al. Covid-19 growth rate analysis: application of a low-complexity tool for understanding and comparing epidemic curves. Rev Soc Bras Med Trop. 2020;53: e20200331. doi:https://dx.doi.org/10.1590/0037-8682-0331-2020
30. Pinzón M, Arango D, Betancur J, Holguín H, Arias C, Muñoz B, et al. Clinical Outcome of Patients with COVID-19 Pneumonia Treated with Corticosteroids and Colchicine in Colombia. ResearchSquare. 2020. doi:10.21203/rs.3.rs-94922/v1
31. Pio-Abreu A, do Nascimento MM, Vieira MA, de Menezes Neves PDM, Lugon JR, Sesso R. High mortality of CKD patients on hemodialysis with Covid-19 in Brazil. J Nephrol. 2020. doi:10.1007/s40620-020-00823-z
32. Pitts WJ, Inkpen CS. Assessing the effects of covid-19 in prisons in the northern triangle of central america. Vict Offender. 2020. doi:10.1080/15564886.2020.1828211
33. Platt VB, Guedert JM, Coelho EBS. VIOLENCE AGAINST CHILDREN AND ADOLESCENTS: NOTIFICATION AND ALERT IN TIMES OF PANDEMIC. Rev Paul Pediatr. 2020;39: e2020267. doi:http://dx.doi.org/10.1590/1984-0462/2021/39/2020267
34. Plotnikow GA, Matesa A, Nadur JM, Alonso M, Nunez II, Vergara G, et al. Characteristics and outcomes of patients infected with nCoV19 requiring invasive mechanical ventilation in Argentina. Rev Bras Ter intensiva. 2020;32: 348–353. doi:http://dx.doi.org/10.5935/0103-507X.20200062
35. Plotnikow GA, Matesa A, Nadur JM, Alonso M, Nuñez I I, Vergara G, et al. Características y resultados de los pacientes infectados con nCoV19 con requerimiento de ventilación mecánica invasiva en la Argentina. Rev Bras Ter intensiva. 2020;32: 348–353. doi:10.5935/0103-507x.20200062
36. Poblete Umanzor R, Saldías Peñafiel F, Sabatini Ugarte N, Vite Valverde A, Ceriani Bravo A, Schaffeld Pernas S, et al. Infección respiratoria aguda por coronavirus Sars-CoV-2 en personal de salud. Implementación de un programa de detección precoz y seguimiento de casos en un hospital universitario. Rev Med Chil. 2020;148: 724–733.
37. Poterico JA, Mestanza O. Genetic variants and source of introduction of SARS-CoV-2 in South America. J Med Virol. 2020. doi:https://dx.doi.org/10.1002/jmv.26001
38. Prado MFD, Antunes BBP, Bastos L, Peres IT, Silva A, Dantas LF, et al. Analysis of COVID-19 under-reporting in Brazil. Rev Bras Ter intensiva. 2020;32: 224–228. doi:10.5935/0103-507X.20200030
39. Prado T, Fumian TM, Mannarino CF, Maranhão AG, Siqueira MM, Miagostovich MP. Preliminary results of SARS-CoV-2 detection in sewerage system in Niterói municipality, Rio de Janeiro, Brazil. Mem Inst Oswaldo Cruz. 2020;115. doi:10.1590/0074-02760200196
40. Prado-Galbarro FJ, Sanchez-Piedra C, Gamino-Arroyo AE, Cruz-Cruz C. Determinants of survival after severe acute respiratory syndrome coronavirus 2 infection in Mexican outpatients and hospitalised patients. Public Health. 2020;189: 66–72. doi:http://dx.doi.org/10.1016/j.puhe.2020.09.014
41. Prado-Gascó V, Gómez-Domínguez MT, Soto-Rubio A, Díaz-Rodríguez L, Navarro-Mateu D. Stay at Home and Teach: A Comparative Study of Psychosocial Risks Between Spain and Mexico During the Pandemic. Front Psychol. 2020;11: 566900. doi:10.3389/fpsyg.2020.566900
42. Prata DN, Rodrigues W, Bermejo PH. Temperature significantly changes COVID-19 transmission in (sub)tropical cities of Brazil. Sci Total Environ. 2020;729: 138862. doi:https://dx.doi.org/10.1016/j.scitotenv.2020.138862
43. Prata-Barbosa A, Lima-Setta F, Santos GRD, Lanziotti VS, de Castro RE V, de Souza DC, et al. Pediatric patients with COVID-19 admitted to intensive care units in Brazil: a prospective multicenter study. J Pediatr (Rio J). 2020. doi:10.1016/j.jped.2020.07.002
44. Priolo Filho SR, Goldfarb D, Zibetti MR, Aznar-Blefari C. Brazilian Child Protection Professionals’ Resilient Behavior during the COVID-19 Pandemic. Child Abus Negl. 2020; 104701. doi:http://dx.doi.org/10.1016/j.chiabu.2020.104701
45. Pulido L, Solis-Aramayo MA, Ibarrola M, Heres M, Falco J, Tomaszuk G, et al. Initial experience in the attention of patients with COVID-19 in a private third-level hospital in Buenos Aires City. Medicina (B Aires). 2020;80: 433–438.
46. Quevedo-Ramírez A, Al-Kassab-Córdova A, Mendez-Guerra C, Cornejo-Venegas G, Alva-Chavez KP. Altitude and excess mortality during COVID-19 pandemic in Peru. Respir Physiol Neurobiol. 2020;281: 103512. doi:10.1016/j.resp.2020.103512
47. Quintana-Salcedo A, Vargas-Ortiz L, Severiche-Sierra C, Castro-Bocanegra V, Garcia-Moreno A, Vidal-Tovar C, et al. Knowledge and attitudes of health workers for the care of patients covid-19 in some cities of the Colombian Caribbean. Indian J Public Heal Res Dev. 2020;11: 219–229.
48. Rafael Gonzalez G, Francisco M, Pablo SM, Miguel K. Is a COVID19 Quarantine Justified in Chile or USA Right Now? medRxiv. 2020. doi:10.1101/2020.03.23.20042002
49. Rafael Marques Da S. Modeling the COVID-19 dissemination in the South Region of Brazil and testing gradual mitigation strategies. medRxiv. 2020. doi:10.1101/2020.07.02.20145136
50. Rafael Marques Da S, Carlos Fabio de Oliveira M, Cesar M. Scrutinizing the heterogeneous spreading of COVID-19 outbreak in Brazilian territory. medRxiv. 2020. doi:10.1101/2020.06.05.20123604
51. Rafael Santos Rodrigues V, Erisson Linhares de A, Helmar Abreu Rocha V, Nara Michelle de Araujo E, Marcelo O, Sergio Antonio Bastos S. Management and Clinical Characteristics in Children with SARS-CoV-2 Infection: Experience in a highly complex public hospital in the city of Sao Paulo. medRxiv. 2020. doi:10.1101/2020.06.22.20136994
52. Rafael HMP, Carlos Kaue Vieira B, Luciana Mendes S, Bernardo S, Pedro A, Nelson G, et al. Geographic access to COVID-19 healthcare in Brazil using a balanced float catchment area approach. medRxiv. 2020. doi:10.1101/2020.07.17.20156505
53. Rafael RM, Marcos BC, Ana BQ, Andrea D, Joao PL, Tatiana P-C, et al. COVID-19 challenges to dentistry in the new pandemic epicenter: Brazil. medRxiv. 2020. doi:10.1101/2020.06.11.20128744
54. Rafael R de MR, Neto M, Depret DG, Gil AC, Fonseca MHS, Souza-Santos R. Effect of income on the cumulative incidence of COVID-19: an ecological study. Rev latinoam enferm. 2020;28: e3344–e3344.
55. Rahul Kalippurayil M. Role of Weather Factors in COVID-19 Deaths in Tropical Climate: A Data-Driven Study Focused on Brazil. medRxiv. 2020. doi:10.1101/2020.09.13.20193532
56. Raigam Jafet M-P, Alexadros S, Johnatan T-T, Charzakis C, Ameth H-V, Jose Rafael V-B, et al. Risk factors for mortality in pregnant women with SARS-CoV-2 infection. medRxiv. 2020. doi:10.1101/2020.05.31.20107276
57. Rainisch G, Undurraga EA, Chowell G. A dynamic modeling tool for estimating healthcare demand from the COVID19 epidemic and evaluating population-wide interventions. Int J Infect Dis. 2020;96: 376–383. doi:https://dx.doi.org/10.1016/j.ijid.2020.05.043
58. Rajneesh B. A Predictive Model for the Evolution of COVID-19. medRxiv. 2020. doi:10.1101/2020.04.13.20063271
59. Ramirez-Sandoval JC, Gaytan-Arocha JE, Xolalpa-Chavez P, Mejia-Vilet JM, Arvizu-Hernandez M, Rivero-Sigarroa E, et al. Prolonged Intermittent Renal Replacement Therapy for Acute Kidney Injury in COVID-19 Patients with Acute Respiratory Distress Syndrome. Blood Purif. 2020. doi:http://dx.doi.org/10.1159/000510996
60. Ramsingh RAE, Duval JL, Rahaman NC, Rampersad RD, Angelini GD, Teodori G. Adult cardiac surgery in Trinidad and Tobago during the COVID-19 pandemic: Lessons from a developing country. J Card Surg. 2020. doi:http://dx.doi.org/10.1111/jocs.14975
61. Raphael B, Alexsandros C, Luis M. Filling the Void? Organized Crime and COVID-19 in Rio De Janeiro. SSRN. 2020. doi:10.2139/ssrn.3678840
62. Raphael B, Sergio F, Michael F, Luis M. Racial Inequality in Health Care During a Pandemic. SSRN. 2020. doi:10.2139/ssrn.3691313
63. Rasa Z, Renne V, Daniel B, Adrian B, Danilo M, Rafael Z, et al. Drastic Improvements in Air Quality in Ecuador during the COVID-19 Outbreak. Aerosol Air Qual Res. 2020;20. doi:10.4209/AAQR.2020.05.0254
64. Ravelo-Hernández J, Cáceres-Bernaola U, Becerra-Nuñez C, Mendívil-Tuchia S, Vásquez-Rivas ME, Aguirre-Quispe W, et al. EARLY PREDICTORS OF HOSPITAL MORTALITY IN PATIENTS WITH COVID-19 PNEUMONIA AT A LEVEL III HOSPITAL. LIMA, PERU. 2020. doi:10.1590/SciELOPreprints.1314
65. Reina Ortiz M, Grijalva MJ, Turell MJ, Waters WF, Montalvo AC, Mathias D, et al. Biosafety at Home: How to Translate Biomedical Laboratory Safety Precautions for Everyday Use in the Context of COVID-19. Am J Trop Med Hyg. 2020. doi:https://dx.doi.org/10.4269/ajtmh.20-0677
66. Reis RF, de Melo Quintela B, de Oliveira Campos J, Gomes JM, Rocha BM, Lobosco M, et al. Characterization of the COVID-19 pandemic and the impact of uncertainties, mitigation strategies, and underreporting of cases in South Korea, Italy, and Brazil. Chaos Solitons Fractals. 2020; 109888. doi:https://dx.doi.org/10.1016/j.chaos.2020.109888
67. Remes-Troche J, Coss-Adame E, Amieva-Balmori M, Velasco J, Gómez-Castaños P, Flores-Rendón R, et al. Incidence of “new-onset” constipation and associated factors during lockdown due to the coronavirus-19 pandemic. ResearchSquare. 2020. doi:10.21203/rs.3.rs-77783/v1
68. Remes-Troche JM, Ramos-de-la-Medina A, Manriquez-Reyes M, Martinez-Perez-Maldonado L, Lara EL, Solis-Gonzalez MA. Initial Gastrointestinal Manifestations in Patients with SARS-CoV-2 in 112 patients from Veracruz (Southeastern Mexico). Gastroenterology. 2020. doi:https://dx.doi.org/10.1053/j.gastro.2020.05.055
69. Renata Maurício S. Teaching and literacy practices in COVID-19 pandemic times. Res Soc Dev. 2020;9. doi:10.33448/RSD-V9I7.4430
70. Renato Rodrigues S, Wisley Donizetti V, Wanderson da Silva M, Carlos Augusto Goncalves T. A Bayesian analysis of the total number of cases of the COVID 19 when only a few data is available. A case study in the state of Goias, Brazil. medRxiv. 2020. doi:10.1101/2020.04.19.20071852
71. Renzo JCC-A, Jay SK. Impact of COVID-19 Lockdown Policy on Homicide, Suicide, and Motor Vehicle Deaths in Peru. medRxiv. 2020. doi:10.1101/2020.07.11.20150193
72. Requena D, Medico A, Chacon RD, Ramirez M, Marin-Sanchez O. Identification of Novel Candidate Epitopes on SARS-CoV-2 Proteins for South America: A Review of HLA Frequencies by Country. Front Immunol. 2020;11: 2008. doi:http://dx.doi.org/10.3389/fimmu.2020.02008
73. Requia WJ, Kondo EK, Adams MD, Gold DR, Struchiner CJ. Risk of the Brazilian health care system over 5572 municipalities to exceed health care capacity due to the 2019 novel coronavirus (COVID-19). Sci Total Environ. 2020;730: 139144. doi:https://dx.doi.org/10.1016/j.scitotenv.2020.139144
74. Rex FE, Borges CA de S, Kafer PS. Spatial analysis of the COVID-19 distribution pattern in Sao Paulo State, Brazil. Cien Saude Colet. 2020;25: 3377–3384. doi:https://dx.doi.org/10.1590/1413-81232020259.17082020
75. Reyes-Olavarría D, Latorre-Román PÁ, Guzmán-Guzmán IP, Jerez-Mayorga D, Caamaño-Navarrete F, Delgado-Floody P. Positive and Negative Changes in Food Habits, Physical Activity Patterns, and Weight Status during COVID-19 Confinement: Associated Factors in the Chilean Population. Int J Environ Res Public Health. 2020;17: 1–14. doi:10.3390/ijerph17155431
76. Rezende LFM, Thome B, Schveitzer MC, Souza-Junior PRB de, Szwarcwald CL. Adults at high-risk of severe coronavirus disease-2019 (Covid-19) in Brazil. Rev Saude Publica. 2020;54: 50. doi:https://dx.doi.org/10.11606/s1518-8787.2020054002596
77. Ribeiro H V, Sunahara AS, Sutton J, Perc M, Hanley QS. City size and the spreading of COVID-19 in Brazil. PLoS One. 2020;15: e0239699. doi:https://dx.doi.org/10.1371/journal.pone.0239699
78. Ribeiro MHDM, da Silva RG, Mariani VC, Coelho LDS. Short-term forecasting COVID-19 cumulative confirmed cases: Perspectives for Brazil. Chaos Solitons Fractals. 2020;135: 109853. doi:https://dx.doi.org/10.1016/j.chaos.2020.109853
79. Ribeiro SP, Castro e Silva A, Dattilo W, Reis AB, Goes-Neto A, Alcantara LC, et al. Severe airport sanitarian control could slow down the spreading of COVID-19 pandemics in Brazil. PeerJ. 2020;2020: e9446. doi:http://dx.doi.org/10.7717/peerj.9446
80. Ricardo Vicente F, Marcos RM, Rogerio HT, Luiza MA, Michael Richard D, Eric MD. Applying a Prospective Space-Time Scan Statistic to Examine the Evolution of COVID-19 Clusters in the State of Sao Paulo, Brazil. medRxiv. 2020. doi:10.1101/2020.06.04.20122770
81. Ricardo A, Edgar CC, Anabel SB, Adrian LP, Orlando G, Fernando H, et al. Decision-making on management of ms and nmosd patients during the COVID-19 pandemic: A latin american survey. Mult Scler Relat Disord. 2020;44: 102310. doi:https://dx.doi.org/10.1016/j.msard.2020.102310
82. Ricardo P, Daniel G, Hubert Blas R, Juan Carlos R, albadio P, Lissette del Rosario L, et al. Therapeutic effectiveness of interferon alpha 2b treatment for COVID-19 patient recovery. medRxiv. 2020. doi:10.1101/2020.07.28.20157974
83. Ricardo P, Daniel G, Hubert R, Juan R, Albadio P, Lizet del Rosario L, et al. Therapeutic effectiveness of interferon-alpha2b against COVID-19: the Cuban experience. medRxiv. 2020. doi:10.1101/2020.05.29.20109199
84. Ricardo V, Juan D, Jose F, Jorge S, Fernando P, Dardo R, et al. Convalescent plasma as potential therapy for severe COVID-19 pneumonia. medRxiv. 2020. doi:10.1101/2020.09.01.20184390
85. Richard Oliveira J, Claudia Rakel Pena P. PANDEMIA DO CORONAVÍRUS (COVID-19): análise da evolução de casos frente à disponibilidade de leitos no estado do Maranhão, Brasil, 2020. 2020;5.
86. Ridwan K, Jeff C. Mayoral Party Identity and Social Distancing Behaviors in Brazil. SSRN. 2020. doi:10.2139/ssrn.3642710
87. Rios AFM, Lira LSSP, Reis IM, Silva GA. Atenção Primária à Saúde frente à COVID-19: Relato de experiência de um Centro de Saúde. Enferm foco. 2020;11: 246–251.
88. Rios AFM, Lira LSSP, Reis IM, Silva GA. Primary health care in front of COVID-19: experience report from a health center. Enferm foco. 2020;11: 246–251.
89. Rios-González CM. Knowledge, attitudes and practices towards COVID-19 in Paraguayans during outbreaks: a quick online survey. 2020.
90. Rios-González CM, Palacios JM. Symptoms of Anxiety and depression during the outbreak of COVID-19 in Paraguay. 2020.
91. Rios-Silva M, Murillo-Zamora E, Mendoza-Cano O, Trujillo X, Huerta M. COVID-19 mortality among pregnant women in Mexico: A retrospective cohort study. J Glob Health. 2020;10: 20512. doi:http://dx.doi.org/10.7189/jogh.10.020512
92. Risi LR, Oliveira AB de, Bernardes MMR, Ramos R de S, Moutinho ECV da S, Passos AC. Desenvolvimento de máscaras de tecido e celulose para resposta emergencial à pandemia provocada pelo SARS-CoV-2. Rev enferm UERJ. 2020;28: e51476–e51476.
93. Rivera B, Leyva A, Portela MM, Moratorio G, Moreno P, Durán R, et al. Quantitative proteomic dataset from oro- and naso-pharyngeal swabs used for COVID-19 diagnosis: Detection of viral proteins and host’s biological processes altered by the infection. Data Br. 2020;32: 106121. doi:10.1016/j.dib.2020.106121
94. Rivera VM, Gracia F, Diaz A. Therapeutic attitudes towards multiple sclerosis in Central America and the Caribbean facing the SARS-CoV-2 pandemia. Neurologia. 2020. doi:http://dx.doi.org/10.1016/j.nrl.2020.07.009
95. Rivera-Franco MM, Saldana-Gonzalez ES, Leon-Rodriguez E. Changes within the care of hematopoietic cell transplantation patients after the shift of a Mexican institution to a COVID-19-only center. Hematol Transfus cell Ther. 2020;42: 313–315. doi:http://dx.doi.org/10.1016/j.htct.2020.08.001
96. Rizo-Téllez SA, Méndez-García LA, Flores-Rebollo C, Alba-Flores F, Alcántara-Suárez R, Manjarrez-Reyna AN, et al. The Neutrophil-to-Monocyte Ratio and Lymphocyte-to-Neutrophil Ratio at Admission Predict In-Hospital Mortality in Mexican Patients with Severe SARS-CoV-2 Infection (Covid-19). Microorganisms. 2020;8. doi:10.3390/microorganisms8101560
97. Roberto E, Rodrigo Q. Estimate of the actual number of COVID-19 cases from the analysis of deaths. medRxiv. 2020. doi:10.1101/2020.09.21.20198416
98. Rocha HAL, Alcantara ACC, Netto FCB, Ibiapina FLP, Lopes LA, Rocha SGMO, et al. Dealing with the impact of the COVID-19 pandemic on a Rapid Response Team operation in Brazil: Quality in practice. Int J Qual Heal care  J Int Soc Qual Heal Care. 2020. doi:http://dx.doi.org/10.1093/intqhc/mzaa114
99. Rodrigues Aguilar AC, Frange C, Huebra L, Dias Gomes AC, Tufik S, Santos Coelho FM. The effects of the COVID-19 pandemic on patients with narcolepsy. J Clin Sleep Med. 2020. doi:http://dx.doi.org/10.5664/jcsm.8952
100. Rodrigues NCP, Andrade MKN, Monteiro DLM, Lino VTS, Reis IDN, Frossard VC, et al. COVID-19 morbidity and mortality in 2020: the case of the city of Rio de Janeiro. J Bras Pneumol. 2020;46: e20200341. doi:10.36416/1806-3756/e20200341
101. Rodrigues NH, Silva LGA da. Management of the coronavirus pandemic in a hospital: professional experience report. J nurs Heal. 2020;10: 20104004.
102. Rodríguez Monteagudo MA, Avello Martínez R, Morejón Giraldoni A, Rodríguez Monteagudo P, Avello Rodríguez A. A weekly analysis of cases confirmed to COVID-19 in Cuba: first 70 days. Medisur. 2020;18: 485–491.
103. Rodriguez J, Fletcher A, Heredia F, Fernandez R, Ramirez Salazar H, Sanabria D, et al. Alternative management for gynecological cancer care during the COVID-2019 pandemic: A Latin American survey. Int J Gynaecol Obstet. 2020. doi:https://dx.doi.org/10.1002/ijgo.13272
104. Rodriguez M, Peters A, Perez I, Spencer M, Barbe M, Porte L, et al. Covid-19 in South America: Clinical and Epidemiological Characteristics Among 381 Patients During the Early Phase of the Pandemic in Santiago, Chile. ResearchSquare. 2020. doi:10.21203/rs.3.rs-64458/v1
105. Rodriguez R, Mondeja B, Valdes O, Resik S, Vizcaino A, Acosta E, et al. SARS-CoV-2: Enhancement and Segmentation of High-ResolutionMicroscopy Images. Part I. ResearchSquare. 2020. doi:10.21203/rs.3.rs-65818/v1
106. Rodriguez-Benito C V, Navarro G, Caballero I. Using Copernicus Sentinel-2 and Sentinel-3 data to monitor harmful algal blooms in Southern Chile during the COVID-19 lockdown. Mar Pollut Bull. 2020;161: 111722. doi:http://dx.doi.org/10.1016/j.marpolbul.2020.111722
107. Rogrigues P, Reis EC, Bianchi L, Palma A. Factors associated with physical activity during the COVID-19 pandemic in Rio de Janeiro, Brazil. Rev bras ativ fís saúde. 2020;25: 1–9.
108. Romero P, Sánchez-Yupari M, Montero S, Tsukayama P. Use of complete SARS-CoV-2 genomes to estimate the Rt in Peru during March ­ April 2020. 2020.
109. Romero-Lopez JP, Carnalla-Cortes M, Pacheco-Olvera DL, Ocampo-Godinez JM, Oliva-Ramirez J, Moreno-Manjon J, et al. A bioinformatic prediction of antigen presentation from SARS-CoV-2 spike protein revealed a theoretical correlation of HLA-DRB1*01 with COVID-19 fatality in Mexican population: An ecological approach. J Med Virol. 2020. doi:http://dx.doi.org/10.1002/jmv.26561
110. Rosa MFP, Silva WNT da, Carvalho WRG de, Oliveira SV de. Epidemiology of COVID-19 in Uberlândia (MG): preliminary analysis of the impact of commercial activities’s openness. J Heal NPEPS. 2020;5.
111. Rosalinda P, Carlos A, Jose N, Wilneg Carolina R, Nathalia P, Emily L, et al. PREDICTIVE VALUE OF SMELL AND TASTE TEST VS PCR-RT SARS-COV-2 AND RAPID DIAGNOSTIC TESTS IN THE DIAGNOSIS OF INFECTION BY COVID-19. A PROSPECTIVE MULTI-CENTRIC STUDY. medRxiv. 2020. doi:10.1101/2020.08.31.20185298
112. Rosana Moraes P, Paula Fernanda Ribeiro L, Keulle Oliveira da S, Amanda Alves F, Claudio Alberto Gellis de Mattos D, Sylvia Maria T, et al. Covid-19 and the current scenario of the City of Castanhal – PA, Brazil. Res Soc Dev. 2020;9. doi:10.33448/RSD-V9I8.5717
113. Rosario DKA, Mutz YS, Bernardes PC, Conte-Junior CA. Relationship between COVID-19 and weather: Case study in a tropical country. Int J Hyg Environ Health. 2020;229: 113587. doi:https://dx.doi.org/10.1016/j.ijheh.2020.113587
114. Rosse VP, Pereira JN, Boari A, Costa G V, Ribeiro JPC, Vieira-Filho M. São Paulo’s atmospheric pollution reduction and its social isolation effect, Brazil. Air Qual Atmos Health. 2020; 1–10. doi:10.1007/s11869-020-00959-8
115. Roy KH, Johns D. Transcervical Fibroid Ablation (TFA) in an Ambulatory Surgical Center Setting: Utility during the COVID-19 Pandemic. J Minim Invasive Gynecol. 2020;27: S113. doi:http://dx.doi.org/10.1016/j.jmig.2020.08.178
116. Rubens Lichtenthaler Filho V, Daniel Gomes  Sr. L. A dynamic model for Covid-19 in Brazil. medRxiv. 2020. doi:10.1101/2020.05.10.20097550
117. Ruiz Brunner M de LM, Escobar Zuluaga LJ, Cieri ME, Condinanzi AL, Cuestas E. [COVID-19 and the reality of people with disabilities in Argentina: possibilities of complying with the considerations of the World Health Organization]. COVID-19 y la Real las Pers con Discapac en Argentina posibilidades del cumplimiento las consideraciones planteadas por la Organ Mund la Salud. 2020;77: 168–175. doi:https://dx.doi.org/10.31053/1853.0605.v77.n3.28767
118. Ruiz Mamani PG, Morales-Garcia WC, White M, Marquez-Ruiz MS. Properties of a scale of concern for COVID-19: Exploratory analysis in a Peruvian sample. Med Clin (Barc). 2020. doi:http://dx.doi.org/10.1016/j.medcli.2020.06.022
119. Ruiz-Manriquez J, Leon-Lara X, Campos-Murguia A, Solis-Ortega AA, Perez-Gonzalez B, Uscanga LF, et al. Knowledge of Latin American gastroenterologists and endoscopists regarding SARS-CoV-2 infection. Rev Gastroenterol Mex. 2020. doi:https://dx.doi.org/10.1016/j.rgmx.2020.04.003
120. Ruiz-Patino A, Arrieta O, Pino LE, Rolfo C, Ricaurte L, Recondo G, et al. Mortality and Advanced Support Requirement for Patients With Cancer With COVID-19: A Mathematical Dynamic Model for Latin America. JCO Glob Oncol. 2020;6: 752–760. doi:https://dx.doi.org/10.1200/GO.20.00156
121. Ruíz-Roso MB, de Carvalho Padilha P, Matilla-Escalante DC, Brun P, Ulloa N, Acevedo-Correa D, et al. Changes of Physical Activity and Ultra-Processed Food Consumption in Adolescents from Different Countries during Covid-19 Pandemic: An Observational Study. Nutrients. 2020;12: 1–13. doi:10.3390/nu12082289
122. Ruiz-Roso MB, de Carvalho Padilha P, Mantilla-Escalante DC, Ulloa N, Brun P, Acevedo-Correa D, et al. Covid-19 Confinement and Changes of Adolescent’s Dietary Trends in Italy, Spain, Chile, Colombia and Brazil. Nutrients. 2020;12. doi:https://dx.doi.org/10.3390/nu12061807
123. Saavedra D, Añé-Kourí A, Sánchez N, Filgueira L, Betancourt J, Herrera C, et al. An Anti-CD6 Monoclonal Antibody (Itolizumab) Reduces Circulating IL-6 in Severe Covid-19 Elderly Patients. ResearchSquare. 2020. doi:10.21203/rs.3.rs-32335/v1
124. Sakellariou D, Malfitano APS, Rotarou ES. Disability inclusiveness of government responses to COVID-19 in South America: a framework analysis study. Int J Equity Health. 2020;19: 131. doi:10.1186/s12939-020-01244-x
125. Salama M, Ataman-Millhouse L, Braham M, Berjeb K, Khrouf M, Rodrigues JK, et al. Installing oncofertility programs for common cancers in limited resource settings (Repro-Can-OPEN Study): An extrapolation during the global crisis of Coronavirus (COVID-19) pandemic. J Assist Reprod Genet. 2020. doi:https://dx.doi.org/10.1007/s10815-020-01821-7
126. Saldana F, Flores-Arguedas H, Camacho-Gutierrez JA, Barradas I. Modeling the transmission dynamics and the impact of the control interventions for the COVID-19 epidemic outbreak. Math Biosci Eng. 2020;17: 4165–4183. doi:https://dx.doi.org/10.3934/mbe.2020231
127. Salgotra R, Gandomi M, Gandomi AH. Evolutionary modelling of the COVID-19 pandemic in fifteen most affected countries. Chaos Solitons Fractals. 2020;140: 110118. doi:10.1016/j.chaos.2020.110118
128. Salinas-Escudero G, Carrillo-Vega MF, Granados-Garcia V, Martinez-Valverde S, Toledano-Toledano F, Garduno-Espinosa J. A survival analysis of COVID-19 in the Mexican population. BMC Public Health. 2020;20: 1616. doi:http://dx.doi.org/10.1186/s12889-020-09721-2
129. Samaniego A, Urzúa A, Buenahora M, Vera-Villarroel P. Sintomatología asociada a trastornos de salud mental en trabajadores sanitarios en paraguay: Efecto COVID-19. Rev Interam Psicol. 2020;54. doi:10.30849/ripijp.v54i1.1298
130. Samara Avelino de Souza F, Angelo Cezar Pinho T. A COVID-19 e os desafios da urbanização e habitabilidade nas cidades amazônicas: estudo de caso em Belém do Pará. 2020;29. doi:10.18542/PAPERSNAEA.V29I1.8861
131. Samy D, Alexandre BS, Bruno AF, Rodrigo NR, Leandro Lane da Costa V, Jose G-N. Brazilian Modeling of COVID-19 (BRAM-COD): a Bayesian Monte Carlo approach for COVID-19 spread in a limited data set context. medRxiv. 2020. doi:10.1101/2020.04.29.20081174
132. Sánchez Barajas DA, Maita Blanco EC, Garrido Urdaneta F, García Rojas JM. Epidemiological analysis of COVID-19. Bolivarian Republic of Venezuela. Obs del Conoc Rev Espec en Gestión Soc del Conoc. 2020;5: 32–52.
133. Sánchez Hernández E, Cardona Gordo O, Ferrer Castro JE, Pérez Fouces F, Despaigne Bicet A. Variability of the evolutionary PCR results in patients with COVID -19. Medisan. 2020;24: 565–577.
134. Sandes S, Freitas A dos S. SIR model with exposure rate for the study of the projection of COVID-19 cases in Sergipe. 2020.
135. Santamaría-Holek I, Castaño V. Possible fates of the spread of SARS-CoV-2 in the Mexican context. R Soc open Sci. 2020;7: 200886. doi:10.1098/rsos.200886

Ivan S-H, Victor C. Possible fates of the dispersion of SARS-COV-2 in the Mexican context. medRxiv. 2020. doi:10.1101/2020.07.15.20154526

1. Santana F, Lopes J, Perez M, Campana G, Levi J, Lopes F, et al. Seroconversion for SARS-CoV-2 in rheumatic patients on synthetic and biologics Disease Modifying Anti-Rheumatic Drugs in São Paulo, Brazil. ResearchSquare. 2020. doi:10.21203/rs.3.rs-97191/v1
2. Santana-Cibrian M, Acuna-Zegarra MA, Velasco-Hernandez JX. Lifting mobility restrictions and the effect of superspreading events on the short-term dynamics of COVID-19. Math Biosci Eng. 2020;17: 6240–6258. doi:http://dx.doi.org/10.3934/mbe.2020330

Mario S-C, Manuel AA-Z, Jorge XV-H. Lifting mobility restrictions and the induced short-term dynamics of COVID-19. medRxiv. 2020. doi:10.1101/2020.07.23.20161026

1. Santos DS, Menezes MO, Andreucci CB, Nakamura-Pereira M, Knobel R, Katz L, et al. Disproportionate impact of COVID-19 among pregnant and postpartum Black Women in Brazil through structural racism lens. Clin Infect Dis. 2020. doi:10.1093/cid/ciaa1066
2. Santos HLPCD, Maciel FBM, Santos KR, Conceicao CDVSD, Oliveira RS, Silva NRFD, et al. Necropolitics and the impact of COVID-19 on the Black community in Brazil: a literature review and a document analysis. Cien Saude Colet. 2020;25: 4211–4224. doi:http://dx.doi.org/10.1590/1413-812320202510.2.25482020
3. Santos I, Almeida G, Moura F. Adaptive SIR model for propagation of SARS-CoV-2 in Brazil. ResearchSquare. 2020. doi:10.21203/rs.3.rs-40116/v1
4. Santos JPC Dos, Siqueira ASP, Praca HLF, Albuquerque HG. Vulnerability to severe forms of COVID-19: an intra-municipal analysis in the city of Rio de Janeiro, Brazil. Cad Saude Publica. 2020;36: e00075720. doi:https://dx.doi.org/10.1590/0102-311x00075720
5. Santos JF, Dahmer Santos D. Hierarchy of Flu-like symptoms related to COVID-19 according to sex and color or race in reports of patients with Severe Acute Respiratory Syndrome in Brazil. 2020. doi:10.1590/SciELOPreprints.1161
6. Santos LSE, Nunes LMM, Rossi BA, Taets G. Impacts of the COVID-19 pandemic on violence against women: reflections from the theory of human motivation from Abraham Maslow. 2020.
7. Santos MM, Lucena EES, Lima KC, Brito AAC, Bay MB, Bonfada D. Survival and predictors of deaths of patients hospitalised due to COVID-19 from a retrospective and multicentre cohort study in Brazil. Epidemiol Infect. 2020;148: e198. doi:https://dx.doi.org/10.1017/S0950268820002034
8. Santos VS, Quintans-Júnior LJ, de Souza Barboza W, Antunes de Souza Araújo A, Ricardo Martins-Filho P. Clinical Characteristics and Outcomes in Patients With COVID-2019 and Leprosy. J Eur Acad Dermatol Venereol. 2020. doi:10.1111/jdv.16899
9. Santos VS, Souza Araujo AA, de Oliveira JR, Quintans-Junior LJ, Martins-Filho PR. COVID-19 mortality among Indigenous people in Brazil: a nationwide register-based study. J Public Health (Oxf). 2020. doi:http://dx.doi.org/10.1093/pubmed/fdaa176
10. São Paulo Secretaria da S. Serological survey for SARS-COV-2: prevalence of infection in students from public and private schools in the city of São Paulo: phases 1 to 3. 2020.
11. Sarabia-Cobo C, Pérez V, De Lorena P, Hermosilla-Grijalbo C, Sáenz-Jalón M, Fernández-Rodríguez A, et al. Experiences of geriatric nurses in nursing home settings across four countries in the face of the COVID-19 pandemic. J Adv Nurs. 2020. doi:10.1111/jan.14626
12. Saravia-Bartra MM, Cazorla-Saravia P, Cedillo-Ramirez L. Anxiety level of first-year medicine students at a private university in peru in times of covid-19. Rev la Fac Med Humana. 2020;20: 568–573. doi:http://dx.doi.org/10.25176/RFMH.V20I4.3198
13. Saueressig MG, Hackmann CL, Silva CES da, Ferreira J. Estimation of patients hospitalized for COVID-19 in an intensive care unit at the peak of the pandemic in Porto Alegre: Study with epidemiological model SEIHDR. 2020.
14. Saulo BB, Marcelo MM, Daniel OC anda JEN-R. The COVID-19 (SARS-CoV-2) Uncertainty Tripod in Brazil: Assessments on model-based predictions with large under-reporting. arXiv Popul Evol. 2020.
15. Savi P V, Savi MA, Borges B. A Mathematical Description of the Dynamics of Coronavirus Disease 2019 (COVID-19): A Case Study of Brazil. Comput Math Methods Med. 2020;2020: 9017157. doi:http://dx.doi.org/10.1155/2020/9017157

Marcelo AS, Beatriz B, Pedro VS. A Mathematical Description of the Dynamics of Coronavirus Disease (COVID-19): A Case Study of Brazil. arXiv Popul Evol. 2020.

1. Schinkothe T, Gabri MR, Mitterer M, Gouveia P, Heinemann V, Harbeck N, et al. A Web- and App-Based Connected Care Solution for COVID-19 In- and Outpatient Care: Qualitative Study and Application Development. JMIR public Heal Surveill. 2020;6: e19033. doi:https://dx.doi.org/10.2196/19033
2. Schmulson M, Gudino-Zayas M, Hani A. The Impact of COVID-19 Pandemic on Neurogastroenterologists in Latin America: Results of an Online Survey. J Clin Gastroenterol. 2020. doi:http://dx.doi.org/10.1097/MCG.0000000000001413
3. Schuch FB, Bulzing RA, Meyer J, Vancampfort D, Firth J, Stubbs B, et al. Associations of moderate to vigorous physical activity and sedentary behavior with depressive and anxiety symptoms in self-isolating people during the COVID-19 pandemic: A cross-sectional survey in Brazil. Psychiatry Res. 2020;292: 113339. doi:10.1016/j.psychres.2020.113339
4. Sebastian Iglesias O, Johnny Leandro Saavedra C. Aplicaciones de búsqueda de Google para la comunicación de riesgos en el manejo de la COVID-19. 2020;24.
5. Seclen SN, Nunez-Robles E, Yovera-Aldana M, Arias- Chumpitaz A. Incidence of COVID-19 infection and prevalence of diabetes, obesity and hypertension according to altitude in Peruvian population. Diabetes Res Clin Pract. 2020;169: 108463. doi:http://dx.doi.org/10.1016/j.diabres.2020.108463
6. Segovia-Juarez J, Castagnetto JM, Gonzales GF. High altitude reduces infection rate of COVID-19 but not case-fatality rate. Respir Physiol Neurobiol. 2020;281: 103494. doi:https://dx.doi.org/10.1016/j.resp.2020.103494
7. Seligmann H, Iggui S, Rachdi M, Vuillerme N, Demongeot J. Inverted Covariate Effects for First versus Mutated Second Wave Covid-19: High Temperature Spread Biased for Young. Biology (Basel). 2020;9. doi:10.3390/biology9080226
8. Sergio C. Modeling COVID-19 as a National Dynamics with a SARS-CoV-2 Prevalent Variant: Brazil - A Study Case. medRxiv. 2020. doi:10.1101/2020.09.25.20201558
9. Sergio C. Quantifying Effects, Forecasting Releases, and Herd Immunity of the Covid-19 Epidemic in S. Paulo, Brazil. medRxiv. 2020. doi:10.1101/2020.05.20.20107912
10. Serpa Franco A, de Mendonça Henrique D, Ferreira de Almeida L, Moreira do Prado L, Schwenck Gomes RV, Lyra da Silva RC. Segurança na intubação de sequência rápida recomendada no COVID-19: Relato de experiência. Rev Cuid. 2020;11: 1–11. doi:10.15649/cuidarte.1260
11. Servin-Caamano A, Reyes-Herrera D, Flores-Lopez A, Robiou-Vivero EJA, Martinez-Rivera F, Galindo-Hernandez V, et al. Impact of liver enzymes on SARSCoV-2 infection and on the severity of clinical disease. Ann Hepatol. 2020;19: 20. doi:http://dx.doi.org/10.1016/j.aohep.2020.08.044
12. Shanbhag NM, Duncan A, Santos EGD, Yazigi H, Grant-Tate M, Girgis NFF, et al. Results of the Survey Conducted Among Caribbean Physicians on a Zoom Meeting Discussing the Article "A Practical Approach to the Management of Cancer Patients During the Novel Coronavirus Disease 2019 (COVID-19) Pandemic: An International Collaborative Gr. Oncologist. 2020. doi:http://dx.doi.org/10.1002/onco.13556
13. Shen X, Cai C, Li H. Socioeconomic restrictions slowdown COVID-19 far more effectively than favorable weather-evidence from the satellite. Sci Total Environ. 2020;748: 141401. doi:10.1016/j.scitotenv.2020.141401
14. Shima S, Soheil N, Jiang L, Ayesha K, Durgesh C, Ghassem F, et al. Risk of Cerebrovascular Events in Hospitalized Patients with SARS-CoV-2 Infection. SSRN. 2020. doi:10.2139/ssrn.3605289
15. Shunxiang H, Lin W, Li X, Aihong Z, Li S, Feng L, et al. Transmission Dynamics of Coronavirus Disease 2019 (COVID-19) in the World: The Roles of Intervention and Seasonality. medRxiv. 2020. doi:10.1101/2020.07.17.20156430
16. Siciliano B, Carvalho G, da Silva CM, Arbilla G. The Impact of COVID-19 Partial Lockdown on Primary Pollutant Concentrations in the Atmosphere of Rio de Janeiro and Sao Paulo Megacities (Brazil). Bull Environ Contam Toxicol. 2020. doi:https://dx.doi.org/10.1007/s00128-020-02907-9
17. Siciliano B, Dantas G, da Silva CM, Arbilla G. Increased ozone levels during the COVID-19 lockdown: Analysis for the city of Rio de Janeiro, Brazil. Sci Total Environ. 2020;737: 139765. doi:https://dx.doi.org/10.1016/j.scitotenv.2020.139765
18. Siewe Fodjo JN, Faria de Moura Villela E, Van Hees S, Tibério Dos Santos T, Vanholder P, Vanholder P, et al. Impact of the COVID-19 pandemic on the medical follow-up and psychosocial well-being of people living with HIV: A cross-sectional survey. J Acquir Immune Defic Syndr. 2020. doi:10.1097/QAI.0000000000002468
19. Silberman P, López E, Medina A, Díaz Bazán JM, Gómez Marquisio MD, López GA. Tele-revistas en unidades de terapia intensiva: Coordinación asistencial y aprendizaje en el marco de la pandemia. Rev argent salud publica. 2020;12: 1–5.
20. Silva APSC, Maia LTS, Souza W V. Severe Acute Respiratory Syndrome in Pernambuco: comparison of patterns before and during the COVID-19 pandemic. Cien Saude Colet. 2020;25: 4141–4150. doi:http://dx.doi.org/10.1590/1413-812320202510.2.29452020
21. Silva ARA da, Leal IA, Gonçalves FM, Setti RF, Tavares MC, Souza CV, et al. INFLUENCE OF SOCIAL DISTANCING DUE TO COVID-19 PANDEMIC IN EMERGENCY ATTENDANCE AND HOSPITALIZATIONS IN PEDIATRICS. 2020. doi:10.1590/SciELOPreprints.364
22. Silva CPG da, Silva VC da, Britto PF, Jesus D de, Nonato VWC, Vilella RR. Educational activities for proper use of personal protection equipment in a federal reference hospital. Enferm foco. 2020;11: 228–233.
23. Silva DF da, Oliveira MLC de. Epidemiologia da COVID-19: comparação entre boletins epidemiológicos. Comun ciênc saúde. 2020;31.
24. Silva GCDA, Abe DK, Pedrenho R, Vilares RN, Cordeiro MD, Coelho RF, et al. Evaluation of uro-oncological surgical treatment during the Sars-CoV-2 pandemic in a Brazilian tertiary oncology institution, the new world epicenter. Int Braz J Urol. 2020;47. doi:http://dx.doi.org/10.1590/S1677-5538.IBJU.2020.0479
25. Silva GAE, Jardim BC, Santos CVB Dos. Excess mortality in Brazil in times of Covid-19. Excesso mortalidade no Bras em tempos COVID-19. 2020;25: 3345–3354. doi:https://dx.doi.org/10.1590/1413-81232020259.23642020
26. Silva JHD, Oliveira EC, Hattori TY, Lemos ERS, Terças-Trettel ACP. Description of COVID-19 cluster: isolation and testing in asymptomatic individuals as strategies to prevent local dissemination in Mato Grosso state, Brazil, 2020. Epidemiol e Serv saude  Rev do Sist Unico Saude do Bras. 2020;29: e2020264. doi:10.5123/s1679-49742020000400005
27. Silva L, Figueiredo Filho D. Using Benford’s law to assess the quality of COVID-19 register data in Brazil. J Public Health (Oxf). 2020. doi:http://dx.doi.org/10.1093/pubmed/fdaa193
28. Silva L, Figueiredo Filho D, Fernandes A. The effect of lockdown on the COVID-19 epidemic in Brazil: evidence from an interrupted time series design. Cad Saude Publica. 2020;36: e00213920. doi:http://dx.doi.org/10.1590/0102-311X00213920
29. Silva LLS da, Lima AFR, Polli DA, Razia PFS, Pavao LFA, Cavalcanti MAF de H, et al. Social distancing measures in the fight against COVID-19 in Brazil: description and epidemiological analysis by state. Medidas distanciamento Soc para o enfrentamento da COVID-19 no Bras Caracter e Anal Epidemiol por estado. 2020;36: e00185020. doi:https://dx.doi.org/10.1590/0102-311X00185020
30. Silva MT, Quintanilha G, Giesel L, Soldati AB, Jabarra C, Pereira C de A, et al. The Impact of the COVID-19 pandemic on a stroke center in Latin America. Int J Stroke. 2020; 1747493020941637. doi:https://dx.doi.org/10.1177/1747493020941637
31. Silva RM da. Using the SIRD model to characterize the COVID-19 spreading in the states of Paraná, Rio Grande do Sul, and Santa Catarina. 2020.
32. Silva R da. Estimates of effective reproduction number of SARS­CoV­2 in Brazil using phylodynamic analysis. 2020.
33. Silva RR da, Santos MB dos, Santos AD dos, Tavares D dos S, Santos PL dos. Coronavirus disease and basic sanitation: too early to be worried? Rev Soc Bras Med Trop. 2020;53. doi:10.1590/0037-8682-0345-2020
34. Silva RJ, Silva K, Mattos J. Spatial analysis on the dispersion of covid-19 in the state of Bahia. 2020.
35. Silva R, Pessanha C, Meireles I, Coura C, Silva C, Peregrino A. Budgetarry impact of the incorporation of claritromycin in the treatment of infection by new coronavirus. 2020. doi:10.1590/SciELOPreprints.1026
36. Silva R, Silva C, Machado D, Peregrino A, Marta C, Pestana L, et al. Lost years of life adjusted for disability (DALY) among nursing professionals due to COVID-19 infection in Brazil. 2020.
37. Silva-Júnior MJ da, Mendonça KS, Lima CA de, Pires PLS, Calegari T, Oliveira SV de. Analysis of the spatial-temporal dynamics of incidence, mortality and test rates (rapid and RT-PCR) of COVID-19 in the state of Minas Gerais, Brazil. 2020.
38. Silva-Malta MCF, Rodrigues DDOW, Chaves DG, Magalhaes NNS, Ribeiro MA, Cioffi JGM, et al. Impact of COVID-19 in the attendance of blood donors and production on a Brazilian Blood Centres. Transfus Med. 2020. doi:http://dx.doi.org/10.1111/tme.12733
39. Silvana F, Vanina P, Lorena L, Julieta S, Magdalena Wagner M, Alicia M, et al. Community-level SARS-CoV-2 Seroprevalence Survey in urban slum dwellers of Buenos Aires City, Argentina: a participatory research. medRxiv. 2020. doi:10.1101/2020.07.14.20153858
40. Silvano B de O, Victor Bertollo Gomes P, Fabiana G, Fabio Macedo M, Maria A, Wanderson Kleber de O, et al. Monitoring social distancing and SARS-CoV-2 transmission in Brazil using cell phone mobility data. medRxiv. 2020. doi:10.1101/2020.04.30.20082172
41. Silva-Rodriguez EA, Galvez N, Swan GJF, Cusack JJ, Moreira-Arce D. Urban wildlife in times of COVID-19: What can we infer from novel carnivore records in urban areas? Sci Total Environ. 2020; 142713. doi:http://dx.doi.org/10.1016/j.scitotenv.2020.142713
42. Silva-Tinoco R, GonzÁLez-CantÚ A, De La Torre-SaldaÑA V, GuzmÁN-Olvera E, Cuatecontzi-Xochitiotzi T, Castillo-MartÍNez L, et al. Effect in self-care behavior and difficulties in coping with diabetes during the COVID-19 pandemic. Rev Mex Endocrinol Metab y Nutr. 2020;7: 1–7. doi:10.24875/RME.20000063
43. Silveira MF, Barros AJD, Horta BL, Pellanda LC, Victora GD, Dellagostin OA, et al. Population-based surveys of antibodies against SARS-CoV-2 in Southern Brazil. Nat Med. 2020. doi:10.1038/s41591-020-0992-3
44. Silvina R, Hector C, Javier P, Sandra Di L, Maria Jose C, Fernando Adrian T, et al. Children hospitalized for COVID-19 during first winter of the pandemic in Buenos Aires, Argentina. medRxiv. 2020. doi:10.1101/2020.11.05.20225300
45. Simian M ME, Cifuentes D M. Epidemiological characterization of patients with Covid-19 in the Hospital Clínico Universidad de Chile. Rev Hosp Clin Univ Chile. 2020;31: 103–108.
46. Singh RK, Rani M, Bhagavathula AS, Sah R, Rodriguez-Morales AJ, Kalita H, et al. Prediction of the COVID-19 Pandemic for the Top 15 Affected Countries: Advanced Autoregressive Integrated Moving Average (ARIMA) Model. JMIR public Heal Surveill. 2020;6: e19115. doi:https://dx.doi.org/10.2196/19115
47. Sippy R, Prado EO, Pizarro Fajardo F, Hidalgo I, Aguilar G V, Bonville CA, et al. Medically Attended Outpatient Coronavirus Infections in Ecuadorean Children During the 20 Months Preceding Countrywide Lockdown Related to the SARS-CoV-2 Pandemic of 2020. Pediatr Infect Dis J. 2020. doi:10.1097/INF.0000000000002840
48. Skums P, Kirpich A, Icer Baykal P, Zelikovsky A, Chowell G. Global transmission network of SARS-CoV-2: from outbreak to pandemic. medRxiv  Prepr Serv Heal Sci. 2020. doi:https://dx.doi.org/10.1101/2020.03.22.20041145
49. Soares RCM, Mattos LR, Raposo LM. Risk Factors for Hospitalization and Mortality due to COVID-19 in Espírito Santo State, Brazil. Am J Trop Med Hyg. 2020. doi:10.4269/ajtmh.20-0483
50. Sola A, Rodríguez S, Cardetti M, Dávila C. [Perinatal COVID-19 in Latin America]. Rev Panam Salud Publica. 2020;44: e47. doi:10.26633/RPSP.2020.47
51. Sorbara M, Graviotto HG, Lage-Ruiz GM, Turizo-Rodriguez CM, Sotelo-Lopez LA, Serra A, et al. COVID-19 and the forgotten pandemic: follow-up of neurocognitive disorders during lockdown in Argentina. Neurologia. 2020. doi:http://dx.doi.org/10.1016/j.nrl.2020.07.015
52. Soriano Sanchez JA, Perilla Cepeda TA, Zenteno M, Campero A, Yampolsky C, Varela ML, et al. Early Report on the Impact of COVID-19 Outbreak in Neurosurgical Practice Among Members of the Latin American Federation of Neurosurgical Societies. World Neurosurg. 2020. doi:https://dx.doi.org/10.1016/j.wneu.2020.04.226
53. Soto R N, Cortés M C, Muñoz P M, Herrera G ME, Postigo V J, Suquilanda J A, et al. Clinical and epidemiological characterization of patients with Covid-19 treated in the Emergency Service of the Hospital Clínico Universidad de Chile between March and May 2020. Rev Hosp Clin Univ Chile. 2020;31: 97–102.
54. Sousa AR de, Carvalho ES de S, Santana T da S, Sousa AFL, Figueiredo TFG, Escobar OJV, et al. Men’s feelings and emotions in the Covid-19 framing. Sentim e emocoes homens no enquadramento da doenca Covid-19. 2020;25: 3481–3491. doi:https://dx.doi.org/10.1590/1413-81232020259.18772020
55. Sousa AR de, Santana TDS, Palma EMS, Sousa AF de, Moreira WC, Rezende MF, et al. SARS-CoV-2 in Brazil and the psychosocial repercussions on male health: a socio-historical study. 2020.
56. Sousa GJB, Garces TS, Cestari VRF, Florencio RS, Moreira TMM, Pereira MLD. Mortality and survival of COVID-19. Epidemiol Infect. 2020;148: e123. doi:https://dx.doi.org/10.1017/S0950268820001405
57. Sousa GJB, Garces TS, Cestari VRF, Moreira TMM, Florêncio RS, Pereira MLD. Estimation and prediction of COVID-19 cases in Brazilian metropolises. Rev latinoam enferm. 2020;28: e3345–e3345.
58. Souza A, Mota C, Rosa A, Figueiredo C, Candeias A. An Environmental GIS-based Variable Analysis on SARS-CoV-2 in the City of Recife, Brazil. ResearchSquare. 2020. doi:10.21203/rs.3.rs-95748/v1
59. Souza CT V, Santana CS, Ferreira P, Nunes JA, Teixeira MLB, Gouvêa M. Caring in the age of COVID-19: lessons from science and society. Cad Saude Publica. 2020;36: e00115020. doi:10.1590/0102-311X00115020
60. Souza CDF de, Leal TC, Santos LG. Circulatory System Diseases in Patients with COVID-19: Description of Clinical and Epidemiological Profile of 197 Deaths. Doencas do Aparel Circ em Individuos com COVID-19 Descricao do Perf Clin e Epidemiol 197 Obitos. 2020;115: 281–283. doi:https://dx.doi.org/10.36660/abc.20200453
61. Souza CDF de, Paiva JPS de, Leal TC, Silva LF da, Santos LG. Spatiotemporal evolution of case fatality rates of COVID-19 in Brazil, 2020. J Bras Pneumol. 2020;46: e20200208. doi:https://dx.doi.org/10.36416/1806-3756/e20200208
62. Souza JB de, Conceição VM da, Araújo JS, Bitencourt JV de OV, Silva Filho CC da, Rossetto M. Cancer in time of COVID-19: repercussions in the lives of women undergoing oncological treatment. Rev enferm UERJ. 2020;28: e51821–e51821.
63. Souza L, Lima F, Roca T, Santos A, Naveca F, Maia A, et al. SARS-COV-2 genomic surveillance in the Brazilian Western Amazon region: evolutionary history of epidemic dissemination and genetic signature in Rondônia, Brazil. ResearchSquare. 2020. doi:10.21203/rs.3.rs-72476/v1
64. Souza LG, Randow R, Lima PC. Reflections about the COVID-19: differentials by sex and age. Comun ciênc saúde. 2020;31.
65. Souza LF de. Informal app workers and the impact of disease by the new Coronavirus: a theoretical reflection. J nurs Heal. 2020;10: 20104011.
66. Srijit B, Md Moinul I, Alokkumar DE. Search for trends of Covid-19 infection in India, China, Denmark, Brazil, France. Germany and the USA on the basis of power law scaling. medRxiv. 2020. doi:10.1101/2020.04.03.20052878
67. Steele EM, Rauber F, Costa CDS, Leite MA, Gabe KT, Louzada ML da C, et al. Dietary changes in the NutriNet Brasil cohort during the covid-19 pandemic. Rev Saude Publica. 2020;54: 91. doi:https://dx.doi.org/10.11606/s1518-8787.2020054002950
68. Stephen XZ, Jiyao C, Asghar Afshar J, Aldo A-R, Huiyang D, Jizhen L, et al. Succumbing to the COVID-19 Pandemic: Healthcare Workers not Satisfied and Intend to Leave Their Jobs. medRxiv. 2020. doi:10.1101/2020.05.22.20110809
69. Stephen XZ, Yifei W, Asghar Afshar J, Jianfeng J, Valentina Gomes Haensel S. First study on mental distress in Brazil during the COVID-19 crisis. medRxiv. 2020. doi:10.1101/2020.04.18.20070896
70. Studart-Neto A, Guedes BF, Tuma RLE, Camelo Filho AE, Kubota GT, Iepsen BD, et al. Neurological consultations and diagnoses in a large, dedicated COVID-19 university hospital. Arq Neuropsiquiatr. 2020. doi:10.1590/0004-282x20200089
71. Suarez V, Suarez Quezada M, Oros Ruiz S, Ronquillo De Jesus E. Epidemiology of COVID-19 in Mexico: from the 27th of February to the 30th of April 2020. Rev Clin Esp. 2020. doi:https://dx.doi.org/10.1016/j.rce.2020.05.007
72. Sun SY, Guazzelli CAF, de Morais LR, Dittmer FP, Augusto MN, Soares AC, et al. Effect of delayed obstetric labor care during the COVID-19 pandemic on perinatal outcomes. Int J Gynaecol Obstet. 2020;151: 287–289. doi:10.1002/ijgo.13357
73. Szente Fonseca SN, de Queiroz Sousa A, Wolkoff AG, Moreira MS, Pinto BC, Valente Takeda CF, et al. Risk of hospitalization for Covid-19 outpatients treated with various drug regimens in Brazil: Comparative analysis. Travel Med Infect Dis. 2020;38: 101906. doi:http://dx.doi.org/10.1016/j.tmaid.2020.101906
74. Szwarcwald CL, Souza PRB de, Malta DC, Barros MB de A, Magalhães M de AFM, Xavier DR, et al. Adherence to physical contact restriction measures and the spread of COVID-19 in Brazil. Epidemiol e Serviços Saúde. 2020;29.
75. Taboada B, Vazquez-Perez JA, Muñoz Medina JE, Ramos Cervantes P, Escalera-Zamudio M, Boukadida C, et al. Genomic Analysis of Early SARS-CoV-2 Variants Introduced in Mexico. J Virol. 2020. doi:10.1128/JVI.01056-20

Blanca T, Joel AV-P, Jose Esteban M-M, Pilar R-C, Marina E-Z, Celia B, et al. Genomic analysis of early SARS-CoV-2 strains introduced in Mexico. bioRxiv. 2020. doi:10.1101/2020.05.27.120402

1. Tagliazucchi E, Balenzuela P, Travizano M, Mindlin GB, Mininni PD. Lessons from being challenged by COVID-19. Chaos Solitons Fractals. 2020;137: 109923. doi:https://dx.doi.org/10.1016/j.chaos.2020.109923
2. Takemoto MLS, Menezes MO, Andreucci CB, Knobel R, Sousa LAR, Katz L, et al. Maternal mortality and COVID-19. J Matern Fetal Neonatal Med. 2020; 1–7. doi:10.1080/14767058.2020.1786056
3. Takemoto MLS, Menezes MO, Andreucci CB, Knobel R, Sousa LAR, Katz L, et al. Clinical characteristics and risk factors for mortality in obstetric patients with severe COVID-19 in Brazil: a surveillance database analysis. BJOG. 2020. doi:10.1111/1471-0528.16470
4. Tang Y, Serdan TDA, Masi LN, Tang S, Gorjao R, Hirabara SM. Epidemiology of COVID-19 in Brazil: using a mathematical model to estimate the outbreak peak and temporal evolution. Emerg Microbes Infect. 2020; 1–11. doi:https://dx.doi.org/10.1080/22221751.2020.1785337
5. Tarcisio MRF, Fabiana SG dos S, Victor BG, Thiago AHR, Julio HRC, Walter MR, et al. Expected impact of COVID-19 outbreak in a major metropolitan area in Brazil. medRxiv. 2020. doi:10.1101/2020.03.14.20035873
6. Tatiane Cristina Moraes de S, Natalia de Paula M, Jose K, Isabel Seelaender CR, Marcela Z, Maria Amelia SMV, et al. Socioeconomic Vulnerabilities and the Intensity of RT-PCR SARS-CoV-2 Testing Efforts in the Public Health System in Sao Paulo State. medRxiv. 2020. doi:10.1101/2020.10.29.20221960
7. Tavares DM dos S, Oliveira NGN, Marchiori GF, Guimarães MSF, Santana LPM. Elderly individuals living by themselves: knowledge and measures to prevent the novel coronavirus. Rev Lat Am Enfermagem. 2020;28. doi:10.1590/1518-8345.4675.3383
8. Teich VD, Klajner S, Almeida FAS, Dantas ACB, Laselva CR, Torritesi MG, et al. Epidemiologic and clinical features of patients with COVID-19 in Brazil. Einstein (Sao Paulo). 2020;18: eAO6022. doi:10.31744/einstein_journal/2020ao6022
9. Teixeira L, Freitas RL de, Abad A, Silva JA da, Antonelli-Ponti M, Bastos S, et al. Anxiety-related psychological impacts in the COVID-19 pandemic on cardiovascular diseases and diabetes. 2020. doi:10.1590/SciELOPreprints.1010
10. Teixeira SC. Circumnavigating the challenges of COVID-19 for Indigenous people: perspectives for public health. Public Health. 2020;186: 127–128. doi:http://dx.doi.org/10.1016/j.puhe.2020.07.021
11. Tenorio-Mucha J, Lazo-Porras M, Monroy-Hidalgo A, Malaga G, Cardenas MK. Prices of essential drugs for management and treatment of COVID-19 in public and private Peruvian pharmacies. Acta Medica Peru. 2020;37: 267–277. doi:http://dx.doi.org/10.35663/amp.2020.373.1560
12. Teoh JY-C, Ong WLK, Gonzalez-Padilla D, Castellani D, Dubin JM, Esperto F, et al. A Global Survey on the Impact of COVID-19 on Urological Services. Eur Urol. 2020. doi:https://dx.doi.org/10.1016/j.eururo.2020.05.025
13. Tercio D-C, Daniel Souza L, Antonio  Jr. CM, Bruno MP, Marcelo Augusto Fontenelle Ribeiro J. The trauma and acute care surgeon in the covid-19 pandemic era/ O cirurgião de trauma e emergência na era da pandemia de covid-19. 2020;1.
14. The Gibraltar C-RGHS, Daniel G. Oxygen and mortality in COVID-19 pneumonia: a comparative analysis of supplemental oxygen policies and health outcomes across 26 countries. medRxiv. 2020. doi:10.1101/2020.07.03.20145763
15. Thiago Barbosa S, Thais de Rezende Bessa G, Larissa Gomes da S, Cintia Braga da S, Larissa Antônio da S, Amanda Felix A. The psychological health of Nursing professionals during direct coping with COVID-19 in Brazil. Res Soc Dev. 2020;9. doi:10.33448/RSD-V9I8.5913
16. Thiago Christiano S, Leandro A, Liang Z. Quantitative Analysis of the Effectiveness of Public Health Measures on COVID-19 Transmission. medRxiv. 2020. doi:10.1101/2020.05.15.20102988
17. Thiago Gomes H, Matias Nunes F, Carlos Henrique Ramires F, Mirna Stela L, Marilia Arndt M, Giovano Pereira B, et al. Insufficient social distancing may be related to a future COVID-19 outbreak in Ijui-Brazil: Predictions of further social interventions. medRxiv. 2020. doi:10.1101/2020.06.22.20132910
18. Thiago Henrique Evangelista A, Tafarel Andrade de S, Samyla de Almeida S, Nayani Alves R, Stefan Vilges de O. Underreporting of death by COVID-19 in Brazil’s second most populous state. medRxiv. 2020. doi:10.1101/2020.05.20.20108415
19. Thomas AM, Henrique HH, Swapnil M, Charlie W, Ricardo PS, Axel G, et al. Report 21: Estimating COVID-19 cases and reproduction number in Brazil. medRxiv. 2020. doi:10.1101/2020.05.09.20096701
20. Thomas P, Tara P, Djane Clarys B-S, Vanderson S, Wuelton Marcelo M, James W, et al. INFERRED RESOLUTION THROUGH HERD IMMMUNITY OF FIRST COVID-19 WAVE IN MANAUS, BRAZILIAN AMAZON. medRxiv. 2020. doi:10.1101/2020.09.25.20201939
21. Thomaz Ugliara Barone M, Bega Harnik S, Vieira de Luca P, Letícia de Souza Lima B, José Pineda Wieselberg R, Ngongo B, et al. The Impact of COVID-19 on People with Diabetes in Brazil. Diabetes Res Clin Pract. 2020;166: 108304. doi:10.1016/j.diabres.2020.108304
22. Thome B, Rezende LFM, Schveitzer MC, Monteiro CN, Goldbaum M. Differences in the prevalence of risk factors for severe COVID-19 across regions of Sao Paulo City. Rev Bras Epidemiol. 2020;23: e200087. doi:https://dx.doi.org/10.1590/1980-549720200087
23. Timothy MT, Fresia C, Harold Andre G, Rómulo F-M, Francisco CV, Claudia M. Altitude as a protective factor from COVID-19. medRxiv. 2020. doi:10.1101/2020.08.03.20167262
24. Tomas de-C-B. A modified SEIR Model with Confinement and Lockdown of COVID-19 for Costa Rica. medRxiv. 2020. doi:10.1101/2020.05.19.20106492
25. Tomazini BM, Maia IS, Cavalcanti AB, Berwanger O, Rosa RG, Veiga VC, et al. Effect of Dexamethasone on Days Alive and Ventilator-Free in Patients with Moderate or Severe Acute Respiratory Distress Syndrome and COVID-19: The CoDEX Randomized Clinical Trial. JAMA - J Am Med Assoc. 2020;324: 1307–1316. doi:http://dx.doi.org/10.1001/jama.2020.17021

Bruno Martins T, Israel SM, Flavia RB, Maria Vitoria AOS, Franca PB, Eduardo Leite VC, et al. COVID-19-associated ARDS treated with DEXamethasone (CoDEX): Study design and rationale for a randomized trial. medRxiv. 2020. doi:10.1101/2020.06.24.20139303

1. Toro L, Parra A, Alvo M. [COVID-19 epidemic in chile: impact on emergency services care and specific pathologies]. Epidemia COVID-19 en Chile impacto en atenciones Serv Urgenc y Patol Especificas. 2020;148: 558–560. doi:https://dx.doi.org/10.4067/s0034-98872020000400558
2. Torre D, Pibaque R, Veloz T, Beltrán P, Baldeón L. In-House Development, Standardization, and Validation of a RT-qPCR Assay for the Detection of SARS-CoV-2 Virus in Ecuador. ResearchSquare. 2020. doi:10.21203/rs.3.rs-91429/v1
3. Torrealba-Rodriguez O, Conde-Gutiérrez RA, Hernández-Javier AL. Modeling and prediction of COVID-19 in Mexico applying mathematical and computational models. Chaos Solitons Fractals. 2020;138: 109946. doi:10.1016/j.chaos.2020.109946
4. Torres JP, Izquierdo G, Acuna M, Pavez D, Reyes F, Fritis A, et al. Multisystem inflammatory syndrome in children (MIS-C): Report of the clinical and epidemiological characteristics of cases in Santiago de Chile during the SARS-CoV-2 pandemic. Int J Infect Dis. 2020;100: 75–81. doi:http://dx.doi.org/10.1016/j.ijid.2020.08.062
5. Torres JP, Piñera C, De La Maza V, Lagomarcino AJ, Simian D, Torres B, et al. SARS-CoV-2 antibody prevalence in blood in a large school community subject to a Covid-19 outbreak: a cross-sectional study. Clin Infect Dis. 2020. doi:10.1093/cid/ciaa955
6. Torres R, Soares W, Ohashi O, Pessin G. The quest for better machine learning models to forecast COVID-19-related infections: A case study in the state of Pará-Brazil. ResearchSquare. 2020. doi:10.21203/rs.3.rs-37547/v1
7. Torres TS, Hoagland B, Bezerra DRB, Garner A, Jalil EM, Coelho LE, et al. Impact of COVID-19 Pandemic on Sexual Minority Populations in Brazil: An Analysis of Social/Racial Disparities in Maintaining Social Distancing and a Description of Sexual Behavior. AIDS Behav. 2020. doi:10.1007/s10461-020-02984-1
8. Torres-Roman JS, Kobiak IC, Valcarcel B, Diaz-Velez C, La Vecchia C. The reproductive number R0 of COVID-19 in Peru: An opportunity for effective changes. Travel Med Infect Dis. 2020; 101689. doi:https://dx.doi.org/10.1016/j.tmaid.2020.101689
9. Trudeau JM, Alicea-Planas J, Vasquez WF. The value of COVID-19 tests in Latin America. Econ Hum Biol. 2020;39: 100931. doi:http://dx.doi.org/10.1016/j.ehb.2020.100931
10. Trujillo A, Salazar F, Torres R, Arias P, Sotomayor K. An integer programming model to assign patients based on mental health impact for tele-psychotherapy intervention during the Covid–19 emergency. ResearchSquare. 2020. doi:10.21203/rs.3.rs-53836/v1
11. Turner S, Niño N. Qualitative Analysis of the Coordination of Major System Change Within the Colombian Health System in Response to COVID-19: Study Protocol. ResearchSquare. 2020. doi:10.21203/rs.3.rs-46942/v1
12. Ugas-Charcape C, Ucar M, Almanza-Aranda J, Rizo-Patrón E, Rantes C, Caro-Domínguez P, et al. Pulmonary Imaging Findings in Coronavirus Disease 2019 (COVID-19): A Series of 140 Latin American Children. ResearchSquare. 2020. doi:10.21203/rs.3.rs-94820/v1
13. Ugliara Barone MT, Harnik SB, Chaluppe M, Vieira de Luca P, Ngongo B, Pedrosa HC, et al. Decentralized COVID-19 measures in Brazil were ineffective to protect people with diabetes. Diabetes Metab Syndr Clin Res Rev. 2020;14: 1973–1978. doi:http://dx.doi.org/10.1016/j.dsx.2020.10.005
14. Urban RC, Nakada LYK. COVID-19 pandemic: Solid waste and environmental impacts in Brazil. Sci Total Environ. 2021;755: 142471. doi:http://dx.doi.org/10.1016/j.scitotenv.2020.142471
15. valderilio F azevedo, leiticia chaffin Barbosa P, Gabriel mondin N, faissal Nemer hajar, guilherme kyuzaemon Osako N, leonardo krieger rafael, et al. STRATEGY FOR THE CONTAINMENT, MITIGATION, AND SUPPRESSION OF THE COVID-19 PANDEMIC IN FRAGILIZED COMMUNITIES ON THE PERIPHERY OF A LARGE BRAZILIAN CITY. medRxiv. 2020. doi:10.1101/2020.09.28.20203174
16. Valente TM, Ferreira LPS, Silva RAD, Leite JMRS, Tiraboschi FA, Barboza MCC. Brazil Covid-19: Change of hospitalizations and deaths due to burn injury? Burns. 2020. doi:http://dx.doi.org/10.1016/j.burns.2020.10.009
17. Valenti VE, Menezes P de L, Abreu ACG de, Vieira GNA, Garner DM. Social distancing measures could have reduced estimated deaths related to COVID-19 in Brazil. J Hum Growth Dev. 2020;30: 164–169.
18. Valentina V, Milva G, Juan C, Ezequiel J. Incubation period and serial interval of Covid-19 in a chain of infections in Bahia Blanca (Argentina). medRxiv. 2020. doi:10.1101/2020.06.18.20134825
19. Valenzuela-Rodriguez G, Zambrano LI, Munoz-Lara F, Pecho-Silva S, Arteaga-Livias K, Rodriguez-Morales AJ. Intranational differences in the case fatality rates for COVID-19 among Peruvian physicians. Int J Infect Dis. 2020;101: 226–227. doi:http://dx.doi.org/10.1016/j.ijid.2020.09.018
20. Valeria Oliveira S, Elaine Lopes de O, Marcia Jorge C, Rosemeire Y, Cintia Mayumi A, Giselle Ibette L-L, et al. PREVALENCE OF ANTIBODIES AGAINST SARS-CoV-2 IN PROFESSIONALS OF A PUBLIC HEALTH LABORATORY AT SAO PAULO, SP, BRAZIL. medRxiv. 2020. doi:10.1101/2020.10.19.20213421
21. Valerio M, Miguel Q. An estimate of the COVID-19 infection fatality rate in Brazil based on a seroprevalence survey. medRxiv. 2020. doi:10.1101/2020.08.18.20177626
22. Valle JAM. Predicting the number of total COVID-19 cases and deaths in Brazil by the Gompertz model. Nonlinear Dyn. 2020; 1–7. doi:10.1007/s11071-020-06056-w
23. Vancini RL, Camargo-Neto L, de Lira CAB, Andrade MS, Viana RB, Nikolaidis PT, et al. Physical Activity and Sociodemographic Profile of Brazilian People during COVID-19 Outbreak: An Online and Cross-Sectional Survey. Int J Environ Res Public Health. 2020;17. doi:http://dx.doi.org/10.3390/ijerph17217964
24. Vandormael A, Adam M, Greuel M, Barnighausen T. A short, animated video to improve good COVID-19 hygiene practices: a structured summary of a study protocol for a randomized controlled trial. Trials. 2020;21: 469. doi:https://dx.doi.org/10.1186/s13063-020-04449-1
25. Varotsos CA, Krapivin VF. A new model for the spread of COVID-19 and the improvement of safety. Saf Sci. 2020; 104962. doi:10.1016/j.ssci.2020.104962
26. Vasconcelos GL, Brum AA, Duarte-Filho GC, Ospina R, Almeida FAG, Macêdo AMS. OVERVIEW OF THE COVID-19 IN NORTHEAST BRAZIL: ANALYSES AND FORECASTS VIA GROWTH MODELS. 2020.
27. Vasconcelos GL, Duarte-Filho GC, Brum AA, Ospina R, Almeida FAG, Macêdo AMS. Analysis of Covid-19 epidemic curves via generalized growth models: Case study for the cities of Recife and Teresina. 2020.
28. Vasquez L, Sampor C, Villanueva G, Maradiegue E, Garcia-Lombardi M, Gomez-Garcia W, et al. Early impact of the COVID-19 pandemic on paediatric cancer care in Latin America. Lancet Oncol. 2020;21: 753–755. doi:https://dx.doi.org/10.1016/S1470-2045(20)30280-1
29. Vasquez-Apestegui V, Parras-Garrido E, Tapia V, Paz-Aparicio VM, Rojas JP, Sánchez-Ccoyllo OR, et al. Association Between Air Pollution in Lima and the High Incidence of COVID-19: Findings from a Post Hoc Analysis. Res Sq. 2020. doi:10.21203/rs.3.rs-39404/v1
30. Vasquez-Hoyos P, Diaz-Rubio F, Monteverde-Fernandez N, Jaramillo-Bustamante JC, Carvajal C, Serra A, et al. Reduced PICU respiratory admissions during COVID-19. Arch Dis Child. 2020; archdischild-2020. doi:http://dx.doi.org/10.1136/archdischild-2020-320469
31. Vaz SN, Santana DSD, Netto EM, Pedroso C, Wang WK, Santos FDA, et al. Saliva is a reliable, non-invasive specimen for SARS-CoV-2 detection. Brazilian J Infect Dis. 2020;24: 422–427. doi:http://dx.doi.org/10.1016/j.bjid.2020.08.001
32. Vecchio G, Zapico V, Catanzariti A, Bisso IC, Heras ML. Adverse effects of lopinavir/ritonavir in critically ill patients with COVID-19. Med. 2020;80: 439–441.
33. Veiga E Silva L, de Andrade Abi Harb MDP, Teixeira Barbosa Dos Santos AM, de Mattos Teixeira CA, Macedo Gomes VH, Silva Cardoso EH, et al. COVID-19 Mortality Underreporting in Brazil: Analysis of Data From Government Internet Portals. J Med Internet Res. 2020;22: e21413. doi:https://dx.doi.org/10.2196/21413
34. Veiga ESL, de Andrade Abi Harb MDP, Teixeira Barbosa Dos Santos AM, de Mattos Teixeira CA, Macedo Gomes VH, Silva Cardoso EH, et al. An analysis of COVID-19 mortality underreporting based on data available from official Brazilian government internet portals. J Med Internet Res. 2020;22: e21413. doi:10.2196/21413
35. Vejar-Aguirre T, Jáuregui-Ulloa E, Gallo-Sánchez K, Mejía-García JA, Zavala-Hernández R, Arredondo A. [Evidences and trends for decision making about measures of containment and mitigation of Covid-19 in Jalisco, Mexico]. Salud Publica Mex. 2020. doi:10.21149/11724
36. Vera-Hernandez CJ, Mendias-Alarcon A, Castillo-Olea C. 81 Implementing a Telehealth System in Baja California, Mexico to Address COVID-19 Pandemic. Ann Emerg Med. 2020;76: S32. doi:http://dx.doi.org/10.1016/j.annemergmed.2020.09.091
37. Vera-Ponce VJ, Torres-Malca JR, Tello-Quispe EK, Orihuela-Manrique EJ, De La Cruz-Vargas JA. Validation of scale of changes in lifestyles during the quarantine period in a population of university students from lima, peru. Rev la Fac Med Humana. 2020;20: 614–623. doi:http://dx.doi.org/10.25176/RFMH.V20I4.3193
38. Vicente CR, Sant’Ana MCS, Reis AP, Costa CF, Cerutti Junior C, Lanes CM, et al. Increasing Demand for Chest CT Due to COVID-19 in Brazil. Rev Soc Bras Med Trop. 2020;53: e20200608. doi:http://dx.doi.org/10.1590/0037-8682-0608-2020
39. Victor D, Abayuba P, Enrique S, Alvaro N, Natalia P, Alejandro C, et al. Impact of COVID-19 Outbreak on Quality of Care for STEMI in Uruguay: A Whole Country Population Retrospective Study. SSRN. 2020. doi:10.2139/ssrn.3631297
40. Victor JS, Diana F-L, Brian RZ, Eduardo R. Environmental and climatic impact on the infection and mortality of SARS-CoV-2 in Peru. medRxiv. 2020. doi:10.1101/2020.09.16.20196170
41. Víctor SP. Preparados para un virus nocturno: coronavirus, decisiones de política pública y vulneración de derechos en cinco municipios de Sonora, México. Región y Soc. 2020;32. doi:10.22198/RYS2020/32/1324
42. Victoria Izabela C, Carlos M. Percepción de riesgo y consumo mediático durante el inicio de la pandemia de COVID-19 en México. 2020.
43. Victoria E, Gordon WH, Batsirai M, Sabine S, Paul Z, Christian M, et al. SARS-CoV-2 genomic characterization and clinical manifestation of the COVID-19 outbreak in Uruguay. medRxiv. 2020. doi:10.1101/2020.10.08.20208546
44. Vieira MA da CES, Vieira CP de B, Borba A de S, Melo MC de C, Oliveira M de S, Melo RM, et al. Sequential serological surveys in the early stages of the coronavirus disease epidemic: limitations and perspectives. Rev Soc Bras Med Trop. 2020;53: e20200351. doi:https://dx.doi.org/10.1590/0037-8682-0351-2020
45. Vieira-Meyer APGF, Coutinho MB, Guerra HP, Saintrain M V, Candeiro GTM. Brazilian primary and secondary public oral health attention: are dentists ready to face COVID-19 Pandemic? Disaster Med Public Health Prep. 2020; 1–22. doi:http://dx.doi.org/10.1017/dmp.2020.342
46. Vilar-Compte M, Perez V, Teruel G, Alonso A, Perez-Escamilla R. Costing of actions to safeguard vulnerable Mexican households with young children from the consequences of COVID-19 social distancing measures. Int J Equity Health. 2020;19: 70. doi:https://dx.doi.org/10.1186/s12939-020-01187-3
47. Villarreyes E, Luna A, Soriano A. Estimating the number of deaths due to COVID-19 in Lima and Peru during March and April 2020 using ARIMA time series and modeling. ResearchSquare. 2020. doi:10.21203/rs.3.rs-62317/v1
48. Villela D. How limitations in data of health surveillance impact decision making in the COVID-19 epidemic. 2020.
49. Villela E, Lopez R, Sato A, Oliveira F, Waldman E, Bergh R, et al. COVID-19 outbreak in Brazil: Adherence to national preventive measures and impact on people’s lives. ResearchSquare. 2020. doi:10.21203/rs.3.rs-40186/v1
50. Vinícius Carvalho J, Marcos Silveira B. Análise sistêmica do município de São Paulo e suas implicações para o avanço dos casos de Covid-19. 2020;34. doi:10.1590/S0103-4014.2020.3499.010
51. Vinueza-Veloz AF, Aldaz-Pachacama NR, Mera-Segovia CM, Pino-Vaca DP, Tapia-Veloz EC, Vinueza-Veloz MF. Burnout syndrome among Ecuadorian medical doctors and nurses during COVID-19 pandemic. 2020.
52. Viviana Guzzo L, Maria Sanali Moura P, Giordana Zeferino M, Marcelo Antônio Cartaxo Queiroga L, Ricardo Alves da C, Gláucia Maria Moraes de O. Brazilian Cardiology Interventional Registry during COVID-19 pandemic (RBCI-COVID19). 2020;28. doi:10.31160/JOTCI202028A202010
53. Viviane Alves Dos Santos B, Roseane Christhina da Nova S-S, Maria Da Penha De Lima C, Emerson Araújo Do B, Maria Edna Silva De A. Representations and social anchorages of the new coronavirus and the COVID-19 treatment by Brazilians/ Representações e ancoragens sociais do novo coronavírus e do tratamento da COVID-19 por brasileiros. Estud Psicol. 2020.
54. Vojvodic Hernández I, Ortega Checa D, Pinares Carrillo D, Alemán López J, Aburto Loroña V. Operaciones abdominopélvicas de emergencia en pacientes con COVID-19 en el Hospital Edgardo Rebagliati Martins - EsSalud, Lima, Perú. Rev colomb cir. 2020;35: 414–421.
55. von Humboldt S, Mendoza-Ruvalcaba NM, Arias-Merino ED, Costa A, Cabras E, Low G, et al. Smart technology and the meaning in life of older adults during the Covid-19 public health emergency period: a cross-cultural qualitative study. Int Rev Psychiatry. 2020. doi:http://dx.doi.org/10.1080/09540261.2020.1810643
56. Wachholz PA, Moreira VG, Oliveira D, Watanabe HAW, Villas Boas PJF. OCORRÊNCIA DE INFECÇÃO E MORTALIDADE POR COVID-19 EM RESIDENCIAIS PARA IDOSOS NO BRASIL. 2020.
57. Wainer P, Saavedra F, Tagliapietra V, Abeledo D, Migliore D, Lapadula P, et al. [COVID-19 experience in a private institution in Buenos Aires during the first month of the pandemic: 26 cases]. Medicina (B Aires). 2020;80: 193–196.
58. Waldecy  Sr. R, David Nadler  Sr. P, Wainesten  Sr. C. REGIONAL DETERMINANTS OF THE EXPANSION OF COVID-19 IN BRAZIL. medRxiv. 2020. doi:10.1101/2020.04.13.20063925
59. Wang P, Zheng X, Ai G, Liu D, Zhu B. Time series prediction for the epidemic trends of COVID-19 using the improved LSTM deep learning method: Case studies in Russia, Peru and Iran. Chaos Solitons Fractals. 2020;140: 110214. doi:10.1016/j.chaos.2020.110214
60. Wang P, Zheng X, Li J, Zhu B. Prediction of epidemic trends in COVID-19 with logistic model and machine learning technics. Chaos Solitons Fractals. 2020;139: 110058. doi:10.1016/j.chaos.2020.110058
61. Wang Y, Xu C, Yao S, Zhao Y. Forecasting the epidemiological trends of COVID-19 prevalence and mortality using the advanced alpha-Sutte Indicator. Epidemiol Infect. 2020;148: e236. doi:http://dx.doi.org/10.1017/S095026882000237X
62. Wassenaar TM, Buzard GS, Newman DJ. BCG vaccination early in life does not improve COVID-19 outcome of elderly populations, based on nationally reported data. Lett Appl Microbiol. 2020. doi:10.1111/lam.13365
63. Wendel S, Kutner JM, Machado R, Fontao-Wendel R, Bub C, Fachini R, et al. Screening for SARS-CoV-2 antibodies in convalescent plasma in Brazil: Preliminary lessons from a voluntary convalescent donor program. Transfusion. 2020. doi:http://dx.doi.org/10.1111/trf.16065
64. Werberich GM, Marchiori E, Barreto MM, Rodrigues RS. Computed tomography findings in a Brazilian cohort of 48 patients with pneumonia due to coronavirus disease. Rev Soc Bras Med Trop. 2020;53. doi:10.1590/0037-8682-0405-2020
65. Werneck AO, Silva DRD, Malta DC, Souza-Junior PRB, Azevedo LO, Barros MBA, et al. Lifestyle behaviors changes during the COVID-19 pandemic quarantine among 6,881 Brazilian adults with depression and 35,143 without depression. Cien Saude Colet. 2020;25: 4151–4156. doi:http://dx.doi.org/10.1590/1413-812320202510.2.27862020
66. Werneck AO, Silva DR, Malta DC, Gomes CS, Souza-Junior PRB, Azevedo LO, et al. Associations of sedentary behaviors and incidence of unhealthy diet during the COVID-19 quarantine in Brazil. Public Health Nutr. 2020; 1–12. doi:http://dx.doi.org/10.1017/S1368980020004188
67. Werneck AO, Silva DR, Malta DC, Lima MG, Souza-Junior PRB, Azevedo LO, et al. The mediation role of sleep quality in the association between the incidence of unhealthy movement behaviors during the COVID-19 quarantine and mental health. Sleep Med. 2020;76: 10–15. doi:http://dx.doi.org/10.1016/j.sleep.2020.09.021
68. Werneck AO, Silva DR, Malta DC, Souza-Junior PRB, Azevedo LO, Barros MBA, et al. Changes in the clustering of unhealthy movement behaviors during the COVID-19 quarantine and the association with mental health indicators among Brazilian adults. Transl Behav Med. 2020. doi:http://dx.doi.org/10.1093/tbm/ibaa095
69. Werneck AO, Silva DR, Malta DC, Souza-Júnior PRB, Azevedo LO, Barros MBA, et al. Physical inactivity and elevated TV-viewing reported changes during the COVID-19 pandemic are associated with mental health: A survey with 43,995 Brazilian adults. J Psychosom Res. 2021;140: 110292.
70. Wesley  Sr. D, Alcides Castro e S, Roger  Sr. G, Ian M-F, Servio Pontes R. COVID-19 most vulnerable Mexican cities lack the public health infrastructure to face the pandemic: a new temporally-explicit model. medRxiv. 2020. doi:10.1101/2020.04.10.20061192
71. Wilches Visbal JH, Castillo Pedraza MC. [Mathematical approach of the SIR epidemiological model for the comprehension of the containment measures against the Covid-19.]. Aproximacion Mat del Model Epidemiol SIR para la comprension las medidas contencion contra la COVID-19. 2020;94.
72. William JA-B, Aveiro R, Telmo R, Fernández, María F, Diana C-P, et al. Progresión de casos de Coronavirus en Latinoamérica: Análisis comparativo a una semana de iniciada la pandemia en cada país. Kasmera. 2020;48.
73. Willy R, Juan A, Jhony ADLC-V, Luis O, Maria V, Yovanna S, et al. Epidemiological characteristics of deaths from COVID-19: an analysis at almost three months of the first confirmed case in Peru. medRxiv. 2020. doi:10.1101/2020.11.05.20226639
74. Wollenstein-Betech S, Silva AAB, Fleck JL, Cassandras CG, Paschalidis IC. Physiological and socioeconomic characteristics predict COVID-19 mortality and resource utilization in Brazil. PLoS One. 2020;15: e0240346. doi:http://dx.doi.org/10.1371/journal.pone.0240346
75. Wollenstein-Betech S, Cassandras CG, Paschalidis IC. Personalized Predictive Models for Symptomatic COVID-19 Patients Using Basic Preconditions: Hospitalizations, Mortality, and the Need for an ICU or Ventilator. medRxiv  Prepr Serv Heal Sci. 2020. doi:https://dx.doi.org/10.1101/2020.05.03.20089813
76. Ximenes Neto FRG, Araújo CR de C e, Silva RCC da, Ribeiro MA, Sousa LA de, Serafim TF, et al. Coordination of care, surveillance and monitoring of cases of COVID-19 in primary health care. Enferm foco. 2020;11: 239–245.
77. Xu C, Dong Y, Yu X, Wang H, Tsamlag L, Zhang S, et al. Estimation of reproduction numbers of COVID-19 in typical countries and epidemic trends under different prevention and control scenarios. Front Med. 2020. doi:https://dx.doi.org/10.1007/s11684-020-0787-4
78. Yáñez JA, Afshar Jahanshahi A, Alvarez-Risco A, Li J, Zhang SX. Anxiety, Distress, and Turnover Intention of Healthcare Workers in Peru by Their Distance to the Epicenter during the COVID-19 Crisis. Am J Trop Med Hyg. 2020. doi:10.4269/ajtmh.20-0800
79. Yang HM, Lombardi Junior LP, Castro FFM, Yang AC. Mathematical model describing CoViD-19 in Sao Paulo, Brazil - evaluating isolation as control mechanism and forecasting epidemiological scenarios of release. Epidemiol Infect. 2020;148: e155. doi:https://dx.doi.org/10.1017/S0950268820001600

Hyun Mo Y, Luis Pedro Lombardi J, Fabio Fernandes Morato C, Ariana Campos Y. Mathematical model describing CoViD-19 in Sao Paulo State, Brazil - Evaluating isolation as control mechanism and forecasting epidemiological scenarios of release. medRxiv. 2020. doi:10.1101/2020.04.29.20084830

1. Yaranay L-A, Javier M-N, Fabiola S-D, Rodolfo Á-J. La Inteligencia emocional en apoderados chilenos confinados por la COVID-19. 2020;9. doi:10.33210/CA.V9I2.320
2. Yasmany Damián G-R, María Soledad S-M, Belizario Amador Z-T, Marlon Agusto C-R. Relación entre las restricciones del tránsito vehicular y las tendencias del COVID-19: un caso de estudio ecuatoriano. 2020;9. doi:10.33210/CA.V9I2.308
3. Yige L, Eduardo AU, Jose Ramon Z. Efficacy of Localized Lockdowns in the SARS-CoV-2 Pandemic. medRxiv. 2020. doi:10.1101/2020.08.25.20182071
4. Yomayusa N, Acevedo K, Reina A, Rincón K, Toloza C, Gomez O, et al. Clinical course, biomarkers, management and outcomes of patients hospitalised due to COVID-19 in Colombia. ResearchSquare. 2020. doi:10.21203/rs.3.rs-57978/v1
5. You C, Yubo F, Chao Y, Xinmeng Z, Cheng G. Modeling COVID-19 Growing Trends to Reveal the Differences in the Effectiveness of Non-Pharmaceutical Interventions among Countries in the World. medRxiv. 2020. doi:10.1101/2020.04.22.20075846
6. Yunier B-P, Zoylen F-F, Elizabeth J-P, Evelyn F-C, Boris CR-M. Gender and Fear of COVID-19 in a Cuban Population Sample. Int J Ment Health Addict. 2020. doi:10.1007/S11469-020-00343-8
7. Yuri Castro R, Olenka Valenzuela T. Repercusiones de la pandemia de COVID 19 en la atención odontológica, una perspectiva de los odontólogos clínicos. 2020;19.
8. Zambrano-Monserrate MA, Ruano MA. Has air quality improved in Ecuador during the COVID-19 pandemic? A parametric analysis. Air Qual Atmos Health. 2020; 1–10. doi:10.1007/s11869-020-00866-y
9. Zavala-Flores E, Salcedo-Matienzo J. Pre-hospitalary medication in COVID-19 patients from a public hospital in Lima-Peru. Acta Medica Peru. 2020;37: 393–395. doi:http://dx.doi.org/10.35663/amp.2020.373.1277
10. Zavarce Castillo C, Zavarce Castillo F. Modeling the trend and seasonal components of the SARS-COV2 in the Bolivarian Republic of Venezuela. Obs del Conoc Rev Espec Gestión Soc del Conoc. 2020;5: 46–56.
11. Zavarce Castillo C, Zavarce Castillo F. Stochastic behavior of covid-19 in the Bolivarian Republic of Venezuela: persistence or antipersistence in infections? Obs del Conoc Rev Espec en Gestión Soc del Conoc. 2020;5: 91–110.
12. Zhang SX, Sun S, Afshar Jahanshahi A, Alvarez-Risco A, Ibarra VG, Li J, et al. Developing and testing a measure of COVID-19 organizational support of healthcare workers - results from Peru, Ecuador, and Bolivia. Psychiatry Res. 2020;291: 113174. doi:https://dx.doi.org/10.1016/j.psychres.2020.113174
13. Zhiwei D, Feng S, Bingyu L, Jing K, Yi Z, Yip P, et al. Predicting the Impact of the COVID-19 Pandemic for the Low- and Middle-Income Countries. medRxiv. 2020. doi:10.1101/2020.08.13.20167064
14. Zhu D, Mishra SR, Han X, Santo K. Social distancing in Latin America during the COVID-19 pandemic: an analysis using the Stringency Index and Google Community Mobility Reports. J Travel Med. 2020. doi:10.1093/jtm/taaa125
15. Zhu L, Liu X, Huang H, Avellan-Llaguno RD, Lazo MML, Gaggero A, et al. Meteorological impact on the COVID-19 pandemic: A study across eight severely affected regions in South America. Sci Total Environ. 2020;744: 140881. doi:https://dx.doi.org/10.1016/j.scitotenv.2020.140881
16. Zimman S, Cura MJ, Luna PC, Echeverria CM, Mazzuoccolo LD. Impact of the COVID-19 Pandemic on Immunomodulatory and Immunosuppressive Therapies in Dermatology: Patient and Physician Attitudes in Argentina. Actas Dermosifiliogr. 2020. doi:https://dx.doi.org/10.1016/j.ad.2020.04.004
17. Zimmerer KS, de Haan S. Informal food chains and agrobiodiversity need strengthening-not weakening-to address food security amidst the COVID-19 crisis in South America. Food Secur. 2020; 1–4. doi:10.1007/s12571-020-01088-x
18. Zimmermann IR, Sanchez M, Brant J, Alves D. Demand for ICU beds by COVID-19 in the Federal District, Brazil: an analysis of the impact of social distance measures with Monte Carlo simulations. 2020.
19. Zoilo Emilio  Sr. G-B, Kiero  Sr. G-P, Vahid Vahid Nouri K, Maria Isabel M, Luis Eduardo  Sr. G, Luisa Marilia C-G, et al. COVID-19 pandemic and health worker stress: The mediating effect of emotional regulation. medRxiv. 2020. doi:10.1101/2020.06.19.20135574
20. Zuniga-Moya JC, Norwood DA, Romero Reyes LE, Barrueto Saavedra E, Diaz R, Fajardo WC, et al. Epidemiology, outcomes and associated factors of COVID-19 RT-PCR confirmed cases in the San Pedro Sula Metropolitan Area, Honduras. Clin Infect Dis. 2020. doi:10.1093/cid/ciaa1188
21. Zylberman V, Sanguineti S, Pontoriero A V, Higa S V, Cerutti ML, Morrone Seijo SM, et al. Development of a hyperimmune equine serum therapy for COVID-19 in Argentina. Desarro un suero equino hiperinmune para el Trat COVID-19 en Argentina. 2020;80 Suppl 3: 1–6.

## Protocol registry of included studies

1. Efficacy and Safety Study of Nitazoxanide (NTX) in the Treatment of Patients With SARS-CoV-2 Virus Infection (COVID-19). 2020. Available in https://clinicaltrials.gov/ct2/show/NCT04463264
2. Efficacy Assessment of Methylprednisolone and Heparin in Patients With COVID-19 Pneumonia. 2020. Available in https://clinicaltrials.gov/ct2/show/NCT04485429
3. Efficacy of Ivermectin in Adult Patients With Early Stages of COVID-19. 2020. Available in https://clinicaltrials.gov/ct2/show/NCT04405843
4. Hydroxychloroquine and Ivermectin for the Treatment of COVID-19 Infection. 2020. Available in https://clinicaltrials.gov/ct2/show/NCT04391127
5. Randomized Clinical Trial of Intranasal Dexamethasone as an Adjuvant in Patients With COVID-19. 2020. Available in https://clinicaltrials.gov/ct2/show/NCT04513184
6. A clinical trial to study the effects of additional treatments for patients hospitalized and receiving treatment due to COVID -19.. 2020. Available in http://www.ctri.nic.in/Clinicaltrials/pmaindet2.php?trialid=42897
7. A clinical, randomized study to evaluate the efficacy and safety of Naproxen compared to placebo in combination with Azithromycin or Levofloxacin in patients with Severe Acute Respiratory Syndrome during the Covid-19 pandemic. 2020. Available in http://www.ensaiosclinicos.gov.br/rg/RBR-3rywwg/
8. A Phase 1b Trial to Evaluate Safety and Effect of SAR443122 on Immune System in Severe COVID-19. 2020. Available in https://clinicaltrials.gov/show/NCT04469621
9. A Phase 2, Open-Label, Randomized Study of the Efficacy and Safety of Acalabrutinib with Best Supportive Care Versus Best Supportive Care in Subjects Hospitalized with COVID-19. 2020. Available in https://www.clinicaltrialsregister.eu/ctr-search/search?query=eudract_number:2020-001644-25
10. A PHASE 2/3, RANDOMIZED, DOUBLE-BLIND, PLACEBO-CONTROLLED STUDY TO EVALUATE THE EFFICACY AND SAFETY OF MAVRILIMUMAB (KPL-301) TREATMENT IN ADULT SUBJECTS HOSPITALIZED WITH SEVERE COVID-19 PNEUMONIA AND HYPER-INFLAMMATION. 2020. Available in https://www.ins.gob.pe/ensayosclinicos/rpec/recuperarECPBNuevoEN.asp?numec=032-20
11. A phase III study to investigate a vaccine against COVID-19. 2020. Available in http://isrctn.com/ISRCTN89951424
12. A Pragmatic Randomized Controlled Trial of Therapeutic Anticoagulation Versus Standard Care as a Rapid Response to COVID-19 Pandemic. 2020. Available in https://clinicaltrials.gov/show/NCT04444700
13. A RANDOMIZED, DOUBLE-BLIND, PLACEBO- CONTROLLED, MULTICENTER STUDY TO EVALUATE THE EFFICACY AND SAFETY OF TOCILIZUMAB IN HOSPITALIZED PATIENTS WITH COVID-19 PNEUMONIA. 2020. Available in https://www.ins.gob.pe/ensayosclinicos/rpec/recuperarECPBNuevoEN.asp?numec=027-20
14. A Study Looking at the Efficacy, Immune Response, and Safety of a COVID-19 Vaccine in Adults at Risk for SARS-CoV-2. 2020. Available in https://clinicaltrials.gov/ct2/show/NCT04611802
15. A Study of Ad26.COV2.S for the Prevention of SARS-CoV-2-Mediated COVID-19 in Adult Participants. 2020. Available in https://clinicaltrials.gov/show/NCT04505722
16. A Study of Baricitinib (LY3009104) in Participants With COVID-19. 2020. Available in https://clinicaltrials.gov/show/NCT04421027
17. A Study of Immune Responses to the Virus That Causes COVID-19. 2020. Available in https://clinicaltrials.gov/show/NCT04431414
18. A Study to Compare the Efficacy and Safety of Different Doses of Ivermectin for COVID-19. 2020. Available in https://clinicaltrials.gov/ct2/show/NCT04431466
19. A Study to Evaluate Efficacy and Safety of PTC299 in Hospitalized Participants With Coronavirus (COVID-19). 2020. Available in https://clinicaltrials.gov/show/NCT04439071
20. A Study to Evaluate the Efficacy and Safety of Remdesivir Plus Tocilizumab Compared With Remdesivir Plus Placebo in Hospitalized Participants With Severe COVID-19 Pneumonia. 2020. Available in https://clinicaltrials.gov/show/NCT04409262
21. A Study to Evaluate the Efficacy and Safety of Sirukumab in Confirmed Severe or Critical Confirmed Coronavirus Disease (COVID)-19. 2020. Available in https://clinicaltrials.gov/show/NCT04380961
22. A Study to Evaluate the Efficacy and Safety of Tocilizumab in Hospitalized Participants With COVID-19 Pneumonia. 2020. Available in https://clinicaltrials.gov/show/NCT04372186
23. A Study to Evaluate the Safety and Efficacy of MSTT1041A (Astegolimab) or UTTR1147A in Patients With Severe COVID-19 Pneumonia. 2020. Available in https://clinicaltrials.gov/show/NCT04386616
24. A Study to Evaluate the Safety and Efficacy of MSTT1041A or UTTR1147A in Patients with Severe Covid-19 Pneumonia. 2020. Available in https://www.clinicaltrialsregister.eu/ctr-search/search?query=eudract_number:2020-002713-17
25. A Study to Evaluate the Safety, Pharmacokinetics and Antiviral Effects of Galidesivir in Yellow Fever or COVID-19. 2020. Available in https://clinicaltrials.gov/show/NCT03891420
26. A Study to Investigate the Pharmacokinetics, Efficacy and Safety of INM005 in Patients With COVID-19.. 2020. Available in https://clinicaltrials.gov/show/NCT04494984
27. A Survey to Assess Effects of Covid-19 on Cardiovascular Patients. 2020. Available in https://clinicaltrials.gov/show/NCT04508920
28. ABX464 in Treating Inflammation and Preventing Acute Respiratory Failure in Patients With COVID-19. 2020. Available in https://clinicaltrials.gov/show/NCT04393038
29. Acalabrutinib Study With Best Supportive Care Versus Best Supportive Care in Subjects Hospitalized With COVID-19.. 2020. Available in https://clinicaltrials.gov/show/NCT04346199
30. Acute and chronic cardiovascular and respiratory changes in COVID-19 hospitalized patients and the effect of physical rehabilitation supervised by telecommunication. 2020. Available in http://www.ensaiosclinicos.gov.br/rg/RBR-9y32yy/
31. Acute Encephalopathy in Critically Ill Patients With COVID-19. 2020. Available in https://clinicaltrials.gov/show/NCT04320472
32. Acute impairment of renal function in COVID-19: study on incidence, risk factors and mortality. 2020. Available in http://www.ensaiosclinicos.gov.br/rg/RBR-62y3h7/
33. Adaptive COVID-19 Treatment Trial (ACTT). 2020. Available in https://clinicaltrials.gov/show/NCT04280705
34. Advanced Endoscopy During COVID-19. 2020. Available in https://clinicaltrials.gov/show/NCT04385147
35. An international randomised trial of additional treatments for COVID-19 in hospitalised patients who are all receiving the local standard of care. 2020. Available in https://www.clinicaltrialsregister.eu/ctr-search/search?query=eudract_number:2020-001366-11
36. Analysis of the Inflammatory Response and the Development of Humoral and Cellular Immunity in Patients With COVID-19. 2020. Available in https://clinicaltrials.gov/show/NCT04423640
37. Analytical study of the profile of pro-inflammatory and pro-resolution factors in non-obese males with pulmonary symptomatology resulting from SARS Covid-19 verified with PCR blood analysis.. 2020. Available in http://www.drks.de/DRKS00022337
38. Anti-Androgen Treatment for COVID-19. 2020. Available in https://clinicaltrials.gov/show/NCT04446429
39. Antithrombotic Therapy to Ameliorate Complications of COVID-19 ( ATTACC ). 2020. Available in https://clinicaltrials.gov/show/NCT04372589
40. Antiviral Agents Against COVID-19 Infection. 2020. Available in https://clinicaltrials.gov/show/NCT04468087
41. Argentinian Registry of Patients With Rheumatic Diseases and COVID-19 Infection. 2020. Available in https://clinicaltrials.gov/ct2/show/NCT04568421
42. Assessment cardiorespiratory in recovered Covid-19 patients. 2020. Available in http://www.ensaiosclinicos.gov.br/rg/RBR-6xqcr4/
43. Assessment of Seroprevalence and Incidence of SARS-CoV-2 Infection in Brazil. 2020. Available in https://clinicaltrials.gov/ct2/show/NCT04355338
44. Association of the Neutrophil/Lymphocyte Ratio With Clinical Complications and Mortality in COVID-19 Patients. 2020. Available in https://clinicaltrials.gov/ct2/show/NCT04434157
45. ATENEA-Co-300 study. 2020. Available in https://rpcec.sld.cu/en/trials/RPCEC00000317-En
46. Awake Prone Positioning and Oxygen Therapy in Patients With COVID-19. 2020. Available in https://clinicaltrials.gov/show/NCT04407468
47. BCG vaccine use to prevent COVID-19 in health care workers. 2020. Available in http://www.ensaiosclinicos.gov.br/rg/RBR-5ysj54/
48. Biomodulina T - IM- covid-19 - older adultos - Phase IV clinical trial. 2020. Available in https://rpcec.sld.cu/en/trials/RPCEC00000310-En
49. Brief Cognitive Behavioral Therapy Versus Crisis Intervention Therapy Through Telepsychiatry on Psychiatric Symptoms. 2020. Available in https://clinicaltrials.gov/show/NCT04394455
50. Burnout and Distress preventiOn With caNnabidiol in Front-line Health Care workerS deAling wIth COVID-19. 2020. Available in https://clinicaltrials.gov/ct2/show/NCT04504877
51. CANnabiDiol for CoviD-19 pATiEnts With Mild to Moderate Symptoms. 2020. Available in https://clinicaltrials.gov/ct2/show/NCT04467918
52. Cerebral Compliance Impairment in COVID-19. 2020. Available in https://clinicaltrials.gov/show/NCT04429477
53. Characteristics of Patients and Healthcare Workers With COVID-19 in Meta State, Colombia. 2020. Available in https://clinicaltrials.gov/ct2/show/NCT04456426
54. Characterizing SARS-CoV-2-specific Immunity in Individuals Who Have Recovered From COVID-19. 2020. Available in https://clinicaltrials.gov/show/NCT04403880
55. Chloroquine + Losartan Compared to Chloroquine Alone for the Treatment of COVID-19 Pneumonia. 2020. Available in https://clinicaltrials.gov/show/NCT04428268
56. Chloroquine Diphosphate in the Prevention of SARS in Covid-19 Infection. 2020. Available in https://clinicaltrials.gov/show/NCT04342650
57. Chloroquine Phosphate Prophylactic Use in Health Personnel Exposed to COVID-19 Patients. 2020. Available in https://clinicaltrials.gov/show/NCT04443270
58. Cholecalciferol to Improve the Outcomes of COVID-19 Patients. 2020. Available in https://clinicaltrials.gov/show/NCT04411446
59. CIGB 2020 in contacts and SARS-CoV-2 infection suspects. 2020. Available in https://rpcec.sld.cu/en/trials/RPCEC00000306-En
60. Clinical Characteristics and Outcomes of 187 Critically Ill Patients With COVID-19. 2020. Available in https://clinicaltrials.gov/ct2/show/NCT04454372
61. Clinical Characteristics and Outcomes of Patients With COVID-19 on Mechanical Ventilation in Argentina. 2020. Available in https://clinicaltrials.gov/ct2/show/results/NCT04611269
62. Clinical Effect of Dialyzable Leukocyte Extract in Suspected or Confirmed Cases of COVID-19 (FUTURE-T). 2020. Available in https://clinicaltrials.gov/ct2/show/NCT04379479
63. Clinical Evaluation of Ventilador Innovation Product in Colombia in the SARS COVID 19 Pandemic, Unisabana Herons. 2020. Available in https://clinicaltrials.gov/ct2/show/NCT04497623
64. Clinical Impact of BACTEK-R in Subject With Mild Pneumonia Due to COVID-19 Infection. 2020. Available in https://clinicaltrials.gov/show/NCT04363814
65. Clinical trial for the treatment of COVID-19 with Chloroquine and Colchicine. 2020. Available in http://www.ensaiosclinicos.gov.br/rg/RBR-8jyhxh/
66. Clinical Trial to Evaluate the Efficacy, Immunogenicity and Safety of the Inactivated SARS-CoV-2 Vaccine (COVID-19). 2020. Available in https://clinicaltrials.gov/ct2/show/NCT04560881
67. Colchicine Twice Daily During 10 Days as an Option for the Treatment of Symptoms Induced by Inflammation in Patients With Mild and Severe Coronavirus Disease. 2020. Available in https://clinicaltrials.gov/show/NCT04367168
68. Combined Simultaneous EGD-colonoscopy Trial (CoSi Endoscopy). 2020. Available in https://clinicaltrials.gov/ct2/show/NCT04473456
69. Convalescent Plasma and Placebo for the Treatment of COVID-19 Severe Pneumonia. 2020. Available in https://clinicaltrials.gov/show/NCT04383535
70. CONVALESCENT PLASMA AS TREATMENT FOR COVID-19. 2020. Available in https://www.ins.gob.pe/ensayosclinicos/rpec/recuperarECPBNuevoEN.asp?numec=013-20
71. Convalescent Plasma Compared to Anti-COVID-19 Human Immunoglobulin and Standard Treatment (TE) in Hospitalized Patients. 2020. Available in https://clinicaltrials.gov/ct2/show/NCT04395170
72. Convalescent Plasma Compared to the Best Available Therapy for the Treatment of SARS-CoV-2 Pneumonia. 2020. Available in https://clinicaltrials.gov/show/NCT04358783
73. Convalescent Plasma for Patients With COVID-19: a Randomized, Open Label, Parallel, Controlled Clinical Study. 2020. Available in https://clinicaltrials.gov/ct2/show/NCT04332835
74. Convalescent Plasma for the Treatment of Severe SARS-CoV-2 (COVID-19). 2020. Available in https://clinicaltrials.gov/show/NCT04391101
75. Convalescent Plasma for Treating Patients With COVID-19 Pneumonia Without Indication of Ventilatory Support. 2020. Available in https://clinicaltrials.gov/ct2/show/NCT04528368
76. Convalescent Plasma of Covid-19 to Treat SARS-COV-2 a Randomized Doble Blind 2 Center Trial. 2020. Available in https://clinicaltrials.gov/show/NCT04405310
77. Convalescent Plasma vs Human Immunoglobulin to Treat COVID-19 Pneumonia. 2020. Available in https://clinicaltrials.gov/ct2/show/NCT04381858
78. CORE Study COVID-19. 2020. Available in https://clinicaltrials.gov/ct2/show/NCT04531202
79. CORONATION: Coronavirus (COVID-19) multinational observational registry. 2020. Available in http://isrctn.com/ISRCTN17717312
80. CoV-Hep study : Comparative study between different anti-coagulation strategies in continuous hemodialysis in COVID-19 patients. 2020. Available in http://www.ensaiosclinicos.gov.br/rg/RBR-45kf9p/
81. CoV-Hep Study: Regional Anticoagulation Modalities in Continuous Venous Venous Hemodialysis in Patients With COVID-19. 2020. Available in https://clinicaltrials.gov/show/NCT04487990
82. COVID-19 Antibodies Among Healthcare Workers. 2020. Available in https://clinicaltrials.gov/show/NCT04425889
83. COVID-19 Global Rheumatology Alliance. 2020. Available in http://www.ctri.nic.in/Clinicaltrials/pmaindet2.php?trialid=46228
84. COVID-19 in Immunosuppressed Children. 2020. Available in https://clinicaltrials.gov/show/NCT04511429
85. COVID-19 Prophylaxis With Hydroxychloroquine Associated With Zinc For High-Risk Healthcare Workers. 2020. Available in https://clinicaltrials.gov/show/NCT04384458
86. COVID-19 Serology Screening and Strict Protocol in the Endoscopy Unit. 2020. Available in https://clinicaltrials.gov/ct2/show/NCT04374123
87. COVID-19 Symptom Tracker. 2020. Available in http://www.drks.de/DRKS00021305
88. COVID-19: BCG As Therapeutic Vaccine, Transmission Limitation, and Immunoglobulin Enhancement. 2020. Available in https://clinicaltrials.gov/ct2/show/study/NCT04369794
89. COVID19-Convalescent Plasma for Treating Patients With Active Symptomatic COVID 19 Infection (FALP-COVID). 2020. Available in https://clinicaltrials.gov/show/NCT04384588
90. CovPall: Improving palliative care for people affected by the COVID-19 pandemic by sharing learning – the national and international response. 2020. Available in <http://isrctn.com/ISRCTN16561225>
91. Cri Analog PG1 Effectiveness and Safety in Covid-19. 2020. Available in https://clinicaltrials.gov/ct2/show/NCT04536363
92. CROWN CORONATION: Chloroquine repurposing to healthworkers for novel coronavirus mitigation. 2020. Available in http://isrctn.com/ISRCTN99916292
93. Cyclosporine A Plus Low-steroid Treatment in COVID-19 Pneumonia. 2020. Available in https://clinicaltrials.gov/ct2/show/NCT04540926
94. Daily Regimen of Tenofovir/Emtricitabine as Prevention for COVID-19 in Health Care Personnel in Colombia. 2020. Available in https://clinicaltrials.gov/ct2/show/NCT04519125
95. DALIA Study. 2020. Available in https://rpcec.sld.cu/en/trials/RPCEC00000330-En
96. Desidustat in the Management of COVID-19 Patients. 2020. Available in <https://clinicaltrials.gov/show/NCT04463602>
97. Dipeptidyl Peptidase-4 Inhibitor (DPP4i) for the Control of Hyperglycemia in Patients With COVID-19 (Covid19DPP4i). 2020. Available in https://clinicaltrials.gov/ct2/show/NCT04542213
98. Distress and resilience of healthcare professionals during the COVID-19 (coronavirus) pandemic. 2020. Available in http://isrctn.com/ISRCTN13694948
99. Early prone position in patients on spontaneous ventilation. 2020. Available in http://www.ensaiosclinicos.gov.br/rg/RBR-7d7ngw/
100. Early Treatment With Ivermectin and LosarTAN for Cancer Patients With COVID-19 Infection. 2020. Available in https://clinicaltrials.gov/show/NCT04447235
101. Early Use of Corticosteroids in Hospitalized Patients With Moderate COVID19 Pneumonia. 2020. Available in https://clinicaltrials.gov/ct2/show/NCT04451174
102. Educational intervention by cell phone to increase informed choices at childbirth, in the context of the Covid-19 pandemic.. 2020. Available in http://www.ensaiosclinicos.gov.br/rg/RBR-3g5f9f/
103. Effect of a Nss to Reduce Complications in Patients With Covid-19 and Comorbidities in Stage III. 2020. Available in https://clinicaltrials.gov/ct2/show/NCT04507867
104. Effect of a special nutritional oral supplement use on inflammation in patients with COVID-19. 2020. Available in http://www.ensaiosclinicos.gov.br/rg/RBR-7jrxqm/
105. Effect of convalescent plasma in patients with severe COVI-19. 2020. Available in http://www.ensaiosclinicos.gov.br/rg/RBR-4vm3yy/
106. Effect of COVID-19 convalescent plasma produced by HEMOPE: A randomized study, with a comparative group in several centers. 2020. Available in http://www.ensaiosclinicos.gov.br/rg/RBR-7jqpnw/
107. Effect of COVID-19 on Platelet Aggregation. 2020. Available in https://clinicaltrials.gov/show/NCT04447131
108. Effect of eating habits and exposure to light on sleep, emotion, memory and headache in college students during the COVID-19 pandemic. 2020. Available in http://www.ensaiosclinicos.gov.br/rg/RBR-5kkpg6/
109. Effect of High vs Low Doses of Chloroquine Diphosphate as Adjunctive Therapy for Patients Hospitalized With Severe Acute Respiratory Syndrome Coronavirus 2 (SARS-CoV-2) Infection: A Randomized Clinical Trial. 2020. Available in https://www.clinicaltrials.gov/ct2/show/NCT04323527
110. Effect of the Use of Anticoagulant Therapy During Hospitalization and Discharge in Patients With COVID-19 Infection. 2020. Available in https://clinicaltrials.gov/show/NCT04508439
111. Effect of two covers to prevent injuries caused to professionals working in the health area due to the use of personal protective equipment. 2020. Available in http://www.ensaiosclinicos.gov.br/rg/RBR-7drpyr/
112. Effectiveness and Safety of Convalescent Plasma in Patients With High-risk COVID-19. 2020. Available in https://clinicaltrials.gov/show/NCT04425837
113. Effectiveness and Safety of Ivermectin for the Prevention of Covid-19 Infection in Colombian Health Personnel (IveprofCovid19). 2020. Available in https://clinicaltrials.gov/ct2/show/NCT04527211
114. Effectiveness and Safety of Medical Treatment for SARS-CoV-2 (COVID-19) in Colombia. 2020. Available in https://clinicaltrials.gov/ct2/show/NCT04359095
115. Effects of digital home exercise programs on physical and mental well-being during the COVID-19 pandemic: a multi-center trial. 2020. Available in <http://www.drks.de/DRKS00021273>
116. Effects of tests for covid-19 on the preservation of the healthcare workforce during pandemia in Brazil. 2020. Available in https://ensaiosclinicos.gov.br/rg/RBR-6w5tbk/
117. Efficacy and Safety of MTX-loaded Nanoparticles to Treat Severe COVID-19 Patients. 2020. Available in https://clinicaltrials.gov/show/NCT04352465
118. Efficacy and Safety of Nitazoxanide for the Treatment of Hospitalized Patients With Moderate COVID-19. 2020. Available in https://clinicaltrials.gov/show/NCT04348409
119. Efficacy of BACMUNE (MV130) in the Prevention of Disease Due to COVID-19 Infection in Healthcare Personnel. 2020. Available in https://clinicaltrials.gov/show/NCT04452643
120. Efficacy of Chloroquine or Hydroxychloroquine in Treating Pneumonia Caused by SARS-Cov-2 - COVID-19. 2020. Available in https://clinicaltrials.gov/ct2/show/NCT04420247
121. Efficacy of L. Plantarum and P. Acidilactici in Adults With SARS-CoV-2 and COVID-19. 2020. Available in https://clinicaltrials.gov/ct2/show/NCT04517422
122. Efficacy of Vitamin D Treatment in Pediatric Patients Hospitalized by COVID-19. 2020. Available in https://clinicaltrials.gov/show/NCT04502667
123. Efficacy, Safety and Immunogenicity of Inactivated SARS-CoV-2 Vaccines (Vero Cell) in Healthy Adult Population In Peru. 2020. Available in https://clinicaltrials.gov/ct2/show/NCT04612972
124. Efficacy, Safety and Tolerability of Ivermectin in Subjects Infected With SARS-CoV-2 With or Without Symptoms. 2020. Available in https://clinicaltrials.gov/ct2/show/NCT04407507
125. Elmo Respiratory Support Project - COVID-19. 2020. Available in https://clinicaltrials.gov/ct2/show/NCT04470258
126. Estrogen Therapy in Non-severe COVID-19 Patients. 2020. Available in https://clinicaltrials.gov/ct2/show/NCT04539626
127. Evaluation of an Alternative Method of Obtaining Viral RNA for the Detection of SARS-CoV-2 Virus Using PCR. 2020. Available in https://clinicaltrials.gov/show/NCT04468217
128. Evaluation of COVID-19 in patients with Chronic Inflammatory Rheumatic Diseases.. 2020. Available in http://www.ensaiosclinicos.gov.br/rg/RBR-33ytqc/
129. Evaluation of immunity in the evolution of the severe or mild form of COVID-19. 2020. Available in http://www.ensaiosclinicos.gov.br/rg/RBR-62zdkk/
130. Evaluation of muscle weakness, function and quality of life after Intensive Care Unit discharge in survivors of COVID-19. 2020. Available in http://www.ensaiosclinicos.gov.br/rg/RBR-564y4n/
131. Evaluation of symptoms, respiratory, pulmonary image and inflammation in patients who have been hospitalized for coronavirus infection in the period of a year, multicentric study (fenix). 2020. Available in http://www.ensaiosclinicos.gov.br/rg/RBR-8j9kqy/
132. Evaluation of the Immune Response to SARS-CoV-2 Among Patients With Covid-19 in Costa Rica. 2020. Available in https://clinicaltrials.gov/ct2/show/NCT04537338
133. Evaluation of the synergistic effect of sequential administration of two immunostimulant products: Immuno-1 and Immuno-2 for the stimulation of the immune response in older adults. Exploratory study. 2020. Available in https://rpcec.sld.cu/en/trials/RPCEC00000315-En
134. Evaluation of the use of Hydroxychlorochine in Chinese flu. 2020. Available in http://www.ensaiosclinicos.gov.br/rg/RBR-3k4wxb/
135. Evaluation with ultrasound on the bedside in serious patients with COVID-19. 2020. Available in http://www.ensaiosclinicos.gov.br/rg/RBR-43hbks/
136. EXercise TRAining and Sedentary Lifestyle on Clinical Outcomes in Patients With COVID-19. 2020. Available in https://clinicaltrials.gov/show/NCT04396353
137. Exploratory study of BMT IM to stimulate immune response in patients with CKD-5 on iterated hemodialysis. 2020. Available in https://rpcec.sld.cu/en/trials/RPCEC00000316-En
138. Frailty and negative consequences in adults hospitalized for COVID-19. 2020. Available in http://www.ensaiosclinicos.gov.br/rg/RBR-7w5zhr/
139. Full Anticoagulation Versus Prophylaxis in COVID-19: COALIZAO ACTION Trial. 2020. Available in https://clinicaltrials.gov/show/NCT04394377
140. Global Assessment of Acute and Chronic Kidney Disease Incidence and Outcomes in Patients With COVID-19 Infection. 2020. Available in https://clinicaltrials.gov/show/NCT04491227
141. Glycine Supplement for Severe COVID-19. 2020. Available in https://clinicaltrials.gov/show/NCT04443673
142. Hemostasis in COVID-19: an Adaptive Clinical Trial. 2020. Available in https://clinicaltrials.gov/show/NCT04466670
143. Home-based Aerobic Training Among Adolescents With Chronic Diseases During COVID-19 Pandemic. 2020. Available in https://clinicaltrials.gov/ct2/show/NCT04458246
144. Home-based Exercise Training During Covid-19 Pandemic in Post-bariatric. 2020. Available in https://clinicaltrials.gov/show/NCT04425005
145. HOPE: a Trial of Hydroxichloroquine Plus Azithromycin in High Risk COVID-19. 2020. Available in https://clinicaltrials.gov/ct2/show/NCT04575558
146. How Brazilian Chiropractors Are Facing the COVID-19 Pandemic. 2020. Available in https://clinicaltrials.gov/ct2/show/NCT04534621
147. Hydroxychloroquine and Lopinavir/ Ritonavir to Improve the Health of People With COVID-19: "The Hope Coalition - 1". 2020. Available in https://clinicaltrials.gov/show/NCT04403100
148. Hydroxychloroquine Chemoprophylaxis in Healthcare Personnel in Contact With COVID-19 Patients (PHYDRA Trial). 2020. Available in https://clinicaltrials.gov/show/NCT04318015
149. Hydroxychloroquine Treatment for Severe COVID-19 Pulmonary Infection (HYDRA Trial). 2020. Available in https://clinicaltrials.gov/show/NCT04315896
150. Hyperbaric Oxygen as an Adjuvant Treatment for Patients With Covid-19 Severe Hypoxemia. 2020. Available in https://clinicaltrials.gov/show/NCT04477954
151. Hyperbaric Oxygen Therapy for hospitalized patients with Covid-19. 2020. Available in http://www.ensaiosclinicos.gov.br/rg/RBR-2fvjjm/
152. HYPONATREMIA IN COVID-19 PATIENTS. 2020. Available in https://clinicaltrials.gov/show/NCT04493268
153. Immune Monitoring of Prophylactic Effect of Hydroxychloroquine in Healthcare Providers Highly Exposed to COVID-19. 2020. Available in https://clinicaltrials.gov/show/NCT04346329
154. Impact in the Medical Care Among the Rheumatic Diseases Patients in a Tertiary Hospital in MÃ©xico During the COVID-19 Pandemic. 2020. Available in https://clinicaltrials.gov/ct2/show/NCT04557358
155. Impact of Colchicine in Hospitalized Colombian Patients With COVID-19 ((COLCOVID19)). 2020. Available in https://clinicaltrials.gov/ct2/show/NCT04539873
156. Impact of COVID-19 on Mental Health of Patients With Skin Picking. 2020. Available in https://clinicaltrials.gov/ct2/show/NCT04522492
157. Impact of Tele-interventions During the COVID-19 Pandemic in Patients With Diabetes Mellitus. 2020. Available in https://clinicaltrials.gov/ct2/show/NCT04344210
158. Inactivated Convalescent Plasma as a Therapeutic Alternative in Patients CoViD-19. 2020. Available in https://clinicaltrials.gov/show/NCT04385186
159. Influence Physical Activity Psychological Responses COVID-19 Pandemic. 2020. Available in https://clinicaltrials.gov/ct2/show/NCT04352517
160. International SARS-CoV-2 (COVID-19) Infection Observational Study. 2020. Available in https://clinicaltrials.gov/show/NCT04385251
161. Investigating otilimab in patients with severe coronavirus related lung disease.. 2020. Available in https://www.clinicaltrialsregister.eu/ctr-search/search?query=eudract_number:2020-001759-42
162. IVERMECTIN Aspirin Dexametasone and Enoxaparin as Treatment of Covid 19. 2020. Available in https://clinicaltrials.gov/show/NCT04425863
163. Ivermectin Effect on SARS-CoV-2 Replication in Patients With COVID-19. 2020. Available in https://clinicaltrials.gov/show/NCT04381884
164. Ivermectin in Adults With Severe COVID-19. 2020. Available in https://clinicaltrials.gov/ct2/show/NCT04602507
165. Ivermectin to Prevent Hospitalizations in COVID-19. 2020. Available in https://clinicaltrials.gov/ct2/show/NCT04529525
166. Ivermectin-Azithromycin-Cholecalciferol (IvAzCol) Combination Therapy for COVID-19. 2020. Available in https://clinicaltrials.gov/show/NCT04399746
167. Lactoferrin for Prevention of COVID-19 in Health Care Workers. 2020. Available in https://clinicaltrials.gov/ct2/show/NCT04526821
168. Less Frequency Hemodialysis and COVID-19. 2020. Available in https://clinicaltrials.gov/show/NCT04374058
169. Lifestyle modification of adolescents from different countries during confinement by Covid-19. 2020. Available in http://isrctn.com/ISRCTN14025343
170. Liver Injury in Patients With COVID-19. 2020. Available in https://clinicaltrials.gov/ct2/show/NCT04358380
171. Long-term Characterization of Patients With Severe/Critical Infection by COVID-19 Virus. 2020. Available in https://clinicaltrials.gov/ct2/show/NCT04559100
172. Long-term Impact in Intensive Care Survivors of Coronavirus disease-19 (COVID-19). 2020. Available in https://anzctr.org.au/ACTRN12620000799954.aspx
173. Losmapimod Safety and Efficacy in COVID-19. 2020. Available in https://clinicaltrials.gov/show/NCT04511819
174. Low-dose Hydroxychloroquine and Bromhexine: a Novel Regimen for COVID-19 Prophylaxis in Healthcare Professionals. 2020. Available in https://clinicaltrials.gov/ct2/show/NCT04340349
175. Lung Function, Exercise Capacity and Health-Related Quality of Life After Severe COVID-19. 2020. Available in https://clinicaltrials.gov/show/NCT04410107
176. M5049 Study in Participants With Coronavirus Disease 2019 (COVID-19) Pneumonia. 2020. Available in https://clinicaltrials.gov/show/NCT04448756
177. Mechanical Ventilation Strategy for Coronavirus Disease 2019 (COVID-19). 2020. Available in https://clinicaltrials.gov/show/NCT04497454
178. Melatonin Agonist on Hospitalized Patients With Confirmed or Suspected COVID-19. 2020. Available in https://clinicaltrials.gov/ct2/show/NCT04470297
179. Mental health affected by COVID-19. 2020. Available in http://www.ctri.nic.in/Clinicaltrials/pmaindet2.php?trialid=45764
180. Mental Health and Academic Performance in COVID-19. 2020. Available in https://clinicaltrials.gov/show/NCT04420416
181. Mental Health Impact of the COVID-19 Pandemic Among Migrants in Chile. 2020. Available in https://clinicaltrials.gov/ct2/show/NCT04497636
182. Mesenchymal Stem Cell for Acute Respiratory Distress Syndrome Due for COVID-19. 2020. Available in https://clinicaltrials.gov/show/NCT04416139
183. Methylprednisolone in the Treatment of Patients With Signs of Severe Acute Respiratory Syndrome in Covid-19. 2020. Available in https://clinicaltrials.gov/show/NCT04343729
184. Microvascular Flow and Reactivity in Patients Presenting in the Acute Phase of COVID-19.. 2020. Available in https://clinicaltrials.gov/show/NCT04406545
185. Muscle Mass and Strength as Predictors of Time to Discharge in Patients With COVID-19. 2020. Available in https://clinicaltrials.gov/show/NCT04406233
186. Needs, Perceptions and Acute Stress of Healtcare Workers Caring for COVID-19 Patients in South America. 2020. Available in https://clinicaltrials.gov/ct2/show/NCT04486404
187. NestaCell® Mesenchymal Stem Cell to Treat Patients With Severe COVID-19 Pneumonia (HOPE). 2020. Available in https://clinicaltrials.gov/ct2/show/NCT04315987
188. Northeast COVID-19 and Pregnancy Study Group. 2020. Available in https://regroup- https://clinicaltrials.gov/ct2/keydates/NCT04462367
189. Novel Coronavirus infection among health workers. 2020. Available in http://www.ensaiosclinicos.gov.br/rg/RBR-7nky44/
190. Oncological Surgery in Times of COVID-19: Effectiveness of Preoperative Screening for Sars-Cov-2. 2020. Available in https://clinicaltrials.gov/show/NCT04434261
191. One year follow-ups of Chilean patients with COVID-19. 2020. Available in 10.1186/ISRCTN16865246
192. Online meditation as an aid in reducing symptoms of Depression, Anxiety and Psychological Stress in Resident Physicians during COVID-19 pandemic in Brazil. 2020. Available in https://ensaiosclinicos.gov.br/rg/RBR-2w7ghc/
193. Online Positive Psychology Intervention for Depression and Anxiety Due to COVID-19. 2020. Available in https://clinicaltrials.gov/ct2/show/NCT04468893
194. Opaganib, a Sphingosine Kinase-2 (SK2) Inhibitor in COVID-19 Pneumonia. 2020. Available in https://clinicaltrials.gov/ct2/show/NCT04467840
195. Outcomes Of Cancer Surgery During COVID-19 Pandemic. 2020. Available in http://www.ctri.nic.in/Clinicaltrials/pmaindet2.php?trialid=43628
196. Outcomes of Patients With COVID-19 in the Intensive Care Unit. 2020. Available in https://clinicaltrials.gov/show/NCT04336345
197. Outpatient Treatment With CoVid-19 With Prexablu. 2020. Available in https://clinicaltrials.gov/ct2/show/NCT04619290
198. P2Et Extract in the Symptomatic Treatment of Subjects With COVID-19. 2020. Available in https://clinicaltrials.gov/ct2/show/NCT04410510
199. Pegylated Interferon - a2b With SARSCoV- 2 (COVID-19). 2020. Available in https://clinicaltrials.gov/show/NCT04480138
200. Performance Evaluation of BCG vs COVID-19. 2020. Available in https://clinicaltrials.gov/ct2/show/NCT04362124
201. PERUCONPLASMA: Evaluating the Use of Convalescent Plasma as Management of Covid-19. 2020. Available in https://clinicaltrials.gov/ct2/show/NCT04497324
202. Pharmacodynamics of the Nasalferon in healthy volunteers.. 2020. Available in https://rpcec.sld.cu/en/trials/RPCEC00000308-En
203. Phase 2/3 study of ABX464, once daily oral capsule, in high risk patients infected by SARS-CoV-2 prior to respiratory distress.. 2020. Available in https://www.clinicaltrialsregister.eu/ctr-search/search?query=eudract_number:2020-001673-75
204. Phase 3 Randomized, Double-blind, Placebo-controlled Multi-center Study to Assess the Efficacy and Safety of Ruxolitinib in Patients With COVID-19 Associated Cytokine Storm (RUXCOVID). 2020. Available in https://clinicaltrials.gov/show/NCT04362137
205. PHASE 3 RANDOMIZED, DOUBLE-BLIND, PLACEBO-CONTROLLED, MULTI-CENTER STUDY TO ASSESS THE EFFICACY AND SAFETY OF RUXOLITINIB IN PATIENTS WITH COVID-19 ASSOCIATED CYTOKINE STORM (RUXCOVID). 2020. Available in https://www.ins.gob.pe/ensayosclinicos/rpec/recuperarECPBNuevoEN.asp?numec=030-20
206. Phase I / II Clinical Study of Immunotherapy Based on Adoptive Cell Transfer as a Therapeutic Alternative for Patients With COVID-19 in Colombia. 2020. Available in https://clinicaltrials.gov/ct2/show/NCT04344548
207. Photobiomodulation Therapy Combined With Static Magnetic Field in Patients With COVID-19. 2020. Available in https://clinicaltrials.gov/show/NCT04386694
208. Plasma Therapy of COVID-19 in Severely Ill Patients. 2020. Available in https://clinicaltrials.gov/ct2/show/NCT04359810
209. Postural Recruitment Maneuver in Patients With Acute Respiratory Distress Syndrome Due to COVID-19 Infection. 2020. Available in https://clinicaltrials.gov/show/NCT04475068
210. Prevalence of COVID-19 (Coronavirus Disease 19) in Pregnant Women on Labor in a Public Hospital in Chile (COroNavirus diSease Covid-19 pandEmic iN ChilE). 2020. Available in https://clinicaltrials.gov/ct2/show/NCT04551690
211. Prevalence of COVID-19 in High Altitude : Insights From the Highest City of the World. 2020. Available in https://clinicaltrials.gov/ct2/show/NCT04604249
212. PrevengHo-Vir Homeoprohylaxis COVID-2019. 2020. Available in https://rpcec.sld.cu/en/trials/RPCEC00000312-En
213. Prevention of Respiratory Complications In At Surgery in COVID-19 Pandemic. 2020. Available in http://www.ctri.nic.in/Clinicaltrials/pmaindet2.php?trialid=43482
214. Prevention of Severe Covid-19 in Infected Elderly by Early Administration of Convalescent Plasma With High-titers of Antibody Against SARS-CoV2. 2020. Available in https://clinicaltrials.gov/ct2/show/NCT04479163
215. Prevention, Efficacy and Safety of BCG Vaccine in COVID-19 Among Healthcare Workers. 2020. Available in https://clinicaltrials.gov/ct2/show/NCT04461379
216. Profile of hospitalized patients with Covid-19 and repercussions of physiotherapy. 2020. Available in http://www.ensaiosclinicos.gov.br/rg/RBR-2f9k8p/
217. Prone Positioning in Non-intubated Patients With COVID-19 Associated Acute Respiratory Failure. 2020. Available in https://clinicaltrials.gov/show/NCT04477655
218. Prospective Brazilian Registry for Clinical Presentation of Individuals With COVID-19 (SARS-Brazil). 2020. Available in https://clinicaltrials.gov/ct2/show/NCT04479488
219. Prospective Determination of COVID-19 Infection Rate in a Chemotherapy Unit in Mexico. 2020. Available in https://clinicaltrials.gov/ct2/show/NCT04567979
220. Protection of Health Workers Against COVID-19. 2020. Available in https://clinicaltrials.gov/ct2/show/NCT04446065
221. Psychological effects of social isolation in the coronavirus pandemic. 2020. Available in http://www.ensaiosclinicos.gov.br/rg/RBR-9pgwfc/
222. Public health emergency SOLIDARITY trial of treatments for COVID-19 infection in hospitalized patients. 2020. Available in http://isrctn.com/ISRCTN83971151
223. Pulmonary rehabilitation in individuals affected by Covid-19. 2020. Available in http://www.ensaiosclinicos.gov.br/rg/RBR-4wnx2q/
224. Pyridostigmine in Severe SARS-CoV-2 Infection. 2020. Available in https://clinicaltrials.gov/ct2/show/NCT04343963
225. Quality of Life and Patient-centered Outcomes After Hospitalization for COVID-19. 2020. Available in https://clinicaltrials.gov/ct2/show/NCT04376658
226. RANDOMIZED PHASE IIA CLINICAL TRIAL TO COMPARE THE EFFICACY OF IVERMECTIN VERSUS PLACEBO TO OBTAIN NEGATIVE PCR RESULTS IN PATIENTS WITH EARLY PHASE COVID-19. 2020. Available in https://www.ins.gob.pe/ensayosclinicos/rpec/recuperarECPBNuevoEN.asp?numec=034-20
227. Randomized Placebo-controlled Trial of Hydroxychloroquine in Outpatient Cases With Coronavirus Disease 2019 (COVID-19). 2020. Available in https://clinicaltrials.gov/show/NCT04466540
228. Randomized, study to evaluate early prone position in patients with COVID-19. 2020. Available in http://www.ensaiosclinicos.gov.br/rg/RBR-2k66ft/
229. Randomized, pragmatic, double-blind, placebo-controlled clinical study, evaluation of Hydroxychloroquine for prevention of hospitalizations and respiratory complications in outpatients with confirmed or probable diagnosis of COVID-19. 2020. Available in https://ensaiosclinicos.gov.br/rg/RBR-3cbs3w/
230. Rectal Ozone in the COVID-19 treatment. 2020. Available in https://rpcec.sld.cu/en/trials/RPCEC00000320-En
231. Ruxolitinib for Acute Respiratory Disorder Syndrome Due to COVID-19. 2020. Available in https://www.clinicaltrials.gov/ct2/show/NCT04477993
232. Safety and Antiviral Activity of BLD-2660 in COVID-19 Hospitalized Subjects. 2020. Available in https://clinicaltrials.gov/show/NCT04334460
233. Safety and Efficacy of Anti-SARS-CoV-2 Equine Antibody Fragments (INOSARS) for Hospitalized Patients With COVID-19. 2020. Available in https://clinicaltrials.gov/show/NCT04514302
234. Safety and Efficacy of Intravenous Wharton's Jelly Derived Mesenchymal Stem Cells in Acute Respiratory Distress Syndrome Due to COVID 19. 2020. Available in https://clinicaltrials.gov/show/NCT04390152
235. Safety and Efficacy of Maraviroc and/or Favipiravir vs Currently Used Therapy in Severe COVID-19 Adults. 2020. Available in https://clinicaltrials.gov/ct2/show/NCT04475991
236. Safety and Efficacy of Viusid and Asbrip in Hospitalized Patients Infected by SARS-Cov-2 With COVID-19. 2020. Available in https://clinicaltrials.gov/ct2/show/NCT04407182
237. Sarilumab COVID-19. 2020. Available in https://clinicaltrials.gov/show/NCT04327388
238. SARS-COV-2 Infection in Kidney Transplant Recipients: a Brazilian Multicenter Study. 2020. Available in https://clinicaltrials.gov/ct2/show/NCT04494776
239. Seroepidemiological Study of SARS-CoV-2 (COVID-19) Infection in Population Subgroups in the State of SÃ£o Paulo. 2020. Available in https://clinicaltrials.gov/ct2/show/NCT04408014
240. SERO-EPIDEMIOLOGY OF SARS-COV2 INFECTION IN THE AUTONOMOUS CITY OF BUENOS AIRES. 2020. Available in https://clinicaltrials.gov/ct2/show/NCT04482361
241. Serologic Surveillance for SARS-CoV-2 (COVID-19) in a Prospective Cohort of Health Care. 2020. Available in https://clinicaltrials.gov/ct2/show/NCT04387890
242. Seroprevalence of Coronavirus Disease 2019 (COVID-19) Antibodies in a Vulnerable Neighbourhood, Buenos Aries Argentina. 2020. Available in https://clinicaltrials.gov/ct2/show/NCT04472078
243. Sildenafil in COVID-19. 2020. Available in https://clinicaltrials.gov/show/NCT04489446
244. SOBERANA 01. 2020. Available in https://rpcec.sld.cu/en/trials/RPCEC00000332-En
245. Software to evaluate clinical, epidemiological data and chest computed tomography to predict which patients with COVID-19 will develop a severe form of the disease.. 2020. Available in http://www.ensaiosclinicos.gov.br/rg/RBR-7dsxsv/
246. SOLIDARITY: AN INTERNATIONAL RANDOMIZED CONTROLLED TRIAL TO EVALUATE NON-LICENSED COVID-19 TREATMENTS IN ADDITION TO STANDARD OF CARE AMONG HOSPITALIZED PATIENTS. 2020. Available in https://www.ins.gob.pe/ensayosclinicos/rpec/recuperarECPBNuevoEN.asp?numec=010-20
247. Statistical and Epidemiological Study Based on the Use of Convalescent Plasma for the Management of Patients With COVID-19. 2020. Available in https://clinicaltrials.gov/show/NCT04452812
248. Stem cells in patients with lung lesions (COVID-19). 2020. Available in https://rpcec.sld.cu/en/trials/RPCEC00000322-En
249. Stress Echocardiography in Patients Recovery From Mild COVID-19 Illness. 2020. Available in https://clinicaltrials.gov/show/NCT04498299
250. Study in Hospitalized COVID-19 patients with Acalabrutinib along with the Best Supportive Care versus Best Supportive Care. 2020. Available in http://www.ctri.nic.in/Clinicaltrials/pmaindet2.php?trialid=44554
251. Study of the Safety and Efficacy of STI-5656 (Abivertinib Maleate) in Subjects Hospitalized Due to COVID-19. 2020. Available in https://clinicaltrials.gov/ct2/show/NCT04528667
252. Study of vascular complications in critically ill patients with coronavirus. 2020. Available in http://www.ensaiosclinicos.gov.br/rg/RBR-4qjzh7/
253. Study to Assess Efficacy and Safety Relative to Standard of Care in Patients With COVID-19 Pneumonia. 2020. Available in https://clinicaltrials.gov/show/NCT04459676
254. Study to Describe the Safety, Tolerability, Immunogenicity, and Efficacy of RNA Vaccine Candidates Against COVID-19 in Healthy Adults. 2020. Available in https://clinicaltrials.gov/show/NCT04368728
255. Sulodexide in the treatment of COVID-19. 2020. Available in http://isrctn.com/ISRCTN59048638
256. Suloexide in the Treatment of Early Stages of COVID-19. 2020. Available in https://clinicaltrials.gov/ct2/show/NCT04483830
257. Surgery Outcomes in COVID patients. 2020. Available in http://www.ctri.nic.in/Clinicaltrials/pmaindet2.php?trialid=43610
258. Switch of Renin-Angiotensin System Inhibitors in Patients With Covid-19. 2020. Available in https://clinicaltrials.gov/show/NCT04493359
259. TAF/FTC for Pre-exposure Prophylaxis of COVID-19 in Healthcare Workers (CoviPrep Study). 2020. Available in https://clinicaltrials.gov/ct2/show/NCT04405271
260. Tannin Specific Natural Extract for COVID-19 Infection. 2020. Available in https://clinicaltrials.gov/ct2/show/NCT04403646
261. Telemedicine in the Covid-19 pandemic in Brazil in a medical clinic in the supplementary health sector in SÃ£o Paulo, Brazil. 2020. Available in https://ensaiosclinicos.gov.br/rg/RBR-658khm/
262. Telmisartan for Treatment of COVID-19 Patients. 2020. Available in https://clinicaltrials.gov/ct2/show/NCT04355936
263. Telmisartan in Respiratory Failure Due to COVID-19. 2020. Available in https://clinicaltrials.gov/show/NCT04510662
264. Testing of BCG vaccine against COVID-19. 2020. Available in http://www.ensaiosclinicos.gov.br/rg/RBR-4kjqtg/
265. The Containing Coronavirus Disease 19 (COVID-19) Trial. 2020. Available in https://clinicaltrials.gov/ct2/show/NCT04552379
266. The Covid-19 HEalth caRe wOrkErS (HEROES) Study. 2020. Available in https://clinicaltrials.gov/ct2/show/NCT04352634
267. The ECLA PHRI COLCOVID Trial. Effects of Colchicine on Moderate/High-risk Hospitalized COVID-19 Patients.. 2020. Available in https://clinicaltrials.gov/show/NCT04328480
268. The impact of the COVID-19 pandemic on the provision, practice, and outcomes of vascular surgery (COVER study). 2020. Available in http://isrctn.com/ISRCTN80453162
269. The role of early dialysis in the evolution of patients with COVID19. 2020. Available in http://www.ensaiosclinicos.gov.br/rg/RBR-3rdhgm/
270. The Use of Brazilian Green Propolis Extract (EPP-AF) in Patients Affected by COVID-19. 2020. Available in https://clinicaltrials.gov/ct2/show/NCT04480593
271. Therapeutic Plasmapheresis in Critically Ill Adult Patients With COVID-19 Confirmed Diagnosis. 2020. Available in https://clinicaltrials.gov/ct2/show/NCT04480632
272. Therapy for COVID-19-Phase I-II. 2020. Available in https://rpcec.sld.cu/en/trials/RPCEC00000309-En
273. Tomographic Findings in COVID-19 and Influenza. 2020. Available in https://clinicaltrials.gov/show/NCT04497311
274. Treatment of Critically Ill Patients With Covid-19 With Convalescent Plasma. 2020. Available in https://clinicaltrials.gov/show/NCT04468009
275. Treatment of Patients With COVID-19 With Convalescent Plasma. 2020. Available in https://clinicaltrials.gov/ct2/show/NCT04415086
276. Treatment of severe COVID-19 with angiotensin-(1-7). 2020. Available in http://www.ensaiosclinicos.gov.br/rg/RBR-35734p/
277. Use of Anti-IL-17 in patients with Covid-19 associated pneumonia. 2020. Available in https://ensaiosclinicos.gov.br/rg/RBR-5vpyh4/
278. Use of Hydroxychloroquine Alone or Associated for Inpatients With SARS-CoV2 Virus (COVID-19). 2020. Available in https://clinicaltrials.gov/show/NCT04361461
279. Use of the Nasal Cannula During COVID-19. 2020. Available in https://clinicaltrials.gov/show/NCT04376580
280. USEFULNESS of Topic Ivermectin and Carrageenan to Prevent Contagion of Covid 19. 2020. Available in https://clinicaltrials.gov/show/NCT04425850
281. Using blood plasma to develop passive immunity to coronavirus in Ecuador. 2020. Available in https://www.isrctn.com/ISRCTN85216856
282. Using Tocilizumab in critically ill patients with COVID-19.. 2020. Available in http://www.ensaiosclinicos.gov.br/rg/RBR-3zdynp/
283. Validation of Laboratory Techniques, Strategies, and Types of Samples for Epidemiological Control in the Covid-19 Pandemic. 2020. Available in https://clinicaltrials.gov/ct2/show/NCT04581083
284. VA-MENGOC-BC to stimulate the innate response against SARS-CoV-2. 2020. Available in https://rpcec.sld.cu/en/trials/RPCEC00000314-En
285. Video Dance Class and Unsupervised Physical Activity During Covid-19 Pandemic in People With Parkinson's Disease. 2020. Available in https://clinicaltrials.gov/show/NCT04422353
286. Vitamin D Supplementation in Patients With COVID-19. 2020. Available in https://clinicaltrials.gov/show/NCT04449718
287. WORLD HEART FEDERATION (WHF) COVID-19 and Cardiovascular Disease Survey. 2020. Available in https://clinicaltrials.gov/show/NCT04475471

# Excluded studies

## Articles excluded with their reason

| **Reference** | **Title** | **Exclusion reason** |
| --- | --- | --- |
| A 2020 | Diffusion processes in multilayer transportation networks: the flight of the Coronavirus | Not focused in LAC countries |
| Ã‘amendys-Silva 2020 | Health care workers with COVID-19 in Mexico | Not empirical primary research |
| AbrahÃ£odeCastro 2020 | Social protection in times of Covid-19 | Not empirical primary research |
| Abrantes 2020 | Oral cancer diagnosis during the COVID-19 pandemic in an oral pathology laboratory in Rio de Janeiro, Brazil | Not empirical primary research |
| Abu-Rayash 2020 | Analysis of mobility trends during the COVID-19 coronavirus pandemic: Exploring the impacts on global aviation and travel in selected cities | Not focused in LAC countries |
| Accinelli 2020 | At High Altitude COVID-19 Is Less Frequent: The Experience of Peru | Not empirical primary research |
| Adam 2020 | Networks in a World Unknown: Public WhatsApp Groups in the Venezuelan Refugee Crisis | Not produced in response to the COVID-19 pandemic |
| Aguilar-Guerra 2020 | Mobilizing Primary Health Care:Cuba's Powerful Weapon against COVID-19. | Not empirical primary research |
| Aisa-Alvarez 2020 | A Randomized clinical trial of antioxidant therapy in patients with septic shock. Reference study to propose adjuvant therapy in patients with critical organic damage by COVID-19 | Not produced in response to the COVID-19 pandemic |
| Albuquerque 2020 | Overview of confirmed cases of COVID-19 in five countries facing community transmission | Not focused in LAC countries |
| Albuquerque 2020 | Planejamento operacional durante a pandemia de Covid-19: comparaÃ§Ã£o entre recomendaÃ§Ãµes da OrganizaÃ§Ã£o Mundial de SaÃºde e o Plano de de ContigÃªncia Nacional | Not empirical primary research |
| AlcidesEduardodosReis 2020 | Viral Surveillance: Governing Social Isolation in SÃ£o Paulo, Brazil, During the COVID-19 Pandemic | Not empirical primary research |
| Alexandre 2020 | A COVID-19 na Baixada Fluminense: Colapso e apreensÃ£o a partir da periferia metropolitana do Rio de Janeiro | Not focused in LAC countries |
| Almeida-Espinosa 2020 | COVID-19: Implications of SARS-CoV-2 in Colombia | Not empirical primary research |
| Alonso 2020 | Recurrence of SARS-CoV-2 infection with a more severe case after mild COVID-19, reversion of RT-qPCR for positive and late antibody response: case report | Not empirical primary research |
| Alvarado-Moreno 2020 | COVID-19 and Dysfunctional Endothelium: The Mexican Scenario. | Not empirical primary research |
| Andrew 2020 | Rhythmic components of COVID-19 daily cases in various countries | Not focused in LAC countries |
| Angus 2020 | The Randomized Embedded Multifactorial Adaptive Platform for Community-acquired Pneumonia (REMAP-CAP) Study: rationale and Design | Not focused in LAC countries |
| Ankarali 2020 | Modeling and short-term forecasts of indicators for COVID-19 outbreak in 25 countries at the end of march | Not focused in LAC countries |
| Anonymous 2020 | COVID-19 in Argentina, what is the current epidemiological situation? | Not empirical primary research |
| AntÃºnez-Montes 2020 | Feasibility of Lung Point-of-Care Ultrasound for Patients With COVID-19 in Air Medical Transport: Triage of 2 Initially Suspected Cases on Mexico's Front Line | Not empirical primary research |
| Aquino 2020 | Social distancing measures to control the COVID-19 pandemic: potential impacts and challenges in Brazil. | Not empirical primary research |
| Araujo 2020 | Factors associated with Severe Acute Respiratory Syndrome in a Brazilian central region | Not produced in response to the COVID-19 pandemic |
| Araujo 2020 | In defense of the Unified Health System in the context of SARS-CoV-2 pandemic | Not empirical primary research |
| Araujo 2020 | Nursing management in an accredited public general hospital in the response to the COVID-19 pandemic | Not empirical primary research |
| Araujo-Dos-Santos 2020 | Nursing Comiittee to Coping with COVID-19 in Bahia | Not empirical primary research |
| Armandoda 2020 | Regression Polynomial Analysis of the COVID-19 Epidemics: Some Initial Findings | Not focused in LAC countries |
| Artaza 2020 | COVID-19: Lessons for Chile | Not empirical primary research |
| Atique 2020 | A nursing informatics response to COVID-19: perspectives from five regions of the world. | Not empirical primary research |
| Atkinson 2020 | SARS-CoV-2 shedding and infectivity | Not empirical primary research |
| Avancini 2020 | Absence of specific cutaneous manifestations of SARS-Cov-2 in a reference center in Brazil | Not empirical primary research |
| Avaneesh 2020 | SEIHCRD Model for COVID-19 spread scenarios, disease predictions and estimates the basic reproduction number, case fatality rate, hospital, and ICU beds requirement | Not focused in LAC countries |
| Aveiro-Robalo 2020 | [Learning at home during the COVID-19 pandemic: An initiative of Latin American students]. | Not empirical primary research |
| Avila-Castro 2020 | Proposal for the management of COVID-19-associated coagulopathy in children | Not focused in LAC countries |
| Ayala 2020 | Pregnant woman with moderate COVID 19 and care process in the newborn | Not empirical primary research |
| Azarpazhooh 2020 | COVID-19 pandemic and burden of non-communicable diseases: An ecological study on data of 185 countries | Not focused in LAC countries |
| Balilla 2020 | Characterizing the Impact of COVID-19 on the Global Stock Indices through Comparative Analysis | Not focused in LAC countries |
| Baptista 2020 | Contributions of residents from multiple specializations in managing the COVID-19 pandemic in the largest public hospital Brazil | Not empirical primary research |
| Barajas-Ochoa 2020 | Retos para la educacion medica en Mexico en los tiempos del COVID-19, Challenges for medical education in Mexico in the time of COVID-19 | Not empirical primary research |
| Barberia 2020 | Political and institutional perils of Brazil's COVID-19 crisis | Not empirical primary research |
| Barbero 2020 | SARS-CoV-2 in pregnancy: characteristics and outcomes of hospitalized and non-hospitalized women due to COVID-19 | Not focused in LAC countries |
| Barrios 2020 | Volver a empezar: cirugÃ­a electiva durante la pandemia del SARS-CoV2. Recomendaciones desde la AsociaciÃ³n Colombiana de CirugÃ­a | Not empirical primary research |
| Barros 2020 | Brazilian Nursing Process Research Network contributions for assistance in the COVID-19 pandemic | Not empirical primary research |
| BarrosdeSouza 2020 | ESTRATÃ‰GIA MUSICAL PARA CUIDAR DE DISCENTES DE ENFERMAGEM: EXPERIÃŠNCIA NO ENFRENTAMENTO DA COVID-19 | Not empirical primary research |
| Bautista-Molano 2020 | Exploring the Impact of COVID-19 in Latin America | Not empirical primary research |
| Behrens 2020 | COVID-19: IFSO LAC Recommendations for the Resumption of Elective Bariatric Surgery | Not empirical primary research |
| Belarmino 2020 | Collaborative practices from health care teams to face the covid-19 pandemic | Not empirical primary research |
| BelizÃ¡n 2020 | [Reflections on translational health research and the COVID-19 case in Argentina] | Not empirical primary research |
| Ben-HurFrancisco 2020 | Universal scaling law for COVID-19 propagation in urban centers | Not focused in LAC countries |
| Betonico 2020 | Challenges in COVID-19 medical response: A nephrology perspective | Not empirical primary research |
| BispoJunior 2020 | Community participation in the fight against COVID-19: between utilitarianism and social justice | Not empirical primary research |
| Bitencourt 2020 | Nurse's protagonism in structuring and managing a specific unit for covid-19 | Not empirical primary research |
| Blanc 2020 | What the world could learn from the Haitian resilience while managing COVID-19 | Not empirical primary research |
| Blukacz 2020 | COVID-19: leaving no one behind in Latin America | Not empirical primary research |
| Boccalatte 2020 | Recomendaciones para prevenir la infecciÃ³n por COVID-19 en cirujanos de cabeza y cuello: el comienzo de la pandemia en Argentina | Not empirical primary research |
| BomfimRibeiro 2020 | The Brazilian ongoing intervention studies for COVID-19: Clinical trials protocols | Not focused in LAC countries |
| Boni 2020 | Websurveys nos tempos de COVID-19, Web surveys in the time of COVID-19 | Not empirical primary research |
| Bonifaz 2020 | Of viruses, coronaviruses and imagination | Not empirical primary research |
| Bonilla-Aldana 2020 | Importance of the One Health approach to study the SARS-CoV-2 in Latin America | Not empirical primary research |
| Bonilla-Aldana 2020 | Una nueva zoonosis viral de preocupaciÃ³n global: COVID-19, enfermedad por coronavirus 2019 | Not empirical primary research |
| Borges 2020 | Military Nursing in "Operation Return to Brazil": aeromedical evacuation in the coronavirus pandemic | Not empirical primary research |
| Branco 2020 | Sus hospital emergency service: service flows for suspected or confirmed patients for COVID-19 | Not empirical primary research |
| BrandÃ£o 2020 | Clinical simulation strategies for knowledge integration relating to initial critical recognition and management of COVID-19 for use within continuing education and health-related academia in Brazil: a descriptive study | Not empirical primary research |
| Brasil 2020 | AtenÃ§Ã£o Ã  saÃºde do recÃ©m-nascido no contexto da infecÃ§Ã£o do novo CoronavÃ­rus (SARS-CoV-2) | Not empirical primary research |
| Brasil 2020 | AtencÃŒÂ§aÃŒÂƒo aÃŒÂ€ sauÃŒÂde do receÃŒÂm-nascido no contexto da infeccÃŒÂ§aÃŒÂƒo pelo novo coronaviÃŒÂrus (SARS-CoV-2) | Not empirical primary research |
| Brasil 2020 | Cabine de proteÃ§Ã£o para o tratamento de pacientes com COVID-19 | Not focused in LAC countries |
| Brasil 2020 | Como registrar no e-SUS APS quando for realizado uma teleconsulta | Not empirical primary research |
| Brasil 2020 | Condutas para a doaÃ§Ã£o de leite materno aos bancos de leite humano e postos de coleta de leite humano no contexto da infecÃ§Ã£o Covid 19 causada pelo novo CoronavÃ­rus (SARS-CoV-2) | Not empirical primary research |
| Brasil 2020 | Condutas para a doaÃ§Ã£o de leite materno aos bancos de leite humano e postos de coleta de leite humano no contexto da infecÃ§Ã£o pelo CoronavÃ­rus (SARS-CoV-2) | Not empirical primary research |
| Brasil 2020 | CoronavÃ­rus CoViD-19: Atendimento odontolÃ³gico no SUS | Not empirical primary research |
| Brasil 2020 | CoronavÃ­rus Covid-19: Fast-track de teleatendimento para a atenÃ§Ã£o primÃ¡ria: fluxo rÃ¡pido - versÃ£o 6 | Not empirical primary research |
| Brasil 2020 | CoronavÃ­rus Covid-19: Fast-track de teleatendimento para a atenÃ§Ã£o primÃ¡ria: fluxo rÃ¡pido - versÃ£o 8 | Not empirical primary research |
| Brasil 2020 | CoronavÃ­rus Covid-19: Fast-track de teleatendimento para a atenÃ§Ã£o primÃ¡ria: fluxo rÃ¡pido - versÃ£o 9 | Not empirical primary research |
| Brasil 2020 | CoronavÃ­rus CoViD-19: Fast-Track para a atenÃ§Ã£o primÃ¡ria em locais com transmissÃ£o comunitÃ¡ria fluxo rÃ¡pido - versÃ£o 8 | Not empirical primary research |
| Brasil 2020 | CoronavÃ­rus CoViD-19: Fast-Track para a atenÃ§Ã£o primÃ¡ria em locais com transmissÃ£o comunitÃ¡ria fluxo rÃ¡pido - versÃ£o 9 | Not empirical primary research |
| Brasil 2020 | CoronavÃ­rus CoViD-19: Fast-track para a atenÃ§Ã£o primÃ¡ria em locais com transmissÃ£o comunitÃ¡ria fluxo rÃ¡pido: versÃ£o 6 | Not empirical primary research |
| Brasil 2020 | CoronavÃ­rus CoViD-19: Fluxo de manejo clÃ­nico de gestantes na atenÃ§Ã£o especializada | Not empirical primary research |
| Brasil 2020 | CoronavÃ­rus Covid-19: Fluxo de manejo clÃ­nico na atenÃ§Ã£o primÃ¡ria Ã  saÃºde em transmissÃ£o comunitÃ¡ria: versÃ£o 9 | Not empirical primary research |
[truncated: 300,711 more chars]
