# Supplementary material for: Characterising COVID-19 empirical research production in Latin America and the Caribbean: A scoping review
Source: PLoS One. 2022 Feb 16;17(2):e0263981. doi: 10.1371/journal.pone.0263981 (PMC8849471; doi:10.1371/journal.pone.0263981)
Supplement: Attachment — Submitted filename: Responses to the editor and peer review. PLOS ONE 2.0.docx [file pone.0263981.s004.docx]

PONE-D-21-25411

**Characterising COVID-19 empirical research production in Latin America and the Caribbean: a scoping review**

PLOS ONE

By Mansilla, Herrera, Boeira, et al.

*Response to Editors’ Report*

We thank the editor and reviewers for their thoughtful and productive critiques of our manuscript. All concerns and comments have been addressed in the revised manuscript, which we think is now much clearer and stronger. We respond to each of the editor and reviewer comments below, indicating where changes to the text and tables/figures can be found.

**Journal requirements:**

When submitting your revision, we need you to address these additional requirements.

1. Please ensure that your manuscript meets PLOS ONE's style requirements, including those for file naming. The PLOS ONE style templates can be found at

<https://journals.plos.org/plosone/s/file?id=wjVg/PLOSOne_formatting_sample_main_body.pdf> and

<https://journals.plos.org/plosone/s/file?id=ba62/PLOSOne_formatting_sample_title_authors_affiliations.pdf>.

Many thanks for this reminder. We have now adjusted our manuscript according to these guidelines.

2. Thank you for stating the following in your Competing Interests section:

The authors have declared that no competing interests exist. Authors hold sole responsibility for the views expressed in the manuscript, which may not necessarily reflect the opinion or policy of the Pan American Health Organization nor the World Bank Group.

Please complete your Competing Interests on the online submission form to state any Competing Interests. If you have no competing interests, please state ""The authors have declared that no competing interests exist."", as detailed online in our guide for authors at <http://journals.plos.org/plosone/s/submit-now>

This information should be included in your cover letter; we will change the online submission form on your behalf.

Many thanks for this guidance on competing interest. We have included this information in a new version of the cover letter as well.

3. We note that Figures 3a and 3b in your submission contain [map/satellite] images which may be copyrighted. All PLOS content is published under the Creative Commons Attribution License (CC BY 4.0), which means that the manuscript, images, and Supporting Information files will be freely available online, and any third party is permitted to access, download, copy, distribute, and use these materials in any way, even commercially, with proper attribution. For these reasons, we cannot publish previously copyrighted maps or satellite images created using proprietary data, such as Google software (Google Maps, Street View, and Earth). For more information, see our copyright guidelines: <http://journals.plos.org/plosone/s/licenses-and-copyright>.

We require you to either (1) present written permission from the copyright holder to publish these figures specifically under the CC BY 4.0 license, or (2) remove the figures from your submission:

a. You may seek permission from the original copyright holder of Figures 3a and 3b to publish the content specifically under the CC BY 4.0 license.

We recommend that you contact the original copyright holder with the Content Permission Form (<http://journals.plos.org/plosone/s/file?id=7c09/content-permission-form.pdf>) and the following text:

“I request permission for the open-access journal PLOS ONE to publish XXX under the Creative Commons Attribution License (CCAL) CC BY 4.0 (<http://creativecommons.org/licenses/by/4.0/>). Please be aware that this license allows unrestricted use and distribution, even commercially, by third parties. Please reply and provide explicit written permission to publish XXX under a CC BY license and complete the attached form.”

Please upload the completed Content Permission Form or other proof of granted permissions as an ""Other"" file with your submission.

In the figure caption of the copyrighted figure, please include the following text: “Reprinted from [ref] under a CC BY license, with permission from [name of publisher], original copyright [original copyright year]

b. If you are unable to obtain permission from the original copyright holder to publish these figures under the CC BY 4.0 license or if the copyright holder’s requirements are incompatible with the CC BY 4.0 license, please either i) remove the figure or ii) supply a replacement figure that complies with the CC BY 4.0 license. Please check copyright information on all replacement figures and update the figure caption with source information. If applicable, please specify in the figure caption text when a figure is similar but not identical to the original image and is therefore for illustrative purposes only.

The following resources for replacing copyrighted map figures may be helpful:

USGS National Map Viewer (public domain): <http://viewer.nationalmap.gov/viewer/>

The Gateway to Astronaut Photography of Earth (public domain): <http://eol.jsc.nasa.gov/sseop/clickmap/>

Maps at the CIA (public domain): <https://www.cia.gov/library/publications/the-world-factbook/index.html> and <https://www.cia.gov/library/publications/cia-maps-publications/index.html>

NASA Earth Observatory (public domain): <http://earthobservatory.nasa.gov/>

Landsat: <http://landsat.visibleearth.nasa.gov/>

USGS EROS (Earth Resources Observatory and Science (EROS) Center) (public domain): [http://eros.usgs.gov/#](http://eros.usgs.gov/)

Natural Earth (public domain): <http://www.naturalearthdata.com/>

We wrote to Tableau® asking for permission to use their images but didn’t receive an answer. Therefore, we decided to delete the images from the manuscript and replace them with a Table showing the data for each country. This Table was previously in the Supporting information file, so we just moved it to the main manuscript. We’ve also kept the link to the images in Tableau as a footnote of the Table.

4. We note that this manuscript is a systematic review or meta-analysis; our author guidelines therefore require that you use PRISMA guidance to help improve reporting quality of this type of study. Please upload copies of the completed PRISMA checklist as Supporting Information with a file name “PRISMA checklist”.

We have included the PRISMA checklist as a separate file named “PRISMA-ScR-Fillable-Checklist final.docx".

Please review your reference list to ensure that it is complete and correct. If you have cited papers that have been retracted, please include the rationale for doing so in the manuscript text, or remove these references and replace them with relevant current references. Any changes to the reference list should be mentioned in the rebuttal letter that accompanies your revised manuscript. If you need to cite a retracted article, indicate the article’s retracted status in the References list and also include a citation and full reference for the retraction notice.

Thank you for this reminder. We checked our reference list, and we do not have retracted papers.

Reviewers' comments:

Reviewer's Responses to Questions

Many thanks to the reviewers for responding to these questions. We will provide responses to the specific comments they made after these questions.

**Comments to the Author**

1. Is the manuscript technically sound, and do the data support the conclusions?

The manuscript must describe a technically sound piece of scientific research with data that supports the conclusions. Experiments must have been conducted rigorously, with appropriate controls, replication, and sample sizes. The conclusions must be drawn appropriately based on the data presented.

Reviewer #1: Yes

Reviewer #2: Partly

2. Has the statistical analysis been performed appropriately and rigorously?

Reviewer #1: Yes

Reviewer #2: Yes

3. Have the authors made all data underlying the findings in their manuscript fully available?

The [PLOS Data policy](http://www.plosone.org/static/policies.action#sharing) requires authors to make all data underlying the findings described in their manuscript fully available without restriction, with rare exception (please refer to the Data Availability Statement in the manuscript PDF file). The data should be provided as part of the manuscript or its supporting information, or deposited to a public repository. For example, in addition to summary statistics, the data points behind means, medians and variance measures should be available. If there are restrictions on publicly sharing data—e.g. participant privacy or use of data from a third party—those must be specified.

Reviewer #1: No

Reviewer #2: Yes

4. Is the manuscript presented in an intelligible fashion and written in standard English?

PLOS ONE does not copyedit accepted manuscripts, so the language in submitted articles must be clear, correct, and unambiguous. Any typographical or grammatical errors should be corrected at revision, so please note any specific errors here.

Reviewer #1: Yes

Reviewer #2: Yes

5. Review Comments to the Author

Please use the space provided to explain your answers to the questions above. You may also include additional comments for the author, including concerns about dual publication, research ethics, or publication ethics. (Please upload your review as an attachment if it exceeds 20,000 characters)

**Reviewer #1:** This paper aims to map and analyse the existing published research related to COVID-19 by researchers with a LAC affiliation and contribute to identify opportunities for strengthening future research. This is novel and relevant research, but I have many comments relate to methods and interpretation of findings. I think the main finding that “LAC countries have contributed to the global research effort primarily with epidemiological studies, with little participation on vaccines, meaning that this type of knowledge would be imported from elsewhere.” is important. However, it needs to be contextualised with discussions about what types of infrastructures, resources and labs would be needed in LAC to produce vaccines.
Many thanks for this comment. Although the main objective of the study was not to provide new information about what types of infrastructures, resources and labs would be needed in LAC to produce vaccines, we have made an effort to include such points in the discussion with more detail and citing papers where readers could be redirected to learn more about it.

Comments:

• Line 58-59: “The pandemic seems more difficult to bring under control comparing with other parts of the world, considering that the LAC region has particularly high levels of inequality and labour informality”. Compared with what? Africa? High income countries? Please elaborate as it is crucial to understand in your introduction why it is relevant to study covid-19 research in LAC.
We have updated to late October 2021 the information regarding cases and deaths of the COVID-19 pandemic in LAC compared to the world, which makes it clearer to understand the disproportionate impact in the region. In addition, we have mentioned that the socioeconomic differences in LAC are compared to other regions of the world.

• 108: “Articles in any language were eligible”. How did you compare topics?
We have a team that speaks all the languages of the studies that were screened (Spanish, English, Portuguese, French), so there was not a problem to compare topics.

• 117: “the research must be directly or explicitly connected to the current COVID-19 pandemic.” You need to explain to the reader that you used a query-based approach to find articles and also how you found the terms that you used in the query.

The full search strategy is included as part of the Supplementary information. We have included now in the text the rationale for the terms used in the query.

• 126: Brilliant that you were able to combine several bibliographic databases beyond MEDLINE. However, we need to know what is the proportion of articles from each database, and even better what is the percentage of overlap between them.

We have added this information as part of the PRISMA diagram on Figure 1.

• Figure 2: Why is there a decline after July? It would make more sense to have a constant rise until November. Is there a reason related to your process of data collection?

The data collection process was exactly the same for the whole time period covered. It could be due to a seasonal behaviour, but this is something that we cannot respond with our study.

• Figure 3: Please improve image quality. Also, “Brazil concentrated most of inclusions in publications with 54.6% of the 199 total, followed by Mexico (19.1%), Colombia (11.2%), Argentina (10.4%), Peru (10.3%), and Chile (10%), while Caribbean countries concentrated 15.3% of studies.” Would be relevant to compare these shares with shares of total medical research in LAC or total research in general. Is it possible to add all LAC research in the platforms you used and make the comparison?
Please see also: <https://unesdoc.unesco.org/in/documentViewer.xhtml?v=2.1.196&id=p%3A%3Ausmarcdef_0000377433&file=/in/rest/annotationSVC/DownloadWatermarkedAttachment/attach_import_a8477af4-1d6a-442f-af2f-7e77b02e5c31%3F_%3D377433eng.pdf&updateUrl=updateUrl7576&ark=/ark%3A/48223/pf0000377433/PDF/377433eng.pdf.multi&fullScreen=true&locale=en#page=222>
In the Discussion we provide comparisons with previous studies analysing research production in LAC in diverse areas such as oncologic clinical trials, pharma RCTs, and clinical trials in general.
It is not possible to add all LAC research in the databases we searched, because this would require conducting a new review with different objective and methods.

• Figure 4: This figure appears in the document without any reference in the text. Although the quality of the image is very low, this is one of the most relevant analytical pieces in this article in my opinion. You need to explain how you applied “modified version of the WHO Coordinated Global Research Roadmap classification of COVID19 research” and discuss better your results.

We have added the reference to the new Figure 3 in the text (previously Figure 4). Thank you for noticing this omission.
In Methods section we describe how we applied the modified version of the WHO classification, and in the Discussion section we provide several citations to the findings of this analysis, including some critics to the original WHO classification. It is not clear for us how we could better discuss the results, after reading your comment.

• Table 2. It is relevant for readers need to know which of these funding sources are national, LAC or foreign. You might consider using a different kind of classification to do so: 1) LAC government/public funding; 2) Non-LAC public Funding (e.g. NIH); 3) Multilateral funding (e.g. WHO); 4) Philanthropic funding (e.g. Gates Foundation) and 5) Corporation funding. It would be interesting to know also what the biggest individual funders are and in what thematic areas they specialise.
Unfortunately, we did not extract the information from the included studies to create this newly proposed classification for funding sources. In order to do it, we would require extracting and analyse it from the 1,626 included studies, which would significantly delay the publication. In addition, the main difference of the proposed classification with the one we have in the paper now relates to the LAC and non-LAC public funding – all the other sources are currently present in our classification.

• 247: “This number is difficult to compare with other regions as there are not many studies exploring this issue.” I am sure you will find several articles doing bibliometric analysis to COVID-19 research across the globe. Maybe not only looking at “empirical research” as you did but just using a “covid-19” query and analysing articles in PUBMED or WoS. Some examples:

• <https://www.ncbi.nlm.nih.gov/pmc/articles/PMC7174863/>

• <https://www.scielo.br/j/spmj/a/5SyhpMcdW6RXpNnxGq88wVD/?lang=en>

Many thanks for providing more references that relates to our review. We have included these papers in the introduction in the same vein as the three studies we cited signalling that COVID-19 production has been low in general terms in LAC. However, the point we make in the Discussion is that this review has the additional value of review empirical research along with several of its characteristics, and this is what is difficult to compare. We have made adjustment in the wording to show this more clearly.

• 265: “Remarkably, 198 studies for candidate therapeutics and only 11 studies exploring candidate vaccines were found, signaling a relatively low participation of LAC population in this type of research.” Interesting, but compared to what? It seems a relatively high ratio when compared to Guleid et al (2021). Please find more literature that contextualizes this finding, or you need to do an international comparison to know if this is a low or high number.

We have modified this sentence to say that this is a relatively low proportion of all empirical studies identified at the time when the present review was conducted. Therefore, we are not comparing with other studies, because it was not the aim of our study and it was a difficult task with the information available worldwide back in November 2020.

• 277: “Regarding funding sources, close to half of studies (47.5%) did not report their funding provenance. This is higher than what has been reported in previous research, for instance related to health policy and systems research(21) and clinical trials(22) where 31% and 11% of studies, respectively, did not include funding statements.” I wonder if this difference is due to the type of bibliographic databases that you use to collect articles. Can you compare MEDLINE vs other databases? I might be wrong, but I think fewer studies in MEDLINE will not report funding.
Medline does not impose different rules in terms of funding reporting than other databases. Therefore, there is no reason to expect finding differences in terms of databases where each study was found.

• What about institutional research collaboration? What are the institutions producing more research in LAC and who are the main collaborators? Affiliation data can provide you with evidence about that.

We did not include authors affiliation as part of the extraction data as this was not part of the review objective. Thus, we cannot conduct this type of analysis. In order to do it, we would require extracting it and analyse it from the 1,626 included studies, which would significantly delay the publication.

**Reviewer #2:** First of all, I want to congratulate the authors of this research because COVID-19 pandemic is a very complex issue that deserves much attention and the goals of the article are both very relevant, particularly the second one ("contribute to identify opportunities for strengthening future research") and may support future decision making. I like the approach of the authors and the document is very well written and supported with references to other high quality publications.
Many thanks for your kind comments.

Minor comments

1. In the section of eligibility criteria, I noticed Paraguay is missing in the list of countries. Not sure if this was a mistake or the country was excluded from the scope of the study.
Many thanks for noticing this. It is a mistake and we have included Paraguay in the list.

2. I would suggest that tables present data sorted by some criteria. For example, table 2 could be sorted by % of the number of publications (descent)
We have made the changes in the two tables accordingly.

3. line 238 says "The majority of papers ... (47.5%) ...", I would suggest to rewrite it as "Almost half of the papers ..."
Correction made.

4. A question about funding sources (table 2): what does "none" and "Not reported" suggest to the authors? Does it mean that people do research in their free time? Is it un-paid research? Any idea?
We cannot really know. We can assume that the alternative for “none” are un-paid research or research conducted during academic hours that are not considered to be funding a specific paper. For the not reported, it could be the same or the authors omitted to disclose the information for reasons we do not know. Since there is a large uncertainty on this, we prefer not to include a statement about this in the paper.

5. 278 "... higher than what has been reported ..." . Shouldn't it say "... higher than what has NOT been reported"?
We are referring to other studies reporting on funding sources disclosure, so we have kept the sentence the same.

Major comments

1. About the period of the scoping review. This study analyzes articles published between 12/2019 and 11/2020, and I understand that the cut-off date depends on the dates in which this study has been conducted. However, this pandemic has had several waves with different spreads and impacts in each country (i.e. some countries managed it well at the beginning but lost control later, others were unable to content the spread of the virus from the beginning, etc) and research communities have reacted at different speeds (some countries were faster and some ones were slower). Questions for the team: would it be possible that you extend the cut-off date of the scoping review in order to include a few more months, so you can include more research papers? and see how research evolved over time, controlling for different variables (country, research questions, methods, funding). If you can't do this, I strongly suggest that (if possible) you conduct a second study, with the same approach and data sources, for the period 12/2020 to 9/2021. This second study would help understand research focus and efforts during vaccination campaigns in LAC countries, and this would help understand other relevant questions about the pandemic.
We would love to expand the time scope of the review, but this would require a large amount of extra work, time, and resources, which will substantially delay this publication. As suggested by the reviewer, such an effort would require conducting a second study (an update of the review), which would lead to a separate paper. As authors of this first review, we certainly encourage such an effort to complement what we are presenting here.

2. 293 says "LAC countries can use the findings of this review to ...". Here, it is extremely important that the authors suggest which institutions in the countries could use these findings (i.e. Ministries of Health and Social Protection, Ministries of Science and Technology, ...who?)

We have specified what actors in LAC can benefit from the information coming out from the review.

3. 297-300 "There is also a need to build capacity to have more flexible research production in order to act fast in responding to public health emergencies such as pandemics, which can be readily translated to decision making at different levels of health and social systems (28)". I agree with this statement but here the key questions are: who could/should decide and change priorities about what research matters?, and where should this capacity be built? Could you elaborate on these?

We have provided further discussion to the fact multilateral collaboration between international organisations, governments and research actors can help to better understand the R&D environment in LAC and generate information as public goods to foster health research improvements.

4. The paper presents several very relevant findings and recommendations that may foster thinking, discussion and inform future science policies. For example, along the paper, the authors write:
in 50-51 "Research agendas could be further coordinated aiming to enhance shared self-sufficiency regarding knowledge needs in the region"
in 89-91 "research funding has also been questioned in terms of the distribution and sources for achieving a fair and sustainable research and development environment (13)"
in 91-94 "In LAC, a study searching for COVID-19 trials on treatment and prevention in the region identified “a trend towards small repetitive non-rigorous studies that duplicate efforts and drain limited resources without producing meaningful conclusions on the safety and efficacy of the interventions being tested (14)."
in 264-266 regarding a relatively very low research effort in candidate therapeutics and candidates vaccines
in 266-267 "This is more worrying due to the fact that RCTs protocols for such studies have also been found to be of low quality and potential waste of research resources (14)"
in 283-286 "In LAC, these findings could highlight opportunities to collaborate and create synergies between public and academic funding sources, where national health research agendas could help to align priorities and efforts (23)"

These six comments/findings are extremely relevant and could be the basis (or part of) to respond to the second aim of the study (contribute to identify opportunities for strengthening future research). However, I don't find a section in the paper that responds to that aim in a clear and comprehensive way, proposing recommendations for such contribution. I strongly suggest the team to elaborate on this. And it would also help an aspirational tone in such recommendations, if they want the audience to take action after reading the paper.

Many thanks for this very relevant comment. We have created a new subsection in the Discussion called “Policy considerations” where we now have grouped all the policy implications of our findings, putting them in context. We have also expanded the section with updated information on initiatives related to the issue.

6. PLOS authors have the option to publish the peer review history of their article ([what does this mean?](https://journals.plos.org/plosone/s/editorial-and-peer-review-process#loc-peer-review-history)). If published, this will include your full peer review and any attached files.


If you choose “no”, your identity will remain anonymous but your review may still be made public.


**Do you want your identity to be public for this peer review?** For information about this choice, including consent withdrawal, please see our [Privacy Policy](https://www.plos.org/privacy-policy).

Reviewer #1: No

Reviewer #2: **Yes:** Rafael Anta
