# Supplementary material for: Characterising COVID-19 empirical research production in Latin America and the Caribbean: A scoping review
Source: PLoS One. 2022 Feb 16;17(2):e0263981. doi: 10.1371/journal.pone.0263981 (PMC8849471; doi:10.1371/journal.pone.0263981)
Supplement: Attachment — Submitted filename: Response to Reviewers. PLOS ONE 3.0.docx [file pone.0263981.s005.docx]

PONE-D-21-25411

**Characterising COVID-19 empirical research production in Latin America and the Caribbean: a scoping review**

PLOS ONE

By Mansilla, Herrera, Boeira, et al.

*Response to Reviewers*

We thank the editor and reviewers for their new thoughtful revision of our manuscript. We respond to each of the editor and reviewer comments below, indicating where changes to the text and tables/figures can be found.

Journal Requirements:

Please review your reference list to ensure that it is complete and correct. If you have cited papers that have been retracted, please include the rationale for doing so in the manuscript text, or remove these references and replace them with relevant current references. Any changes to the reference list should be mentioned in the rebuttal letter that accompanies your revised manuscript. If you need to cite a retracted article, indicate the article’s retracted status in the References list and also include a citation and full reference for the retraction notice.

We have checked our reference list and there is no retracted article.

Additional Editor Comments:

Both reviewers appreciate your work and how you addressed their comments. One of them requires some additional minor revisions. Please follow her/his suggestions and resubmit the paper explaining how you addressed these changes. At this stage, the paper will not have to be resubmitted to the external reviewers again as I will review the changes made.

Many thanks. We provide responses to each comment below.

[Note: HTML markup is below. Please do not edit.]

Reviewers' comments:

Reviewer's Responses to Questions

**Comments to the Author**

1. If the authors have adequately addressed your comments raised in a previous round of review and you feel that this manuscript is now acceptable for publication, you may indicate that here to bypass the “Comments to the Author” section, enter your conflict of interest statement in the “Confidential to Editor” section, and submit your "Accept" recommendation.

Reviewer #1: (No Response)

Reviewer #2: All comments have been addressed

Many thanks.

2. Is the manuscript technically sound, and do the data support the conclusions?

The manuscript must describe a technically sound piece of scientific research with data that supports the conclusions. Experiments must have been conducted rigorously, with appropriate controls, replication, and sample sizes. The conclusions must be drawn appropriately based on the data presented.

Reviewer #1: Yes

Reviewer #2: Yes

Many thanks.

3. Has the statistical analysis been performed appropriately and rigorously?

Reviewer #1: Yes

Reviewer #2: Yes

Many thanks.

4. Have the authors made all data underlying the findings in their manuscript fully available?

The [PLOS Data policy](http://www.plosone.org/static/policies.action#sharing) requires authors to make all data underlying the findings described in their manuscript fully available without restriction, with rare exception (please refer to the Data Availability Statement in the manuscript PDF file). The data should be provided as part of the manuscript or its supporting information, or deposited to a public repository. For example, in addition to summary statistics, the data points behind means, medians and variance measures should be available. If there are restrictions on publicly sharing data—e.g. participant privacy or use of data from a third party—those must be specified.

Reviewer #1: Yes

Reviewer #2: Yes

Many thanks.

5. Is the manuscript presented in an intelligible fashion and written in standard English?

PLOS ONE does not copyedit accepted manuscripts, so the language in submitted articles must be clear, correct, and unambiguous. Any typographical or grammatical errors should be corrected at revision, so please note any specific errors here.

Reviewer #1: Yes

Reviewer #2: Yes

Many thanks.

6. Review Comments to the Author

Please use the space provided to explain your answers to the questions above. You may also include additional comments for the author, including concerns about dual publication, research ethics, or publication ethics. (Please upload your review as an attachment if it exceeds 20,000 characters)

Reviewer #1: I am ok with the authors answers and changes.

However, I am unconvinced that decline of covid-19 publications from July onwards is a natural phenomenon. Please compare it with other studies in other regions to understand if this decline also appears in different regions. And please make a footnote in figure 2 saying that the month of November is incomplete.

We have double checked the study we cite in the paper on research production in Africa (Guleid et al. 2021 (18)), along with other bibliometric analysis we found now on COVID19 publications in Asia ([Tantengco OAG 2021](https://www.sciencedirect.com/science/article/pii/S1871402121003453)) and in specific fields such as vaccines research ([Ahmad T et al. 2021](https://www.tandfonline.com/doi/full/10.1080/21645515.2021.1886806)) and older adults research ([Soytas RB 2021](https://www.e-agmr.org/journal/view.php?doi=10.4235/agmr.21.0060)). Unfortunately, none of these studies makes a timeline analysis of the number of studies published during the pandemic. However, we would like to precise that we report that the total number of empirical studies published in LAC per month reached a peak of 252 in July, then remained relatively stable in 234 in August and 233 in September, and only in October it went down to 190. Since we don’t have the data for November onwards, we cannot really ascertain much about a significant decline in the number of publications. Therefore, we believe it would be better not to mention this issue in the paper as we don’t have enough information to make a well-grounded statement regarding a significant decline in the number of empirical studies being published per month from July onwards.

We have added the suggested footnote in Figure 2 to make it more clear for the reader.

Also, please reorder the categories of figure 3 for the categories to appear as a ranking (top category with more pubs 1st, etc.)

We have modified Figure 3 as suggested by Reviewer #1. Many thanks for this comment.

Reviewer #2: I read the new version of the paper, and also the responses of the authors to my comments from the previous review and I found all were addressed. Again, congratulations to the authors for such a good paper that will help promote thinking a discussion about the need for empirical research on COVID-19, and most important: how to do more and better with little resources. Coordination, collaboration and synergies will be key for research in the next future.

Many thanks. We fully agree with the comments of Reviewer #2 and hope our paper can contribute along the lines mentioned.

7. PLOS authors have the option to publish the peer review history of their article ([what does this mean?](https://journals.plos.org/plosone/s/editorial-and-peer-review-process#loc-peer-review-history)). If published, this will include your full peer review and any attached files.

If you choose “no”, your identity will remain anonymous but your review may still be made public.

**Do you want your identity to be public for this peer review?** For information about this choice, including consent withdrawal, please see our [Privacy Policy](https://www.plos.org/privacy-policy).

Reviewer #1: No

Reviewer #2: **Yes:** Rafael Anta
